# The global, regional, and national burden of cancer among adolescents and young adults in 204 countries and territories, 1990–2019: a population-based study

**DOI:** 10.1186/s13045-021-01093-3

**Published:** 2021-06-09

**Authors:** Yi Feng Wen, Meng Xuan Chen, Guosheng Yin, Ruitao Lin, Yu Jie Zhong, Qian Qian Dong, Hai Ming Wong

**Affiliations:** 1 Key Laboratory of Shaanxi Province for Craniofacial Precision Medicine Research, College of Stomatology, Xi’an Jiaotong University, Xi’an, China; 2 Department of Statistics and Actuarial Science, The University of Hong Kong, Pok Fu Lam, Hong Kong; 3 Department of Biostatistics, The University of Texas MD Anderson Cancer Center, Houston, USA; 4 Department of Pulmonary and Critical Care Medicine, The Second Affiliated Hospital of Xi’an Jiaotong University, Xi’an, Shaanxi China; 5 Paediatric Dentistry and Orthodontics, Faculty of Dentistry, The University of Hong Kong, 34 Hospital Road, Pok Fu Lam, Hong Kong

**Keywords:** Adolescents and young adults, Global burden of disease study, Cancer, Prevalence, Mortality

## Abstract

**Background:**

Accurate appraisal of burden of adolescents and young adults (AYAs) cancers is crucial to informing resource allocation and policy making. We report on the latest estimates of burden of AYA cancers in 204 countries and territories between 1990 and 2019 in association with socio-demographic index (SDI).

**Patients and methods:**

Estimates from the Global Burden of Disease study 2019 were used to analyse incidence, mortality, and disability-adjusted life years (DALYs) due to AYA cancers at global, regional, and national levels by sex. Association between AYA cancer burden and SDI were investigated. Burdens of AYA cancers were contextualized in comparison with childhood and older adult cancers. All estimates are reported as counts and age-standardized rates per 100,000 person-years.

**Results:**

In 2019, there were 1.2 million incident cases, 0.4 million deaths, and 23.5 million DALYs due to AYA cancers globally. The highest age-standardized incidence rate occurred in Western Europe (75.3 [Females] and 67.4 [Males] per 100,000 person-years). Age-standardized death (23.2 [Females] and 13.9 [Males] per 100,000 person-years) and DALY (1328.3 [Females] and 1059.2 [Males] per 100,000 person-years) rates were highest in Oceania. Increasing SDI was associated with a higher age-standardized incidence rate. An inverted U-shaped association was identified between SDI and death and DALY rates. AYA cancers collectively is the second leading cause of non-communicable diseases-related deaths globally in 2019. DALYs of AYA cancers ranked the second globally and the first in low and low-middle SDI locations when compared with that of childhood and older adult cancers.

**Conclusion:**

The global burden of AYA cancers is substantial and disproportionally affect populations in limited-resource settings. Capacity building for AYA cancers is essential in promoting equity and population health worldwide.

**Supplementary Information:**

The online version contains supplementary material available at 10.1186/s13045-021-01093-3.

## Introduction

Cancer, as a major disease cluster of noncommunicable diseases, is a global health problem affecting populations of all ages [1]. Despite global commitment to reducing burden of cancers, a recent United Nations high-level meeting on noncommunicable diseases indicated inadequate progress towards achieving Target 3.4 of their Sustainable Development Goal [2, 3].

The present cohort of over 2.9 billion adolescents and young adults (AYAs) aged 15–39 years is the largest in history, among which nearly 90% live in low-income and middle-income countries (LMICs) [4, 5]. With 1.2 million cases and 0.4 million deaths attributable to cancers among AYAs in 2018 [6], addressing AYA cancers provides an unequivocal opportunity to accelerate the achievement of development goals and promote equity. AYA cancer patients have many unique characteristics that may influence therapy outcomes, including distinct cancer biology [7], delays in diagnosis [8], high uninsured rate [9], low accrual to clinical trials [10], and high risk of long-term and late effects of treatment [11]. Unfortunately, healthcare needs of AYA cancer patients have been historically neglected due to the enormous burden of older adult cancer and rising attention towards paediatric oncology [12]. Accurate appraisal of the global burden of AYA cancers is an important first step towards improving outcomes of AYA cancers.

Two studies have been conducted to address the scale and trend of AYA cancers worldwide. However, several limitations are worth noting in those studies [13, 14]. First, metrics used were limited to incidence and mortality. Second, trend analysis was restricted to incidence and was limited to a selected number of countries. Third, cancer epidemiology was not adequately addressed in association with socio-demographic development. Fourth, data in these studies were outdated (collected up to 2012). These limitations impeded a comprehensive understanding and vivid contextualization of the burden of AYA cancers. Miller and colleagues provided a detailed analysis of the trends in the burden of AYA cancers over the past decades [15]. However, the analysis focused on data from the US only. As efforts to expand cancer surveillance systems are still underway in many countries, the Global Burden of Disease (GBD) study, which provides authoritative model-based estimates of disease burden, is uniquely positioned to fill in the current data gap [16, 17]. In this dedicated analysis of the most recent data from the GBD 2019 study, we describe burden of AYA cancers in 2019, highlight trends of AYA cancers from 1990 to 2019, investigate its association with socio-demographic development, and contextualize AYA cancer burden by comparing with childhood and older adult cancers. Our findings are of great value in informing global and national resource allocation and policy prioritization to better address the unmet needs of AYA cancer patients.

## Methods

### Overview

Cancer cases among AYAs at the global, regional, and national levels were ascertained from the GBD 2019. The burden of 369 diseases and injuries, 286 causes of death, and 87 risk factors were estimated globally, for 21 GBD world regions, and for 204 countries and territories, by year, age-group, and sex in the GBD 2019. The GBD 2019 classified cancers into 29 groups according to the International Classification of Diseases 10th edition (ICD-10). Mapping of ICD codes to 29 cancer groups has been described previously [16]. Estimates are available for all ICD 9 codes pertaining to cancer and ICD 10 codes except for Kaposi sarcoma (ICD 10: C46) in the GBD 2019. Estimates of incidence, mortality, years lived with disability (YLDs), years of life lost (YLLs), and disability-adjusted life years (DALYs) by cancer types are the primary measures of cancer burden in the present analysis. The present study is reported in compliance with the Guidelines for Accurate and Transparent Health Estimates Reporting statement (Additional file 1: Appendix pages 1–2) [18]. Data for this study were retrieved from the Global Health Data Exchange query tool [19].

### Estimation of cancer burden

A detailed methodology of the GBD 2019 has been presented in a series of capstone publications [15, 17, 20–22]. References to these publications were made where relevant to the present study (Additional file 1: Appendix pages 3–5). Measures of disease burden used in this study are herein briefly introduced. Observed mortality data were collected from vital registration and verbal autopsy. For locations where observed mortality data are unavailable, estimated mortality were used. Estimated mortality data were derived by multiplying high-quality cancer registry incidence data by the corresponding, independently modelled, mortality-to-incidence ratio (MIR). MIRs were estimated through linear-step mixed-effects models with a logit link function and were smoothed and adjusted with spatiotemporal Gaussian process regression [16]. Both the observed and estimated mortality data were submitted to a Cause of Death Ensemble model [17] to obtain modelled mortality estimates.

Estimates of incidence of each cancer type were obtained by dividing modelled mortality estimates by MIR. For estimation of YLDs, the total prevalence was divided into phases of cancer treatment (sequelae), and then YLD estimates were calculated as the weighted sum of sequelae prevalence, where sequelae prevalence was estimated from cancer survival data modelled on the basis of MIR [16]. Sequela-specific disability weight ranged from 0 (full health) to 1 (death) and values have been defined for each cancer type previously [16]. YLLs were estimated as the weighted sum of the number of deaths by age, where the weights were the standard life expectancy at each age-group of deaths [16]. DALYs were calculated as the sum of YLDs and YLLs.

### Socio-demographic Index (SDI)

SDI is a composite indicator of development status which is strongly correlated with health outcome. It integrates the total fertility rate under the age of 25 (TFU25), mean education for those aged 15 and older (EDU15 +), and lag distributed income (LDI) per capita. SDI ranges from 0 (the theoretical minimum level of development) to 100 (the theoretical maximum level of development) and values for each country by year can be retrieved from the GBD 2019 capstone papers [17].

### Data analysis

The AYA age-group in this study encompasses 15- to 39-year-olds as defined traditionally by AYA oncology [9, 23]. Only malignant neoplasms were included for analysis. Different types of leukaemia were analysed separately due to differences in treatment options and prognosis [24, 25]. Non-melanoma skin cancers were not analysed. In this study, data on burden of 32 cancer types among AYAs were extracted from the GBD 2019 study at global, regional, and national scales, for both sexes, from 1990 to 2019. Cancer data extracted from the GBD 2019 were in the format of 5-year age-groups (15–19-, 20–24-, 25–29-, 30–34-, and 35–39-year-olds).

Absolute numbers of incident cases, deaths, YLDs, YLLs, and DALYs were calculated as the sum of the corresponding values from all relevant 5-year age-groups, from 15–19 years to 35–39 years. Truncated age-standardized rates were calculated as $\frac{\sum_{i=1}^{A}{a}_{i}{w}_{i}}{\sum_{i=1}^{A}{w}_{i}}\times 100{,}000$ [26], where ${a}_{i}$ denotes the age-specific rate for the *i*th age-group, ${w}_{i}$ represents weights for the *i*th age-group calculated from the GBD 2019 world standard population [17], and $A$ is the total number of age-groups, which is 5 in our case.

Results were presented at the global, regional, and national levels for all AYA cancers collectively and by cancer types. LOWESS regression was performed to illustrate the association between SDI and age-standardized incidence, death, and DALY rates. Coefficient of determination (${R}^{2}$) was estimated from the LOWESS model to reflect the proportion of variation in burden of AYA cancers attributable to variations in SDI. To contextualize disease burden, AYA cancer-related deaths were compared with deaths from other diseases, by SDI quintile. In addition, AYA cancers were compared with cancers of children and older adults with respect to incident cases, deaths, and DALYs, globally and by SDI quintile.

Because of variations in data availability and quality, uncertainty exists for measures of cancer burden. The 95% uncertainty intervals (95% UIs), specified on the basis of the 2.5th and 97.5th percentile of the distribution of 1000 draws during Bayesian modelling, were extracted along with point estimates of measures of cancer burden whenever possible. It should be noted that due to methodological issues relating to the estimates of age-standardized rates for 15–39 year-olds (Additional file 1: Appendix pages 6–7), age-standardized rates for AYAs were calculated manually. Although this generated estimates of AYA cancer burden measures that are better suited for investigation of temporal changes, a limitation is that we were unable to generate 95% UIs since data on the 1000 draws during Bayesian modelling have not been made publicly accessible. The 95% confidence intervals (95% CIs) were used to quantify the magnitude of uncertainty of the association between SDI and cancer burden measures.

## Results

Absolute numbers and age-specific rates of incident cases, deaths, and DALYs of AYA cancers in 2019, together with associated 95% UIs, are presented in 5-year age-groups in Additional file 1: Tables S1–S5. In 2019, there were 1.2 million incident cases, 0.4 million deaths, and 23.5 million DALYs due to AYA cancers worldwide (Table 1). Age-standardized rates were 39.7, 13.2, and 782.3 per 100,000 person-years in terms of incidence, deaths, and DALYs, respectively. In 2019, AYA cancers collectively was the second leading cause of non-communicable disease-related deaths globally and across all SDI quintiles (Additional file 1: Table S6). Percentage of non-communicable disease-related deaths due to AYA cancers was higher in middle (28.9%), high-middle (31.9%), and high (26.1%) SDI locations than in low (21.6%) and low-middle (22.4%) SDI locations.

**Table 1:** Burden of AYA cancers and percentage change in age-standardized rates from 1990 to 2019

	Incidence			Deaths			DALYs		
	Number of incident cases, 2019	Age-standardized incidence rate (per 100,000 population), 2019	Percentage change in rates, 1990–2019	Number of deaths, 2019	Age-standardized death rate (per 100,000 population), 2019	Percentage change in rates, 1990–2019	Number of DALYs, 2019	Age-standardized DALYs rate (per 100,000 population), 2019	Percentage change in rates, 1990–2019
Global	1,194,382	39.7	10.7	396,117	13.2	− 21	23,491,284	782.3	− 20
Female	685,639	45.9	9.5	202,452	13.6	− 20.9	11,973,819	804.1	− 19.9
Male	508,743	33.5	12	193,665	12.8	− 21.2	11,517,464	760.4	− 20.2
High SDI	213,165	59.6	5.2	33,370	9.2	− 33.7	2,017,829	564.4	− 32
High-middle SDI	301,329	53.1	30.1	76,612	13.4	− 28.4	4,515,373	801.5	− 27
Middle SDI	355,636	36.9	16.5	131,819	13.7	− 25.4	7,783,143	810.2	− 24.1
Low-middle SDI	207,656	29.2	16.5	100,583	14.2	− 5.1	5,968,398	836.8	− 4.8
Low SDI	97,858	24.3	3.9	53,478	13.3	− 3.7	3,191,436	781.2	− 3.2
Acute lymphoid leukaemia	38,747	1.3	66.6	11,696	0.4	− 10.6	765,511	26.1	− 9.9
Acute myeloid leukaemia	20,183	0.7	5.6	12,166	0.4	− 0.1	747,561	25.2	− 0.2
Bladder cancer	14,095	0.5	29	2050	0.1	− 22.6	124,475	4.1	− 20.2
Brain and central nervous system cancer	61,511	2.1	23.9	29,105	1	− 6.9	1,746,906	58.4	− 6.6
Breast cancer	169,859	5.6	25.7	43,088	1.4	− 4.7	2,489,420	82.1	− 2.8
Cervical cancer	119,258	3.9	− 4.2	27,168	0.9	− 24.3	1,560,541	51.4	− 23.7
Chronic lymphoid leukaemia	4263	0.2	113.4	1010	0	1.7	61,375	2.6	4.3
Chronic myeloid leukaemia	9203	0.3	− 16	4956	0.2	− 33.1	294,861	9.8	− 33
Colon and rectum cancer	76,090	2.5	38.8	28,352	0.9	− 2.7	1,633,410	53.9	− 2.1
Esophageal cancer	8088	0.3	− 23.9	6215	0.3	− 29.7	343,752	14.5	− 29.4
Gallbladder and biliary tract cancer	3841	0.2	− 10.5	2390	0.1	− 17.1	132,562	5.6	− 17
Hodgkin lymphoma	33,388	1.1	− 8.6	8093	0.3	− 34.3	508,216	17.1	− 33.3
Kidney cancer	21,136	0.7	62.9	4017	0.1	20.7	239,153	7.9	22.1
Larynx cancer	4214	0.2	− 15.4	2250	0.1	− 29.3	128,189	5.4	− 28.7
Lip and oral cavity cancer	29,440	1	29.9	10,043	0.3	19.3	580,333	19.2	19.4
Liver cancer	25,430	0.8	− 50.9	18,573	0.6	− 57.5	1,046,876	34.6	− 57.1
Malignant skin melanoma	37,266	1.2	16.3	4248	0.1	− 23.9	259,089	8.6	− 21.6
Mesothelioma	1466	0.1	− 17.8	990	0	− 16.1	56,365	2.4	− 16.2
Multiple myeloma	2931	0.1	35.9	1680	0.1	19	95,563	4	20.3
Nasopharynx cancer	28,562	0.9	57.8	6079	0.2	− 41.9	362,911	12.1	− 40.6
Non-Hodgkin lymphoma	52,427	1.8	20.5	20,797	0.7	− 1.5	1,275,943	42.8	− 1.1
Other leukaemia	28,811	1	− 47.1	15,278	0.5	− 54.7	948,803	32	− 55.1
Other malignant neoplasms	141,110	4.8	28	51,453	1.7	2.3	3,230,198	109.1	3.3
Other pharynx cancer	7103	0.3	36.5	4360	0.2	21.6	244,994	10.4	21.8
Ovarian cancer	35,831	1.2	30.9	8896	0.3	12.8	529,030	17.6	13.8
Pancreatic cancer	9401	0.3	18.2	7609	0.3	16.2	420,797	13.9	16
Prostate cancer	5472	0.2	77.9	876	0	− 0.7	54,331	2.3	2.8
Stomach cancer	49,008	1.6	− 30.9	27,895	0.9	− 46.8	1,573,897	52	− 46.4
Testicular cancer	57,401	1.9	42	5352	0.2	− 3.9	349,083	11.7	− 0.6
Thyroid cancer	46,832	1.6	65.8	2853	0.1	− 0.6	190,923	6.4	4.7
Tracheal, bronchus, and lung cancer	32,601	1.1	− 24.1	24,772	0.8	− 29.4	1,386,001	45.8	− 29
Uterine cancer	19,415	0.8	14.9	1808	0.1	− 37.8	110,215	4.7	− 35

Absolute incidence, deaths, and DALYs represent the total AYA cancer (35–39 years, both sexes combined) values. Rates are reported per 100,000 person-years. Uncertainty intervals are not reported because draw level data are not available for this customized age-group. DALY = disability-adjusted life-year. SDI = Socio-demographic Index

**Fig. 1:**
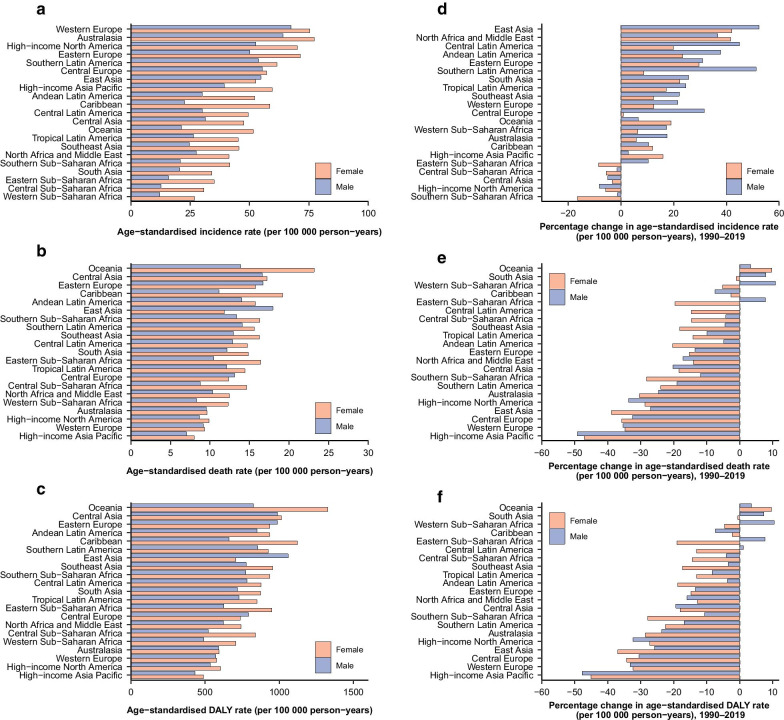
Levels and trends in burden of AYA cancers across 21 GBD regions by sex. The age-standardized incidence (**a**), death (**b**), and DALY (**c**) rates of AYA cancers in 2019. The percentage change in age-standardized incidence (**d**), death (**e**), and DALY (**f**) rates of AYA cancers from 1990 to 2019. GBD = Global Burden of Diseases, Injuries, and Risk Factors Study

In 2019, the region with the highest age-standardized incidence rate was Western Europe (75.3 [Females] and 67.4 [Males] per 100,000 person-years; Fig. 1a) and those with the lowest rates were in sub-Saharan Africa. Age-standardized death (23.2 [Females] and 13.9 [Males] per 100,000 person-years) and DALY (1328.3 [Females] and 1059.2 [Males] per 100,000 person-years) rates were highest in Oceania (Fig. 1b, c) and were lowest among high-income GBD regions except for Southern Latin America. Between 1990 and 2019, most world regions demonstrated increased AYA cancer incidence rates and decreased death and DALY rates (Fig. 1d–f). Further analysis of regions whose trends were at odds with global trends (Additional file 1: Table S7) suggested that decreased incidence in Eastern, Central, and Southern Sub-Saharan Africa were driven by reduction in cervical cancer.

**Fig. 2:**
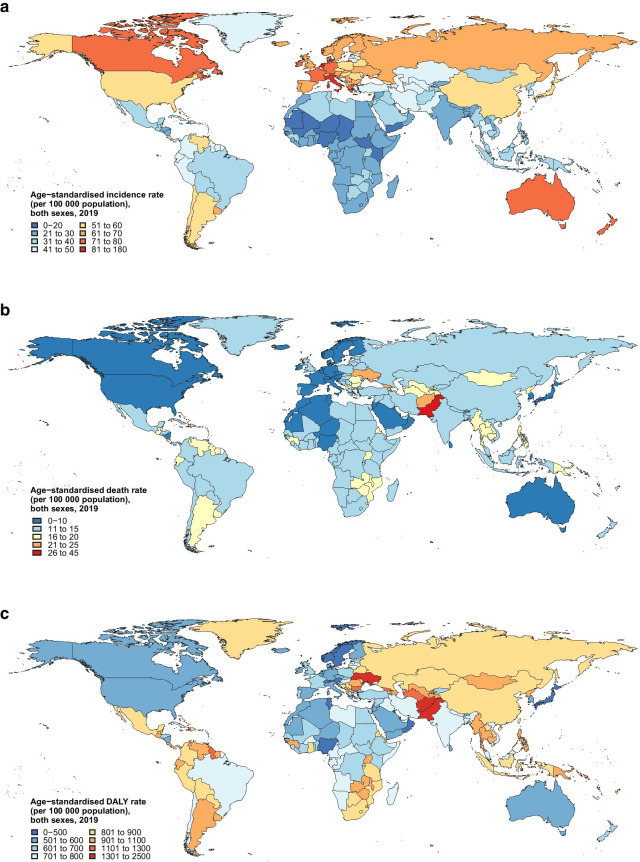
Burden of AYA cancers across 204 countries and territories in both sexes, 2019. Age-standardized rates of incidence (**a**), deaths (**b**), and DALYs (**c**) of AYA cancers

At the national level, the burden of AYA cancers in 2019 (Fig. 2) and associated percentage changes during 1990–2019 (Additional file 1: Fig. S1) varied widely by countries. The highest age-standardized incidence rate in 2019 was noted in Monaco (177.6 per 100,000 person-years) and the highest age-standardized death rate and DALY rate were noted in Solomon Islands (Death rate: 42.1 per 100,000 person-years; DALY rate: 2440.7 per 100,000 person-years). In 2019, uncategorized AYA cancers, collectively termed other malignant neoplasms in GBD, ranked top two in terms of the number of incident cases, deaths, and DALYs globally among 32 cancer types (Additional file 1: Tables S8–S10). In particular, other malignant neoplasms ranked highest globally and in 74 countries with regard to DALY burden (Additional file 1: Table S10).

**Fig. 3:**
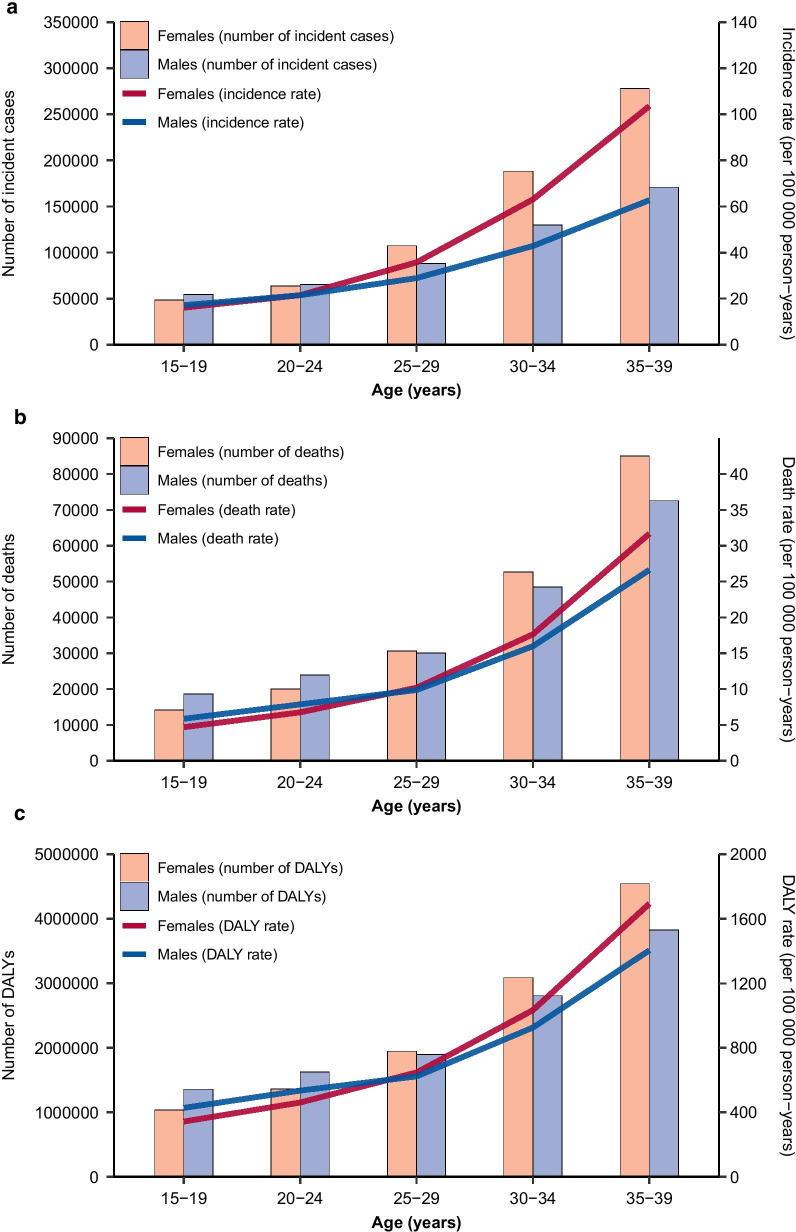
Burden of AYA cancers by age and sex, 2019. Global age-specific counts and rates of incident cases (**a**), deaths (**b**), and DALYs (**c**) of AYA cancers per 100,000 population by sex, 2019

**Fig. 4:**
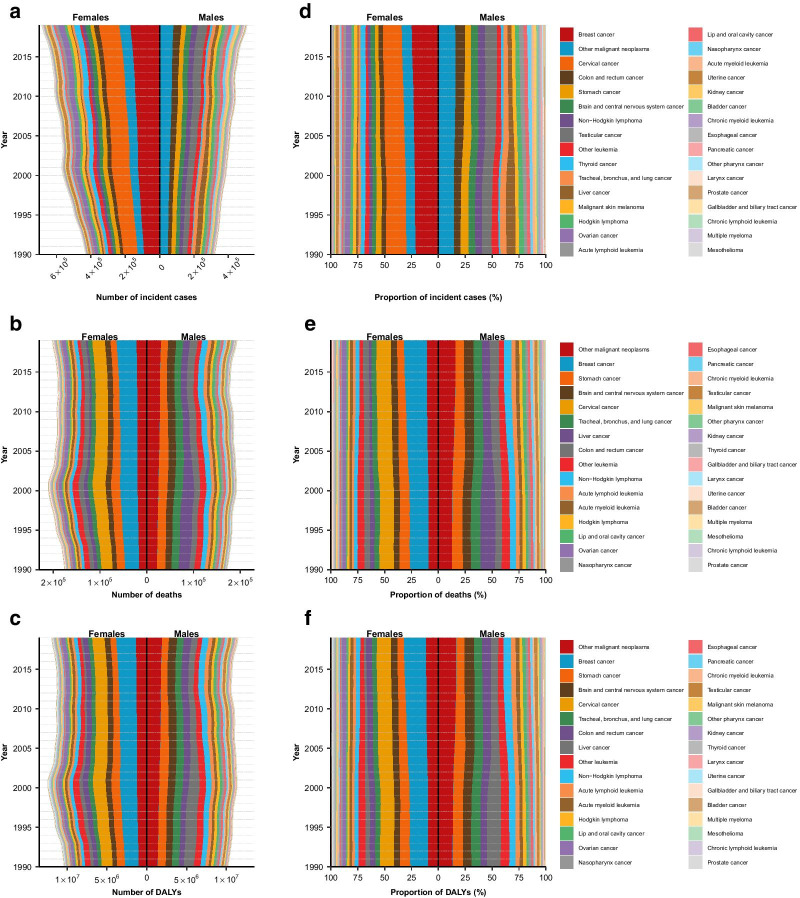
Global trends in absolute and proportional burden of incident cases, deaths, and DALYs by AYA cancer type and sex among 20- to 29-year-olds, 1990–2019. (**a**) Trends in the number of incident cases of AYA cancers by type. (**b**) Trends in composition of total incident cases of AYA cancers. (**c**) Trends in the number of deaths of AYA cancers by types. (**d**) Trends in composition of total deaths of AYA cancers. (**e**) Trends in the number of DALYs of AYA cancers by types. (**f**) Trends in composition of total DALYs of AYA cancers. AYA cancer types were sorted in decreasing magnitude in terms of the total number of incident cases, deaths, or DALYs among 15- to 39-year-olds for both sexes combined from 1990 to 2019

Absolute numbers and age-standardized rates for AYA cancer incidence, deaths, and DALYs increased progressively with age in both sexes (Fig. 3). Burden of AYA cancers in terms of all three measures concentrated increasingly with age among females. YLLs were disproportionally larger than YLDs across all AYA age-groups (Additional file 1: Fig. S2), accounting for 97.3% of the total DALYs associated with AYA cancers. Higher AYA cancer incidence among females was driven by the disproportionally large number of incident cases of breast cancer (Total number from 1990 to 2019: $3.8\times {10}^{6}$) and cervical cancer ($3.1\times {10}^{6}$, Fig. 4a, b). Combining through all AYA cancer deaths from 1990 to 2019, the highest proportions of categorized AYA cancer deaths and DALYs (Fig. 4b, c, e, f) were attributed to breast cancer (Deaths: 9.7%; DALYs: 9.4%), stomach cancer (8.8%; 8.3%), and brain and central nervous system cancer (7.0%; 7.1%). Other malignant neoplasms contributed the largest proportion of DALYs overall from 1990 to 2019 ($83.6\times {10}^{6}$, percentage: 12.4%). Age-related transitions in the spectrum of cancers that affect AYAs were evident (Additional file 1: Figs. S3–S5). Leukaemias, lymphomas, and brain and nervous system cancer contributed the greatest cancer burden among 15- to 19-year-olds, whereas breast cancer, cervical cancer, colon and rectum cancer, and stomach cancer were the major contributors of cancer burden among 30- to 39-year-olds. A mix of paediatric cancers and adult cancers was observed for 20- to 29-year-olds.

**Fig. 5:**
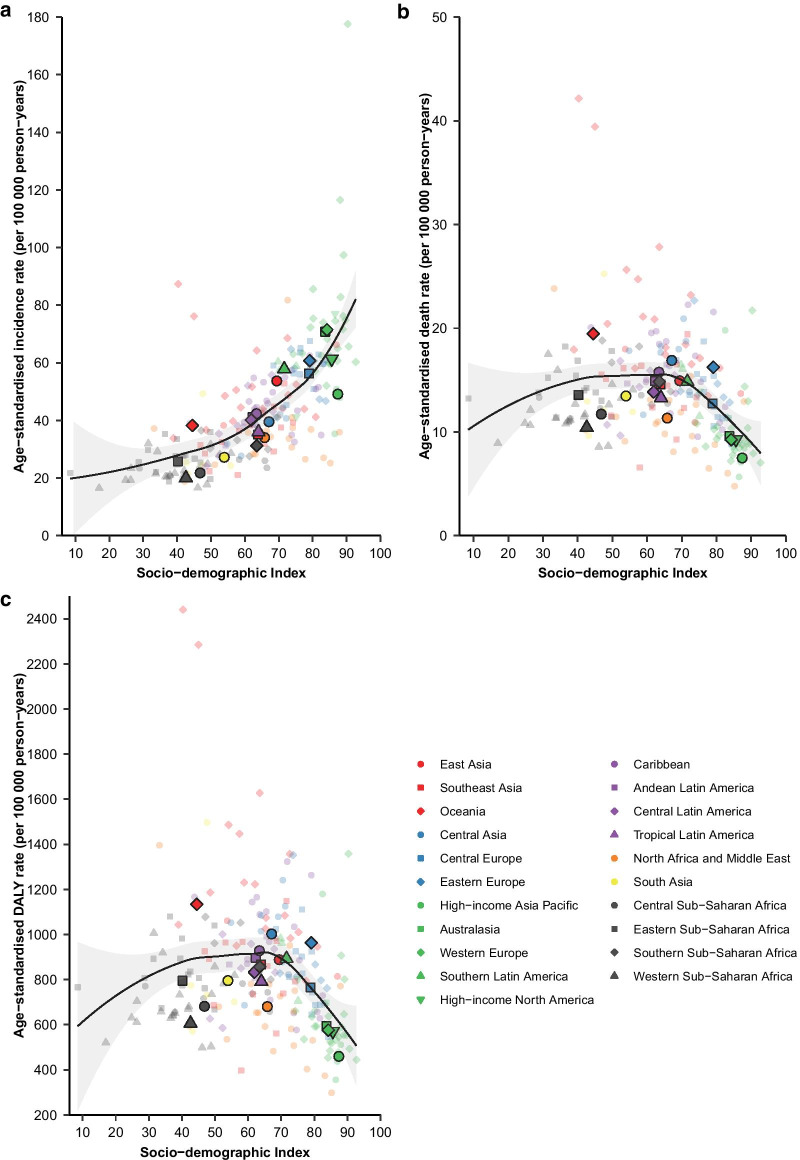
Association between SDI and AYA cancer burden at national level, both sexes combined, 2019. The association between SDI and the age-standardized incidence rate (**a**), death rate (**b**), and DALY rate (**C**) are depicted. Each colour represents one of the seven GBD super-regions. The small, lighter-coloured points represent countries. The larger, darker-coloured points represent GBD regions. The black lines indicate locally weighted smoothing estimates of age-standardized rates of relevant measures based on SDI in all countries. The grey shading around the black lines represents the 95% confidence interval of the estimated rates

Age-standardized incidence rates (Additional file 1: Fig. S6) increased as SDI increased (${R}^{2}$ = 82.9%). An inverted U-shaped association was observed between SDI and death (Additional file 1: Fig. S7, ${R}^{2}$ = 41.6%) and DALY (Additional file 1: Fig. S8, ${R}^{2}$ = 40.9%) rates of AYA cancers. Regional variations around expected incidence rates increased progressively as SDI increased. Death and DALY rates for high SDI locations clustered tightly around expected rates based on SDI, suggesting a close association of socio-demographic status and death and DALY rates of AYA cancers. The high incidence rates in high SDI locations are attributable to several factors other than socio-demographic status. In addition, relatively large year-to-year variation in burden of AYA cancers was noted in Southern Sub-Saharan Africa (Additional file 1: Figs. S6–S8). At national level (Fig. 5), in 2019, the associations between SDI and measures of the burden of AYA cancers were reminiscent of the associations at regional level (${R}^{2}$ = 47.9%, 16.1%, and 14.3% for age-standardized incidence, death, and DALY rate).

In 2019, compared with cancers of children and older adults, AYA cancers collectively ranked the sixth in terms of contribution to the total number of incident cases (Additional file 1: Table S11), the ninth in terms of deaths (Additional file 1: Table S12), and the second in terms of DALYs globally (Additional file 1: Table S13). It should be noted that all types of AYA cancers and childhood cancers were collectively treated as an entity to be compared with older adult cancers. Such comparisons aimed to provide an appreciation of the scale of AYA cancers collectively and should not be used to overemphasize the burden of AYA cancers. There was a substantial burden of AYA cancers among low and low-middle SDI locations. Numbers of AYA cancer incident cases and DALYs ranked the first in low and low-middle SDI locations. In addition, the number of AYA cancer deaths ranked the first in low SDI locations.

## Discussion

This study provides the most comprehensive and up-to-date evaluation on the global burden of AYA cancers from 1990 to 2019. This is the first study to use DALYs to quantify burden of AYA cancers. DALYs is a measure of disease burden that incorporates a lifelong perspective by taking into account both fatal and non-fatal components of disease burden. It can be used by policy makers in health system planning and policy prioritization. Based on DALYs, the global burden of AYA cancers in 2019 is substantial and is concentrated among LMICs.

AYA cancer incidence was found to be lower in low SDI settings. One plausible explanation is that cancer incidence tends to be underestimated in limited-resource settings due to potential misdiagnosis and missed diagnosis, inadequate infrastructures, and weak cancer surveillance systems [27]. The inverted U-shaped association between AYA cancer death and DALY rate and SDI is likewise associated with the potential underestimation of AYA cancer burden in socio-demographically less developed areas. However, global variations in genetic predispositions and temporal trends in lifestyles and environmental exposures may contribute as well. An increase in cancer incidence beyond that can be explained by improved reporting has been noted among European adolescents from the 1970s to 1990s [28]. Increased global cancer incidence over time likely reflects the combined effect of improved reporting and increased risk factor exposure. The magnitude of interactions between genetic, environmental, and non-medical factors remains unclear.

AYA cancer incidence rate decreased from 1990 to 2019 in Eastern, Central, and Southern Sub-Saharan Africa, largely driven by the decrease in cervical cancer. Cervical cancer is less strongly associated with HIV infection compared with other AIDS-defining cancers in Africa and its association with HIV is weaker in Africa than in Western countries [29]. Furthermore, it is unclear whether an increased risk of cervical cancer among women with AIDS is the result of immunosuppression or a higher risk of infection with human papillomavirus in the same population [30]. Therefore, progress in controlling HIV infection in Sub-Saharan Africa may not explain the sizable reduction in cervical cancer incidence. Elucidating reasons for this decline will suggest new opportunities of reducing burden of AYA cancers in Sub-Saharan Africa.

In the 2000s, the National Cancer Registry in South Africa was faced with a series of administrative challenges [31]. The relative contribution of administrative difficulties and other factors to the rapid decline in burden of AYA cancers during this period is difficult to be determined due to the historic lack of relevant data. Paying close attention to future burden of AYA cancers will reveal outcomes of the revitalization of the National Cancer Registry in South Africa in the 2010s [32]. Volume X of Cancer Incidence in Five Continents accepts data from eight cancer registries in Africa, which encompasses only 2% of its population [33]. There is a distinct need to strengthen the data surveillance system in LMICs to generate robust estimates from high-quality observed data from population-based cancer registries.

Improving the cancer surveillance system alone, however, will not resolve the high burden of AYA cancers in LMICs. Increasing cancer awareness through encouraging timely screening, increasing vaccination coverage, and reducing high-risk behaviours is essential given that an appreciable proportion of AYA cancers are preventable. While recommendations of breast cancer screening for AYAs are well in place, much less is known about the role of screening for colorectal cancer [34, 35]. The global burden of cervical cancer remains substantial. Cervical cancer awareness programmes aiming at increasing vaccination uptake and screening adoption, particularly in LMICs, are likely to contribute substantially in reducing global AYA cancer incidence. Promoting healthy lifestyles is particularly relevant in high-income countries and will become increasingly important in LMICs as these countries experience epidemiological transition from infectious to noncommunicable diseases [14, 27]. Policy restrictions on sunbed use in Australia have resulted in precipitous declines in malignant skin melanoma [14]. Lifestyle changes which ameliorate outcomes of obesity will be essential to controlling the rising obesity-related cancer burden among young adults [36]. In addition, greater cancer advocacy is needed to garner support for AYA cancers as a high priority. Redirecting health budgets and donor interest towards AYA cancers is essential in sustaining capacity-building activities. Improvements of healthcare systems in LMICs are urgently needed, such as infrastructural development and retention of trained physicians [27]. In high-income countries, a higher percentage of AYA deaths are due to neoplasms. Continuously innovating the healthcare system by fully integrating research into clinical pathways will assist in addressing treatment-refractory tumors and the long-term toxicity of current treatment regimens. Continued vigilance for AYA cancers is needed for high-income countries.

This study has several limitations, which highlight important opportunities to improve estimation of the burden of AYA cancers from future iterations of the GBD study. First, high-quality data in many regions and countries were sparse, particularly in low-income locations. Estimates for these locations may change in future rounds with the addition of new data sources. Estimates from low-income locations were also subject to ascertainment bias, detection bias, and diagnostic inaccuracy. Second, the present topography-based coding system in the GBD is not well-suited for AYA cancers. Uncategorized tumours collectively contributed the largest number of AYA cancer DALYs globally in 2019. This prevented analysis of several important types of AYA cancers, such as Kaposi sarcoma, whose incidence was high among AYAs in LMICs [13]. Classification schemes customized for AYA cancer patients do exist, and we suggest that they be considered, revised if necessary, and incorporated into future GBD iterations [37]. Third, although the GBD 2019 newly incorporated the age-group 15- to 39-year-olds for direct extraction of age-standardized rates, the rates for different years are standardized against each year’s population structure instead of a single standard population (Additional file 1: Appendix pages 6–7). Age-standardized rates for AYA cancers were therefore manually calculated rather than directly extracted. However, this prevented us from deriving uncertainty intervals, which impeded incorporation of quality and availability of source information and statistical uncertainty into our estimates. Fourth, our findings of the association between burden of AYA cancers and SDI should be interpreted with caution. Potential factors associated with AYA cancer burden were not controlled for during analysis due unavailability of covariate data from the GBD. In addition, the identified association should not be construed to suggest causality. Fine-grained analysis performed at local levels would be of value in identifying local problems and informing effective local public health policies.

## Conclusions

In conclusion, our analysis of the GBD 2019 study identified a substantial burden of AYA cancers concentrated among LMICs where resources are limited. Scaling up investment towards AYA cancer prevention will facilitate equitable access to care and promote population health in LMICs and worldwide. The young generation, along with others, will benefit from the capacity building activities for AYA cancers.

## Supplementary Information

**Additional file 1: Fig. S1.** The percentage change in age-standardized incidence (A), death (B) and DALY (C) rates of AYA cancers for 204 countries and territories in both sexes from 1990 to 2019. **Fig. S2.** Global age-specific counts and rates of YLLs and YLDs per 100 000 population due to AYA cancers, 2019. **Fig. S3**. Global trends in absolute and proportional burden of incident cases, deaths, and DALYs by AYA cancer type and sex among 15- to 19-year-olds, 1990–2019. (A) Trends in the number of incident cases of AYA cancers by type. (B) Trends in composition of total incident cases of AYA cancers. (C) Trends in the number of deaths of AYA cancers by type. (D) Trends in composition of total deaths of AYA cancers. (E) Trends in the number of DALYs of AYA cancers by type. (F) Trends in composition of total DALYs of AYA cancers. AYA cancer types were sorted in decreasing magnitude f the total number of incident cases, deaths, or DALYs among 15- to 19-year-olds for both sexes combined from 1990 to 2019. **Fig. S4**. Global trends in absolute and proportional burden of incident cases, deaths, and DALYs by AYA cancer type and sex among 20- to 29-year-olds, 1990–2019. (A) Trends in the number of incident cases of AYA cancers by type. (B) Trends in composition of total incident cases of AYA cancers. (C) Trends in the number of deaths of AYA cancers by type. (D) Trends in composition of total deaths of AYA cancers. (E) Trends in the number of DALYs of AYA cancers by type. (F) Trends in composition of total DALYs of AYA cancers. AYA cancer types were sorted in decreasing magnitude of the total number of incident cases, deaths, or DALYs among 20- to 29-year-olds for both sexes combined from 1990 to 2019. **Fig. S5**. Global trends in absolute and proportional burden of incident cases, deaths, and DALYs by AYA cancer type and sex among 30- to 39-year-olds, 1990–2019. (A) Trends in the number of incident cases of AYA cancers by type. (B) Trends in composition of total incident cases of AYA cancers. (C) Trends in the number of deaths of AYA cancers by type. (D) Trends in composition of total deaths of AYA cancers. (E) Trends in the number of DALYs of AYA cancers by type. (F) Trends in composition of total DALYs of AYA cancers. AYA cancer types were sorted in decreasing magnitude of total number of incident cases, deaths, or DALYs among 30- to 39-year-olds for both sexes combined from 1990 to 2019. **Fig. S6**. The trend in age-standardized incidence rates of AYA cancers across 21 GBD regions by SDI for both sexes combined, 1990–2019. Coloured lines and symbols represent global and regional estimates of incidence rates. Each point on a line represents 1 year, starting from 1990 and ending in 2019. In all regions, the SDI has increased constantly over time. Therefore, points further to the right denote later years for a given region and higher SDI. The black line indicates locally weighted smoothing estimates of incidence rates based on SDI in all regions. The grey shading around the black line represents the 95% confidence interval of the estimated incidence rates. **Fig. S7**. The trend in age-standardized death rates of AYA cancers across 21 GBD regions by SDI for both sexes combined, 1990–2019. Coloured lines and symbols represent global and regional estimates of death rates. Each point on a line represents 1 year, starting from 1990 and ending in 2019. In all regions, the SDI has increased constantly over time. Therefore, points further to the right denote later years for a given region and higher SDI. The black line indicates locally weighted smoothing estimates of death rates based on SDI in all regions. The grey shading around the black line represents the 95% confidence interval of the estimated death rates. **Fig. S8**. The trend in age-standardized DALY rates of AYA cancers across 21 GBD regions by SDI for both sexes combined, 1990–2019. Coloured lines and symbols represent global and regional estimates of DALY rates. Each point on a line represents 1 year, starting from 1990 and ending in 2019. In all regions, the SDI has increased constantly over time. Therefore, points further to the right denote later years for a given region and higher SDI. The black line indicates locally weighted smoothing estimates of DALY rates based on SDI in all regions. The grey shading around the black line represents the 95% confidence interval of the estimated DALY rates. **Table S1**. Incident cases, deaths, and DALYs of AYA cancers among 15 to 19 years in 2019, and percentage change in age-specific rates from 1990 to 2019, by sex, SDI quintile, and cancer types. Absolute incidence, deaths, and DALYs represent AYA cancers among 15- to 19-year-olds (both sexes combined). Rates are reported per 100 000 person-years. Data in parentheses are 95% uncertainty intervals. DALY=disability-adjusted life-year. SDI=Socio-demographic Index. UI=uncertainty interval. **Table S2**. Incident cases, deaths, and DALYs of AYA cancers among 20- to 24-year-olds in 2019, and percentage change in age-specific rates from 1990 to 2019, by sex, SDI quintile, and cancer types. Absolute incidence, deaths, and DALYs represent AYA cancers among 20- to 24-year-olds (both sexes combined). Rates are reported per 100 000 person-years. Data in parentheses are 95% uncertainty intervals. DALY=disability-adjusted life-year. SDI=Socio-demographic Index. UI=uncertainty interval. **Table S3**. Incident cases, deaths, and DALYs of AYA cancers among 25- to 29-year-olds in 2019, and percentage change in age-specific rates from 1990 to 2019, by sex, SDI quintile, and cancer types. Absolute incidence, deaths, and DALYs represent AYA cancers among 25- to 29-year-olds (both sexes combined). Rates are reported per 100 000 person-years. Data in parentheses are 95% uncertainty intervals. DALY=disability-adjusted life-year. SDI=Socio-demographic Index. UI=uncertainty interval. **Table S4**. Incident cases, deaths, and DALYs of AYA cancers among 30- to 34-year-olds in 2019, and percentage change in age-specific rates from 1990 to 2019, by sex, SDI quintile, and cancer types. Absolute incidence, deaths, and DALYs represent AYA cancers among 30- to 34-year-olds (both sexes combined). Rates are reported per 100 000 person-years. Data in parentheses are 95% uncertainty intervals. DALY=disability-adjusted life-year. SDI=Socio-demographic Index. UI=uncertainty interval. **Table S5**. Incident cases, deaths, and DALYs of AYA cancers among 35- to 39-year-olds in 2019, and percentage change in age-specific rates from 1990 to 2019, by sex, SDI quintile, and cancer types. Absolute incidence, deaths, and DALYs represent AYA cancers among 35- to 39-year-olds (both sexes combined). Rates are reported per 100 000 person-years. Data in parentheses are 95% uncertainty intervals. DALY=disability-adjusted life-year. SDI=Socio-demographic Index. UI=uncertainty interval. **Table S6**. Global cause of disease-related death among AYAs in 2019, by GBD SDI quintile. Numbers of deaths are presented outside the parentheses. Numbers inside the parentheses represent percentage of the total deaths due to non-communicable diseases. **Table S7**. Absolute magnitude of changes in age-standardized rates of AYA cancers among GBD regions, from 1990 to 2019, by sex. GBD regions demonstrating temporal trends at odds with global trends are selected for reporting. The majority of GBD regions demonstrated increased AYA cancer incidence rate and decreased death and DALY rates. Therefore, GBD regions with decreased incidence rate and increased death and DALY rates are selected for reporting. Within selected GBD regions, top 10 cancer types leading to increased incidence rate, decreased death rate, and decreased DALY rate from 1990 to 2019 are reported. Negative value indicates lower age-standardized rates in 2019 compared to 1990. **Table S8**. AYA cancer ranking by the number of incident cases at the global level and according to SDI quintile, super-regions, regions, and countries, both sexes, 2019. Colour intensity and number ranking are assigned according to the rank of absolute number of incident cases of each cancer type among all cancer types. Dark red and number ranking of 1 indicate the highest rank and greatest absolute number of incident cases. Dark green and number ranking of 32 indicate the lowest rank and the smallest absolute number of incident cases. **Table S9**. AYA cancer ranking by the number of deaths at the global level and according to SDI quintile, super-regions, regions, and countries, both sexes, 2019. Colour intensity and number ranking are assigned according to the rank of absolute number of deaths of each cancer type among all cancer types. Dark red and number ranking of 1 indicate the highest rank and greatest absolute number of deaths. Dark green and number ranking of 32 indicate the lowest rank and the smallest absolute number of deaths. **Table S10**. AYA cancer ranking by the number of DALYs at the global level and according to SDI quintile, super-regions, regions, and countries, both sexes, 2019. Colour intensity and number ranking are assigned according to the rank of absolute number of DALYs of each cancer type among all cancer types. Dark red and number ranking of 1 indicate the highest rank and greatest absolute DALY burden. Dark green and number ranking of 32 indicate the lowest rank and the smallest absolute DALY burden. **Table S11**. Ranking of the number of incident cases of childhood cancer, AYA cancer, and cancers among the population aged

## Data Availability

The dataset supporting the conclusions of this article is based on the Global Burden of Disease Study 2019 and is freely available on the internet (http://ghdx.healthdata.org/gbd-results-tool).

## Abbreviations

AYA: Adolescents and young adults
DALY: Disability-adjusted life years
GBD: Global Burden of Disease study
LMIC: Low-income and middle-income country
MIR: Mortality-to-incidence ratio
YLD: Years lived with disability
YLL: Years of life lost
