# Supplementary material for: The global, regional, and national burden of cancer among adolescents and young adults in 204 countries and territories, 1990–2019: a population-based study
Source: J Hematol Oncol. 2021 Jun 9;14:89. doi: 10.1186/s13045-021-01093-3 (PMC8191013; doi:10.1186/s13045-021-01093-3)
Supplement: Supplementary file 1 — Additional file 1: Fig. S1. The percentage change in age-standardized incidence (A), death (B) and DALY (C) rates of AYA cancers for 204 countries and territories in both sexes from 1990 to 2019. Fig. S2. Global age-specific counts and rates of YLLs and YLDs per 100 000 population due to AYA cancers, 2019. Fig. S3. Global trends in absolute and proportional burden of incident cases, deaths, and DALYs by AYA cancer type and sex among 15- to 19-year-olds, 1990–2019. (A) Trends in the number of incident cases of AYA cancers by type. (B) Trends in composition of total incident cases of AYA cancers. (C) Trends in the number of deaths of AYA cancers by type. (D) Trends in composition of total deaths of AYA cancers. (E) Trends in the number of DALYs of AYA cancers by type. (F) Trends in composition of total DALYs of AYA cancers. AYA cancer types were sorted in decreasing magnitude f the total number of incident cases, deaths, or DALYs among 15- to 19-year-olds for both sexes combined from 1990 to 2019. Fig. S4. Global trends in absolute and proportional burden of incident cases, deaths, and DALYs by AYA cancer type and sex among 20- to 29-year-olds, 1990–2019. (A) Trends in the number of incident cases of AYA cancers by type. (B) Trends in composition of total incident cases of AYA cancers. (C) Trends in the number of deaths of AYA cancers by type. (D) Trends in composition of total deaths of AYA cancers. (E) Trends in the number of DALYs of AYA cancers by type. (F) Trends in composition of total DALYs of AYA cancers. AYA cancer types were sorted in decreasing magnitude of the total number of incident cases, deaths, or DALYs among 20- to 29-year-olds for both sexes combined from 1990 to 2019. Fig. S5. Global trends in absolute and proportional burden of incident cases, deaths, and DALYs by AYA cancer type and sex among 30- to 39-year-olds, 1990–2019. (A) Trends in the number of incident cases of AYA cancers by type. (B) Trends in composition of total incident cases of AYA [file 13045_2021_1093_MOESM1_ESM.docx]

Table of Contents

GATHER Guidelines Checklist 1

Definition of Indicator 3

Data Sources 4

Cancer incidence data sources 4

Cancer mortality data sources 4

Cancer mortality-to-incidence ratio data sources 4

Bias of categories of input data 4

SDI 4

Data analysis 5

Cancer registry data formatting 5

CODEm models 5

CoDCorrect 5

Incidence estimation 5

Prevalence, YLD, and YLL estimation 5

Uncertainty estimates 5

Derivation of age-standardised rates for cancers among 15 to 39-year-olds based on data from the GBD 2019 6

eFigures 8

eFigure 1. The percentage change in age-standardised incidence (A), death (B) and DALY (C) rates of AYA cancers for 204 countries and territories in both sexes from 1990 to 2019 8

eFigure 2. Global age-specific counts and rates of YLLs and YLDs per 100 000 population due to AYA cancers, 2019 9

eFigure 3. Global trends in absolute and proportional burden of incident cases, deaths, and DALYs by AYA cancer types and sex among 15 to 19-year-olds, 1990-2019 10

eFigure 4. Global trends in absolute and proportional burden of incident cases, deaths, and DALYs by AYA cancer type and sex among 20 to 29-year-olds, 1990-2019 11

eFigure 5. Global trends in absolute and proportional burden of incident cases, deaths, and DALYs by AYA cancer type and sex among 30 to 39-year-olds, 1990-2019 12

eFigure 6. The trend in age-standardised incidence rates of AYA cancers across 21 GBD regions by SDI for both sexes combined, 1990–2019 13

eFigure 7. The trend in age-standardised death rates of AYA cancers across 21 GBD regions by SDI for both sexes combined, 1990–2019 14

eFigure 8. The trend in age-standardised DALY rates of AYA cancers across 21 GBD regions by SDI for both sexes combined, 1990–2019 15

eTables 16

eTable 1. Incident cases, deaths, and DALYs of AYA cancers among 15 to 19-years in 2019, and percentage change in age-specific rates from 1990 to 2019, by sex, SDI quintile, and cancer types 16

eTable 2. Incident cases, deaths, and DALYs of AYA cancers among 20 to 24-year-olds in 2019, and percentage change in age-specific rates from 1990 to 2019, by sex, SDI quintile, and cancer types 17

eTable 3. Incident cases, deaths, and DALYs of AYA cancers among 25 to 29-year-olds in 2019, and percentage change in age-specific rates from 1990 to 2019, by sex, SDI quintile, and cancer types 18

eTable 4. Incident cases, deaths, and DALYs of AYA cancers among 30 to 34-year-olds in 2019, and percentage change in age-specific rates from 1990 to 2019, by sex, SDI quintile, and cancer types 19

eTable 5. Incident cases, deaths, and DALYs of AYA cancers among 35 to 39-year-olds in 2019, and percentage change in age-specific rates from 1990 to 2019, by sex, SDI quintile, and cancer types 20

eTable 6. Global cause of disease-related death among AYAs in 2019, by GBD SDI quintile 21

eTable 7. Absolute magnitude of changes in age-standardised rates of AYA cancers among GBD regions, from 1990 to 2019, by sex 22

eTable 8. AYA cancer ranking by the number of incident cases at the global level and according to SDI quintile, super-regions, regions, and countries, both sexes, 2019 24

eTable 9. AYA cancer ranking by the number of deaths at the global level and according to SDI quintile, super-regions, regions, and countries, both sexes, 2019 30

eTable 11. Ranking of the number of incident cases of childhood cancer, AYA cancer, and cancers among the population aged above 39 years at the global level and according to SDI quintile, both sexes, 2019 42

eTable 12. Ranking of the number of deaths of childhood cancer, AYA cancer, and cancers among the population aged above 39 years at the global level and according to SDI quintile, both sexes, 2019 43

eTable 13. Ranking of the number of DALYs of childhood cancer, AYA cancer, and cancers among population aged above 39 years at the global level and according to SDI quintile, both sexes, 2019 44

# GATHER Guidelines Checklist

| **Objectives and Funding** |  |
| --- | --- |
| 1. Define the indicator(s), populations (including age, sex, and geographic entities), and time period(s) for which estimates were made. | Main text page 7-8 and appendix text page 4: “Definition of indicator” |
| 2. List the funding sources for the work. | Refer to funding section of the manuscript |
| **Data Inputs** |  |
| **For all data inputs from multiple sources that are synthesized as part of the study:** |  |
| 3. Describe how the data were identified and how the data were accessed. | Main text page 8-9: “Estimation of cancer burden” and appendix text page 4: “Data sources” |
| 4. Specify the inclusion and exclusion criteria. Identify all ad-hoc exclusions | Main text page 8-10 and appendix text page 4: “Data sources” |
| 5. Provide information about all included data sources and their main characteristics. For each data source used, report reference information or contact name/institution, population represented, data collection method, year(s) of data collection, sex and age range, diagnostic criteria or measurement method, and sample size, as relevant. | http://ghdx.healthdata.org |
| 6. Identify and describe any categories of input data that have potentially important biases (e.g., based on characteristics listed in item 5). | Appendix text page 4: “Bias of categories of input data” |
| **For data inputs that contribute to the analysis but were not synthesized as part of the study:** |  |
| 7. Describe and give sources for any other data inputs. | http://ghdx.healthdata.org |
| **For all data inputs:** |  |
| 8. Provide all data inputs in a file format from which data can be efficiently extracted (e.g., a spreadsheet rather than a PDF), including all relevant meta-data listed in item 5. For any data inputs that cannot be shared because of ethical or legal reasons, such as third-party ownership, provide a contact name or the name of the institution that retains the right to the data. | http://ghdx.healthdata.org |
| **DATA ANALYSIS** |  |
| 9. Provide a conceptual overview of the data analysis method. A diagram may be helpful. | Briefly introduced in page 5 of the main text. For details, see flowchart of GBD 2019 cancer cause of death estimation on page 195 and flowchart of GBD 2019 cancer incidence, prevalence and YLD estimation on page 803 of Appendix 1 to “Global burden of 369 diseases and injuries in 204 countries and territories, 1990–2019: a systematic analysis for the Global Burden of Disease Study 2019”. |
| 10. Provide a detailed description of all steps of the analysis, including mathematical formulae. This description should cover, as relevant, data cleaning, data pre-processing, data adjustments and weighting of data sources, and mathematical or statistical model(s). | Main text page 9-10 and appendix text page 6-7: “Data Analysis” |
| 11. Describe how candidate models were evaluated and how the final model(s) were selected. | CODEm models. Described in page 6 of the main text and page 6 of the appendix text. |
| 12. Provide the results of an evaluation of model performance, if done, as well as the results of any relevant sensitivity analysis. | See Figure S6 on p 1446 of Supplement 1 to “Global burden of 369 diseases and injuries in 204 countries and territories, 1990–2019: a systematic analysis for the Global Burden of Disease Study 2019” |
| 13. Describe methods of calculating uncertainty of the estimates. State which sources of uncertainty were, and were not, accounted for in the uncertainty analysis. | Main text page 9-10 and appendix text page 6-7: “Data Analysis” |
| 14. State how analytic or statistical source code used to generate estimates can be accessed. | http://ghdx.healthdata.org/gbd-2019/code |
| **RESULTS AND DISCUSSION** |  |
| 15. Provide published estimates in a file format from which data can be efficiently extracted. | GBD 2019 estimates are available Online (http://www.healthdata.org/gbd/data-visualizations and http://ghdx.healthdata.org/gbd-results-tool) |
| 16. Report a quantitative measure of the uncertainty of the estimates (e.g., uncertainty intervals). | See main manuscript “Results” |
| 17. Interpret results in light of existing evidence. If updating a previous set of estimates, describe the reasons for changes in estimates. | See main manuscript “Discussion” |
| 18. Discuss limitations of the estimates. Include a discussion of any modelling assumptions or data limitations that affect interpretation of the estimates. | See main manuscript “Discussion” |

# Definition of Indicator

In this publication, data on 32 cancer groups for adolescents and young adults were presented at global, regional, and national scales, for both sexes, from 1990 to 2019. Data were extracted from the GBD 2019 study in the format of five-year age groups (15-19, 20-24, 25-29, 30-34, and 35-39 year-olds). Total number of cases and truncated age-standardised rates were derived from the data extracted. A complete list of GBD 2019 location hierarchy with levels are available on Appendix Table 1, pages 78-104 of Appendix 1 of “Global age-sex-specific fertility, mortality, healthy life expectancy (HALE), and population estimates in 204 countries and territories, 1950–2019: a comprehensive demographic analysis for the Global Burden of Disease Study 2019”.

These estimates consist of all ICD 9 codes pertaining to cancer and ICD 10 codes except for Kaposi sarcoma (ICD10: C46) and non-melanoma skin cancer (ICD10: C44). The complete lists of ICD codes can be found in “Table S5: List of International Classification of Diseases (ICD) codes mapped to the Global Burden of Disease cause list for causes of death” on page 1478-1483 of Appendix 1 of “Global burden of 369 diseases and injuries in 204 countries and territories, 1990–2019: a systematic analysis for the Global Burden of Disease Study 2019”.

# Data Sources

## Cancer incidence data sources

A detailed description is found on page 23 of Appendix 1 of “Global burden of 369 diseases and injuries in 204 countries and territories, 1990–2019: a systematic analysis for the Global Burden of Disease Study 2019”. Data sources in our study are available in the online GBD citation tool, http://ghdx.healthdata.org and pages 195-196 of Appendix 1 of “Global burden of 369 diseases and injuries in 204 countries and territories, 1990–2019: a systematic analysis for the Global Burden of Disease Study 2019”. Countries and territories that constitute each of the 21 GBD world regions are detailed in page 78 of Appendix Table 1 of “Global age-sex-specific fertility, mortality, healthy life expectancy (HALE), and population estimates in 204 countries and territories, 1950–2019: a comprehensive demographic analysis for the Global Burden of Disease Study 2019”.

## Cancer mortality data sources

The data sources and transformation process for cancer mortality estimates are provided on pages of 197-198 of Appendix 1 of “Global burden of 369 diseases and injuries in 204 countries and territories, 1990–2019: a systematic analysis for the Global Burden of Disease Study 2019”.

## Cancer mortality-to-incidence ratio data sources

Descriptions can be found in “Section 2.2.5: Population-based cancer registries” on page 17 of Supplement 1 of “Global burden of 369 diseases and injuries in 204 countries and territories, 1990–2019: a systematic analysis for the Global Burden of Disease Study 2019”.

## Bias of categories of input data

Please refer to page 196 of Appendix 1 of “Global burden of 369 diseases and injuries in 204 countries and territories, 1990–2019: a systematic analysis for the Global Burden of Disease Study 2019”.

## SDI

Detailed explanation of SDI is available in Section 6 of Appendix 1 of “Global burden of 369 diseases and injuries in 204 countries and territories, 1990–2019: a systematic analysis for the Global Burden of Disease Study 2019”. SDI estimates for all countries from 1990 to 2019 are available on Table S5 from Appendix 1 of “GBD 2019 Risk Factors Collaborators. Global burden of 87 risk factors in 204 countries and territories, 1990–2019: a systematic analysis for the Global Burden of Disease Study 2019”.

# Data analysis

## Cancer registry data formatting

Steps of modelling are described in detail on pages 197-199 of Appendix 1 of “Global burden of 369 diseases and injuries in 204 countries and territories, 1990–2019: a systematic analysis for the Global Burden of Disease Study 2019”.

## CODEm models

This modelling was applied to generate mortality estimates for each cancer. Details can be found on appendix page 48 in Section 3.1 of Appendix 1 of “Global burden of 369 diseases and injuries in 204 countries and territories, 1990–2019: a systematic analysis for the Global Burden of Disease Study 2019”. Covariates in CODEm models are listed on pages 203-212 in Appendix 1 of “Global burden of 369 diseases and injuries in 204 countries and territories, 1990–2019: a systematic analysis for the Global Burden of Disease Study 2019”.

## CoDCorrect

This algorithm is used to adjust the sum of predicted single-cause mortalities in an age-sex-location-year group to be consistent with the results from all-cause mortality estimation. Details regarding the estimation processes are available on appendix pages 55-56 in Section 3.3.2 of Appendix 1 of “Global burden of 369 diseases and injuries in 204 countries and territories, 1990–2019: a systematic analysis for the Global Burden of Disease Study 2019”.

## Incidence estimation

Detailed descriptions are available on pages 200-201 and 806-807 of Appendix 1 of “Global burden of 369 diseases and injuries in 204 countries and territories, 1990–2019: a systematic analysis for the Global Burden of Disease Study 2019”.

## Prevalence, YLD, and YLL estimation

Estimation process of prevalence and YLD can be found on pages 806-811 of Appendix 1 of “Global burden of 369 diseases and injuries in 204 countries and territories, 1990–2019: a systematic analysis for the Global Burden of Disease Study 2019”. More details on sequela and associated disability weights by cancer type are available on pages 1539-1542 of Table S13 of Appendix 1 of “Global burden of 369 diseases and injuries in 204 countries and territories, 1990–2019: a systematic analysis for the Global Burden of Disease Study 2019”. Calculation of YLL is introduced in Section 3.3.3 on page 56 of Appendix 1 of “Global burden of 369 diseases and injuries in 204 countries and territories, 1990–2019: a systematic analysis for the Global Burden of Disease Study 2019”.

## Uncertainty estimates

Uncertainty exists for estimates of all measures of cancer burden due to uncertainties associated with the availability of and variability in cancer data, as well as the uncertainties due to the estimation algorithms. The 95% uncertainty intervals were generated from the 2.5th percentile and 97.5th percentile of the posterior distribution.

# Derivation of age-standardised rates for cancers among 15 to 39-year-olds based on data from the GBD 2019

The GBD 2019 newly added rates for 15 to 39 year-olds in its GBD Results Tool. This section aims to describe why rates directly retrieved from the “15 to 39” age group of the GBD Results Tool are inadequate for purpose of the present study.

The table below tabulates the global absolute number of incident cancer cases (GBD cause hierarchy B.1: neoplasms) for both sexes combined in 2019 in 5-year age groups extracted from the GBD 2019 and corresponding population size. Each step towards the estimation of age-standardised rate is illustrated in the table as well.

| **Age** | **Count** | **Population** | **Age-specific rate** | **Weight** | **Weighted age-specific rate** |
| --- | --- | --- | --- | --- | --- |
| 15 to 19 | 9658255 | 619540978.1 | 1558.937308 | 0.208749779 | 325.4278179 |
| 20 to 24 | 11490465 | 600144464.9 | 1914.616457 | 0.202214266 | 387.1627616 |
| 25 to 29 | 14594779 | 605470102.4 | 2410.487218 | 0.2040087 | 491.7603649 |
| 30 to 34 | 18392880 | 601732453.7 | 3056.654223 | 0.202749327 | 619.7345872 |
| 35 to 39 | 20596839 | 540976121.5 | 3807.347139 | 0.182277928 | 693.9953465 |
|  |  |  |  | **Age-standardised rate = 2518.08087796644** | |

In the table above, the “Count” column contains the number of cancer cases by age group. “Population” contains population size of each age group. Age-specific rate $=\frac{Count}{Population}\times100,000$. The “Weight” column is obtained by dividing population size of each 5-year age group by the sum of population size for all 5-year age groups from 15 to 39 years. The “Age-specific rate” column is multiplied by the “Weight” column to obtain “Weighted age-specific rate”. The sum of weighted age-specific rates gives age-standardised rate.

In the table below, age-standardised rate is derived in a similar way as above. The only difference is that the “Weight” column is derived from GBD 2019 world standard population rather than global population size in 2019.

| **Age** | **Count** | **Population** | **Age-specific rate** | **GBD2019 standard population** | **Weight** | **Weighted age-specific rate** |
| --- | --- | --- | --- | --- | --- | --- |
| 15 to 19 | 9658255 | 619540978.1 | 1558.937308 | 8.324362192 | 0.219256633 | 341.8073452 |
| 20 to 24 | 11490465 | 600144464.9 | 1914.616457 | 7.866450176 | 0.207195619 | 396.7001423 |
| 25 to 29 | 14594779 | 605470102.4 | 2410.487218 | 7.632917343 | 0.201044563 | 484.6153487 |
| 30 to 34 | 18392880 | 601732453.7 | 3056.654223 | 7.331511124 | 0.193105779 | 590.2575941 |
| 35 to 39 | 20596839 | 540976121.5 | 3807.347139 | 6.811055 | 0.179397406 | 683.0282016 |
|  |  |  |  |  | **Age-standardised rate =2496.40863198938** | |

Global age-standardised incidence rate of cancers for both sexes combined in 2019 for 15 to 39-year-olds, as extracted from the “15 to 39” age group of the GBD Results Tool, is 2518.081 per 100 000 person-years. The value is identical to the age-standardised rate calculated from the first Table in this section. This suggests that the rates for 15 to 39-year-olds extracted from the GBD Results Tool are age-standardised against population structure of the corresponding year, rather than against a single standard population. The fact that different populations are used to estimate age-standardised rates for different years for 15 to 39-year-olds means that the resulting rates are confounded by the changing population structure across the investigative period. The purpose of age-standardisation is to remove the effect of differences in age structure. Using different populations for age-standardization is at odds with the mentality of age-standardisation. It therefore needs to be cautioned that exploring trends in burden of AYA cancers using rates extracted from the “15 to 39” age group of the GBD Results Tool, although seemingly straightforward, is inadequate.

# eFigures

## eFigure 1. The percentage change in age-standardised incidence (A), death (B) and DALY (C) rates of AYA cancers for 204 countries and territories in both sexes from 1990 to 2019

## eFigure 2. Global age-specific counts and rates of YLLs and YLDs per 100 000 population due to AYA cancers, 2019

## eFigure 3. Global trends in absolute and proportional burden of incident cases, deaths, and DALYs by AYA cancer types and sex among 15 to 19-year-olds, 1990-2019

(A) Trends in the number of incident cases of AYA cancers by types. (B) Trends in composition of total incident cases of AYA cancers. (C) Trends in the number of deaths of AYA cancers by types. (D) Trends in composition of total deaths of AYA cancers. (E) Trends in the number of DALYs of AYA cancers by types. (F) Trends in composition of total DALYs of AYA cancers. AYA cancer types were sorted in decreasing magnitude of the total number of incident cases, deaths, or DALYs among 15 to 19-year-olds for both sexes combined from 1990 to 2019.

## eFigure 4. Global trends in absolute and proportional burden of incident cases, deaths, and DALYs by AYA cancer type and sex among 20 to 29-year-olds, 1990-2019

(A) Trends in the number of incident cases of AYA cancers by type. (B) Trends in composition of total incident cases of AYA cancers. (C) Trends in the number of deaths of AYA cancers by type. (D) Trends in composition of total deaths of AYA cancers. (E) Trends in the number of DALYs of AYA cancers by types. (F) Trends in composition of total DALYs of AYA cancers. AYA cancer types were sorted in decreasing magnitude of the total number of incident cases, deaths, or DALYs among 20 to 29-year-olds for both sexes combined from 1990 to 2019.

## eFigure 5. Global trends in absolute and proportional burden of incident cases, deaths, and DALYs by AYA cancer type and sex among 30 to 39-year-olds, 1990-2019

(A) Trends in the number of incident cases of AYA cancers by types. (B) Trends in composition of total incident cases of AYA cancers. (C) Trends in the number of deaths of AYA cancers by types. (D) Trends in composition of total deaths of AYA cancers. (E) Trends in the number of DALYs of AYA cancers by types. (F) Trends in composition of total DALYs of AYA cancers. AYA cancer types were sorted in decreasing magnitude of total number of incident cases, deaths, or DALYs among 30 to 39-year-olds for both sexes combined from 1990 to 2019.

## eFigure 6. The trend in age-standardised incidence rates of AYA cancers across 21 GBD regions by SDI for both sexes combined, 1990–2019


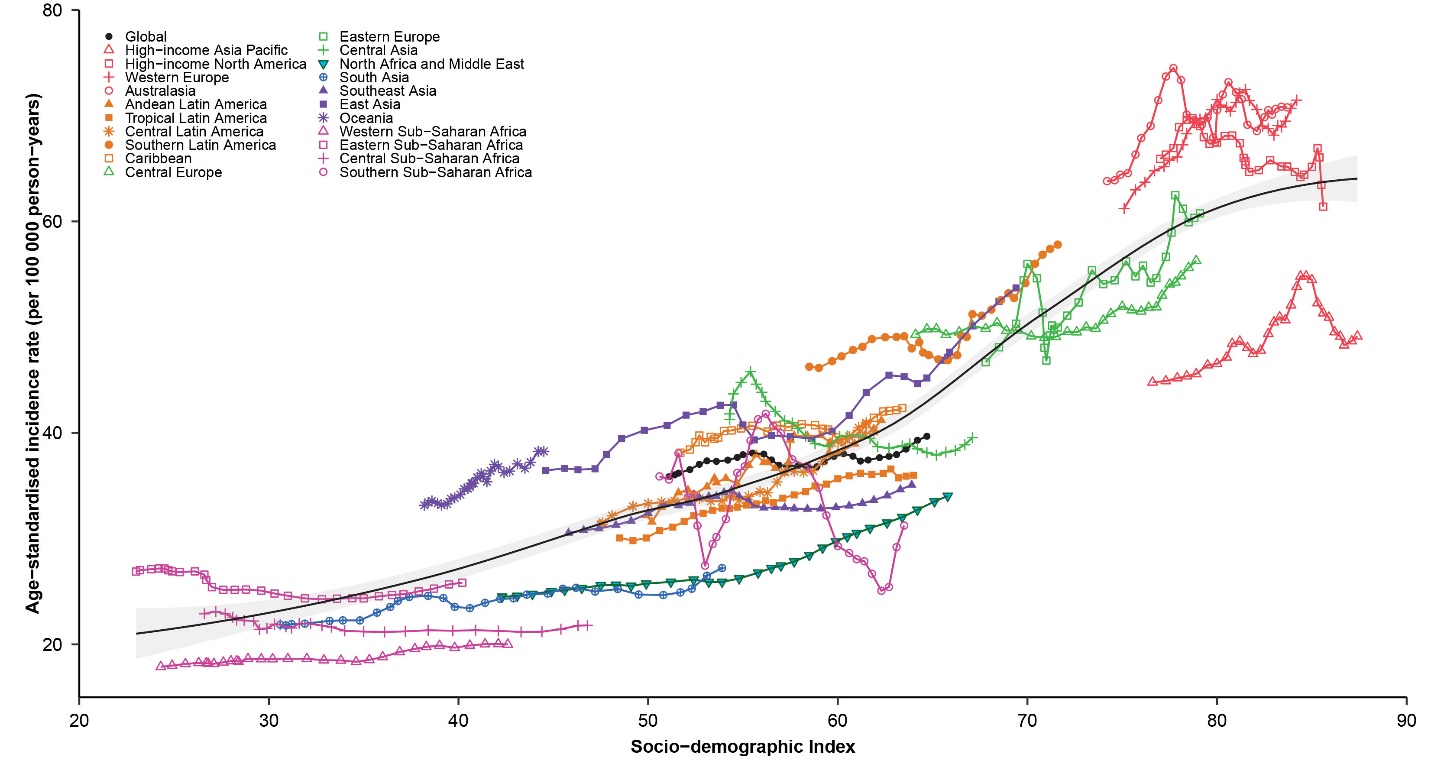
Coloured lines and symbols represent global and regional estimates of incidence rates. Each point on a line represents 1 year, starting from 1990 and ending in 2019. In all regions, the SDI has increased constantly over time. Therefore, points further to the right denote later years for a given region and higher SDI. The black line indicates locally weighted smoothing estimates of incidence rates based on SDI in all regions. The grey shading around the black line represents the 95% confidence interval of the estimated incidence rates.

## eFigure 7. The trend in age-standardised death rates of AYA cancers across 21 GBD regions by SDI for both sexes combined, 1990–2019


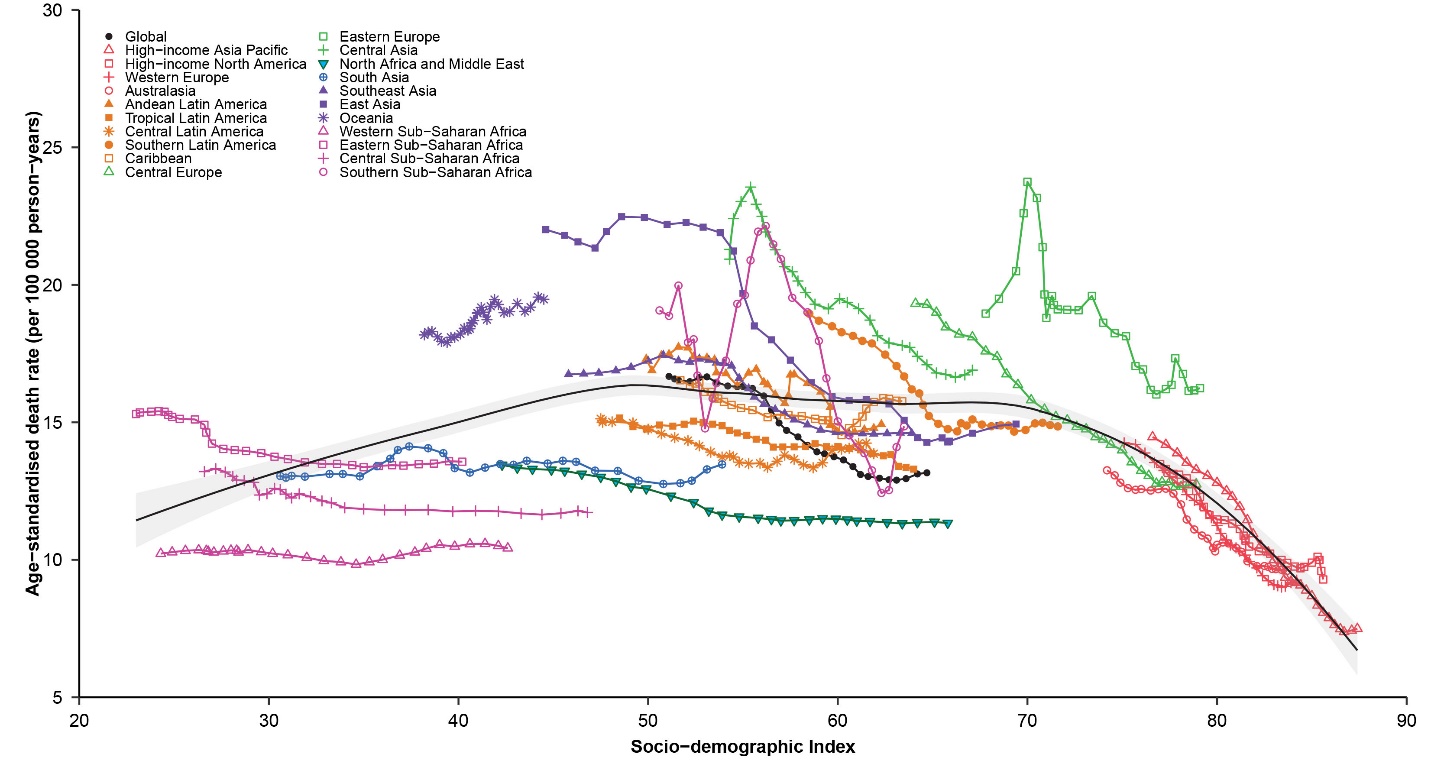
Coloured lines and symbols represent global and regional estimates of death rates. Each point on a line represents 1 year, starting from 1990 and ending in 2019. In all regions, the SDI has increased constantly over time. Therefore, points further to the right denote later years for a given region and higher SDI. The black line indicates locally weighted smoothing estimates of death rates based on SDI in all regions. The grey shading around the black line represents the 95% confidence interval of the estimated death rates.

## eFigure 8. The trend in age-standardised DALY rates of AYA cancers across 21 GBD regions by SDI for both sexes combined, 1990–2019


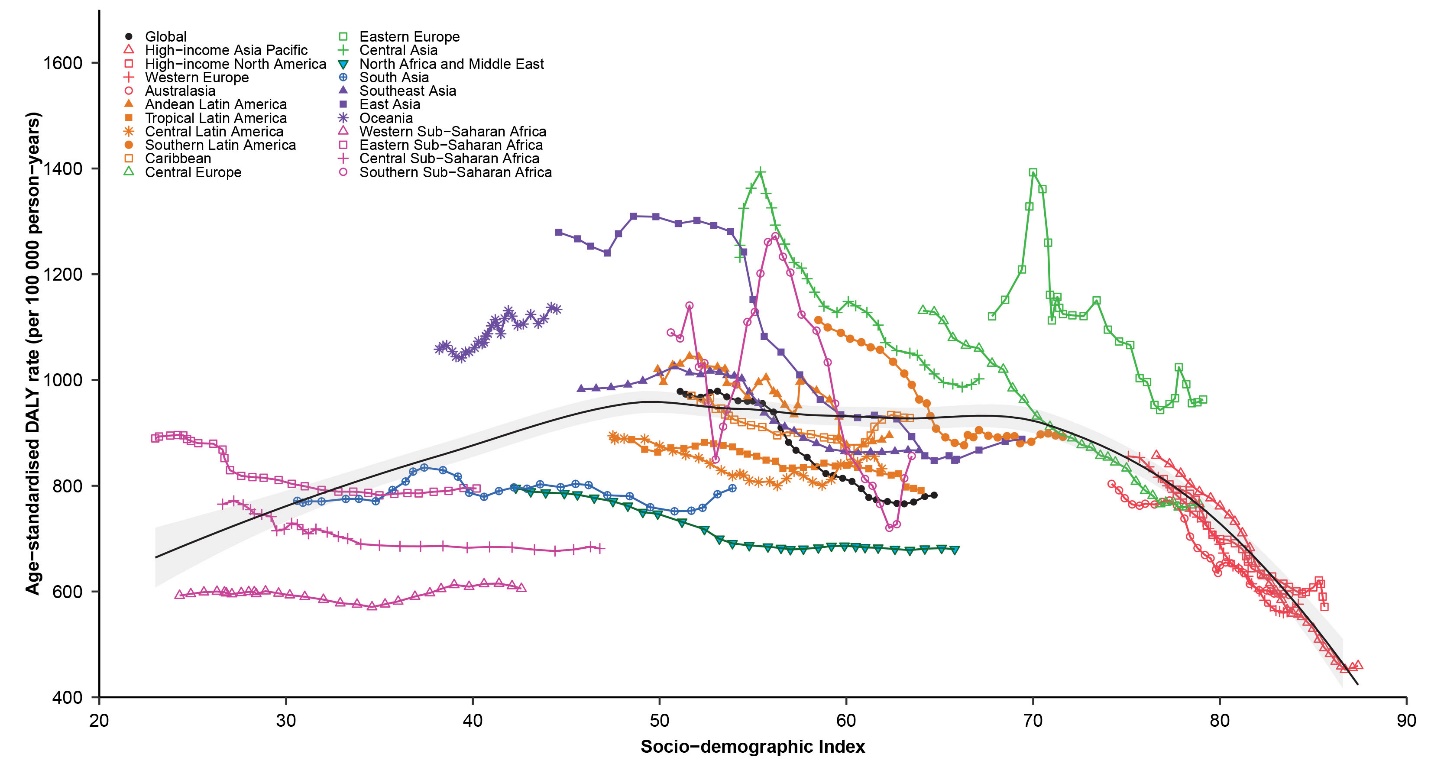
Coloured lines and symbols represent global and regional estimates of DALY rates. Each point on a line represents 1 year, starting from 1990 and ending in 2019. In all regions, the SDI has increased constantly over time. Therefore, points further to the right denote later years for a given region and higher SDI. The black line indicates locally weighted smoothing estimates of DALY rates based on SDI in all regions. The grey shading around the black line represents the 95% confidence interval of the estimated DALY rates.

# eTables

## eTable 1. Incident cases, deaths, and DALYs of AYA cancers among 15 to 19-years in 2019, and percentage change in age-specific rates from 1990 to 2019, by sex, SDI quintile, and cancer types

|  | **Incidence** | | |  | **Deaths** | | |  | **DALYs** | | |
| --- | --- | --- | --- | --- | --- | --- | --- | --- | --- | --- | --- |
|  | **Number of incident cases** | **Age-specific incidence rate (per 100 000 population)** | **Percentage change in rates, 1990–2019** |  | **Number of deaths** | **Age-specific death rate (per 100 000 population)** | **Percentage change in rates, 1990–2019** |  | **Number of DALYs** | **Age-specific DALYs rate (per 100 000 population)** | **Percentage change in rates, 1990–2019** |
| **Global** | 102981 | 16.6 | -1.1 |  | 32746 | 5.3 | -24.2 |  | 2392339 | 386.1 | -23.7 |
| **Female** | 48465 | 16.1 | 3.8 |  | 14125 | 4.7 | -21.9 |  | 1034447 | 342.8 | -21.4 |
| **Male** | 54516 | 17.2 | -5.1 |  | 18621 | 5.9 | -26 |  | 1357892 | 427.3 | -25.6 |
| **High SDI** | 14457 | 25.1 | -0.9 |  | 2044 | 3.5 | -36.3 |  | 153936 | 267.4 | -35.1 |
| **High-middle SDI** | 19233 | 23.2 | 8.4 |  | 4331 | 5.2 | -36.9 |  | 319891 | 386.6 | -35.9 |
| **Middle SDI** | 31876 | 17.4 | 9.1 |  | 10421 | 5.7 | -24.9 |  | 761441 | 416.2 | -24.2 |
| **Low-middle SDI** | 23589 | 13.7 | 5.6 |  | 9745 | 5.7 | -12 |  | 707706 | 411.9 | -11.8 |
| **Low SDI** | 12240 | 9.9 | 5.5 |  | 6183 | 5 | -1.9 |  | 447738 | 360.8 | -1.8 |
| Acute lymphoid leukemia | 8349 (6998 to 9474) | 1.3 (1.1 to 1.5) | -0.8 (-18.8 to 17.1) |  | 3569 (2963 to 4015) | 0.6 (0.5 to 0.6) | -23.9 (-36.8 to -10.2) |  | 259313 (215305 to 291284) | 41.9 (34.8 to 47.0) | -23.5 (-36.5 to -9.8) |
| Acute myeloid leukemia | 4576 (4014 to 5491) | 0.7 (0.6 to 0.9) | -0.3 (-18.7 to 21.0) |  | 2297 (2031 to 2803) | 0.4 (0.3 to 0.5) | -6.8 (-23.6 to 13.3) |  | 165651 (146511 to 201816) | 26.7 (23.6 to 32.6) | -6.7 (-23.6 to 13.3) |
| Bladder cancer | 730 (633 to 910) | 0.1 (0.1 to 0.1) | 13.3 (-3.4 to 39.8) |  | 92 (80 to 112) | 0.0 (0.0 to 0.0) | -29.2 (-40.7 to -12.1) |  | 7119 (6143 to 8688) | 1.1 (1.0 to 1.4) | -27.2 (-38.9 to -9.3) |
| Brain and central nervous system cancer | 7668 (5892 to 8722) | 1.2 (1.0 to 1.4) | 2.0 (-34.8 to 20.9) |  | 3550 (2754 to 4038) | 0.6 (0.4 to 0.7) | -15.3 (-45.6 to 0.1) |  | 256607 (199225 to 291767) | 41.4 (32.2 to 47.1) | -15.1 (-45.5 to 0.4) |
| Breast cancer | 2901 (2505 to 3406) | 0.5 (0.4 to 0.5) | 69.6 (41.6 to 106.8) |  | 715 (593 to 852) | 0.1 (0.1 to 0.1) | 36.9 (10.2 to 74.0) |  | 53071 (44148 to 63553) | 8.6 (7.1 to 10.3) | 37.8 (10.9 to 74.8) |
| Cervical cancer | 1360 (1039 to 1573) | 0.2 (0.2 to 0.3) | -15.9 (-28.6 to 6.7) |  | 236 (177 to 275) | 0.0 (0.0 to 0.0) | -36.0 (-45.8 to -17.8) |  | 17575 (13262 to 20464) | 2.8 (2.1 to 3.3) | -35.3 (-45.1 to -17.1) |
| Chronic lymphoid leukemia | NA | NA | NA |  | NA | NA | NA |  | NA | NA | NA |
| Chronic myeloid leukemia | 893 (762 to 1033) | 0.1 (0.1 to 0.2) | -28.8 (-46.2 to -6.9) |  | 414 (347 to 490) | 0.1 (0.1 to 0.1) | -42.5 (-57.4 to -22.4) |  | 29905 (25075 to 35361) | 4.8 (4.0 to 5.7) | -42.3 (-57.3 to -22.3) |
| Colon and rectum cancer | 2298 (2094 to 2535) | 0.4 (0.3 to 0.4) | -3.6 (-16.1 to 11.1) |  | 786 (713 to 861) | 0.1 (0.1 to 0.1) | -26.8 (-36.9 to -15.9) |  | 57666 (52408 to 63272) | 9.3 (8.5 to 10.2) | -26.4 (-36.4 to -15.4) |
| Esophageal cancer | NA | NA | NA |  | NA | NA | NA |  | NA | NA | NA |
| Gallbladder and biliary tract cancer | NA | NA | NA |  | NA | NA | NA |  | NA | NA | NA |
| Hodgkin lymphoma | 4958 (4346 to 6049) | 0.8 (0.7 to 1.0) | -5.1 (-20.3 to 22.0) |  | 1096 (926 to 1325) | 0.2 (0.1 to 0.2) | -33.6 (-45.4 to -17.4) |  | 80736 (68184 to 97392) | 13.0 (11.0 to 15.7) | -32.9 (-44.7 to -16.5) |
| Kidney cancer | 1330 (1196 to 1475) | 0.2 (0.2 to 0.2) | 29.9 (14.8 to 47.3) |  | 240 (217 to 266) | 0.0 (0.0 to 0.0) | 2.9 (-10.0 to 18.3) |  | 17865 (16098 to 19745) | 2.9 (2.6 to 3.2) | 3.7 (-9.2 to 19.0) |
| Larynx cancer | NA | NA | NA |  | NA | NA | NA |  | NA | NA | NA |
| Lip and oral cavity cancer | 1625 (1397 to 1891) | 0.3 (0.2 to 0.3) | 15.0 (-3.3 to 39.2) |  | 447 (378 to 536) | 0.1 (0.1 to 0.1) | 3.7 (-15.0 to 29.3) |  | 32606 (27672 to 39105) | 5.3 (4.5 to 6.3) | 3.9 (-14.6 to 29.5) |
| Liver cancer | 1196 (1063 to 1351) | 0.2 (0.2 to 0.2) | -50.6 (-59.0 to -41.3) |  | 701 (622 to 793) | 0.1 (0.1 to 0.1) | -54.4 (-62.3 to -44.8) |  | 50391 (44727 to 57040) | 8.1 (7.2 to 9.2) | -54.4 (-62.3 to -44.8) |
| Malignant skin melanoma | 1556 (1248 to 1941) | 0.3 (0.2 to 0.3) | 33.0 (5.8 to 66.3) |  | 169 (141 to 219) | 0.0 (0.0 to 0.0) | -19.2 (-37.7 to 4.0) |  | 12821 (10735 to 16386) | 2.1 (1.7 to 2.6) | -17.1 (-36.2 to 6.6) |
| Mesothelioma | NA | NA | NA |  | NA | NA | NA |  | NA | NA | NA |
| Multiple myeloma | NA | NA | NA |  | NA | NA | NA |  | NA | NA | NA |
| Nasopharynx cancer | 1435 (1277 to 1615) | 0.2 (0.2 to 0.3) | -27.4 (-39.7 to -12.4) |  | 404 (362 to 449) | 0.1 (0.1 to 0.1) | -56.4 (-63.2 to -48.3) |  | 29549 (26493 to 32919) | 4.8 (4.3 to 5.3) | -55.9 (-62.8 to -47.9) |
| Non-Hodgkin lymphoma | 10400 (9058 to 12195) | 1.7 (1.5 to 2.0) | 10.1 (-3.8 to 25.2) |  | 2827 (2564 to 3109) | 0.5 (0.4 to 0.5) | -12.7 (-23.4 to -0.2) |  | 207751 (188459 to 227980) | 33.5 (30.4 to 36.8) | -12.2 (-22.8 to 0.2) |
| Other leukemia | 4264 (3472 to 5024) | 0.7 (0.6 to 0.8) | -63.5 (-70.3 to -49.8) |  | 2541 (2054 to 3008) | 0.4 (0.3 to 0.5) | -64.6 (-71.3 to -51.2) |  | 184220 (148666 to 218370) | 29.7 (24.0 to 35.2) | -64.7 (-71.3 to -51.2) |
| Other malignant neoplasms | 35101 (31054 to 39855) | 5.7 (5.0 to 6.4) | 15.4 (1.4 to 31.9) |  | 9973 (8872 to 11057) | 1.6 (1.4 to 1.8) | 0.7 (-10.3 to 13.4) |  | 731900 (651146 to 811109) | 118.1 (105.1 to 130.9) | 1.1 (-9.7 to 13.7) |
| Other pharynx cancer | NA | NA | NA |  | NA | NA | NA |  | NA | NA | NA |
| Ovarian cancer | 3211 (2672 to 3710) | 0.5 (0.4 to 0.6) | 30.6 (-10.3 to 65.7) |  | 546 (447 to 637) | 0.1 (0.1 to 0.1) | 10.3 (-26.8 to 47.3) |  | 40562 (33222 to 47495) | 6.5 (5.4 to 7.7) | 11.0 (-26.1 to 47.8) |
| Pancreatic cancer | 236 (212 to 264) | 0.0 (0.0 to 0.0) | -6.1 (-18.6 to 8.7) |  | 143 (129 to 159) | 0.0 (0.0 to 0.0) | -8.6 (-20.8 to 6.6) |  | 10291 (9304 to 11468) | 1.7 (1.5 to 1.9) | -8.6 (-20.8 to 6.5) |
| Prostate cancer | NA | NA | NA |  | NA | NA | NA |  | NA | NA | NA |
| Stomach cancer | 1315 (1176 to 1456) | 0.2 (0.2 to 0.2) | -41.2 (-48.0 to -33.8) |  | 661 (593 to 740) | 0.1 (0.1 to 0.1) | -49.8 (-55.7 to -42.3) |  | 47627 (42821 to 53211) | 7.7 (6.9 to 8.6) | -49.8 (-55.6 to -42.3) |
| Testicular cancer | 3774 (3196 to 4522) | 0.6 (0.5 to 0.7) | 40.1 (15.0 to 70.3) |  | 400 (352 to 452) | 0.1 (0.1 to 0.1) | -1.3 (-15.1 to 13.8) |  | 30409 (26726 to 34307) | 4.9 (4.3 to 5.5) | 0.8 (-12.5 to 15.8) |
| Thyroid cancer | 2562 (2211 to 2959) | 0.4 (0.4 to 0.5) | 44.2 (19.2 to 82.8) |  | 201 (176 to 231) | 0.0 (0.0 to 0.0) | -7.9 (-25.1 to 19.7) |  | 15656 (13643 to 17970) | 2.5 (2.2 to 2.9) | -5.0 (-22.9 to 23.2) |
| Tracheal, bronchus, and lung cancer | 1243 (1109 to 1379) | 0.2 (0.2 to 0.2) | -29.7 (-41.7 to -15.8) |  | 738 (663 to 817) | 0.1 (0.1 to 0.1) | -33.3 (-44.1 to -20.6) |  | 53049 (47692 to 58715) | 8.6 (7.7 to 9.5) | -33.3 (-44.1 to -20.7) |
| Uterine cancer | NA | NA | NA |  | NA | NA | NA |  | NA | NA | NA |

Absolute incidence, deaths, and DALYs represent AYA cancers among 15 to 19-year-olds (both sexes combined). Rates are reported per 100 000 person-years. Data in parentheses are 95% uncertainty intervals. NA indicates data unavailable for 15 to 19-year-olds. DALY=disability-adjusted life-year. SDI=Socio-demographic Index. UI=uncertainty interval.

## eTable 2. Incident cases, deaths, and DALYs of AYA cancers among 20 to 24-year-olds in 2019, and percentage change in age-specific rates from 1990 to 2019, by sex, SDI quintile, and cancer types

|  | **Incidence** | | |  | **Deaths** | | |  | **DALYs** | | |
| --- | --- | --- | --- | --- | --- | --- | --- | --- | --- | --- | --- |
|  | **Number of incident cases** | **Age-specific incidence rate (per 100 000 population)** | **Percentage change in rates, 1990–2019** |  | **Number of deaths** | **Age-specific death rate (per 100 000 population)** | **Percentage change in rates, 1990–2019** |  | **Number of DALYs** | **Age-specific DALYs rate (per 100 000 population)** | **Percentage change in rates, 1990–2019** |
| **Global** | 128913 | 21.5 | 15.2 |  | 43948 | 7.3 | -14.4 |  | 2991206 | 498.4 | -13.9 |
| **Female** | 63565 | 21.5 | 16.3 |  | 19989 | 6.8 | -13.9 |  | 1363446 | 461 | -13.2 |
| **Male** | 65348 | 21.5 | 14.2 |  | 23959 | 7.9 | -14.9 |  | 1627760 | 534.8 | -14.5 |
| **High SDI** | 22161 | 35.9 | 16.3 |  | 2920 | 4.7 | -30.2 |  | 206794 | 335.1 | -28.4 |
| **High-middle SDI** | 27301 | 30.3 | 37.5 |  | 6436 | 7.1 | -25.2 |  | 443372 | 491.3 | -23.9 |
| **Middle SDI** | 36938 | 20.3 | 22.3 |  | 13803 | 7.6 | -17.1 |  | 938497 | 514.7 | -16.4 |
| **Low-middle SDI** | 27318 | 16.9 | 18.2 |  | 13315 | 8.2 | -2.1 |  | 898931 | 555.3 | -1.8 |
| **Low SDI** | 13081 | 12.6 | 9 |  | 7447 | 7.2 | 2.9 |  | 501730 | 484.3 | 3 |
| Acute lymphoid leukemia | 7679 (6346 to 8733) | 1.3 (1.1 to 1.5) | 61.8 (28.2 to 91.7) |  | 2514 (2073 to 2793) | 0.4 (0.3 to 0.5) | -4.2 (-23.6 to 12.7) |  | 171987 (141461 to 191649) | 28.7 (23.6 to 31.9) | -2.9 (-22.5 to 14.2) |
| Acute myeloid leukemia | 3828 (3363 to 4394) | 0.6 (0.6 to 0.7) | 12.8 (-5.6 to 32.9) |  | 2124 (1885 to 2481) | 0.4 (0.3 to 0.4) | 6.1 (-10.9 to 25.0) |  | 142616 (126452 to 166153) | 23.8 (21.1 to 27.7) | 6.2 (-10.8 to 25.0) |
| Bladder cancer | 1007 (869 to 1175) | 0.2 (0.1 to 0.2) | 21.4 (2.2 to 44.3) |  | 142 (124 to 162) | 0.0 (0.0 to 0.0) | -24.6 (-36.4 to -10.3) |  | 10110 (8845 to 11582) | 1.7 (1.5 to 1.9) | -22.5 (-34.9 to -8.1) |
| Brain and central nervous system cancer | 8730 (6667 to 9876) | 1.5 (1.1 to 1.6) | 21.1 (-19.5 to 41.5) |  | 3866 (3001 to 4332) | 0.6 (0.5 to 0.7) | -7.5 (-37.9 to 6.6) |  | 261129 (202952 to 291748) | 43.5 (33.8 to 48.6) | -7.1 (-37.7 to 7.2) |
| Breast cancer | 6529 (5747 to 7382) | 1.1 (1.0 to 1.2) | 73.5 (46.0 to 104.9) |  | 1688 (1441 to 1938) | 0.3 (0.2 to 0.3) | 38.3 (14.5 to 68.8) |  | 117014 (100379 to 133825) | 19.5 (16.7 to 22.3) | 39.4 (16.0 to 69.4) |
| Cervical cancer | 5737 (4646 to 6659) | 1.0 (0.8 to 1.1) | -3.3 (-18.0 to 19.4) |  | 1142 (921 to 1349) | 0.2 (0.2 to 0.2) | -24.0 (-36.2 to -2.6) |  | 78892 (63327 to 93415) | 13.1 (10.6 to 15.6) | -23.3 (-35.8 to -2.2) |
| Chronic lymphoid leukemia | 946 (792 to 1102) | 0.2 (0.1 to 0.2) | 99.1 (58.5 to 147.2) |  | 209 (174 to 244) | 0.0 (0.0 to 0.0) | -3.9 (-26.3 to 22.0) |  | 14452 (12091 to 16898) | 2.4 (2.0 to 2.8) | -1.6 (-24.1 to 24.5) |
| Chronic myeloid leukemia | 1389 (1225 to 1576) | 0.2 (0.2 to 0.3) | -14.5 (-32.1 to 7.7) |  | 702 (602 to 826) | 0.1 (0.1 to 0.1) | -30.6 (-45.7 to -10.8) |  | 47192 (40436 to 55429) | 7.9 (6.7 to 9.2) | -30.4 (-45.6 to -10.5) |
| Colon and rectum cancer | 5188 (4738 to 5709) | 0.9 (0.8 to 1.0) | 21.1 (7.1 to 37.4) |  | 1876 (1733 to 2046) | 0.3 (0.3 to 0.3) | -10.9 (-21.2 to 1.5) |  | 128180 (118482 to 139650) | 21.4 (19.7 to 23.3) | -10.2 (-20.6 to 2.0) |
| Esophageal cancer | 561 (484 to 641) | 0.1 (0.1 to 0.1) | -23.9 (-36.6 to -0.7) |  | 360 (312 to 410) | 0.1 (0.1 to 0.1) | -28.9 (-40.9 to -6.2) |  | 24153 (20940 to 27515) | 4.0 (3.5 to 4.6) | -28.9 (-40.8 to -6.1) |
| Gallbladder and biliary tract cancer | 260 (214 to 293) | 0.0 (0.0 to 0.0) | -9.5 (-27.3 to 10.3) |  | 138 (112 to 156) | 0.0 (0.0 to 0.0) | -16.8 (-33.8 to 3.7) |  | 9260 (7503 to 10497) | 1.5 (1.3 to 1.7) | -16.8 (-33.7 to 3.6) |
| Hodgkin lymphoma | 7574 (6714 to 9292) | 1.3 (1.1 to 1.5) | -1.0 (-16.1 to 30.3) |  | 1593 (1344 to 1897) | 0.3 (0.2 to 0.3) | -28.1 (-38.9 to -11.1) |  | 109851 (93223 to 130317) | 18.3 (15.5 to 21.7) | -27.3 (-38.3 to -10.0) |
| Kidney cancer | 1952 (1761 to 2165) | 0.3 (0.3 to 0.4) | 61.9 (43.5 to 82.9) |  | 363 (330 to 401) | 0.1 (0.1 to 0.1) | 22.7 (8.2 to 39.4) |  | 25180 (22838 to 27738) | 4.2 (3.8 to 4.6) | 24.0 (9.6 to 40.9) |
| Larynx cancer | 356 (326 to 390) | 0.1 (0.1 to 0.1) | -8.1 (-18.4 to 5.2) |  | 154 (139 to 170) | 0.0 (0.0 to 0.0) | -26.3 (-35.7 to -14.4) |  | 11162 (10104 to 12323) | 1.9 (1.7 to 2.1) | -25.3 (-34.4 to -14.1) |
| Lip and oral cavity cancer | 2867 (2519 to 3279) | 0.5 (0.4 to 0.5) | 38.0 (19.0 to 62.6) |  | 851 (745 to 987) | 0.1 (0.1 to 0.2) | 24.7 (4.6 to 47.5) |  | 57894 (50608 to 67070) | 9.6 (8.4 to 11.2) | 24.9 (5.0 to 47.8) |
| Liver cancer | 1842 (1654 to 2054) | 0.3 (0.3 to 0.3) | -51.1 (-59.6 to -40.6) |  | 1170 (1048 to 1295) | 0.2 (0.2 to 0.2) | -56.0 (-63.7 to -46.9) |  | 78371 (70170 to 86688) | 13.1 (11.7 to 14.4) | -56.0 (-63.7 to -46.9) |
| Malignant skin melanoma | 3640 (2976 to 4593) | 0.6 (0.5 to 0.8) | 33.3 (12.5 to 63.2) |  | 355 (296 to 450) | 0.1 (0.0 to 0.1) | -13.2 (-30.1 to 9.4) |  | 25422 (21037 to 32158) | 4.2 (3.5 to 5.4) | -10.9 (-28.0 to 12.0) |
| Mesothelioma | 193 (141 to 245) | 0.0 (0.0 to 0.0) | -15.7 (-44.3 to 19.8) |  | 110 (81 to 139) | 0.0 (0.0 to 0.0) | -15.3 (-44.1 to 19.7) |  | 7368 (5443 to 9342) | 1.2 (0.9 to 1.6) | -15.3 (-44.1 to 19.7) |
| Multiple myeloma | 368 (246 to 449) | 0.1 (0.0 to 0.1) | 59.8 (-26.4 to 132.1) |  | 182 (125 to 217) | 0.0 (0.0 to 0.0) | 35.9 (-35.3 to 97.3) |  | 12282 (8460 to 14696) | 2.0 (1.4 to 2.4) | 36.3 (-35.2 to 97.6) |
| Nasopharynx cancer | 2024 (1788 to 2301) | 0.3 (0.3 to 0.4) | -6.1 (-22.8 to 15.4) |  | 592 (537 to 653) | 0.1 (0.1 to 0.1) | -46.8 (-55.3 to -36.2) |  | 40567 (36785 to 44890) | 6.8 (6.1 to 7.5) | -46.0 (-54.7 to -35.3) |
| Non-Hodgkin lymphoma | 8344 (7265 to 9743) | 1.4 (1.2 to 1.6) | 33.3 (17.2 to 51.2) |  | 3673 (3356 to 4034) | 0.6 (0.6 to 0.7) | 6.7 (-5.1 to 20.8) |  | 248466 (227192 to 272686) | 41.4 (37.9 to 45.4) | 7.2 (-4.8 to 21.2) |
| Other leukemia | 4898 (4048 to 5653) | 0.8 (0.7 to 0.9) | -53.1 (-61.3 to -38.3) |  | 3001 (2472 to 3469) | 0.5 (0.4 to 0.6) | -56.0 (-63.7 to -42.6) |  | 203224 (167282 to 235252) | 33.9 (27.9 to 39.2) | -56.0 (-63.6 to -42.5) |
| Other malignant neoplasms | 23680 (21442 to 26009) | 3.9 (3.6 to 4.3) | 31.0 (18.1 to 47.2) |  | 10582 (9448 to 11721) | 1.8 (1.6 to 2.0) | 11.9 (-0.3 to 26.9) |  | 715997 (640457 to 793432) | 119.3 (106.7 to 132.2) | 12.2 (0.0 to 27.3) |
| Other pharynx cancer | 800 (670 to 945) | 0.1 (0.1 to 0.2) | 49.7 (17.3 to 88.5) |  | 431 (352 to 520) | 0.1 (0.1 to 0.1) | 32.0 (-0.7 to 69.4) |  | 28935 (23659 to 34898) | 4.8 (3.9 to 5.8) | 32.1 (-0.5 to 69.4) |
| Ovarian cancer | 5057 (4127 to 5907) | 0.8 (0.7 to 1.0) | 44.3 (-0.3 to 84.4) |  | 948 (771 to 1121) | 0.2 (0.1 to 0.2) | 22.4 (-18.6 to 59.9) |  | 65700 (53252 to 77317) | 10.9 (8.9 to 12.9) | 23.2 (-17.9 to 60.6) |
| Pancreatic cancer | 478 (432 to 531) | 0.1 (0.1 to 0.1) | 7.8 (-8.1 to 26.3) |  | 323 (294 to 361) | 0.1 (0.0 to 0.1) | 5.2 (-9.4 to 23.4) |  | 21614 (19690 to 24167) | 3.6 (3.3 to 4.0) | 5.2 (-9.3 to 23.4) |
| Prostate cancer | 810 (706 to 1007) | 0.1 (0.1 to 0.2) | 69.2 (48.1 to 116.8) |  | 119 (104 to 150) | 0.0 (0.0 to 0.0) | -5.9 (-19.1 to 20.6) |  | 8542 (7455 to 10763) | 1.4 (1.2 to 1.8) | -2.7 (-16.1 to 24.7) |
| Stomach cancer | 3345 (3043 to 3652) | 0.6 (0.5 to 0.6) | -31.1 (-39.5 to -21.6) |  | 1763 (1599 to 1944) | 0.3 (0.3 to 0.3) | -43.8 (-50.5 to -34.8) |  | 118474 (107533 to 130706) | 19.7 (17.9 to 21.8) | -43.7 (-50.3 to -34.7) |
| Testicular cancer | 10470 (8912 to 12367) | 1.7 (1.5 to 2.1) | 52.8 (24.2 to 93.5) |  | 1024 (924 to 1145) | 0.2 (0.2 to 0.2) | 5.8 (-7.0 to 21.5) |  | 73348 (65893 to 81457) | 12.2 (11.0 to 13.6) | 8.3 (-4.1 to 23.4) |
| Thyroid cancer | 5058 (4209 to 5914) | 0.8 (0.7 to 1.0) | 70.3 (37.6 to 122.2) |  | 430 (359 to 515) | 0.1 (0.1 to 0.1) | 6.1 (-17.4 to 42.7) |  | 31192 (26075 to 37035) | 5.2 (4.3 to 6.2) | 9.5 (-14.0 to 46.5) |
| Tracheal, bronchus, and lung cancer | 2178 (1971 to 2412) | 0.4 (0.3 to 0.4) | -17.2 (-30.3 to -3.0) |  | 1423 (1293 to 1569) | 0.2 (0.2 to 0.3) | -21.9 (-33.5 to -9.5) |  | 95317 (86638 to 105147) | 15.9 (14.4 to 17.5) | -21.9 (-33.5 to -9.5) |
| Uterine cancer | 1122 (773 to 1318) | 0.2 (0.1 to 0.2) | -0.1 (-18.2 to 27.4) |  | 101 (68 to 115) | 0.0 (0.0 to 0.0) | -45.6 (-55.4 to -25.7) |  | 7315 (4962 to 8415) | 1.2 (0.8 to 1.4) | -43.3 (-53.4 to -22.7) |

Absolute incidence, deaths, and DALYs represent AYA cancers among 20 to 24-year-olds (both sexes combined). Rates are reported per 100 000 person-years. Data in parentheses are 95% uncertainty intervals. DALY=disability-adjusted life-year. SDI=Socio-demographic Index. UI=uncertainty interval.

## eTable 3. Incident cases, deaths, and DALYs of AYA cancers among 25 to 29-year-olds in 2019, and percentage change in age-specific rates from 1990 to 2019, by sex, SDI quintile, and cancer types

|  | **Incidence** | | |  | **Deaths** | | |  | **DALYs** | | |
| --- | --- | --- | --- | --- | --- | --- | --- | --- | --- | --- | --- |
|  | **Number of incident cases** | **Age-specific incidence rate (per 100 000 population)** | **Percentage change in rates, 1990–2019** |  | **Number of deaths** | **Age-specific death rate (per 100 000 population)** | **Percentage change in rates, 1990–2019** |  | **Number of DALYs** | **Age-specific DALYs rate (per 100 000 population)** | **Percentage change in rates, 1990–2019** |
| **Global** | 195541 | 32.3 | 17.3 |  | 60751 | 10 | -15.4 |  | 3844099 | 634.9 | -14.6 |
| **Female** | 107441 | 35.7 | 14.6 |  | 30654 | 10.2 | -17.2 |  | 1945971 | 647.2 | -16.4 |
| **Male** | 88101 | 28.9 | 20.9 |  | 30097 | 9.9 | -13.4 |  | 1898128 | 622.8 | -12.7 |
| **High SDI** | 35946 | 53 | 12.2 |  | 4614 | 6.8 | -31.4 |  | 304517 | 448.6 | -29.4 |
| **High-middle SDI** | 47029 | 43.2 | 41.7 |  | 10733 | 9.9 | -23.1 |  | 686708 | 630.3 | -21.6 |
| **Middle SDI** | 56985 | 29.2 | 25.8 |  | 20035 | 10.3 | -18.8 |  | 1265157 | 647.8 | -17.9 |
| **Low-middle SDI** | 35467 | 24 | 20.4 |  | 16444 | 11.1 | -3.2 |  | 1029951 | 697.7 | -2.8 |
| **Low SDI** | 16933 | 19.8 | 5 |  | 8884 | 10.4 | -1.6 |  | 555270 | 650.3 | -1.5 |
| Acute lymphoid leukemia | 6734 (5491 to 7770) | 1.1 (0.9 to 1.3) | 80.7 (37.0 to 121.1) |  | 2081 (1661 to 2325) | 0.3 (0.3 to 0.4) | -2.0 (-25.9 to 17.1) |  | 132451 (105932 to 148032) | 21.9 (17.5 to 24.4) | -0.2 (-24.1 to 19.3) |
| Acute myeloid leukemia | 3883 (3466 to 4405) | 0.6 (0.6 to 0.7) | 6.4 (-9.3 to 23.9) |  | 2343 (2100 to 2627) | 0.4 (0.3 to 0.4) | 0.9 (-14.3 to 17.3) |  | 145563 (130535 to 163629) | 24.0 (21.6 to 27.0) | 0.9 (-14.3 to 17.3) |
| Bladder cancer | 1651 (1445 to 1898) | 0.3 (0.2 to 0.3) | 21.5 (3.9 to 43.5) |  | 234 (207 to 266) | 0.0 (0.0 to 0.0) | -27.5 (-38.3 to -14.1) |  | 15530 (13738 to 17671) | 2.6 (2.3 to 2.9) | -25.2 (-36.2 to -11.3) |
| Brain and central nervous system cancer | 12226 (9622 to 13785) | 2.0 (1.6 to 2.3) | 34.1 (-4.3 to 55.1) |  | 5280 (4117 to 5874) | 0.9 (0.7 to 1.0) | -2.0 (-29.8 to 11.6) |  | 330897 (257685 to 368650) | 54.7 (42.6 to 60.9) | -1.3 (-29.5 to 12.4) |
| Breast cancer | 19428 (17302 to 21655) | 3.2 (2.9 to 3.6) | 46.7 (28.1 to 69.2) |  | 4539 (4012 to 5092) | 0.7 (0.7 to 0.8) | 12.7 (-3.8 to 33.3) |  | 293789 (259747 to 327937) | 48.5 (42.9 to 54.2) | 13.9 (-2.2 to 34.1) |
| Cervical cancer | 16746 (13773 to 19142) | 2.8 (2.3 to 3.2) | -4.7 (-19.1 to 12.9) |  | 3451 (2856 to 4002) | 0.6 (0.5 to 0.7) | -24.5 (-36.8 to -6.4) |  | 221015 (182341 to 255946) | 36.5 (30.1 to 42.3) | -23.8 (-36.1 to -5.8) |
| Chronic lymphoid leukemia | 953 (802 to 1120) | 0.2 (0.1 to 0.2) | 110.3 (66.3 to 167.2) |  | 214 (182 to 249) | 0.0 (0.0 to 0.0) | 0.9 (-21.7 to 30.4) |  | 13783 (11756 to 15969) | 2.3 (1.9 to 2.6) | 3.4 (-19.8 to 33.3) |
| Chronic myeloid leukemia | 2043 (1813 to 2348) | 0.3 (0.3 to 0.4) | -10.8 (-27.2 to 10.2) |  | 1080 (938 to 1272) | 0.2 (0.2 to 0.2) | -28.0 (-42.8 to -8.9) |  | 67225 (58374 to 79107) | 11.1 (9.6 to 13.1) | -27.9 (-42.6 to -8.8) |
| Colon and rectum cancer | 10549 (9708 to 11514) | 1.7 (1.6 to 1.9) | 38.6 (24.1 to 54.5) |  | 3772 (3498 to 4067) | 0.6 (0.6 to 0.7) | -3.2 (-13.0 to 7.4) |  | 238899 (221599 to 257585) | 39.5 (36.6 to 42.5) | -2.3 (-12.3 to 8.2) |
| Esophageal cancer | 969 (845 to 1102) | 0.2 (0.1 to 0.2) | -18.5 (-31.2 to 1.1) |  | 675 (595 to 769) | 0.1 (0.1 to 0.1) | -24.0 (-36.5 to -4.3) |  | 41937 (37057 to 47769) | 6.9 (6.1 to 7.9) | -24.0 (-36.4 to -4.3) |
| Gallbladder and biliary tract cancer | 476 (397 to 539) | 0.1 (0.1 to 0.1) | -11.9 (-31.5 to 10.7) |  | 273 (224 to 310) | 0.0 (0.0 to 0.1) | -18.3 (-36.5 to 4.1) |  | 16976 (13893 to 19280) | 2.8 (2.3 to 3.2) | -18.2 (-36.5 to 4.2) |
| Hodgkin lymphoma | 7810 (6885 to 9617) | 1.3 (1.1 to 1.6) | -9.3 (-22.1 to 18.4) |  | 1924 (1618 to 2289) | 0.3 (0.3 to 0.4) | -31.6 (-41.3 to -17.4) |  | 122427 (103324 to 144985) | 20.2 (17.1 to 23.9) | -31.0 (-40.7 to -16.8) |
| Kidney cancer | 3107 (2813 to 3437) | 0.5 (0.5 to 0.6) | 70.7 (51.5 to 92.0) |  | 592 (543 to 653) | 0.1 (0.1 to 0.1) | 27.5 (12.5 to 43.5) |  | 38021 (34717 to 41779) | 6.3 (5.7 to 6.9) | 29.0 (13.9 to 45.2) |
| Larynx cancer | 495 (452 to 539) | 0.1 (0.1 to 0.1) | -9.5 (-19.3 to 3.2) |  | 229 (208 to 253) | 0.0 (0.0 to 0.0) | -28.9 (-36.7 to -17.8) |  | 15049 (13710 to 16564) | 2.5 (2.3 to 2.7) | -27.9 (-35.6 to -17.4) |
| Lip and oral cavity cancer | 4462 (3878 to 5083) | 0.7 (0.6 to 0.8) | 36.7 (17.2 to 57.5) |  | 1411 (1219 to 1639) | 0.2 (0.2 to 0.3) | 24.0 (4.4 to 46.9) |  | 88820 (76971 to 103115) | 14.7 (12.7 to 17.0) | 24.2 (4.8 to 46.8) |
| Liver cancer | 3457 (3107 to 3864) | 0.6 (0.5 to 0.6) | -43.4 (-53.3 to -31.9) |  | 2329 (2102 to 2566) | 0.4 (0.3 to 0.4) | -50.3 (-58.7 to -39.7) |  | 144407 (130428 to 159134) | 23.9 (21.5 to 26.3) | -50.2 (-58.6 to -39.6) |
| Malignant skin melanoma | 7481 (6020 to 9501) | 1.2 (1.0 to 1.6) | 19.7 (3.2 to 46.7) |  | 674 (564 to 837) | 0.1 (0.1 to 0.1) | -20.8 (-33.0 to -2.1) |  | 45256 (37603 to 56169) | 7.5 (6.2 to 9.3) | -18.4 (-30.7 to 0.1) |
| Mesothelioma | 307 (226 to 409) | 0.1 (0.0 to 0.1) | -23.2 (-62.2 to 19.6) |  | 191 (141 to 253) | 0.0 (0.0 to 0.0) | -21.9 (-60.1 to 22.3) |  | 11868 (8770 to 15718) | 2.0 (1.4 to 2.6) | -21.9 (-60.1 to 22.3) |
| Multiple myeloma | 430 (291 to 523) | 0.1 (0.0 to 0.1) | 62.9 (-18.1 to 125.9) |  | 223 (151 to 266) | 0.0 (0.0 to 0.0) | 39.1 (-30.6 to 91.7) |  | 13969 (9453 to 16588) | 2.3 (1.6 to 2.7) | 39.5 (-30.3 to 91.9) |
| Nasopharynx cancer | 3702 (3212 to 4248) | 0.6 (0.5 to 0.7) | 46.3 (20.7 to 79.7) |  | 845 (762 to 931) | 0.1 (0.1 to 0.2) | -39.4 (-48.5 to -27.2) |  | 54095 (48953 to 59729) | 8.9 (8.1 to 9.9) | -37.9 (-47.0 to -25.6) |
| Non-Hodgkin lymphoma | 9229 (8218 to 10406) | 1.5 (1.4 to 1.7) | 26.5 (13.6 to 39.8) |  | 3973 (3681 to 4328) | 0.7 (0.6 to 0.7) | 1.9 (-8.7 to 13.9) |  | 249614 (231073 to 272334) | 41.2 (38.2 to 45.0) | 2.5 (-8.1 to 14.4) |
| Other leukemia | 5523 (4494 to 6333) | 0.9 (0.7 to 1.0) | -42.9 (-52.5 to -25.1) |  | 3000 (2441 to 3390) | 0.5 (0.4 to 0.6) | -50.7 (-59.1 to -36.1) |  | 188274 (153678 to 212767) | 31.1 (25.4 to 35.1) | -50.6 (-59.0 to -35.9) |
| Other malignant neoplasms | 26539 (24269 to 29036) | 4.4 (4.0 to 4.8) | 36.2 (23.3 to 52.0) |  | 9863 (8932 to 10881) | 1.6 (1.5 to 1.8) | 6.2 (-5.5 to 19.5) |  | 620475 (560888 to 685002) | 102.5 (92.6 to 113.1) | 6.8 (-4.9 to 20.0) |
| Other pharynx cancer | 1014 (879 to 1168) | 0.2 (0.1 to 0.2) | 35.7 (7.9 to 66.4) |  | 589 (496 to 682) | 0.1 (0.1 to 0.1) | 17.6 (-9.3 to 46.8) |  | 36551 (30860 to 42293) | 6.0 (5.1 to 7.0) | 17.7 (-9.2 to 46.8) |
| Ovarian cancer | 6892 (5661 to 8077) | 1.1 (0.9 to 1.3) | 40.4 (-0.3 to 75.6) |  | 1388 (1119 to 1610) | 0.2 (0.2 to 0.3) | 20.5 (-18.7 to 54.1) |  | 89061 (71721 to 102505) | 14.7 (11.8 to 16.9) | 21.2 (-18.1 to 54.8) |
| Pancreatic cancer | 1011 (922 to 1111) | 0.2 (0.2 to 0.2) | 19.1 (5.1 to 35.8) |  | 740 (678 to 811) | 0.1 (0.1 to 0.1) | 16.5 (2.4 to 33.2) |  | 45855 (41983 to 50265) | 7.6 (6.9 to 8.3) | 16.6 (2.5 to 33.2) |
| Prostate cancer | 1208 (1063 to 1518) | 0.2 (0.2 to 0.3) | 77.4 (58.6 to 121.2) |  | 198 (174 to 253) | 0.0 (0.0 to 0.0) | 6.2 (-7.1 to 32.0) |  | 13114 (11529 to 16605) | 2.2 (1.9 to 2.7) | 9.3 (-4.1 to 35.6) |
| Stomach cancer | 6739 (6135 to 7336) | 1.1 (1.0 to 1.2) | -27.3 (-35.4 to -18.5) |  | 3581 (3271 to 3900) | 0.6 (0.5 to 0.6) | -43.8 (-49.8 to -36.5) |  | 223068 (204190 to 242918) | 36.8 (33.7 to 40.1) | -43.6 (-49.6 to -36.3) |
| Testicular cancer | 15200 (12910 to 18174) | 2.5 (2.1 to 3.0) | 38.9 (12.4 to 80.8) |  | 1406 (1282 to 1553) | 0.2 (0.2 to 0.3) | -3.4 (-13.8 to 9.5) |  | 94182 (85918 to 104120) | 15.6 (14.2 to 17.2) | -0.8 (-11.3 to 12.5) |
| Thyroid cancer | 8567 (7206 to 9993) | 1.4 (1.2 to 1.7) | 69.1 (39.7 to 116.5) |  | 611 (519 to 706) | 0.1 (0.1 to 0.1) | 2.7 (-19.6 to 36.5) |  | 41952 (35294 to 48902) | 6.9 (5.8 to 8.1) | 7.0 (-15.1 to 42.1) |
| Tracheal, bronchus, and lung cancer | 4011 (3610 to 4425) | 0.7 (0.6 to 0.7) | -15.6 (-27.8 to -3.5) |  | 2804 (2538 to 3082) | 0.5 (0.4 to 0.5) | -21.3 (-32.4 to -9.2) |  | 173903 (157325 to 191122) | 28.7 (26.0 to 31.6) | -21.2 (-32.4 to -9.2) |
| Uterine cancer | 2701 (2058 to 3125) | 0.4 (0.3 to 0.5) | 11.3 (-7.6 to 38.8) |  | 237 (175 to 272) | 0.0 (0.0 to 0.0) | -39.7 (-51.0 to -19.9) |  | 16073 (11879 to 18449) | 2.7 (2.0 to 3.0) | -37.0 (-48.3 to -16.3) |

Absolute incidence, deaths, and DALYs represent AYA cancers among 25 to 29-year-olds (both sexes combined). Rates are reported per 100 000 person-years. Data in parentheses are 95% uncertainty intervals. DALY=disability-adjusted life-year. SDI=Socio-demographic Index. UI=uncertainty interval.

## eTable 4. Incident cases, deaths, and DALYs of AYA cancers among 30 to 34-year-olds in 2019, and percentage change in age-specific rates from 1990 to 2019, by sex, SDI quintile, and cancer types

|  | **Incidence** | | |  | **Deaths** | | |  | **DALYs** | | |
| --- | --- | --- | --- | --- | --- | --- | --- | --- | --- | --- | --- |
|  | **Number of incident cases** | **Age-specific incidence rate (per 100 000 population)** | **Percentage change in rates, 1990–2019** |  | **Number of deaths** | **Age-specific death rate (per 100 000 population)** | **Percentage change in rates, 1990–2019** |  | **Number of DALYs** | **Age-specific DALYs rate (per 100 000 population)** | **Percentage change in rates, 1990–2019** |
| **Global** | 318155 | 52.9 | 15.3 |  | 101122 | 16.8 | -19 |  | 5891695 | 979.1 | -18.2 |
| **Female** | 188263 | 63.1 | 10.4 |  | 52673 | 17.6 | -21.6 |  | 3085516 | 1033.5 | -20.7 |
| **Male** | 129892 | 42.8 | 22.8 |  | 48449 | 16 | -16.2 |  | 2806179 | 925.6 | -15.4 |
| **High SDI** | 56339 | 78.6 | 7.5 |  | 8560 | 11.9 | -31.7 |  | 517032 | 721.5 | -30 |
| **High-middle SDI** | 87435 | 70.7 | 39.7 |  | 21465 | 17.4 | -24.2 |  | 1262510 | 1020.9 | -22.7 |
| **Middle SDI** | 95983 | 48.2 | 21.4 |  | 34684 | 17.4 | -23.7 |  | 2015500 | 1012.5 | -22.8 |
| **Low-middle SDI** | 50012 | 37.2 | 17.7 |  | 24063 | 17.9 | -4.8 |  | 1386279 | 1030.1 | -4.4 |
| **Low SDI** | 23159 | 32 | 3.1 |  | 12286 | 17 | -4.4 |  | 706658 | 975.6 | -4.2 |
| Acute lymphoid leukemia | 7552 (5832 to 8850) | 1.3 (1.0 to 1.5) | 158.1 (93.9 to 221.9) |  | 1797 (1362 to 2017) | 0.3 (0.2 to 0.3) | 2.6 (-23.6 to 22.3) |  | 106632 (81136 to 119507) | 17.7 (13.5 to 19.9) | 6.1 (-21.2 to 25.9) |
| Acute myeloid leukemia | 3760 (3377 to 4202) | 0.6 (0.6 to 0.7) | 4.3 (-10.7 to 21.0) |  | 2512 (2266 to 2803) | 0.4 (0.4 to 0.5) | -1.0 (-14.9 to 14.2) |  | 143337 (129281 to 159993) | 23.8 (21.5 to 26.6) | -0.9 (-14.9 to 14.3) |
| Bladder cancer | 3895 (3433 to 4389) | 0.6 (0.6 to 0.7) | 38.2 (18.8 to 60.1) |  | 560 (500 to 627) | 0.1 (0.1 to 0.1) | -18.9 (-30.8 to -5.3) |  | 34337 (30610 to 38479) | 5.7 (5.1 to 6.4) | -16.0 (-28.1 to -2.6) |
| Brain and central nervous system cancer | 15807 (12575 to 17735) | 2.6 (2.1 to 2.9) | 34.1 (0.8 to 53.2) |  | 7337 (5794 to 8151) | 1.2 (1.0 to 1.4) | -3.7 (-27.1 to 8.8) |  | 422390 (332721 to 468223) | 70.2 (55.3 to 77.8) | -3.1 (-26.8 to 9.4) |
| Breast cancer | 49873 (44957 to 54974) | 8.3 (7.5 to 9.1) | 25.2 (11.4 to 40.3) |  | 12131 (10959 to 13312) | 2.0 (1.8 to 2.2) | -7.2 (-18.0 to 5.3) |  | 722533 (651290 to 790689) | 120.1 (108.2 to 131.4) | -6.0 (-16.8 to 6.6) |
| Cervical cancer | 39557 (33195 to 44639) | 6.6 (5.5 to 7.4) | -6.0 (-19.9 to 9.9) |  | 8198 (6815 to 9366) | 1.4 (1.1 to 1.6) | -26.6 (-38.0 to -11.8) |  | 484445 (405232 to 554907) | 80.5 (67.3 to 92.2) | -25.9 (-37.2 to -11.2) |
| Chronic lymphoid leukemia | 1122 (932 to 1328) | 0.2 (0.2 to 0.2) | 155.5 (102.1 to 220.7) |  | 263 (219 to 301) | 0.0 (0.0 to 0.1) | 15.0 (-9.3 to 45.5) |  | 15568 (12987 to 17800) | 2.6 (2.2 to 3.0) | 18.2 (-6.9 to 48.8) |
| Chronic myeloid leukemia | 2265 (2044 to 2556) | 0.4 (0.3 to 0.4) | -15.8 (-29.2 to -0.1) |  | 1257 (1104 to 1448) | 0.2 (0.2 to 0.2) | -34.2 (-45.4 to -20.2) |  | 71964 (63239 to 82881) | 12.0 (10.5 to 13.8) | -34.0 (-45.2 to -20.0) |
| Colon and rectum cancer | 22464 (20514 to 24594) | 3.7 (3.4 to 4.1) | 47.5 (33.0 to 62.6) |  | 8583 (7937 to 9299) | 1.4 (1.3 to 1.5) | 1.8 (-7.6 to 11.9) |  | 499064 (461641 to 539583) | 82.9 (76.7 to 89.7) | 2.7 (-6.6 to 12.8) |
| Esophageal cancer | 2105 (1868 to 2343) | 0.3 (0.3 to 0.4) | -17.1 (-28.7 to 1.1) |  | 1607 (1436 to 1790) | 0.3 (0.2 to 0.3) | -23.8 (-35.3 to -5.6) |  | 91675 (81995 to 101933) | 15.2 (13.6 to 16.9) | -23.7 (-35.2 to -5.5) |
| Gallbladder and biliary tract cancer | 1069 (899 to 1193) | 0.2 (0.1 to 0.2) | -9.0 (-27.8 to 9.4) |  | 667 (559 to 744) | 0.1 (0.1 to 0.1) | -15.4 (-33.7 to 3.5) |  | 38075 (31911 to 42487) | 6.3 (5.3 to 7.1) | -15.4 (-33.6 to 3.5) |
| Hodgkin lymphoma | 7170 (6321 to 8652) | 1.2 (1.1 to 1.4) | -13.9 (-25.2 to 13.3) |  | 1809 (1497 to 2140) | 0.3 (0.2 to 0.4) | -38.0 (-46.0 to -23.6) |  | 105927 (88083 to 124952) | 17.6 (14.6 to 20.8) | -37.3 (-45.4 to -22.7) |
| Kidney cancer | 5572 (5051 to 6155) | 0.9 (0.8 to 1.0) | 76.8 (57.8 to 98.3) |  | 1029 (939 to 1142) | 0.2 (0.2 to 0.2) | 26.4 (12.3 to 43.1) |  | 60975 (55784 to 67505) | 10.1 (9.3 to 11.2) | 28.3 (13.9 to 44.8) |
| Larynx cancer | 1003 (921 to 1097) | 0.2 (0.2 to 0.2) | -10.1 (-18.7 to 1.3) |  | 537 (493 to 592) | 0.1 (0.1 to 0.1) | -25.6 (-33.4 to -15.1) |  | 31519 (28975 to 34623) | 5.2 (4.8 to 5.8) | -25.1 (-32.7 to -14.9) |
| Lip and oral cavity cancer | 7710 (6844 to 8638) | 1.3 (1.1 to 1.4) | 31.5 (13.7 to 50.6) |  | 2669 (2340 to 3026) | 0.4 (0.4 to 0.5) | 19.5 (1.1 to 38.6) |  | 154429 (135481 to 175177) | 25.7 (22.5 to 29.1) | 19.8 (1.5 to 38.7) |
| Liver cancer | 7268 (6431 to 8217) | 1.2 (1.1 to 1.4) | -42.4 (-53.0 to -30.0) |  | 5382 (4782 to 6048) | 0.9 (0.8 to 1.0) | -50.0 (-58.8 to -38.4) |  | 306460 (272445 to 343978) | 50.9 (45.3 to 57.2) | -49.9 (-58.7 to -38.4) |
| Malignant skin melanoma | 11090 (8983 to 13976) | 1.8 (1.5 to 2.3) | 10.2 (-4.9 to 37.8) |  | 1215 (997 to 1482) | 0.2 (0.2 to 0.2) | -26.8 (-36.4 to -11.0) |  | 74272 (60912 to 91249) | 12.3 (10.1 to 15.2) | -24.9 (-34.6 to -8.6) |
| Mesothelioma | 346 (281 to 412) | 0.1 (0.0 to 0.1) | -11.1 (-30.9 to 12.7) |  | 243 (200 to 286) | 0.0 (0.0 to 0.0) | -8.8 (-28.7 to 15.7) |  | 13859 (11382 to 16308) | 2.3 (1.9 to 2.7) | -8.8 (-28.8 to 15.7) |
| Multiple myeloma | 768 (585 to 876) | 0.1 (0.1 to 0.1) | 39.3 (-12.1 to 71.2) |  | 450 (346 to 507) | 0.1 (0.1 to 0.1) | 24.0 (-22.9 to 52.3) |  | 25882 (19945 to 29106) | 4.3 (3.3 to 4.8) | 24.4 (-22.6 to 52.8) |
| Nasopharynx cancer | 9442 (8087 to 11046) | 1.6 (1.3 to 1.8) | 122.5 (81.3 to 175.1) |  | 1591 (1447 to 1751) | 0.3 (0.2 to 0.3) | -37.8 (-46.7 to -26.5) |  | 95572 (86948 to 105619) | 15.9 (14.4 to 17.6) | -34.8 (-44.3 to -23.7) |
| Non-Hodgkin lymphoma | 11199 (10073 to 12506) | 1.9 (1.7 to 2.1) | 22.3 (10.8 to 34.4) |  | 4674 (4350 to 5068) | 0.8 (0.7 to 0.8) | -0.6 (-9.4 to 8.7) |  | 270677 (252001 to 293256) | 45.0 (41.9 to 48.7) | 0.0 (-8.8 to 9.3) |
| Other leukemia | 5873 (4798 to 6800) | 1.0 (0.8 to 1.1) | -35.2 (-46.1 to -16.3) |  | 3361 (2731 to 3869) | 0.6 (0.5 to 0.6) | -45.4 (-54.8 to -29.5) |  | 193796 (157843 to 222297) | 32.2 (26.2 to 36.9) | -45.2 (-54.6 to -29.2) |
| Other malignant neoplasms | 27321 (24961 to 29892) | 4.5 (4.1 to 5.0) | 36.3 (23.3 to 51.7) |  | 9810 (8873 to 10702) | 1.6 (1.5 to 1.8) | -0.6 (-10.3 to 11.4) |  | 568549 (513695 to 619819) | 94.5 (85.4 to 103.0) | 0.2 (-9.5 to 12.2) |
| Other pharynx cancer | 1735 (1517 to 1947) | 0.3 (0.3 to 0.3) | 36.9 (12.0 to 61.9) |  | 1095 (936 to 1252) | 0.2 (0.2 to 0.2) | 19.6 (-4.8 to 45.7) |  | 62477 (53395 to 71319) | 10.4 (8.9 to 11.9) | 19.7 (-4.7 to 45.8) |
| Ovarian cancer | 8448 (7145 to 9777) | 1.4 (1.2 to 1.6) | 25.9 (-6.2 to 55.1) |  | 2275 (1911 to 2591) | 0.4 (0.3 to 0.4) | 10.1 (-21.9 to 37.9) |  | 133444 (111828 to 152502) | 22.2 (18.6 to 25.3) | 10.6 (-21.5 to 38.5) |
| Pancreatic cancer | 2639 (2399 to 2904) | 0.4 (0.4 to 0.5) | 24.7 (10.5 to 42.1) |  | 2144 (1964 to 2367) | 0.4 (0.3 to 0.4) | 22.6 (8.1 to 39.6) |  | 121969 (111780 to 134645) | 20.3 (18.6 to 22.4) | 22.6 (8.1 to 39.6) |
| Prostate cancer | 1509 (1304 to 1811) | 0.3 (0.2 to 0.3) | 74.0 (57.1 to 104.1) |  | 242 (207 to 294) | 0.0 (0.0 to 0.0) | -2.2 (-12.6 to 14.4) |  | 14818 (12651 to 17834) | 2.5 (2.1 to 3.0) | 1.1 (-9.6 to 17.9) |
| Stomach cancer | 15313 (13992 to 16714) | 2.5 (2.3 to 2.8) | -23.5 (-31.8 to -14.7) |  | 8296 (7639 to 9004) | 1.4 (1.3 to 1.5) | -44.0 (-49.8 to -37.4) |  | 475179 (437770 to 515055) | 79.0 (72.8 to 85.6) | -43.7 (-49.5 to -37.2) |
| Testicular cancer | 15825 (13386 to 18745) | 2.6 (2.2 to 3.1) | 36.6 (11.4 to 74.5) |  | 1389 (1265 to 1536) | 0.2 (0.2 to 0.3) | -9.3 (-19.7 to 4.0) |  | 86527 (78821 to 96227) | 14.4 (13.1 to 16.0) | -6.2 (-16.8 to 7.1) |
| Thyroid cancer | 13786 (11891 to 15641) | 2.3 (2.0 to 2.6) | 68.0 (42.1 to 103.1) |  | 685 (605 to 768) | 0.1 (0.1 to 0.1) | -2.4 (-19.0 to 22.7) |  | 45776 (39493 to 51876) | 7.6 (6.6 to 8.6) | 4.3 (-12.9 to 30.8) |
| Tracheal, bronchus, and lung cancer | 8880 (8039 to 9733) | 1.5 (1.3 to 1.6) | -16.7 (-27.4 to -6.2) |  | 6805 (6188 to 7442) | 1.1 (1.0 to 1.2) | -22.4 (-32.5 to -12.2) |  | 387708 (352799 to 424276) | 64.4 (58.6 to 70.5) | -22.3 (-32.4 to -12.2) |
| Uterine cancer | 5785 (4557 to 6670) | 1.0 (0.8 to 1.1) | 20.1 (1.5 to 43.5) |  | 508 (379 to 581) | 0.1 (0.1 to 0.1) | -34.9 (-46.4 to -17.3) |  | 31859 (23851 to 36366) | 5.3 (4.0 to 6.0) | -31.8 (-43.0 to -13.3) |

Absolute incidence, deaths, and DALYs represent AYA cancers among 30 to 34-year-olds (both sexes combined). Rates are reported per 100 000 person-years. Data in parentheses are 95% uncertainty intervals. DALY=disability-adjusted life-year. SDI=Socio-demographic Index. UI=uncertainty interval.

## eTable 5. Incident cases, deaths, and DALYs of AYA cancers among 35 to 39-year-olds in 2019, and percentage change in age-specific rates from 1990 to 2019, by sex, SDI quintile, and cancer types

|  | **Incidence** | | |  | **Deaths** | | |  | **DALYs** | | |
| --- | --- | --- | --- | --- | --- | --- | --- | --- | --- | --- | --- |
|  | **Number of incident cases** | **Age-specific incidence rate (per 100 000 population)** | **Percentage change in rates, 1990–2019** |  | **Number of deaths** | **Age-specific death rate (per 100 000 population)** | **Percentage change in rates, 1990–2019** |  | **Number of DALYs** | **Age-specific DALYs rate (per 100 000 population)** | **Percentage change in rates, 1990–2019** |
| **Global** | 448791 | 83 | 7 |  | 157550 | 29.1 | -25.1 |  | 8371945 | 1547.6 | -24.3 |
| **Female** | 277905 | 103.5 | 6.8 |  | 85012 | 31.7 | -23.2 |  | 4544440 | 1692.9 | -22.4 |
| **Male** | 170886 | 62.7 | 6.7 |  | 72538 | 26.6 | -27.3 |  | 3827506 | 1404.4 | -26.6 |
| **High SDI** | 84263 | 116.2 | -1.3 |  | 15234 | 21 | -35.9 |  | 835550 | 1152.7 | -34.5 |
| **High-middle SDI** | 120331 | 108.3 | 23.9 |  | 33646 | 30.3 | -31.3 |  | 1802892 | 1622.6 | -30 |
| **Middle SDI** | 133854 | 76.3 | 10.3 |  | 52876 | 30.1 | -30.6 |  | 2802547 | 1597.5 | -29.7 |
| **Low-middle SDI** | 71270 | 59.7 | 16.8 |  | 37016 | 31 | -5.2 |  | 1945531 | 1629.2 | -4.8 |
| **Low SDI** | 32445 | 52.2 | 2.4 |  | 18677 | 30 | -6 |  | 980040 | 1575.3 | -5.7 |
| Acute lymphoid leukemia | 8433 (6865 to 9846) | 1.6 (1.3 to 1.8) | 167.2 (105.2 to 229.6) |  | 1735 (1367 to 1925) | 0.3 (0.3 to 0.4) | -6.1 (-27.2 to 10.3) |  | 95128 (74794 to 105561) | 17.6 (13.8 to 19.5) | -1.9 (-24.1 to 15.0) |
| Acute myeloid leukemia | 4136 (3694 to 4677) | 0.8 (0.7 to 0.9) | 7.1 (-8.2 to 24.2) |  | 2889 (2569 to 3296) | 0.5 (0.5 to 0.6) | 1.5 (-12.2 to 17.1) |  | 150393 (133888 to 171650) | 27.8 (24.7 to 31.7) | 1.6 (-12.2 to 17.1) |
| Bladder cancer | 6811 (6072 to 7729) | 1.3 (1.1 to 1.4) | 29.4 (12.2 to 50.9) |  | 1022 (913 to 1153) | 0.2 (0.2 to 0.2) | -22.3 (-33.1 to -9.0) |  | 57380 (51556 to 64657) | 10.6 (9.5 to 12.0) | -19.5 (-30.5 to -6.1) |
| Brain and central nervous system cancer | 17080 (13415 to 19275) | 3.2 (2.5 to 3.6) | 23.1 (-7.4 to 40.5) |  | 9073 (7089 to 10161) | 1.7 (1.3 to 1.9) | -8.0 (-29.9 to 4.3) |  | 475883 (372257 to 532417) | 88.0 (68.8 to 98.4) | -7.4 (-29.6 to 4.9) |
| Breast cancer | 91128 (82592 to 100114) | 16.8 (15.3 to 18.5) | 18.9 (6.2 to 31.3) |  | 24014 (21868 to 26463) | 4.4 (4.0 to 4.9) | -9.0 (-18.6 to 1.0) |  | 1303012 (1185051 to 1427214) | 240.9 (219.1 to 263.8) | -8.0 (-17.3 to 1.9) |
| Cervical cancer | 55858 (47346 to 63465) | 10.3 (8.8 to 11.7) | -2.4 (-17.4 to 13.8) |  | 14141 (12141 to 16315) | 2.6 (2.2 to 3.0) | -22.6 (-34.7 to -5.8) |  | 758614 (651759 to 874775) | 140.2 (120.5 to 161.7) | -21.9 (-34.1 to -5.0) |
| Chronic lymphoid leukemia | 1241 (1072 to 1434) | 0.2 (0.2 to 0.3) | 98.4 (59.6 to 150.3) |  | 324 (281 to 367) | 0.1 (0.1 to 0.1) | -2.7 (-22.2 to 24.2) |  | 17573 (15214 to 19974) | 3.2 (2.8 to 3.7) | 0.2 (-19.9 to 27.6) |
| Chronic myeloid leukemia | 2613 (2342 to 2944) | 0.5 (0.4 to 0.5) | -15.4 (-27.7 to -2.0) |  | 1503 (1312 to 1726) | 0.3 (0.2 to 0.3) | -33.6 (-44.4 to -20.5) |  | 78574 (68644 to 90066) | 14.5 (12.7 to 16.6) | -33.3 (-44.1 to -20.2) |
| Colon and rectum cancer | 35591 (32580 to 38903) | 6.6 (6.0 to 7.2) | 41.1 (27.8 to 56.0) |  | 13335 (12276 to 14499) | 2.5 (2.3 to 2.7) | -1.8 (-11.6 to 7.8) |  | 709601 (654183 to 770475) | 131.2 (120.9 to 142.4) | -0.8 (-10.8 to 8.9) |
| Esophageal cancer | 4453 (4003 to 4969) | 0.8 (0.7 to 0.9) | -27.6 (-38.0 to -6.0) |  | 3573 (3202 to 4004) | 0.7 (0.6 to 0.7) | -33.1 (-42.5 to -12.1) |  | 185987 (166895 to 208644) | 34.4 (30.9 to 38.6) | -33.0 (-42.4 to -12.0) |
| Gallbladder and biliary tract cancer | 2036 (1749 to 2264) | 0.4 (0.3 to 0.4) | -11.1 (-28.7 to 4.4) |  | 1311 (1123 to 1461) | 0.2 (0.2 to 0.3) | -17.6 (-34.8 to -1.9) |  | 68250 (58355 to 75989) | 12.6 (10.8 to 14.0) | -17.6 (-34.7 to -1.9) |
| Hodgkin lymphoma | 5876 (5126 to 7143) | 1.1 (0.9 to 1.3) | -13.4 (-25.2 to 15.1) |  | 1671 (1373 to 1982) | 0.3 (0.3 to 0.4) | -39.1 (-47.3 to -23.8) |  | 89275 (73589 to 106106) | 16.5 (13.6 to 19.6) | -38.4 (-46.5 to -22.7) |
| Kidney cancer | 9175 (8324 to 10100) | 1.7 (1.5 to 1.9) | 59.6 (42.9 to 79.7) |  | 1793 (1644 to 1980) | 0.3 (0.3 to 0.4) | 18.1 (4.5 to 34.1) |  | 97113 (88983 to 107049) | 18.0 (16.4 to 19.8) | 19.7 (6.3 to 35.8) |
| Larynx cancer | 2360 (2153 to 2595) | 0.4 (0.4 to 0.5) | -19.5 (-27.4 to -10.4) |  | 1330 (1199 to 1483) | 0.2 (0.2 to 0.3) | -31.0 (-38.7 to -21.5) |  | 70460 (63434 to 78293) | 13.0 (11.7 to 14.5) | -30.7 (-38.4 to -21.1) |
| Lip and oral cavity cancer | 12776 (11308 to 14426) | 2.4 (2.1 to 2.7) | 27.3 (9.4 to 45.4) |  | 4666 (4114 to 5280) | 0.9 (0.8 to 1.0) | 18.6 (0.7 to 38.8) |  | 246585 (217160 to 278865) | 45.6 (40.1 to 51.5) | 18.8 (0.9 to 38.6) |
| Liver cancer | 11667 (10200 to 13312) | 2.2 (1.9 to 2.5) | -56.5 (-64.9 to -46.4) |  | 8991 (7843 to 10207) | 1.7 (1.4 to 1.9) | -62.5 (-69.6 to -53.6) |  | 467247 (407752 to 529820) | 86.4 (75.4 to 97.9) | -62.5 (-69.5 to -53.5) |
| Malignant skin melanoma | 13498 (10852 to 17178) | 2.5 (2.0 to 3.2) | 13.7 (-1.4 to 43.2) |  | 1835 (1516 to 2250) | 0.3 (0.3 to 0.4) | -25.5 (-34.2 to -9.4) |  | 101318 (83348 to 124029) | 18.7 (15.4 to 22.9) | -23.6 (-32.8 to -7.0) |
| Mesothelioma | 620 (486 to 832) | 0.1 (0.1 to 0.2) | -18.9 (-58.1 to 22.2) |  | 447 (349 to 600) | 0.1 (0.1 to 0.1) | -17.2 (-58.7 to 25.8) |  | 23270 (18187 to 31231) | 4.3 (3.4 to 5.8) | -17.3 (-58.7 to 25.8) |
| Multiple myeloma | 1366 (1120 to 1557) | 0.3 (0.2 to 0.3) | 22.8 (-13.2 to 43.7) |  | 826 (667 to 927) | 0.2 (0.1 to 0.2) | 9.3 (-24.9 to 28.2) |  | 43430 (35094 to 48754) | 8.0 (6.5 to 9.0) | 9.7 (-24.5 to 28.5) |
| Nasopharynx cancer | 11958 (10365 to 13922) | 2.2 (1.9 to 2.6) | 70.3 (41.2 to 107.1) |  | 2647 (2405 to 2920) | 0.5 (0.4 to 0.5) | -40.5 (-49.1 to -30.4) |  | 143128 (129946 to 157760) | 26.5 (24.0 to 29.2) | -38.6 (-47.2 to -28.2) |
| Non-Hodgkin lymphoma | 13255 (11904 to 14757) | 2.5 (2.2 to 2.7) | 17.2 (6.9 to 27.9) |  | 5650 (5234 to 6133) | 1.0 (1.0 to 1.1) | -2.8 (-11.1 to 5.3) |  | 299436 (277596 to 325663) | 55.4 (51.3 to 60.2) | -2.2 (-10.4 to 5.8) |
| Other leukemia | 8253 (6949 to 9598) | 1.5 (1.3 to 1.8) | -37.6 (-48.4 to -17.6) |  | 3374 (2835 to 3963) | 0.6 (0.5 to 0.7) | -53.7 (-61.7 to -38.9) |  | 179290 (150471 to 210877) | 33.1 (27.8 to 39.0) | -53.3 (-61.4 to -38.6) |
| Other malignant neoplasms | 28468 (26057 to 31081) | 5.3 (4.8 to 5.7) | 29.3 (16.5 to 44.2) |  | 11226 (10145 to 12273) | 2.1 (1.9 to 2.3) | -4.8 (-14.0 to 6.1) |  | 593276 (535236 to 649325) | 109.7 (98.9 to 120.0) | -4.1 (-13.2 to 6.9) |
| Other pharynx cancer | 3553 (3141 to 3995) | 0.7 (0.6 to 0.7) | 33.7 (14.6 to 54.1) |  | 2245 (1951 to 2557) | 0.4 (0.4 to 0.5) | 21.7 (-0.7 to 45.7) |  | 117030 (101701 to 133292) | 21.6 (18.8 to 24.6) | 21.9 (-0.6 to 45.8) |
| Ovarian cancer | 12224 (10514 to 13968) | 2.3 (1.9 to 2.6) | 24.6 (-3.2 to 48.0) |  | 3738 (3143 to 4325) | 0.7 (0.6 to 0.8) | 10.0 (-18.0 to 33.2) |  | 200263 (168493 to 231376) | 37.0 (31.1 to 42.8) | 10.6 (-17.5 to 33.7) |
| Pancreatic cancer | 5036 (4587 to 5560) | 0.9 (0.8 to 1.0) | 17.6 (4.8 to 33.0) |  | 4258 (3893 to 4705) | 0.8 (0.7 to 0.9) | 15.3 (1.9 to 31.2) |  | 221068 (202132 to 244296) | 40.9 (37.4 to 45.2) | 15.4 (1.9 to 31.2) |
| Prostate cancer | 1944 (1650 to 2315) | 0.4 (0.3 to 0.4) | 85.6 (67.6 to 110.8) |  | 316 (266 to 376) | 0.1 (0.0 to 0.1) | -1.4 (-11.7 to 12.5) |  | 17856 (15085 to 21261) | 3.3 (2.8 to 3.9) | 2.5 (-7.9 to 16.4) |
| Stomach cancer | 22296 (20443 to 24399) | 4.1 (3.8 to 4.5) | -35.3 (-41.6 to -27.8) |  | 13595 (12541 to 14780) | 2.5 (2.3 to 2.7) | -49.2 (-53.9 to -43.5) |  | 709548 (653955 to 770645) | 131.2 (120.9 to 142.5) | -49.0 (-53.8 to -43.3) |
| Testicular cancer | 12132 (10275 to 14377) | 2.2 (1.9 to 2.7) | 44.9 (17.1 to 80.0) |  | 1134 (1030 to 1252) | 0.2 (0.2 to 0.2) | -6.7 (-17.5 to 6.6) |  | 64617 (58642 to 71158) | 11.9 (10.8 to 13.2) | -3.1 (-14.0 to 10.3) |
| Thyroid cancer | 16858 (14803 to 18741) | 3.1 (2.7 to 3.5) | 65.2 (43.2 to 89.5) |  | 926 (822 to 1022) | 0.2 (0.2 to 0.2) | -2.6 (-17.2 to 15.2) |  | 56346 (49455 to 63028) | 10.4 (9.1 to 11.7) | 3.9 (-11.7 to 22.7) |
| Tracheal, bronchus, and lung cancer | 16288 (14809 to 17858) | 3.0 (2.7 to 3.3) | -29.6 (-38.9 to -20.4) |  | 13001 (11894 to 14245) | 2.4 (2.2 to 2.6) | -34.3 (-42.8 to -26.0) |  | 676024 (618438 to 739441) | 125.0 (114.3 to 136.7) | -34.3 (-42.7 to -25.9) |
| Uterine cancer | 9808 (8125 to 11106) | 1.8 (1.5 to 2.1) | 15.2 (-0.8 to 34.2) |  | 962 (752 to 1077) | 0.2 (0.1 to 0.2) | -37.8 (-47.4 to -23.1) |  | 54968 (43087 to 61844) | 10.2 (8.0 to 11.4) | -34.8 (-44.6 to -19.7) |

Absolute incidence, deaths, and DALYs represent AYA cancers among 35 to 39-year-olds (both sexes combined). Rates are reported per 100 000 person-years. Data in parentheses are 95% uncertainty intervals. DALY=disability-adjusted life-year. SDI=Socio-demographic Index. UI=uncertainty interval.

## eTable 6. Global cause of disease-related death among AYAs in 2019, by GBD SDI quintile

|  | | **Global** | **Low SDI** | **Low-middle SDI** | **Middle SDI** | **High-middle SDI** | **High SDI** |
| --- | --- | --- | --- | --- | --- | --- | --- |
| **Communicable, maternal, neonatal, and nutritional diseases** | | 1172865 | 442765 | 401191 | 258757 | 60002 | 9349 |
| **Non-communicable diseases** | | 1530030 | 248068 | 451305 | 458605 | 241993 | 129005 |
|  | Cardiovascular diseases | 455854 (29.8%) | 69596 (28.1%) | 140587 (31.2%) | 146880 (32.0%) | 75429 (31.2%) | 23012 (17.8%) |
|  | **Neoplasms** | **398598 (26.1%)** | **53683 (21.6%)** | **101108 (22.4%)** | **132729 (28.9%)** | **77188 (31.9%)** | **33633 (26.1%)** |
|  | Digestive diseases | 244747 (16.0%) | 51223 (20.6%) | 90666 (20.1%) | 62024 (13.5%) | 32726 (13.5%) | 7990 (6.2%) |
|  | Diabetes and kidney diseases | 127052 (8.3%) | 24530 (9.9%) | 39999 (8.9%) | 45392 (9.9%) | 11701 (4.8%) | 5308 (4.1%) |
|  | Substance use disorders | 95088 (6.2%) | 3660 (1.5%) | 13239 (2.9%) | 14654 (3.2%) | 20824 (8.6%) | 42683 (33.1%) |
|  | Other non-communicable diseases | 82762 (5.4%) | 18920 (7.6%) | 24696 (5.5%) | 22037 (4.8%) | 9563 (4.0%) | 7482 (5.8%) |
|  | Chronic respiratory diseases | 57986 (3.8%) | 14317 (5.8%) | 20253 (4.5%) | 15802 (3.4%) | 4780 (2.0%) | 2768 (2.1%) |
|  | Neurological disorders | 51699 (3.4%) | 10374 (4.2%) | 16720 (3.7%) | 12991 (2.8%) | 7030 (2.9%) | 4552 (3.5%) |
|  | Musculoskeletal disorders | 11100 (0.7%) | 981 (0.4%) | 2424 (0.5%) | 4504 (1.0%) | 1965 (0.8%) | 1216 (0.9%) |
|  | Skin and subcutaneous diseases | 4944 (0.3%) | 777 (0.3%) | 1596 (0.4%) | 1561 (0.3%) | 755 (0.3%) | 248 (0.2%) |
|  | Mental disorders | 199 (0.0%) | 6 (0.0%) | 17 (0.0%) | 31 (0.0%) | 33 (0.0%) | 113 (0.1%) |

Numbers of deaths are presented outside the parentheses. Numbers inside the parentheses represent percentage of the total deaths due to non-communicable diseases.

## eTable 7. Absolute magnitude of changes in age-standardised rates of AYA cancers among GBD regions, from 1990 to 2019, by sex

| **Leading cancer types by region and sex resulting in decreased AYA cancer incidence rate** | | | | | | | | | | | | | | | |
| --- | --- | --- | --- | --- | --- | --- | --- | --- | --- | --- | --- | --- | --- | --- | --- |
|  |  | **Eastern Sub−Saharan Africa** | |  | **Central Sub−Saharan Africa** | |  | **Central Asia** | |  | **High-income North America** | |  | **Southern Sub−Saharan Africa** | |
|  |  | **Cancer type** | **Absolute change in rate, 1990-2019** |  | **Cancer type** | **Absolute change in rate, 1990-2019** |  | **Cancer type** | **Absolute change in rate, 1990-2019** |  | **Cancer type** | **Absolute change in rate, 1990-2019** |  | **Cancer type** | **Absolute change in rate, 1990-2019** |
|  | **Females** | |  |  |  |  |  |  |  |  |  |  |  |  |  |
|  |  | Cervical cancer | $-1.60\times{10}^{-2}$ |  | Cervical cancer | $-1.01\times{10}^{-2}$ |  | Stomach cancer | $-4.82\times{10}^{-3}$ |  | Breast cancer | $-1.59\times{10}^{-2}$ |  | Cervical cancer | $-1.23\times{10}^{-2}$ |
|  |  | Stomach cancer | $-3.27\times{10}^{-3}$ |  | Stomach cancer | $-2.25\times{10}^{-3}$ |  | Cervical cancer | $-4.24\times{10}^{-3}$ |  | Cervical cancer | $-4.02\times{10}^{-3}$ |  | Breast cancer | $-3.72\times{10}^{-3}$ |
|  |  | Chronic myeloid leukemia | $-1.71\times{10}^{-3}$ |  | Other leukemia | $-9.87\times{10}^{-4}$ |  | Other leukemia | $-2.75\times{10}^{-3}$ |  | Hodgkin lymphoma | $-3.25\times{10}^{-3}$ |  | Esophageal cancer | $-2.50\times{10}^{-3}$ |
|  |  | Esophageal cancer | $-8.60\times{10}^{-4}$ |  | Esophageal cancer | $-7.85\times{10}^{-4}$ |  | Other malignant neoplasms | $-2.03\times{10}^{-3}$ |  | Non-Hodgkin lymphoma | $-2.92\times{10}^{-3}$ |  | Other leukemia | $-2.46\times{10}^{-3}$ |
|  |  | Other leukemia | $-5.92\times{10}^{-4}$ |  | Chronic myeloid leukemia | $-2.29\times{10}^{-4}$ |  | Breast cancer | $-2.01\times{10}^{-3}$ |  | Ovarian cancer | $-2.68\times{10}^{-3}$ |  | Stomach cancer | $-2.44\times{10}^{-3}$ |
|  |  | Nasopharynx cancer | $-4.58\times{10}^{-4}$ |  | Bladder cancer | $-1.39\times{10}^{-4}$ |  | Colon and rectum cancer | $-1.84\times{10}^{-3}$ |  | Tracheal, bronchus, and lung cancer | $-1.81\times{10}^{-3}$ |  | Liver cancer | $-1.75\times{10}^{-3}$ |
|  |  | Bladder cancer | $-1.84\times{10}^{-4}$ |  | Colon and rectum cancer | $-1.22\times{10}^{-4}$ |  | Tracheal, bronchus, and lung cancer | $-1.26\times{10}^{-3}$ |  | Other leukemia | $-1.03\times{10}^{-3}$ |  | Tracheal, bronchus, and lung cancer | $-1.07\times{10}^{-3}$ |
|  |  | Larynx cancer | $-1.47\times{10}^{-4}$ |  | Nasopharynx cancer | $-1.11\times{10}^{-4}$ |  | Esophageal cancer | $-1.03\times{10}^{-3}$ |  | Chronic myeloid leukemia | $-5.61\times{10}^{-4}$ |  | Bladder cancer | $-8.52E-04$ |
|  |  | Gallbladder and biliary tract cancer | $-1.33\times{10}^{-4}$ |  | Liver cancer | $-1.02\times{10}^{-4}$ |  | Acute lymphoid leukemia | $-7.37\times{10}^{-4}$ |  | Acute myeloid leukemia | $-4.00\times{10}^{-4}$ |  | Colon and rectum cancer | $-7.71\times{10}^{-4}$ |
|  |  | Mesothelioma | $-9.84\times{10}^{-5}$ |  | Uterine cancer | $-7.39\times{10}^{-5}$ |  | Mesothelioma | $-5.20\times{10}^{-4}$ |  | Nasopharynx cancer | $-7.99\times{10}^{-5}$ |  | Non-Hodgkin lymphoma | $-6.39\times{10}^{-4}$ |
|  | **Males** | |  |  |  |  |  |  |  |  |  |  |  |  |  |
|  |  | - | - |  | Stomach cancer | $-1.61\times{10}^{-3}$ |  | Stomach cancer | $-9.06\times{10}^{-3}$ |  | Non-Hodgkin lymphoma | $-9.86\times{10}^{-3}$ |  | Tracheal, bronchus, and lung cancer | $-3.00\times{10}^{-3}$ |
|  |  | - | - |  | Tracheal, bronchus, and lung cancer | $-7.86\times{10}^{-4}$ |  | Tracheal, bronchus, and lung cancer | $-7.16\times{10}^{-3}$ |  | Hodgkin lymphoma | $-7.30\times{10}^{-3}$ |  | Stomach cancer | $-2.14\times{10}^{-3}$ |
|  |  | - | - |  | Other leukemia | $-7.53\times{10}^{-4}$ |  | Other leukemia | $-4.27\times{10}^{-3}$ |  | Tracheal, bronchus, and lung cancer | $-3.66\times{10}^{-3}$ |  | Esophageal cancer | $-2.10\times{10}^{-3}$ |
|  |  | - | - |  | Esophageal cancer | $-5.19\times10^-4$ |  | Colon and rectum cancer | $-2.56\times10^-3$ |  | Other leukemia | $-1.30\times{10}^{-3}$ |  | Other leukemia | $-1.37\times{10}^{-3}$ |
|  |  | - | - |  | Hodgkin lymphoma | $-1.74\times{10}^{-4}$ |  | Esophageal cancer | $-1.37\times{10}^{-3}$ |  | Chronic myeloid leukemia | $-1.18\times{10}^{-3}$ |  | Lip and oral cavity cancer | $-6.74\times{10}^{-4}$ |
|  |  | - | - |  | Lip and oral cavity cancer | $-1.72\times{10}^{-4}$ |  | Larynx cancer | $-8.61\times{10}^{-4}$ |  | Lip and oral cavity cancer | $-9.74\times{10}^{-4}$ |  | Bladder cancer | $-2.28\times{10}^{-4}$ |
|  |  | - | - |  | Larynx cancer | $-8.19\times{10}^{-5}$ |  | Acute lymphoid leukemia | $-6.27\times{10}^{-4}$ |  | Acute myeloid leukemia | $-6.08\times{10}^{-4}$ |  | Larynx cancer | $-2.25\times{10}^{-4}$ |
|  |  | - | - |  | Liver cancer | $-7.23\times{10}^{-5}$ |  | Chronic myeloid leukemia | $-5.16\times{10}^{-4}$ |  | Bladder cancer | $-3.61\times{10}^{-4}$ |  | Nasopharynx cancer | $-1.19\times{10}^{-4}$ |
|  |  | - | - |  | Nasopharynx cancer | $-6.55\times{10}^{-5}$ |  | Acute myeloid leukemia | $-2.40\times{10}^{-4}$ |  | Brain and central nervous system cancer | $-2.66\times{10}^{-4}$ |  | Mesothelioma | $-7.76\times{10}^{-5}$ |
|  |  | - | - |  | Gallbladder and biliary tract cancer | $-7.68\times{10}^{-6}$ |  | Chronic lymphoid leukemia | $-6.93\times{10}^{-5}$ |  | Larynx cancer | $-2.31\times{10}^{-4}$ |  | Chronic myeloid leukemia | $-7.72\times{10}^{-5}$ |
| **Leading cancer types by region and sex resulting in increased AYA cancer death rate** | | | | | | | | | | | | |  |  |  |
|  |  | **Oceania** | |  | **South Asia** | |  | **Western Sub-Saharan Africa** | |  | **Eastern Sub-Saharan Africa** | |  |  | |
|  |  | **Cancer type** | **Absolute change in rate, 1990-2019** |  | **Cancer type** | **Absolute change in rate, 1990-2019** |  | **Cancer type** | **Absolute change in rate, 1990-2019** |  | **Cancer type** | **Absolute change in rate, 1990-2019** |  |  |  |
|  | **Females** | |  |  |  |  |  |  |  |  |  |  |  |  |  |
|  |  | Breast cancer | $7.63\times{10}^{-3}$ |  | - | - |  | - | - |  | - | - |  |  |  |
|  |  | Ovarian cancer | $4.26\times{10}^{-4}$ |  | - | - |  | - | - |  | - | - |  |  |  |
|  |  | Tracheal, bronchus, and lung cancer | $2.54\times{10}^{-4}$ |  | - | - |  | - | - |  | - | - |  |  |  |
|  |  | Uterine cancer | $2.22\times{10}^{-4}$ |  | - | - |  | - | - |  | - | - |  |  |  |
|  |  | Colon and rectum cancer | $1.85\times{10}^{-4}$ |  | - | - |  | - | - |  | - | - |  |  |  |
|  |  | Brain and central nervous system cancer | $1.33\times{10}^{-4}$ |  | - | - |  | - | - |  | - | - |  |  |  |
|  |  | Pancreatic cancer | $7.95\times{10}^{-5}$ |  | - | - |  | - | - |  | - | - |  |  |  |
|  |  | Bladder cancer | $4.66\times{10}^{-5}$ |  | - | - |  | - | - |  | - | - |  |  |  |
|  |  | Nasopharynx cancer | $3.14\times{10}^{-5}$ |  | - | - |  | - | - |  | - | - |  |  |  |
|  |  | Lip and oral cavity cancer | $2.09\times{10}^{-5}$ |  | - | - |  | - | - |  | - | - |  |  |  |
|  | **Males** | |  |  |  |  |  |  |  |  |  |  |  |  |  |
|  |  | Tracheal, bronchus, and lung cancer | $6.65\times{10}^{-4}$ |  | Other malignant neoplasms | $1.74\times{10}^{-3}$ |  | Brain and central nervous system cancer | $6.49\times{10}^{-4}$ |  | Other malignant neoplasms | $1.98\times{10}^{-3}$ |  |  |  |
|  |  | Colon and rectum cancer | $3.97\times{10}^{-4}$ |  | Non-Hodgkin lymphoma | $1.44\times{10}^{-3}$ |  | Other malignant neoplasms | $5.52\times{10}^{-4}$ |  | Colon and rectum cancer | $4.27\times{10}^{-4}$ |  |  |  |
|  |  | Brain and central nervous system cancer | $3.52\times{10}^{-4}$ |  | Lip and oral cavity cancer | $1.26\times{10}^{-3}$ |  | Colon and rectum cancer | $4.09\times{10}^{-4}$ |  | Brain and central nervous system cancer | $3.92\times{10}^{-4}$ |  |  |  |
|  |  | Stomach cancer | $1.77\times{10}^{-4}$ |  | Other pharynx cancer | $1.21\times{10}^{-3}$ |  | Pancreatic cancer | $3.25\times{10}^{-4}$ |  | Liver cancer | $2.02\times{10}^{-4}$ |  |  |  |
|  |  | Other malignant neoplasms | $1.73\times{10}^{-4}$ |  | Colon and rectum cancer | $9.42\times{10}^{-4}$ |  | Non-Hodgkin lymphoma | $2.46\times{10}^{-4}$ |  | Non-Hodgkin lymphoma | $1.92\times{10}^{-4}$ |  |  |  |
|  |  | Pancreatic cancer | $9.70\times{10}^{-5}$ |  | Tracheal, bronchus, and lung cancer | $7.25\times{10}^{-4}$ |  | Tracheal, bronchus, and lung cancer | $1.90\times{10}^{-4}$ |  | Testicular cancer | $1.88\times{10}^{-4}$ |  |  |  |
|  |  | Bladder cancer | $7.88\times{10}^{-5}$ |  | Brain and central nervous system cancer | $6.58\times{10}^{-4}$ |  | Other leukemia | $1.82\times{10}^{-4}$ |  | Acute myeloid leukemia | $1.83\times{10}^{-4}$ |  |  |  |
|  |  | Lip and oral cavity cancer | $5.26\times{10}^{-5}$ |  | Acute myeloid leukemia | $3.70\times{10}^{-4}$ |  | Acute myeloid leukemia | $1.32\times{10}^{-4}$ |  | Pancreatic cancer | $1.56\times{10}^{-4}$ |  |  |  |
|  |  | Prostate cancer | $5.05\times{10}^{-5}$ |  | Pancreatic cancer | $3.53\times{10}^{-4}$ |  | Bladder cancer | $1.30\times{10}^{-4}$ |  | Malignant skin melanoma | $8.52\times{10}^{-5}$ |  |  |  |
|  |  | Esophageal cancer | $4.16\times{10}^{-5}$ |  | Kidney cancer | $2.06\times{10}^{-4}$ |  | Esophageal cancer | $1.30\times{10}^{-4}$ |  | Kidney cancer | $7.76\times{10}^{-5}$ |  |  |  |
| **Leading cancer types by region and sex resulting in increased AYA cancer DALY rate** | | | | | | | | | | | | | | | |
|  |  | **Oceania** | |  | **South Asia** | |  | **Western Sub-Saharan Africa** | |  | **Eastern Sub-Saharan Africa** | |  | **Central Latin America** | |
|  |  | **Cancer type** | **Absolute change in rate, 1990-2019** |  | **Cancer type** | **Absolute change in rate, 1990-2019** |  | **Cancer type** | **Absolute change in rate, 1990-2019** |  | **Cancer type** | **Absolute change in rate, 1990-2019** |  | **Cancer type** | **Absolute change in rate, 1990-2019** |
|  | **Females** | |  |  |  |  |  |  |  |  |  |  |  |  |  |
|  |  | Breast cancer | $3.70\times{10}^{-1}$ |  | - | - |  | - | - |  | - | - |  | - | - |
|  |  | Ovarian cancer | $2.17\times10^-2$ |  | - | - |  | - | - |  | - | - |  | - | - |
|  |  | Tracheal, bronchus, and lung cancer | $1.24\times{10}^{-2}$ |  | - | - |  | - | - |  | - | - |  | - | - |
|  |  | Uterine cancer | $1.11\times{10}^{-2}$ |  | - | - |  | - | - |  | - | - |  | - | - |
|  |  | Colon and rectum cancer | $9.46\times{10}^{-3}$ |  | - | - |  | - | - |  | - | - |  | - | - |
|  |  | Brain and central nervous system cancer | $7.15\times{10}^{-3}$ |  | - | - |  | - | - |  | - | - |  | - | - |
|  |  | Pancreatic cancer | $3.98\times{10}^{-3}$ |  | - | - |  | - | - |  | - | - |  | - | - |
|  |  | Bladder cancer | $2.27\times{10}^{-3}$ |  | - | - |  | - | - |  | - | - |  | - | - |
|  |  | Nasopharynx cancer | $1.58\times{10}^{-3}$ |  | - | - |  | - | - |  | - | - |  | - | - |
|  |  | Lip and oral cavity cancer | $1.16\times{10}^{-3}$ |  | - | - |  | - | - |  | - | - |  | - | - |
|  | **Males** | |  |  |  |  |  |  |  |  |  |  |  |  |  |
|  |  | Tracheal, bronchus, and lung cancer | $3.55\times{10}^{-2}$ |  | Other malignant neoplasms | $9.05\times{10}^{-2}$ |  | Brain and central nervous system cancer | $3.29\times{10}^{-2}$ |  | Other malignant neoplasms | $1.05\times{10}^{-1}$ |  | Testicular cancer | $7.89\times{10}^{-2}$ |
|  |  | Colon and rectum cancer | $2.02\times{10}^{-2}$ |  | Non-Hodgkin lymphoma | $7.51\times{10}^{-2}$ |  | Other malignant neoplasms | $2.89\times{10}^{-2}$ |  | Colon and rectum cancer | $2.06\times{10}^{-2}$ |  | Colon and rectum cancer | $5.95\times{10}^{-2}$ |
|  |  | Brain and central nervous system cancer | $1.86\times{10}^{-2}$ |  | Lip and oral cavity cancer | $6.07\times{10}^{-2}$ |  | Colon and rectum cancer | $2.00\times{10}^{-2}$ |  | Brain and central nervous system cancer | $2.00\times{10}^{-2}$ |  | Acute lymphoid leukemia | $5.76\times{10}^{-2}$ |
|  |  | Other malignant neoplasms | $1.16\times{10}^{-2}$ |  | Other pharynx cancer | $5.73\times{10}^{-2}$ |  | Pancreatic cancer | $1.55\times{10}^{-2}$ |  | Liver cancer | $9.84\times{10}^{-3}$ |  | Brain and central nervous system cancer | $3.21\times{10}^{-2}$ |
|  |  | Stomach cancer | $1.02\times{10}^{-2}$ |  | Colon and rectum cancer | $4.49\times{10}^{-2}$ |  | Non-Hodgkin lymphoma | $1.05\times{10}^{-2}$ |  | Testicular cancer | $9.76\times{10}^{-3}$ |  | Acute myeloid leukemia | $2.34\times{10}^{-2}$ |
|  |  | Pancreatic cancer | $4.55\times{10}^{-3}$ |  | Tracheal, bronchus, and lung cancer | $3.45\times{10}^{-2}$ |  | Other leukemia | $8.95\times{10}^{-3}$ |  | Non-Hodgkin lymphoma | $9.54\times{10}^{-3}$ |  | Kidney cancer | $1.04\times{10}^{-2}$ |
|  |  | Bladder cancer | $3.77\times{10}^{-3}$ |  | Brain and central nervous system cancer | $3.24\times{10}^{-2}$ |  | Tracheal, bronchus, and lung cancer | $8.90\times{10}^{-3}$ |  | Acute myeloid leukemia | $9.46\times{10}^{-3}$ |  | Pancreatic cancer | $6.90\times{10}^{-3}$ |
|  |  | Prostate cancer | $2.81\times{10}^{-3}$ |  | Acute myeloid leukemia | $1.85\times{10}^{-2}$ |  | Acute myeloid leukemia | $6.90\times{10}^{-3}$ |  | Pancreatic cancer | $7.37\times{10}^{-3}$ |  | Malignant skin melanoma | $3.80\times{10}^{-3}$ |
|  |  | Lip and oral cavity cancer | $2.74\times{10}^{-3}$ |  | Pancreatic cancer | $1.65\times{10}^{-2}$ |  | Bladder cancer | $6.39\times{10}^{-3}$ |  | Malignant skin melanoma | $4.18\times{10}^{-3}$ |  | Other malignant neoplasms | $2.96\times{10}^{-3}$ |
|  |  | Esophageal cancer | $1.93\times{10}^{-3}$ |  | Thyroid cancer | $1.03\times{10}^{-2}$ |  | Esophageal cancer | $6.12\times{10}^{-3}$ |  | Kidney cancer | $3.79\times{10}^{-3}$ |  | Multiple myeloma | $1.42\times{10}^{-3}$ |

GBD regions demonstrating temporal trends at odds with global trends are selected for reporting. The majority of GBD regions demonstrated increased AYA cancer incidence rate and decreased death and DALY rates. Therefore, GBD regions with decreased incidence rate and increased death and DALY rates are selected for reporting. Within selected GBD regions, top 10 cancer types leading to increased incidence rate, decreased death rate, and decreased DALY rate from 1990 to 2019 are reported. Negative value indicates lower age-standardised rates in 2019 compared to 1990.

## eTable 8. AYA cancer ranking by the number of incident cases at the global level and according to SDI quintile, super-regions, regions, and countries, both sexes, 2019

| **Location** | | **Breast cancer** | **Other malignant neoplasms** | **Cervical cancer** | **Colon and rectum cancer** | **Brain and central nervous system cancer** | **Testicular cancer** | **Non-Hodgkin lymphoma** | **Stomach cancer** | **Thyroid cancer** | **Acute lymphoid leukemia** | **Malignant skin melanoma** | **Ovarian cancer** | **Hodgkin lymphoma** | **Tracheal, bronchus, and lung cancer** | **Lip and oral cavity cancer** | **Other leukemia** | **Nasopharynx cancer** | **Liver cancer** | **Kidney cancer** | **Acute myeloid leukemia** | **Uterine cancer** | **Bladder cancer** | **Pancreatic cancer** | **Chronic myeloid leukemia** | **Esophageal cancer** | **Other pharynx cancer** | **Prostate cancer** | **Chronic lymphoid leukemia** | **Larynx cancer** | **Gallbladder and biliary tract cancer** | **Multiple myeloma** | **Mesothelioma** |
| --- | --- | --- | --- | --- | --- | --- | --- | --- | --- | --- | --- | --- | --- | --- | --- | --- | --- | --- | --- | --- | --- | --- | --- | --- | --- | --- | --- | --- | --- | --- | --- | --- | --- |
| **Global** | | **1** | **2** | **3** | **4** | **5** | **6** | **7** | **8** | **9** | **10** | **11** | **12** | **13** | **14** | **15** | **16** | **17** | **18** | **19** | **20** | **21** | **22** | **23** | **24** | **25** | **26** | **27** | **28** | **29** | **30** | **31** | **32** |
|  | **Low SDI** | **3** | **2** | **1** | **10** | **8** | **18** | **7** | **6** | **4** | **16** | **21** | **9** | **11** | **15** | **5** | **12** | **20** | **14** | **25** | **13** | **24** | **22** | **26** | **17** | **19** | **23** | **30** | **32** | **27** | **28** | **29** | **31** |
|  | **Low-middle SDI** | **2** | **3** | **1** | **8** | **9** | **15** | **6** | **5** | **7** | **17** | **26** | **10** | **13** | **11** | **4** | **12** | **19** | **16** | **20** | **14** | **23** | **24** | **25** | **21** | **22** | **18** | **29** | **32** | **27** | **28** | **30** | **31** |
|  | **Middle SDI** | **1** | **2** | **3** | **4** | **5** | **12** | **6** | **7** | **8** | **14** | **23** | **10** | **19** | **11** | **16** | **9** | **15** | **13** | **18** | **17** | **20** | **21** | **22** | **26** | **24** | **27** | **28** | **25** | **29** | **30** | **31** | **32** |
|  | **High-middle SDI** | **1** | **2** | **4** | **3** | **6** | **5** | **9** | **7** | **11** | **8** | **12** | **15** | **13** | **14** | **22** | **17** | **10** | **19** | **16** | **20** | **18** | **21** | **23** | **24** | **25** | **28** | **27** | **26** | **29** | **30** | **31** | **32** |
|  | **High SDI** | **1** | **2** | **6** | **5** | **7** | **4** | **11** | **14** | **10** | **8** | **3** | **12** | **9** | **16** | **17** | **23** | **21** | **22** | **13** | **18** | **15** | **20** | **25** | **19** | **27** | **26** | **24** | **30** | **31** | **28** | **29** | **32** |
| **Central Asia** | | **1** | **3** | **2** | **5** | **4** | **8** | **7** | **6** | **16** | **17** | **21** | **9** | **12** | **10** | **19** | **13** | **26** | **18** | **15** | **14** | **11** | **20** | **23** | **28** | **22** | **27** | **24** | **30** | **25** | **32** | **29** | **31** |
|  | Armenia | 1 | 3 | 2 | 6 | 4 | 5 | 8 | 11 | 13 | 14 | 16 | 12 | 15 | 7 | 22 | 10 | 27 | 21 | 17 | 18 | 9 | 19 | 20 | 29 | 30 | 32 | 24 | 25 | 26 | 31 | 28 | 23 |
|  | Azerbaijan | 1 | 2 | 3 | 4 | 5 | 12 | 10 | 8 | 16 | 17 | 25 | 11 | 9 | 7 | 22 | 6 | 26 | 24 | 13 | 14 | 15 | 18 | 20 | 27 | 19 | 28 | 21 | 31 | 23 | 30 | 29 | 32 |
|  | Georgia | 1 | 4 | 3 | 5 | 8 | 2 | 10 | 12 | 14 | 20 | 16 | 13 | 9 | 7 | 21 | 11 | 29 | 23 | 15 | 17 | 6 | 19 | 22 | 25 | 26 | 27 | 18 | 32 | 24 | 30 | 28 | 31 |
|  | Kazakhstan | 2 | 3 | 1 | 5 | 6 | 7 | 10 | 11 | 9 | 17 | 16 | 4 | 8 | 14 | 18 | 23 | 25 | 19 | 15 | 13 | 12 | 20 | 22 | 26 | 24 | 27 | 21 | 29 | 28 | 32 | 30 | 31 |
|  | Kyrgyzstan | 3 | 2 | 1 | 7 | 5 | 8 | 11 | 4 | 14 | 16 | 19 | 6 | 17 | 13 | 18 | 12 | 23 | 21 | 15 | 9 | 10 | 22 | 20 | 27 | 24 | 25 | 26 | 29 | 28 | 30 | 31 | 32 |
|  | Mongolia | 5 | 4 | 1 | 6 | 7 | 25 | 10 | 3 | 18 | 17 | 26 | 11 | 19 | 12 | 15 | 22 | 28 | 2 | 13 | 9 | 8 | 20 | 16 | 24 | 14 | 30 | 21 | 29 | 32 | 23 | 27 | 31 |
|  | Tajikistan | 2 | 3 | 7 | 5 | 1 | 32 | 6 | 4 | 24 | 14 | 21 | 13 | 20 | 9 | 18 | 10 | 28 | 15 | 11 | 12 | 8 | 17 | 19 | 27 | 16 | 26 | 25 | 30 | 23 | 31 | 29 | 22 |
|  | Turkmenistan | 2 | 1 | 3 | 8 | 4 | 6 | 9 | 10 | 15 | 19 | 23 | 7 | 5 | 13 | 18 | 12 | 26 | 16 | 11 | 14 | 24 | 22 | 25 | 29 | 21 | 28 | 17 | 31 | 30 | 32 | 20 | 27 |
|  | Uzbekistan | 1 | 4 | 2 | 6 | 3 | 12 | 5 | 7 | 21 | 16 | 22 | 14 | 13 | 10 | 15 | 9 | 24 | 18 | 17 | 11 | 8 | 19 | 23 | 27 | 20 | 26 | 29 | 28 | 25 | 32 | 30 | 31 |
| **Central Europe** | | **2** | **5** | **3** | **7** | **6** | **1** | **9** | **16** | **11** | **14** | **4** | **10** | **8** | **12** | **19** | **21** | **22** | **24** | **15** | **17** | **13** | **18** | **20** | **25** | **29** | **23** | **28** | **27** | **26** | **30** | **31** | **32** |
|  | Albania | 2 | 3 | 6 | 5 | 4 | 1 | 9 | 11 | 13 | 12 | 16 | 14 | 7 | 8 | 21 | 17 | 26 | 20 | 18 | 15 | 10 | 23 | 22 | 27 | 28 | 29 | 19 | 25 | 24 | 30 | 31 | 32 |
|  | Bosnia and Herzegovina | 1 | 6 | 3 | 4 | 5 | 2 | 8 | 13 | 15 | 14 | 7 | 10 | 9 | 11 | 21 | 22 | 29 | 19 | 17 | 16 | 12 | 20 | 18 | 24 | 30 | 23 | 26 | 27 | 25 | 28 | 31 | 32 |
|  | Bulgaria | 1 | 5 | 2 | 4 | 6 | 3 | 8 | 13 | 17 | 20 | 9 | 12 | 10 | 11 | 19 | 15 | 23 | 22 | 14 | 16 | 7 | 18 | 21 | 29 | 26 | 25 | 27 | 31 | 24 | 30 | 28 | 32 |
|  | Croatia | 2 | 3 | 6 | 7 | 4 | 1 | 9 | 16 | 12 | 10 | 5 | 11 | 8 | 13 | 19 | 23 | 22 | 24 | 14 | 18 | 15 | 17 | 21 | 20 | 31 | 26 | 25 | 29 | 27 | 30 | 28 | 32 |
|  | Czechia | 3 | 6 | 4 | 8 | 7 | 1 | 10 | 16 | 9 | 14 | 2 | 11 | 5 | 15 | 19 | 22 | 26 | 25 | 12 | 18 | 13 | 17 | 21 | 20 | 29 | 23 | 24 | 31 | 27 | 28 | 30 | 32 |
|  | Hungary | 2 | 5 | 3 | 6 | 7 | 1 | 8 | 18 | 14 | 12 | 4 | 10 | 9 | 11 | 17 | 22 | 23 | 26 | 13 | 16 | 15 | 19 | 20 | 24 | 29 | 21 | 28 | 27 | 25 | 30 | 31 | 32 |
|  | Montenegro | 1 | 8 | 4 | 9 | 5 | 2 | 10 | 17 | 11 | 12 | 6 | 13 | 3 | 7 | 22 | 26 | 29 | 21 | 16 | 15 | 14 | 18 | 20 | 23 | 28 | 27 | 24 | 25 | 19 | 31 | 30 | 32 |
|  | North Macedonia | 1 | 5 | 3 | 7 | 6 | 2 | 12 | 14 | 16 | 18 | 4 | 11 | 8 | 10 | 22 | 13 | 27 | 21 | 15 | 19 | 9 | 17 | 20 | 28 | 31 | 26 | 23 | 25 | 24 | 29 | 30 | 32 |
|  | Poland | 2 | 4 | 7 | 8 | 5 | 1 | 9 | 17 | 11 | 12 | 3 | 10 | 6 | 13 | 20 | 22 | 24 | 28 | 16 | 15 | 14 | 18 | 19 | 21 | 27 | 23 | 29 | 26 | 25 | 31 | 30 | 32 |
|  | Romania | 2 | 4 | 1 | 5 | 6 | 3 | 7 | 15 | 12 | 16 | 8 | 10 | 9 | 11 | 19 | 18 | 22 | 25 | 14 | 17 | 13 | 20 | 21 | 29 | 28 | 23 | 27 | 26 | 24 | 31 | 30 | 32 |
|  | Serbia | 2 | 4 | 3 | 7 | 5 | 1 | 9 | 15 | 13 | 16 | 6 | 10 | 8 | 11 | 19 | 18 | 24 | 23 | 14 | 17 | 12 | 21 | 20 | 27 | 29 | 26 | 28 | 22 | 25 | 30 | 31 | 32 |
|  | Slovakia | 2 | 4 | 3 | 6 | 9 | 1 | 8 | 15 | 13 | 12 | 5 | 10 | 7 | 16 | 18 | 24 | 22 | 21 | 14 | 17 | 11 | 19 | 20 | 29 | 30 | 23 | 26 | 31 | 28 | 25 | 27 | 32 |
|  | Slovenia | 3 | 4 | 5 | 7 | 8 | 1 | 9 | 12 | 15 | 10 | 2 | 11 | 6 | 13 | 19 | 26 | 25 | 24 | 14 | 17 | 18 | 20 | 22 | 16 | 31 | 23 | 21 | 30 | 27 | 28 | 29 | 32 |
| **Eastern Europe** | | **1** | **3** | **2** | **6** | **12** | **4** | **10** | **11** | **8** | **16** | **7** | **13** | **5** | **15** | **17** | **21** | **25** | **22** | **14** | **19** | **9** | **20** | **18** | **26** | **28** | **23** | **24** | **29** | **27** | **32** | **31** | **30** |
|  | Belarus | 3 | 1 | 2 | 7 | 10 | 5 | 9 | 8 | 11 | 14 | 6 | 13 | 4 | 16 | 20 | 26 | 27 | 24 | 12 | 17 | 15 | 22 | 21 | 19 | 29 | 28 | 18 | 30 | 25 | 32 | 31 | 23 |
|  | Estonia | 1 | 4 | 3 | 7 | 10 | 5 | 9 | 11 | 8 | 12 | 2 | 13 | 6 | 17 | 20 | 18 | 25 | 24 | 15 | 16 | 14 | 19 | 22 | 21 | 30 | 28 | 23 | 27 | 26 | 31 | 29 | 32 |
|  | Latvia | 1 | 2 | 9 | 8 | 6 | 3 | 7 | 10 | 14 | 16 | 5 | 13 | 4 | 15 | 21 | 17 | 29 | 22 | 12 | 18 | 11 | 20 | 19 | 24 | 27 | 25 | 23 | 28 | 26 | 31 | 30 | 32 |
|  | Lithuania | 1 | 2 | 5 | 8 | 7 | 3 | 9 | 11 | 12 | 17 | 4 | 10 | 6 | 16 | 19 | 23 | 26 | 22 | 13 | 15 | 14 | 21 | 18 | 24 | 28 | 27 | 20 | 30 | 25 | 31 | 29 | 32 |
|  | Republic of Moldova | 2 | 3 | 1 | 4 | 8 | 6 | 7 | 10 | 13 | 21 | 9 | 14 | 5 | 12 | 20 | 17 | 22 | 26 | 15 | 18 | 11 | 19 | 16 | 29 | 28 | 23 | 25 | 31 | 24 | 30 | 27 | 32 |
|  | Russian Federation | 1 | 3 | 2 | 6 | 13 | 4 | 12 | 10 | 9 | 16 | 8 | 11 | 5 | 15 | 17 | 23 | 28 | 22 | 14 | 20 | 7 | 18 | 19 | 25 | 27 | 21 | 24 | 29 | 26 | 31 | 30 | 32 |
|  | Ukraine | 1 | 2 | 4 | 7 | 5 | 9 | 3 | 10 | 11 | 16 | 8 | 12 | 6 | 13 | 18 | 20 | 21 | 23 | 14 | 17 | 15 | 22 | 19 | 27 | 29 | 25 | 24 | 30 | 26 | 32 | 31 | 28 |
| **Australasia** | | **2** | **5** | **7** | **4** | **8** | **3** | **10** | **17** | **9** | **13** | **1** | **12** | **6** | **15** | **14** | **25** | **22** | **20** | **11** | **16** | **18** | **19** | **23** | **24** | **27** | **28** | **21** | **29** | **31** | **30** | **26** | **32** |
|  | Australia | 2 | 6 | 7 | 4 | 9 | 3 | 10 | 16 | 8 | 14 | 1 | 12 | 5 | 15 | 13 | 25 | 22 | 21 | 11 | 17 | 19 | 18 | 23 | 24 | 27 | 28 | 20 | 29 | 30 | 31 | 26 | 32 |
|  | New Zealand | 2 | 4 | 9 | 5 | 6 | 3 | 7 | 16 | 13 | 10 | 1 | 11 | 8 | 17 | 15 | 24 | 25 | 19 | 12 | 14 | 18 | 21 | 23 | 22 | 27 | 30 | 20 | 29 | 31 | 28 | 26 | 32 |
| **High-income Asia Pacific** | | **1** | **2** | **4** | **5** | **7** | **8** | **10** | **6** | **9** | **3** | **14** | **12** | **13** | **16** | **22** | **23** | **21** | **15** | **17** | **18** | **11** | **20** | **24** | **19** | **28** | **29** | **26** | **30** | **27** | **25** | **31** | **32** |
|  | Brunei Darussalam | 1 | 4 | 2 | 5 | 7 | 8 | 3 | 12 | 9 | 22 | 21 | 6 | 11 | 13 | 19 | 20 | 10 | 15 | 18 | 14 | 17 | 23 | 24 | 16 | 30 | 27 | 28 | 25 | 31 | 26 | 29 | 32 |
|  | Japan | 1 | 2 | 4 | 5 | 7 | 6 | 10 | 8 | 11 | 3 | 13 | 12 | 14 | 15 | 20 | 24 | 22 | 21 | 17 | 18 | 9 | 19 | 23 | 16 | 27 | 29 | 25 | 30 | 28 | 26 | 31 | 32 |
|  | Republic of Korea | 1 | 3 | 5 | 7 | 8 | 13 | 9 | 4 | 2 | 6 | 15 | 12 | 14 | 11 | 23 | 22 | 21 | 10 | 18 | 17 | 16 | 20 | 24 | 19 | 29 | 30 | 26 | 27 | 28 | 25 | 31 | 32 |
|  | Singapore | 1 | 3 | 7 | 5 | 6 | 12 | 8 | 14 | 9 | 4 | 17 | 11 | 10 | 13 | 22 | 25 | 2 | 18 | 16 | 19 | 15 | 21 | 23 | 20 | 28 | 27 | 24 | 30 | 26 | 29 | 31 | 32 |
| **High-income North America** | | **2** | **1** | **6** | **5** | **8** | **4** | **9** | **18** | **10** | **14** | **3** | **13** | **7** | **15** | **16** | **21** | **24** | **22** | **11** | **17** | **12** | **20** | **23** | **25** | **27** | **26** | **19** | **31** | **30** | **29** | **28** | **32** |
|  | Canada | 2 | 1 | 5 | 9 | 6 | 3 | 10 | 16 | 11 | 8 | 4 | 13 | 7 | 15 | 19 | 23 | 22 | 21 | 12 | 18 | 17 | 20 | 25 | 14 | 26 | 28 | 24 | 29 | 31 | 30 | 27 | 32 |
|  | Greenland | 2 | 3 | 1 | 4 | 6 | 5 | 9 | 11 | 15 | 21 | 13 | 7 | 14 | 8 | 20 | 26 | 10 | 17 | 12 | 19 | 25 | 24 | 16 | 29 | 18 | 23 | 28 | 22 | 32 | 27 | 30 | 31 |
|  | United States of America | 1 | 2 | 6 | 5 | 8 | 4 | 9 | 17 | 10 | 18 | 3 | 13 | 7 | 15 | 14 | 21 | 24 | 22 | 11 | 16 | 12 | 20 | 23 | 25 | 27 | 26 | 19 | 31 | 30 | 29 | 28 | 32 |
| **Southern Latin America** | | **3** | **4** | **2** | **5** | **7** | **1** | **6** | **14** | **12** | **13** | **9** | **8** | **11** | **16** | **20** | **17** | **27** | **26** | **10** | **15** | **18** | **21** | **19** | **25** | **24** | **32** | **23** | **31** | **29** | **22** | **28** | **30** |
|  | Argentina | 3 | 4 | 1 | 5 | 7 | 2 | 6 | 16 | 12 | 15 | 9 | 8 | 11 | 13 | 20 | 14 | 27 | 26 | 10 | 17 | 18 | 21 | 19 | 25 | 23 | 31 | 24 | 32 | 28 | 22 | 29 | 30 |
|  | Chile | 3 | 4 | 2 | 6 | 7 | 1 | 5 | 12 | 11 | 9 | 8 | 14 | 10 | 17 | 22 | 16 | 26 | 25 | 13 | 15 | 21 | 19 | 20 | 23 | 28 | 31 | 24 | 29 | 32 | 18 | 27 | 30 |
|  | Uruguay | 3 | 5 | 1 | 6 | 8 | 2 | 4 | 15 | 13 | 17 | 9 | 11 | 10 | 12 | 18 | 14 | 22 | 26 | 7 | 16 | 20 | 21 | 19 | 23 | 27 | 30 | 24 | 32 | 28 | 25 | 29 | 31 |
| **Western Europe** | | **1** | **4** | **8** | **9** | **6** | **3** | **10** | **18** | **11** | **5** | **2** | **12** | **7** | **15** | **19** | **23** | **21** | **22** | **13** | **17** | **16** | **20** | **24** | **14** | **27** | **26** | **25** | **28** | **31** | **30** | **29** | **32** |
|  | Andorra | 2 | 4 | 7 | 10 | 1 | 6 | 9 | 16 | 12 | 3 | 5 | 17 | 8 | 13 | 20 | 25 | 22 | 19 | 14 | 18 | 15 | 21 | 24 | 11 | 27 | 26 | 23 | 30 | 31 | 28 | 29 | 32 |
|  | Austria | 2 | 4 | 8 | 9 | 6 | 1 | 10 | 20 | 11 | 5 | 3 | 13 | 7 | 15 | 21 | 24 | 26 | 22 | 16 | 17 | 12 | 18 | 23 | 14 | 27 | 25 | 19 | 28 | 30 | 31 | 29 | 32 |
|  | Belgium | 1 | 3 | 8 | 9 | 4 | 5 | 10 | 21 | 11 | 6 | 2 | 12 | 7 | 13 | 16 | 20 | 22 | 23 | 15 | 17 | 14 | 19 | 25 | 18 | 27 | 26 | 24 | 30 | 28 | 31 | 29 | 32 |
|  | Cyprus | 1 | 2 | 11 | 8 | 5 | 6 | 9 | 14 | 12 | 4 | 3 | 10 | 7 | 15 | 16 | 20 | 23 | 24 | 13 | 17 | 21 | 18 | 25 | 19 | 31 | 27 | 22 | 26 | 29 | 30 | 28 | 32 |
|  | Denmark | 4 | 5 | 7 | 9 | 3 | 2 | 10 | 20 | 11 | 8 | 1 | 12 | 6 | 13 | 19 | 24 | 25 | 21 | 14 | 15 | 16 | 18 | 22 | 17 | 28 | 23 | 26 | 27 | 31 | 30 | 29 | 32 |
|  | Finland | 3 | 4 | 12 | 9 | 2 | 5 | 8 | 14 | 10 | 7 | 1 | 11 | 6 | 18 | 15 | 24 | 25 | 19 | 13 | 17 | 16 | 21 | 23 | 22 | 28 | 26 | 20 | 29 | 31 | 30 | 27 | 32 |
|  | France | 1 | 4 | 8 | 9 | 5 | 3 | 10 | 20 | 14 | 6 | 2 | 12 | 7 | 11 | 18 | 21 | 23 | 22 | 13 | 19 | 15 | 17 | 25 | 16 | 27 | 26 | 24 | 29 | 28 | 31 | 30 | 32 |
|  | Germany | 3 | 2 | 7 | 8 | 9 | 1 | 11 | 17 | 10 | 5 | 4 | 14 | 6 | 15 | 19 | 28 | 24 | 22 | 16 | 12 | 18 | 23 | 20 | 13 | 26 | 27 | 21 | 25 | 31 | 29 | 30 | 32 |
|  | Greece | 1 | 5 | 8 | 9 | 4 | 2 | 10 | 15 | 16 | 7 | 6 | 12 | 3 | 13 | 22 | 21 | 17 | 25 | 14 | 19 | 11 | 18 | 23 | 20 | 29 | 31 | 24 | 26 | 27 | 30 | 28 | 32 |
|  | Iceland | 3 | 4 | 9 | 10 | 1 | 11 | 8 | 18 | 6 | 5 | 2 | 13 | 7 | 14 | 19 | 23 | 25 | 22 | 12 | 16 | 17 | 20 | 21 | 15 | 26 | 29 | 24 | 27 | 30 | 31 | 28 | 32 |
|  | Ireland | 2 | 3 | 8 | 9 | 5 | 4 | 10 | 17 | 12 | 6 | 1 | 11 | 7 | 16 | 20 | 26 | 23 | 22 | 13 | 18 | 15 | 19 | 24 | 14 | 25 | 27 | 21 | 31 | 30 | 29 | 28 | 32 |
|  | Israel | 1 | 3 | 10 | 9 | 4 | 8 | 7 | 14 | 11 | 6 | 2 | 12 | 5 | 15 | 18 | 22 | 21 | 26 | 16 | 13 | 17 | 20 | 23 | 19 | 28 | 30 | 24 | 25 | 29 | 31 | 27 | 32 |
|  | Italy | 1 | 5 | 11 | 8 | 9 | 4 | 7 | 16 | 10 | 2 | 3 | 12 | 6 | 17 | 21 | 22 | 20 | 23 | 13 | 19 | 14 | 18 | 25 | 15 | 31 | 28 | 24 | 27 | 30 | 29 | 26 | 32 |
|  | Luxembourg | 2 | 5 | 10 | 9 | 4 | 3 | 8 | 20 | 11 | 6 | 1 | 12 | 7 | 13 | 18 | 15 | 22 | 23 | 21 | 16 | 14 | 19 | 24 | 17 | 27 | 25 | 26 | 28 | 29 | 31 | 30 | 32 |
|  | Malta | 1 | 3 | 11 | 9 | 5 | 2 | 8 | 19 | 10 | 7 | 4 | 12 | 6 | 16 | 21 | 25 | 13 | 24 | 14 | 17 | 15 | 20 | 22 | 18 | 28 | 26 | 23 | 30 | 27 | 31 | 29 | 32 |
|  | Monaco | 2 | 6 | 12 | 9 | 7 | 1 | 5 | 19 | 13 | 8 | 3 | 11 | 4 | 10 | 24 | 14 | 26 | 20 | 16 | 18 | 23 | 17 | 21 | 15 | 28 | 29 | 22 | 30 | 27 | 31 | 25 | 32 |
|  | Netherlands | 2 | 4 | 9 | 8 | 6 | 3 | 10 | 18 | 12 | 5 | 1 | 11 | 7 | 13 | 20 | 21 | 23 | 22 | 14 | 19 | 17 | 16 | 25 | 15 | 26 | 27 | 24 | 30 | 31 | 28 | 29 | 32 |
|  | Norway | 4 | 5 | 7 | 8 | 2 | 3 | 9 | 18 | 11 | 10 | 1 | 12 | 6 | 14 | 19 | 24 | 25 | 20 | 13 | 17 | 16 | 15 | 23 | 22 | 30 | 26 | 21 | 28 | 31 | 29 | 27 | 32 |
|  | Portugal | 1 | 2 | 8 | 4 | 6 | 3 | 9 | 11 | 13 | 5 | 7 | 15 | 10 | 16 | 19 | 22 | 20 | 23 | 14 | 17 | 12 | 18 | 26 | 21 | 27 | 25 | 24 | 28 | 29 | 30 | 31 | 32 |
|  | San Marino | 4 | 2 | 10 | 9 | 6 | 8 | 7 | 12 | 11 | 1 | 3 | 13 | 5 | 14 | 20 | 15 | 19 | 23 | 16 | 22 | 25 | 18 | 24 | 17 | 30 | 27 | 21 | 31 | 28 | 29 | 26 | 32 |
|  | Spain | 1 | 3 | 10 | 8 | 6 | 5 | 9 | 15 | 11 | 2 | 4 | 12 | 7 | 14 | 17 | 21 | 22 | 23 | 13 | 20 | 16 | 19 | 24 | 18 | 28 | 26 | 25 | 31 | 27 | 30 | 29 | 32 |
|  | Sweden | 2 | 5 | 7 | 9 | 3 | 4 | 10 | 20 | 12 | 6 | 1 | 11 | 8 | 18 | 19 | 23 | 25 | 22 | 14 | 15 | 16 | 17 | 24 | 13 | 29 | 27 | 21 | 26 | 31 | 30 | 28 | 32 |
|  | Switzerland | 2 | 1 | 10 | 8 | 5 | 4 | 9 | 18 | 11 | 6 | 3 | 12 | 7 | 14 | 16 | 25 | 24 | 21 | 15 | 17 | 19 | 20 | 23 | 13 | 28 | 26 | 22 | 29 | 30 | 31 | 27 | 32 |
|  | United Kingdom | 1 | 4 | 5 | 8 | 9 | 3 | 10 | 20 | 13 | 6 | 2 | 11 | 7 | 15 | 14 | 30 | 22 | 21 | 12 | 18 | 16 | 19 | 23 | 17 | 26 | 25 | 24 | 31 | 28 | 29 | 27 | 32 |
| **Andean Latin America** | | **3** | **1** | **2** | **5** | **8** | **7** | **4** | **6** | **11** | **12** | **18** | **9** | **17** | **14** | **20** | **10** | **30** | **19** | **15** | **13** | **16** | **22** | **21** | **25** | **28** | **32** | **24** | **27** | **29** | **23** | **26** | **31** |
|  | Bolivia (Plurinational State of) | 3 | 2 | 1 | 8 | 5 | 12 | 6 | 4 | 10 | 11 | 18 | 9 | 16 | 14 | 20 | 7 | 30 | 19 | 17 | 13 | 15 | 23 | 22 | 24 | 27 | 32 | 25 | 31 | 29 | 21 | 26 | 28 |
|  | Ecuador | 3 | 2 | 1 | 5 | 10 | 7 | 4 | 6 | 9 | 8 | 18 | 11 | 17 | 16 | 19 | 13 | 29 | 21 | 15 | 12 | 14 | 22 | 20 | 23 | 27 | 32 | 25 | 30 | 28 | 24 | 26 | 31 |
|  | Peru | 3 | 1 | 2 | 4 | 8 | 7 | 5 | 6 | 11 | 12 | 19 | 9 | 17 | 13 | 21 | 10 | 30 | 18 | 14 | 15 | 16 | 22 | 20 | 25 | 28 | 32 | 24 | 26 | 29 | 23 | 27 | 31 |
| **Caribbean** | | **2** | **3** | **1** | **5** | **8** | **17** | **4** | **12** | **11** | **16** | **19** | **10** | **9** | **15** | **18** | **7** | **25** | **20** | **13** | **14** | **6** | **21** | **24** | **23** | **26** | **30** | **22** | **32** | **28** | **27** | **29** | **31** |
|  | Antigua and Barbuda | 1 | 3 | 2 | 5 | 8 | 12 | 4 | 14 | 7 | 18 | 15 | 6 | 19 | 17 | 20 | 10 | 25 | 23 | 11 | 13 | 9 | 22 | 21 | 24 | 27 | 29 | 16 | 31 | 28 | 30 | 26 | 32 |
|  | Bahamas | 1 | 3 | 2 | 5 | 8 | 30 | 4 | 12 | 10 | 19 | 15 | 6 | 14 | 13 | 16 | 18 | 26 | 20 | 9 | 11 | 7 | 25 | 21 | 24 | 23 | 28 | 17 | 31 | 27 | 29 | 22 | 32 |
|  | Barbados | 1 | 3 | 2 | 5 | 10 | 18 | 4 | 12 | 11 | 20 | 17 | 7 | 13 | 16 | 21 | 14 | 25 | 24 | 8 | 9 | 6 | 22 | 19 | 26 | 27 | 28 | 15 | 31 | 30 | 29 | 23 | 32 |
|  | Belize | 2 | 3 | 1 | 5 | 8 | 16 | 4 | 10 | 14 | 15 | 21 | 12 | 9 | 13 | 20 | 6 | 25 | 18 | 11 | 19 | 7 | 23 | 17 | 27 | 26 | 30 | 22 | 31 | 29 | 28 | 32 | 24 |
|  | Bermuda | 1 | 2 | 6 | 4 | 9 | 18 | 3 | 20 | 10 | 16 | 5 | 8 | 7 | 13 | 22 | 17 | 23 | 25 | 12 | 14 | 11 | 15 | 21 | 24 | 27 | 30 | 19 | 29 | 31 | 32 | 28 | 26 |
|  | Cuba | 2 | 3 | 1 | 7 | 8 | 10 | 4 | 18 | 9 | 14 | 16 | 11 | 6 | 15 | 17 | 20 | 24 | 26 | 12 | 13 | 5 | 19 | 22 | 21 | 27 | 30 | 23 | 28 | 25 | 31 | 29 | 32 |
|  | Dominica | 2 | 3 | 1 | 5 | 15 | 22 | 4 | 6 | 14 | 8 | 23 | 11 | 13 | 16 | 18 | 7 | 27 | 21 | 9 | 10 | 12 | 20 | 19 | 25 | 26 | 28 | 17 | 32 | 30 | 29 | 24 | 31 |
|  | Dominican Republic | 2 | 3 | 1 | 4 | 16 | 25 | 5 | 9 | 11 | 17 | 26 | 15 | 21 | 10 | 14 | 8 | 28 | 13 | 7 | 12 | 6 | 20 | 24 | 29 | 22 | 27 | 18 | 32 | 23 | 19 | 30 | 31 |
|  | Grenada | 2 | 4 | 1 | 7 | 8 | 21 | 3 | 13 | 10 | 19 | 20 | 6 | 15 | 14 | 18 | 9 | 26 | 23 | 11 | 12 | 5 | 22 | 17 | 25 | 24 | 28 | 16 | 31 | 32 | 29 | 27 | 30 |
|  | Guyana | 2 | 3 | 1 | 5 | 14 | 15 | 6 | 10 | 12 | 8 | 23 | 4 | 13 | 16 | 20 | 11 | 28 | 21 | 9 | 22 | 7 | 24 | 17 | 19 | 26 | 31 | 18 | 32 | 30 | 27 | 29 | 25 |
|  | Haiti | 2 | 3 | 1 | 7 | 8 | 25 | 6 | 5 | 14 | 13 | 20 | 10 | 12 | 15 | 18 | 4 | 21 | 17 | 16 | 11 | 9 | 22 | 24 | 19 | 23 | 31 | 27 | 32 | 30 | 26 | 28 | 29 |
|  | Jamaica | 1 | 4 | 2 | 5 | 11 | 20 | 3 | 13 | 8 | 16 | 21 | 7 | 15 | 9 | 18 | 6 | 17 | 19 | 10 | 12 | 14 | 23 | 24 | 22 | 27 | 29 | 25 | 32 | 30 | 28 | 26 | 31 |
|  | Puerto Rico | 1 | 2 | 3 | 4 | 9 | 6 | 5 | 18 | 12 | 13 | 14 | 10 | 7 | 17 | 20 | 16 | 26 | 23 | 11 | 15 | 8 | 19 | 22 | 24 | 28 | 29 | 21 | 30 | 27 | 31 | 25 | 32 |
|  | Saint Kitts and Nevis | 2 | 3 | 1 | 5 | 12 | 7 | 4 | 13 | 10 | 15 | 24 | 6 | 22 | 16 | 17 | 11 | 23 | 21 | 9 | 20 | 8 | 19 | 18 | 25 | 28 | 31 | 14 | 29 | 30 | 32 | 26 | 27 |
|  | Saint Lucia | 2 | 4 | 1 | 6 | 12 | 10 | 3 | 11 | 7 | 19 | 18 | 5 | 9 | 16 | 20 | 13 | 23 | 26 | 14 | 15 | 8 | 22 | 21 | 24 | 27 | 30 | 17 | 29 | 28 | 31 | 25 | 32 |
|  | Saint Vincent and the Grenadines | 2 | 4 | 1 | 6 | 11 | 20 | 3 | 10 | 8 | 18 | 17 | 7 | 12 | 16 | 15 | 9 | 25 | 22 | 13 | 14 | 5 | 23 | 21 | 24 | 29 | 28 | 19 | 27 | 26 | 31 | 30 | 32 |
|  | Suriname | 2 | 3 | 1 | 5 | 7 | 16 | 4 | 13 | 9 | 18 | 21 | 6 | 10 | 12 | 19 | 8 | 22 | 20 | 11 | 14 | 15 | 24 | 17 | 25 | 29 | 30 | 23 | 32 | 31 | 27 | 26 | 28 |
|  | Trinidad and Tobago | 1 | 2 | 3 | 5 | 11 | 15 | 4 | 18 | 9 | 16 | 23 | 6 | 13 | 10 | 19 | 14 | 25 | 21 | 7 | 8 | 12 | 17 | 20 | 24 | 26 | 30 | 22 | 32 | 29 | 27 | 28 | 31 |
|  | United States Virgin Islands | 1 | 3 | 2 | 4 | 7 | 29 | 5 | 15 | 13 | 24 | 12 | 6 | 18 | 11 | 19 | 14 | 21 | 23 | 8 | 9 | 10 | 20 | 16 | 32 | 27 | 28 | 17 | 25 | 31 | 30 | 22 | 26 |
| **Central Latin America** | | **2** | **3** | **1** | **5** | **10** | **4** | **6** | **8** | **11** | **7** | **17** | **9** | **13** | **16** | **20** | **14** | **27** | **22** | **15** | **12** | **18** | **23** | **19** | **24** | **26** | **32** | **21** | **30** | **29** | **25** | **28** | **31** |
|  | Colombia | 2 | 3 | 1 | 4 | 10 | 5 | 7 | 6 | 11 | 9 | 14 | 8 | 12 | 16 | 22 | 15 | 28 | 23 | 17 | 13 | 18 | 21 | 20 | 24 | 27 | 32 | 19 | 29 | 30 | 25 | 26 | 31 |
|  | Costa Rica | 3 | 2 | 1 | 4 | 11 | 6 | 5 | 8 | 10 | 9 | 14 | 12 | 7 | 20 | 21 | 18 | 17 | 19 | 15 | 13 | 16 | 24 | 22 | 25 | 28 | 29 | 23 | 31 | 30 | 26 | 27 | 32 |
|  | El Salvador | 2 | 3 | 1 | 5 | 7 | 13 | 8 | 6 | 14 | 11 | 21 | 9 | 10 | 12 | 18 | 4 | 27 | 23 | 15 | 16 | 17 | 22 | 19 | 28 | 25 | 30 | 20 | 31 | 26 | 24 | 29 | 32 |
|  | Guatemala | 5 | 3 | 1 | 6 | 9 | 8 | 10 | 2 | 15 | 7 | 23 | 11 | 16 | 12 | 21 | 4 | 27 | 17 | 14 | 13 | 18 | 24 | 20 | 28 | 22 | 30 | 19 | 32 | 26 | 25 | 29 | 31 |
|  | Honduras | 3 | 2 | 1 | 13 | 7 | 18 | 15 | 5 | 4 | 10 | 24 | 6 | 20 | 9 | 16 | 8 | 31 | 14 | 17 | 12 | 11 | 23 | 21 | 19 | 30 | 28 | 22 | 32 | 27 | 25 | 26 | 29 |
|  | Mexico | 2 | 3 | 1 | 5 | 10 | 4 | 7 | 9 | 13 | 6 | 17 | 8 | 12 | 16 | 20 | 15 | 28 | 23 | 14 | 11 | 18 | 21 | 19 | 24 | 26 | 32 | 22 | 31 | 29 | 25 | 27 | 30 |
|  | Nicaragua | 3 | 2 | 1 | 5 | 8 | 10 | 6 | 9 | 11 | 4 | 19 | 7 | 15 | 16 | 21 | 12 | 25 | 18 | 14 | 13 | 17 | 23 | 20 | 24 | 30 | 32 | 22 | 26 | 28 | 27 | 29 | 31 |
|  | Panama | 3 | 2 | 1 | 4 | 6 | 11 | 5 | 7 | 9 | 8 | 18 | 10 | 15 | 16 | 19 | 13 | 25 | 20 | 14 | 12 | 17 | 24 | 21 | 23 | 27 | 30 | 22 | 31 | 29 | 28 | 26 | 32 |
|  | Venezuela (Bolivarian Republic of) | 2 | 3 | 1 | 4 | 11 | 13 | 5 | 7 | 14 | 9 | 18 | 6 | 8 | 15 | 20 | 17 | 25 | 24 | 12 | 10 | 16 | 22 | 21 | 23 | 29 | 30 | 19 | 31 | 26 | 27 | 28 | 32 |
| **Tropical Latin America** | | **2** | **3** | **1** | **5** | **4** | **7** | **6** | **9** | **12** | **15** | **11** | **8** | **13** | **14** | **18** | **17** | **28** | **24** | **16** | **10** | **19** | **22** | **20** | **30** | **23** | **26** | **21** | **32** | **27** | **25** | **31** | **29** |
|  | Brazil | 2 | 3 | 1 | 5 | 4 | 7 | 6 | 9 | 12 | 16 | 11 | 8 | 13 | 14 | 18 | 17 | 28 | 24 | 15 | 10 | 19 | 22 | 20 | 30 | 23 | 26 | 21 | 32 | 27 | 25 | 31 | 29 |
|  | Paraguay | 2 | 3 | 1 | 5 | 8 | 4 | 6 | 13 | 10 | 12 | 17 | 7 | 14 | 15 | 19 | 11 | 30 | 24 | 16 | 9 | 18 | 22 | 20 | 23 | 25 | 27 | 21 | 32 | 26 | 28 | 29 | 31 |
| **North Africa and Middle East** | | **1** | **2** | **8** | **7** | **3** | **6** | **5** | **11** | **4** | **17** | **22** | **14** | **9** | **15** | **23** | **12** | **18** | **19** | **16** | **13** | **20** | **10** | **25** | **24** | **27** | **31** | **21** | **30** | **26** | **28** | **29** | **32** |
|  | Afghanistan | 3 | 1 | 7 | 12 | 6 | 25 | 5 | 2 | 11 | 10 | 22 | 16 | 13 | 15 | 19 | 4 | 24 | 8 | 18 | 9 | 23 | 21 | 27 | 14 | 17 | 31 | 29 | 30 | 20 | 26 | 28 | 32 |
|  | Algeria | 1 | 2 | 4 | 9 | 8 | 10 | 5 | 14 | 3 | 26 | 21 | 11 | 7 | 15 | 17 | 16 | 6 | 23 | 12 | 13 | 25 | 20 | 24 | 18 | 30 | 29 | 19 | 31 | 27 | 22 | 28 | 32 |
|  | Bahrain | 1 | 2 | 10 | 4 | 5 | 23 | 3 | 14 | 8 | 17 | 25 | 7 | 6 | 13 | 19 | 16 | 24 | 22 | 9 | 11 | 21 | 18 | 20 | 15 | 28 | 30 | 12 | 27 | 31 | 29 | 26 | 32 |
|  | Egypt | 2 | 3 | 15 | 7 | 4 | 16 | 5 | 18 | 6 | 13 | 28 | 12 | 17 | 9 | 24 | 11 | 27 | 8 | 14 | 10 | 19 | 1 | 20 | 21 | 25 | 31 | 23 | 29 | 22 | 26 | 30 | 32 |
|  | Iran (Islamic Republic of) | 1 | 2 | 13 | 6 | 3 | 4 | 7 | 8 | 5 | 12 | 19 | 15 | 9 | 11 | 23 | 16 | 28 | 21 | 14 | 10 | 20 | 17 | 25 | 22 | 26 | 31 | 18 | 27 | 24 | 30 | 29 | 32 |
|  | Iraq | 1 | 2 | 10 | 8 | 3 | 9 | 6 | 15 | 5 | 19 | 26 | 7 | 12 | 11 | 20 | 4 | 24 | 17 | 13 | 14 | 18 | 16 | 21 | 22 | 28 | 31 | 23 | 32 | 25 | 29 | 27 | 30 |
|  | Jordan | 1 | 2 | 10 | 8 | 6 | 5 | 3 | 13 | 7 | 24 | 20 | 9 | 14 | 11 | 16 | 4 | 18 | 23 | 12 | 21 | 17 | 15 | 22 | 29 | 26 | 30 | 19 | 31 | 27 | 25 | 28 | 32 |
|  | Kuwait | 1 | 2 | 8 | 6 | 3 | 11 | 5 | 19 | 4 | 15 | 20 | 7 | 12 | 18 | 21 | 10 | 22 | 24 | 13 | 17 | 16 | 14 | 23 | 25 | 26 | 31 | 9 | 30 | 29 | 28 | 27 | 32 |
|  | Lebanon | 1 | 2 | 9 | 7 | 6 | 5 | 8 | 19 | 4 | 12 | 13 | 10 | 3 | 14 | 23 | 16 | 22 | 24 | 17 | 15 | 20 | 11 | 26 | 18 | 31 | 30 | 21 | 28 | 25 | 29 | 27 | 32 |
|  | Libya | 1 | 2 | 7 | 8 | 4 | 14 | 9 | 16 | 3 | 18 | 26 | 10 | 5 | 12 | 21 | 13 | 6 | 19 | 17 | 11 | 22 | 15 | 20 | 23 | 30 | 31 | 24 | 29 | 25 | 27 | 28 | 32 |
|  | Morocco | 1 | 3 | 4 | 10 | 8 | 15 | 5 | 16 | 2 | 19 | 21 | 6 | 7 | 11 | 12 | 17 | 9 | 25 | 14 | 13 | 18 | 22 | 20 | 26 | 28 | 30 | 24 | 31 | 23 | 29 | 27 | 32 |
|  | Oman | 2 | 1 | 9 | 7 | 4 | 8 | 3 | 15 | 5 | 11 | 21 | 14 | 6 | 20 | 19 | 17 | 22 | 16 | 13 | 10 | 26 | 18 | 23 | 24 | 29 | 32 | 12 | 28 | 31 | 30 | 27 | 25 |
|  | Palestine | 1 | 3 | 12 | 5 | 2 | 20 | 6 | 11 | 7 | 18 | 25 | 10 | 8 | 9 | 21 | 4 | 23 | 16 | 14 | 15 | 13 | 17 | 19 | 24 | 28 | 31 | 22 | 27 | 30 | 29 | 26 | 32 |
|  | Qatar | 1 | 2 | 11 | 5 | 3 | 6 | 4 | 17 | 7 | 13 | 22 | 16 | 8 | 15 | 23 | 21 | 20 | 19 | 12 | 10 | 30 | 18 | 24 | 14 | 26 | 31 | 9 | 25 | 27 | 29 | 28 | 32 |
|  | Saudi Arabia | 2 | 1 | 9 | 6 | 5 | 8 | 4 | 21 | 3 | 14 | 25 | 11 | 7 | 15 | 19 | 20 | 12 | 24 | 10 | 16 | 23 | 17 | 22 | 18 | 26 | 30 | 13 | 31 | 29 | 27 | 28 | 32 |
|  | Sudan | 1 | 2 | 7 | 10 | 3 | 16 | 8 | 4 | 6 | 11 | 23 | 14 | 12 | 13 | 21 | 5 | 25 | 19 | 15 | 9 | 24 | 18 | 22 | 17 | 20 | 31 | 26 | 30 | 27 | 28 | 29 | 32 |
|  | Syrian Arab Republic | 1 | 3 | 10 | 6 | 4 | 14 | 5 | 12 | 15 | 9 | 25 | 11 | 21 | 8 | 24 | 2 | 23 | 16 | 18 | 7 | 13 | 17 | 22 | 20 | 29 | 30 | 19 | 26 | 27 | 31 | 28 | 32 |
|  | Tunisia | 1 | 2 | 6 | 7 | 8 | 11 | 9 | 16 | 3 | 17 | 19 | 10 | 5 | 12 | 18 | 22 | 4 | 25 | 14 | 13 | 21 | 15 | 24 | 26 | 31 | 30 | 20 | 28 | 23 | 27 | 29 | 32 |
|  | Turkey | 1 | 3 | 15 | 5 | 4 | 2 | 6 | 8 | 7 | 11 | 16 | 14 | 13 | 10 | 23 | 19 | 17 | 24 | 12 | 9 | 20 | 18 | 21 | 29 | 25 | 32 | 22 | 27 | 26 | 31 | 30 | 28 |
|  | United Arab Emirates | 2 | 1 | 15 | 9 | 4 | 7 | 3 | 21 | 5 | 24 | 23 | 13 | 12 | 18 | 17 | 20 | 25 | 19 | 6 | 14 | 27 | 8 | 10 | 28 | 16 | 30 | 11 | 31 | 22 | 29 | 26 | 32 |
|  | Yemen | 1 | 2 | 5 | 10 | 4 | 17 | 9 | 3 | 7 | 12 | 23 | 15 | 11 | 13 | 21 | 6 | 24 | 20 | 16 | 8 | 25 | 18 | 27 | 14 | 19 | 31 | 26 | 30 | 22 | 28 | 29 | 32 |
| **South Asia** | | **2** | **1** | **3** | **10** | **8** | **11** | **6** | **7** | **5** | **18** | **28** | **9** | **12** | **15** | **4** | **21** | **19** | **17** | **23** | **14** | **25** | **24** | **27** | **16** | **20** | **13** | **30** | **32** | **22** | **26** | **29** | **31** |
|  | Bangladesh | 2 | 1 | 3 | 13 | 7 | 11 | 6 | 9 | 4 | 17 | 26 | 8 | 10 | 15 | 5 | 21 | 19 | 14 | 22 | 12 | 28 | 23 | 25 | 18 | 20 | 16 | 29 | 32 | 24 | 27 | 30 | 31 |
|  | Bhutan | 3 | 1 | 2 | 10 | 7 | 11 | 6 | 9 | 4 | 17 | 28 | 8 | 12 | 14 | 5 | 21 | 20 | 18 | 22 | 13 | 24 | 25 | 23 | 16 | 19 | 15 | 30 | 32 | 27 | 26 | 29 | 31 |
|  | India | 2 | 3 | 1 | 9 | 8 | 12 | 6 | 7 | 5 | 18 | 28 | 10 | 14 | 13 | 4 | 21 | 19 | 17 | 22 | 15 | 27 | 26 | 24 | 16 | 20 | 11 | 30 | 32 | 23 | 25 | 29 | 31 |
|  | Nepal | 3 | 1 | 2 | 12 | 8 | 17 | 6 | 9 | 4 | 15 | 28 | 7 | 11 | 13 | 5 | 20 | 19 | 18 | 22 | 10 | 24 | 27 | 23 | 16 | 21 | 14 | 30 | 32 | 26 | 25 | 29 | 31 |
|  | Pakistan | 1 | 2 | 6 | 11 | 9 | 8 | 10 | 14 | 5 | 15 | 25 | 4 | 7 | 13 | 3 | 18 | 22 | 19 | 27 | 12 | 17 | 20 | 28 | 21 | 16 | 24 | 30 | 31 | 23 | 26 | 29 | 32 |
| **East Asia** | | **1** | **3** | **7** | **2** | **6** | **13** | **12** | **4** | **14** | **9** | **19** | **17** | **23** | **10** | **20** | **11** | **5** | **8** | **15** | **25** | **16** | **18** | **21** | **29** | **24** | **31** | **26** | **22** | **30** | **28** | **27** | **32** |
|  | China | 1 | 3 | 7 | 2 | 6 | 13 | 12 | 4 | 14 | 9 | 19 | 17 | 22 | 10 | 23 | 11 | 5 | 8 | 15 | 25 | 16 | 18 | 20 | 29 | 24 | 31 | 26 | 21 | 30 | 28 | 27 | 32 |
|  | Democratic People's Republic of Korea | 2 | 4 | 1 | 5 | 8 | 22 | 11 | 6 | 10 | 18 | 26 | 12 | 23 | 9 | 17 | 3 | 15 | 7 | 14 | 19 | 13 | 16 | 20 | 30 | 21 | 32 | 27 | 25 | 29 | 24 | 28 | 31 |
|  | Taiwan (Province of China) | 2 | 4 | 8 | 1 | 11 | 6 | 9 | 13 | 7 | 12 | 27 | 10 | 22 | 14 | 5 | 20 | 3 | 17 | 15 | 23 | 16 | 18 | 24 | 25 | 21 | 19 | 28 | 32 | 29 | 26 | 30 | 31 |
| **Oceania** | | **1** | **3** | **2** | **6** | **9** | **15** | **12** | **4** | **11** | **17** | **26** | **13** | **18** | **5** | **10** | **7** | **20** | **16** | **22** | **8** | **14** | **19** | **25** | **21** | **24** | **29** | **23** | **32** | **31** | **28** | **27** | **30** |
|  | American Samoa | 1 | 2 | 3 | 5 | 12 | 25 | 8 | 7 | 10 | 18 | 24 | 4 | 20 | 9 | 17 | 14 | 21 | 11 | 19 | 13 | 6 | 15 | 22 | 23 | 28 | 27 | 16 | 31 | 32 | 29 | 26 | 30 |
|  | Cook Islands | 1 | 2 | 3 | 7 | 11 | 17 | 4 | 10 | 6 | 22 | 24 | 13 | 19 | 5 | 14 | 20 | 25 | 8 | 18 | 15 | 16 | 9 | 21 | 26 | 23 | 28 | 12 | 32 | 30 | 29 | 27 | 31 |
|  | Fiji | 2 | 3 | 1 | 6 | 13 | 8 | 7 | 9 | 5 | 19 | 25 | 17 | 16 | 14 | 10 | 15 | 29 | 12 | 20 | 4 | 11 | 18 | 21 | 23 | 24 | 27 | 22 | 32 | 31 | 28 | 26 | 30 |
|  | Guam | 1 | 6 | 4 | 2 | 16 | 23 | 3 | 8 | 7 | 24 | 28 | 9 | 19 | 5 | 15 | 13 | 18 | 11 | 17 | 10 | 12 | 14 | 20 | 26 | 22 | 25 | 21 | 29 | 32 | 30 | 27 | 31 |
|  | Kiribati | 2 | 3 | 1 | 9 | 17 | 5 | 11 | 6 | 23 | 19 | 28 | 14 | 18 | 10 | 4 | 8 | 21 | 7 | 15 | 16 | 13 | 20 | 26 | 25 | 12 | 22 | 24 | 32 | 31 | 27 | 29 | 30 |
|  | Marshall Islands | 2 | 4 | 1 | 5 | 13 | 25 | 9 | 3 | 14 | 17 | 26 | 11 | 18 | 6 | 15 | 7 | 19 | 8 | 21 | 10 | 12 | 16 | 22 | 20 | 24 | 29 | 23 | 32 | 31 | 28 | 27 | 30 |
|  | Micronesia (Federated States of) | 1 | 3 | 2 | 5 | 14 | 25 | 7 | 4 | 13 | 17 | 26 | 10 | 18 | 6 | 15 | 8 | 23 | 11 | 19 | 9 | 12 | 16 | 22 | 20 | 24 | 29 | 21 | 32 | 31 | 28 | 27 | 30 |
|  | Nauru | 1 | 3 | 2 | 5 | 14 | 24 | 7 | 4 | 8 | 18 | 26 | 13 | 19 | 6 | 15 | 11 | 23 | 12 | 17 | 10 | 9 | 16 | 22 | 20 | 25 | 28 | 21 | 32 | 30 | 29 | 27 | 31 |
|  | Niue | 1 | 3 | 2 | 4 | 12 | 18 | 5 | 7 | 6 | 20 | 24 | 9 | 19 | 8 | 14 | 15 | 23 | 13 | 17 | 11 | 10 | 16 | 22 | 25 | 26 | 28 | 21 | 31 | 30 | 29 | 27 | 32 |
|  | Northern Mariana Islands | 1 | 3 | 2 | 4 | 11 | 24 | 5 | 9 | 10 | 20 | 26 | 14 | 21 | 6 | 7 | 13 | 18 | 15 | 17 | 12 | 8 | 16 | 23 | 27 | 25 | 22 | 19 | 29 | 31 | 30 | 28 | 32 |
|  | Palau | 3 | 2 | 1 | 8 | 11 | 29 | 10 | 6 | 5 | 20 | 18 | 15 | 22 | 7 | 4 | 19 | 25 | 9 | 17 | 16 | 23 | 13 | 14 | 26 | 21 | 24 | 12 | 31 | 32 | 28 | 27 | 30 |
|  | Papua New Guinea | 1 | 3 | 2 | 7 | 9 | 24 | 15 | 4 | 13 | 14 | 25 | 10 | 16 | 5 | 11 | 6 | 18 | 26 | 22 | 8 | 12 | 17 | 23 | 19 | 21 | 29 | 20 | 32 | 31 | 28 | 27 | 30 |
|  | Samoa | 3 | 2 | 1 | 9 | 13 | 6 | 8 | 10 | 4 | 19 | 17 | 7 | 5 | 20 | 18 | 12 | 16 | 14 | 22 | 11 | 15 | 21 | 24 | 23 | 26 | 29 | 25 | 31 | 32 | 27 | 28 | 30 |
|  | Solomon Islands | 1 | 4 | 2 | 8 | 12 | 26 | 5 | 3 | 10 | 17 | 25 | 14 | 19 | 7 | 15 | 6 | 20 | 13 | 22 | 11 | 9 | 16 | 24 | 18 | 21 | 29 | 23 | 32 | 30 | 28 | 27 | 31 |
|  | Tokelau | 1 | 3 | 2 | 4 | 14 | 20 | 5 | 6 | 8 | 17 | 25 | 12 | 19 | 9 | 15 | 13 | 22 | 11 | 18 | 10 | 7 | 16 | 23 | 24 | 26 | 28 | 21 | 31 | 32 | 29 | 27 | 30 |
|  | Tonga | 2 | 6 | 3 | 11 | 13 | 1 | 5 | 7 | 12 | 18 | 25 | 9 | 20 | 8 | 16 | 15 | 23 | 4 | 19 | 10 | 14 | 17 | 22 | 27 | 26 | 28 | 21 | 32 | 31 | 29 | 24 | 30 |
|  | Tuvalu | 1 | 3 | 2 | 5 | 14 | 22 | 7 | 4 | 11 | 17 | 26 | 12 | 18 | 6 | 15 | 8 | 21 | 10 | 19 | 9 | 13 | 16 | 24 | 23 | 25 | 28 | 20 | 32 | 30 | 29 | 27 | 31 |
|  | Vanuatu | 2 | 3 | 1 | 7 | 11 | 26 | 10 | 4 | 12 | 16 | 25 | 15 | 18 | 6 | 13 | 5 | 19 | 9 | 21 | 8 | 14 | 17 | 24 | 20 | 23 | 30 | 22 | 32 | 31 | 28 | 27 | 29 |
| **Southeast Asia** | | **1** | **2** | **3** | **4** | **10** | **15** | **7** | **11** | **5** | **18** | **26** | **6** | **20** | **9** | **12** | **8** | **16** | **14** | **17** | **13** | **19** | **22** | **21** | **24** | **23** | **28** | **27** | **32** | **29** | **25** | **31** | **30** |
|  | Cambodia | 1 | 3 | 2 | 5 | 8 | 20 | 10 | 12 | 9 | 16 | 25 | 6 | 18 | 11 | 13 | 4 | 17 | 7 | 19 | 14 | 15 | 22 | 21 | 24 | 23 | 30 | 29 | 32 | 27 | 28 | 31 | 26 |
|  | Indonesia | 1 | 2 | 3 | 4 | 9 | 18 | 8 | 12 | 10 | 16 | 25 | 5 | 20 | 7 | 13 | 6 | 19 | 21 | 15 | 11 | 14 | 22 | 17 | 24 | 23 | 30 | 26 | 32 | 29 | 28 | 31 | 27 |
|  | Lao People's Democratic Republic | 1 | 3 | 2 | 5 | 7 | 21 | 9 | 11 | 10 | 15 | 25 | 6 | 19 | 8 | 14 | 4 | 17 | 12 | 18 | 13 | 16 | 22 | 20 | 24 | 23 | 30 | 29 | 32 | 28 | 26 | 31 | 27 |
|  | Malaysia | 1 | 2 | 3 | 5 | 10 | 8 | 7 | 16 | 4 | 12 | 24 | 9 | 14 | 15 | 13 | 17 | 6 | 18 | 19 | 11 | 20 | 21 | 23 | 22 | 27 | 28 | 25 | 32 | 26 | 29 | 31 | 30 |
|  | Maldives | 1 | 2 | 7 | 8 | 5 | 19 | 4 | 17 | 9 | 18 | 23 | 3 | 6 | 14 | 11 | 15 | 25 | 13 | 12 | 10 | 20 | 16 | 21 | 27 | 26 | 30 | 22 | 32 | 28 | 31 | 29 | 24 |
|  | Mauritius | 1 | 2 | 5 | 4 | 8 | 16 | 6 | 15 | 10 | 18 | 27 | 3 | 11 | 13 | 9 | 7 | 21 | 23 | 12 | 14 | 17 | 20 | 19 | 30 | 22 | 29 | 24 | 31 | 25 | 28 | 26 | 32 |
|  | Myanmar | 1 | 4 | 2 | 6 | 9 | 20 | 3 | 11 | 10 | 15 | 25 | 7 | 19 | 8 | 13 | 5 | 18 | 17 | 16 | 12 | 14 | 22 | 21 | 24 | 23 | 30 | 29 | 32 | 28 | 27 | 31 | 26 |
|  | Philippines | 1 | 3 | 2 | 4 | 10 | 16 | 8 | 17 | 6 | 19 | 25 | 5 | 22 | 9 | 15 | 7 | 18 | 11 | 14 | 12 | 13 | 21 | 20 | 23 | 24 | 30 | 26 | 32 | 28 | 27 | 31 | 29 |
|  | Seychelles | 1 | 6 | 2 | 3 | 7 | 8 | 4 | 14 | 22 | 12 | 27 | 5 | 10 | 11 | 9 | 13 | 20 | 23 | 21 | 15 | 16 | 19 | 24 | 18 | 28 | 29 | 17 | 26 | 25 | 32 | 30 | 31 |
|  | Sri Lanka | 1 | 2 | 4 | 10 | 7 | 14 | 3 | 17 | 5 | 12 | 28 | 6 | 8 | 16 | 11 | 9 | 19 | 21 | 13 | 15 | 18 | 22 | 23 | 24 | 20 | 26 | 25 | 32 | 29 | 27 | 31 | 30 |
|  | Thailand | 1 | 3 | 2 | 5 | 8 | 11 | 10 | 13 | 12 | 25 | 29 | 9 | 17 | 7 | 14 | 6 | 15 | 4 | 16 | 18 | 19 | 20 | 22 | 24 | 23 | 30 | 26 | 32 | 27 | 21 | 31 | 28 |
|  | Timor-Leste | 3 | 1 | 2 | 5 | 7 | 20 | 8 | 12 | 10 | 14 | 25 | 6 | 17 | 9 | 15 | 4 | 18 | 13 | 19 | 11 | 16 | 22 | 21 | 24 | 23 | 30 | 27 | 32 | 29 | 28 | 31 | 26 |
|  | Viet Nam | 1 | 3 | 4 | 5 | 13 | 7 | 12 | 10 | 2 | 15 | 24 | 8 | 14 | 9 | 6 | 18 | 11 | 23 | 17 | 16 | 20 | 21 | 22 | 26 | 28 | 19 | 27 | 32 | 25 | 29 | 31 | 30 |
| **Central Sub-Saharan Africa** | | **2** | **3** | **1** | **7** | **8** | **17** | **4** | **6** | **18** | **16** | **21** | **10** | **11** | **9** | **14** | **5** | **25** | **13** | **22** | **12** | **24** | **20** | **23** | **19** | **15** | **31** | **26** | **32** | **27** | **30** | **28** | **29** |
|  | Angola | 2 | 3 | 1 | 5 | 8 | 14 | 4 | 7 | 18 | 17 | 21 | 10 | 12 | 9 | 13 | 6 | 25 | 16 | 20 | 11 | 24 | 22 | 23 | 19 | 15 | 31 | 26 | 32 | 27 | 30 | 28 | 29 |
|  | Central African Republic | 2 | 3 | 1 | 8 | 7 | 19 | 6 | 4 | 18 | 15 | 20 | 12 | 10 | 9 | 14 | 5 | 21 | 13 | 24 | 16 | 25 | 22 | 23 | 17 | 11 | 31 | 27 | 32 | 26 | 28 | 29 | 30 |
|  | Congo | 2 | 3 | 1 | 5 | 9 | 17 | 4 | 8 | 15 | 20 | 24 | 6 | 16 | 10 | 11 | 7 | 25 | 14 | 19 | 12 | 23 | 21 | 18 | 22 | 13 | 31 | 26 | 32 | 28 | 30 | 27 | 29 |
|  | Democratic Republic of the Congo | 2 | 3 | 1 | 8 | 7 | 17 | 5 | 6 | 18 | 16 | 21 | 10 | 11 | 9 | 15 | 4 | 25 | 12 | 22 | 14 | 24 | 19 | 23 | 20 | 13 | 31 | 26 | 32 | 28 | 30 | 27 | 29 |
|  | Equatorial Guinea | 2 | 3 | 1 | 5 | 7 | 10 | 4 | 12 | 16 | 19 | 22 | 6 | 17 | 8 | 13 | 9 | 26 | 14 | 15 | 11 | 24 | 21 | 20 | 23 | 18 | 29 | 25 | 32 | 28 | 31 | 27 | 30 |
|  | Gabon | 2 | 3 | 1 | 5 | 7 | 11 | 4 | 10 | 15 | 21 | 23 | 6 | 17 | 8 | 13 | 9 | 26 | 16 | 14 | 12 | 24 | 20 | 19 | 22 | 18 | 31 | 25 | 32 | 28 | 29 | 27 | 30 |
| **Eastern Sub-Saharan Africa** | | **3** | **2** | **1** | **7** | **10** | **20** | **8** | **9** | **4** | **15** | **16** | **5** | **6** | **21** | **11** | **12** | **19** | **18** | **22** | **17** | **23** | **25** | **24** | **14** | **13** | **28** | **31** | **32** | **27** | **29** | **26** | **30** |
|  | Burundi | 3 | 2 | 1 | 7 | 9 | 20 | 8 | 5 | 4 | 18 | 14 | 10 | 6 | 19 | 12 | 11 | 15 | 16 | 23 | 21 | 22 | 24 | 25 | 17 | 13 | 28 | 31 | 32 | 26 | 29 | 27 | 30 |
|  | Comoros | 3 | 2 | 1 | 6 | 10 | 16 | 8 | 9 | 5 | 20 | 14 | 4 | 7 | 17 | 11 | 13 | 18 | 15 | 24 | 19 | 23 | 25 | 22 | 21 | 12 | 28 | 31 | 32 | 27 | 29 | 26 | 30 |
|  | Djibouti | 3 | 2 | 1 | 5 | 10 | 13 | 7 | 8 | 4 | 21 | 16 | 6 | 9 | 15 | 11 | 14 | 19 | 17 | 22 | 18 | 24 | 25 | 23 | 20 | 12 | 28 | 29 | 32 | 27 | 30 | 26 | 31 |
|  | Eritrea | 3 | 2 | 1 | 5 | 10 | 18 | 8 | 4 | 6 | 20 | 14 | 9 | 7 | 15 | 12 | 11 | 16 | 17 | 25 | 21 | 23 | 24 | 22 | 19 | 13 | 28 | 31 | 32 | 26 | 29 | 27 | 30 |
|  | Ethiopia | 4 | 2 | 1 | 9 | 7 | 14 | 15 | 10 | 3 | 8 | 19 | 11 | 6 | 20 | 13 | 16 | 17 | 18 | 21 | 12 | 24 | 23 | 25 | 5 | 22 | 31 | 30 | 32 | 27 | 26 | 28 | 29 |
|  | Kenya | 3 | 2 | 1 | 9 | 7 | 24 | 4 | 8 | 11 | 17 | 18 | 6 | 10 | 19 | 5 | 14 | 13 | 16 | 21 | 15 | 23 | 25 | 20 | 22 | 12 | 27 | 30 | 32 | 28 | 29 | 26 | 31 |
|  | Madagascar | 3 | 2 | 1 | 7 | 10 | 19 | 9 | 6 | 4 | 21 | 14 | 5 | 8 | 16 | 13 | 11 | 17 | 15 | 24 | 18 | 22 | 25 | 23 | 20 | 12 | 28 | 31 | 32 | 26 | 29 | 27 | 30 |
|  | Malawi | 3 | 1 | 2 | 11 | 10 | 15 | 5 | 20 | 4 | 18 | 7 | 8 | 9 | 21 | 13 | 17 | 25 | 19 | 16 | 12 | 24 | 14 | 22 | 23 | 6 | 32 | 26 | 31 | 29 | 30 | 28 | 27 |
|  | Mozambique | 3 | 2 | 1 | 8 | 10 | 17 | 7 | 13 | 4 | 18 | 12 | 6 | 5 | 15 | 22 | 9 | 31 | 11 | 19 | 16 | 21 | 23 | 24 | 14 | 20 | 29 | 30 | 32 | 25 | 27 | 26 | 28 |
|  | Rwanda | 3 | 2 | 1 | 6 | 10 | 16 | 7 | 9 | 4 | 21 | 15 | 5 | 8 | 18 | 11 | 13 | 17 | 12 | 22 | 19 | 24 | 25 | 23 | 20 | 14 | 28 | 31 | 32 | 27 | 29 | 26 | 30 |
|  | Somalia | 3 | 2 | 1 | 11 | 8 | 21 | 10 | 4 | 7 | 16 | 17 | 12 | 5 | 19 | 13 | 6 | 15 | 14 | 25 | 20 | 22 | 23 | 24 | 18 | 9 | 29 | 31 | 32 | 26 | 28 | 27 | 30 |
|  | South Sudan | 3 | 2 | 1 | 4 | 10 | 21 | 5 | 9 | 6 | 18 | 17 | 8 | 7 | 16 | 13 | 11 | 19 | 15 | 23 | 14 | 24 | 25 | 22 | 20 | 12 | 27 | 31 | 32 | 28 | 29 | 26 | 30 |
|  | Uganda | 3 | 2 | 1 | 6 | 12 | 17 | 8 | 9 | 4 | 24 | 14 | 5 | 7 | 18 | 11 | 16 | 15 | 10 | 23 | 21 | 20 | 25 | 19 | 22 | 13 | 26 | 29 | 32 | 28 | 30 | 27 | 31 |
|  | United Republic of Tanzania | 3 | 2 | 1 | 6 | 9 | 15 | 7 | 10 | 4 | 20 | 14 | 5 | 8 | 17 | 11 | 12 | 18 | 21 | 22 | 16 | 24 | 25 | 23 | 19 | 13 | 28 | 31 | 32 | 27 | 29 | 26 | 30 |
|  | Zambia | 3 | 2 | 1 | 5 | 10 | 16 | 7 | 9 | 4 | 20 | 14 | 6 | 8 | 15 | 11 | 13 | 18 | 21 | 22 | 17 | 24 | 25 | 23 | 19 | 12 | 28 | 31 | 32 | 27 | 29 | 26 | 30 |
| **Southern Sub-Saharan Africa** | | **2** | **3** | **1** | **6** | **9** | **17** | **4** | **11** | **12** | **22** | **15** | **7** | **16** | **10** | **14** | **8** | **26** | **5** | **20** | **19** | **23** | **18** | **21** | **31** | **13** | **29** | **25** | **27** | **28** | **30** | **24** | **32** |
|  | Botswana | 2 | 3 | 1 | 4 | 7 | 30 | 5 | 10 | 22 | 18 | 16 | 6 | 23 | 9 | 11 | 8 | 26 | 20 | 15 | 14 | 19 | 13 | 17 | 28 | 12 | 29 | 21 | 25 | 27 | 31 | 24 | 32 |
|  | Eswatini | 3 | 4 | 1 | 6 | 9 | 22 | 5 | 11 | 16 | 20 | 19 | 7 | 15 | 10 | 13 | 8 | 24 | 2 | 17 | 14 | 23 | 21 | 18 | 28 | 12 | 29 | 26 | 30 | 27 | 32 | 25 | 31 |
|  | Lesotho | 2 | 4 | 1 | 7 | 11 | 22 | 5 | 9 | 16 | 18 | 17 | 8 | 14 | 10 | 12 | 6 | 23 | 3 | 20 | 15 | 24 | 21 | 19 | 26 | 13 | 30 | 28 | 32 | 25 | 31 | 27 | 29 |
|  | Namibia | 2 | 3 | 1 | 10 | 7 | 8 | 4 | 18 | 9 | 19 | 6 | 11 | 12 | 16 | 5 | 13 | 24 | 14 | 17 | 15 | 20 | 23 | 22 | 31 | 26 | 25 | 27 | 30 | 21 | 32 | 28 | 29 |
|  | South Africa | 2 | 4 | 1 | 6 | 9 | 13 | 3 | 11 | 17 | 22 | 15 | 8 | 14 | 10 | 16 | 7 | 27 | 5 | 18 | 20 | 23 | 19 | 21 | 32 | 12 | 29 | 24 | 26 | 28 | 30 | 25 | 31 |
|  | Zimbabwe | 2 | 3 | 1 | 7 | 14 | 22 | 17 | 6 | 8 | 21 | 11 | 5 | 16 | 9 | 15 | 18 | 24 | 4 | 25 | 12 | 20 | 10 | 19 | 28 | 13 | 31 | 29 | 27 | 26 | 30 | 23 | 32 |
| **Western Sub-Saharan Africa** | | **2** | **3** | **1** | **7** | **5** | **24** | **4** | **8** | **19** | **14** | **21** | **9** | **6** | **13** | **17** | **10** | **22** | **11** | **20** | **12** | **26** | **15** | **18** | **16** | **23** | **31** | **25** | **32** | **27** | **29** | **28** | **30** |
|  | Benin | 2 | 3 | 1 | 8 | 7 | 23 | 4 | 5 | 19 | 13 | 22 | 11 | 14 | 12 | 18 | 6 | 26 | 9 | 20 | 10 | 25 | 16 | 17 | 15 | 21 | 31 | 24 | 32 | 27 | 28 | 29 | 30 |
|  | Burkina Faso | 2 | 3 | 1 | 8 | 7 | 24 | 4 | 5 | 17 | 13 | 22 | 9 | 15 | 11 | 16 | 6 | 25 | 12 | 21 | 10 | 23 | 18 | 19 | 14 | 20 | 31 | 26 | 32 | 27 | 28 | 29 | 30 |
|  | Cabo Verde | 2 | 3 | 1 | 8 | 6 | 22 | 5 | 7 | 15 | 18 | 24 | 12 | 25 | 9 | 19 | 10 | 26 | 4 | 16 | 13 | 23 | 11 | 14 | 20 | 17 | 27 | 21 | 30 | 28 | 31 | 29 | 32 |
|  | Cameroon | 2 | 4 | 1 | 5 | 8 | 27 | 3 | 6 | 18 | 15 | 24 | 10 | 17 | 11 | 14 | 7 | 16 | 25 | 21 | 9 | 22 | 12 | 13 | 19 | 20 | 31 | 23 | 32 | 26 | 29 | 28 | 30 |
|  | Chad | 3 | 2 | 1 | 9 | 7 | 23 | 5 | 4 | 18 | 12 | 22 | 13 | 14 | 11 | 17 | 6 | 24 | 8 | 21 | 10 | 26 | 16 | 20 | 15 | 19 | 31 | 25 | 32 | 27 | 28 | 29 | 30 |
|  | Côte d'Ivoire | 2 | 3 | 1 | 6 | 7 | 23 | 4 | 5 | 19 | 15 | 22 | 11 | 18 | 10 | 17 | 8 | 25 | 9 | 20 | 12 | 26 | 13 | 16 | 14 | 21 | 30 | 24 | 32 | 27 | 28 | 29 | 31 |
|  | Gambia | 4 | 3 | 2 | 6 | 9 | 14 | 11 | 15 | 13 | 16 | 21 | 5 | 10 | 17 | 12 | 8 | 25 | 1 | 19 | 7 | 23 | 22 | 20 | 18 | 24 | 32 | 26 | 30 | 31 | 27 | 28 | 29 |
|  | Ghana | 2 | 5 | 1 | 6 | 3 | 18 | 4 | 12 | 23 | 16 | 22 | 7 | 21 | 11 | 20 | 14 | 27 | 8 | 15 | 9 | 25 | 10 | 13 | 17 | 24 | 31 | 19 | 32 | 26 | 29 | 30 | 28 |
|  | Guinea | 4 | 3 | 1 | 9 | 14 | 16 | 17 | 5 | 10 | 18 | 15 | 8 | 7 | 11 | 6 | 12 | 24 | 2 | 22 | 13 | 21 | 19 | 23 | 20 | 25 | 29 | 26 | 32 | 27 | 28 | 31 | 30 |
|  | Guinea-Bissau | 2 | 3 | 1 | 6 | 8 | 25 | 5 | 4 | 19 | 14 | 22 | 10 | 15 | 11 | 17 | 7 | 24 | 9 | 21 | 12 | 23 | 16 | 18 | 13 | 20 | 31 | 26 | 32 | 27 | 28 | 30 | 29 |
|  | Liberia | 2 | 3 | 1 | 10 | 6 | 23 | 4 | 5 | 18 | 13 | 22 | 9 | 15 | 12 | 19 | 8 | 26 | 7 | 20 | 11 | 24 | 14 | 17 | 16 | 21 | 31 | 25 | 32 | 27 | 29 | 28 | 30 |
|  | Mali | 2 | 5 | 1 | 6 | 10 | 20 | 13 | 3 | 8 | 16 | 18 | 12 | 14 | 17 | 15 | 9 | 27 | 4 | 22 | 11 | 24 | 7 | 21 | 19 | 23 | 32 | 26 | 30 | 25 | 28 | 31 | 29 |
|  | Mauritania | 2 | 3 | 1 | 6 | 7 | 21 | 4 | 8 | 16 | 14 | 22 | 5 | 17 | 12 | 20 | 11 | 26 | 9 | 18 | 10 | 25 | 13 | 15 | 19 | 23 | 31 | 24 | 32 | 28 | 29 | 27 | 30 |
|  | Niger | 3 | 2 | 1 | 8 | 6 | 24 | 10 | 5 | 25 | 12 | 21 | 11 | 13 | 9 | 15 | 4 | 26 | 20 | 19 | 7 | 23 | 14 | 18 | 16 | 17 | 31 | 22 | 32 | 27 | 28 | 29 | 30 |
|  | Nigeria | 2 | 3 | 1 | 7 | 6 | 25 | 5 | 14 | 20 | 13 | 16 | 8 | 4 | 11 | 19 | 9 | 15 | 12 | 18 | 10 | 26 | 23 | 21 | 17 | 28 | 32 | 22 | 30 | 24 | 29 | 27 | 31 |
|  | Sao Tome and Principe | 3 | 2 | 1 | 5 | 16 | 19 | 6 | 9 | 12 | 14 | 24 | 4 | 26 | 7 | 21 | 13 | 29 | 11 | 15 | 10 | 18 | 8 | 20 | 17 | 22 | 32 | 23 | 31 | 27 | 25 | 28 | 30 |
|  | Senegal | 2 | 3 | 1 | 7 | 8 | 22 | 4 | 6 | 19 | 12 | 23 | 10 | 14 | 11 | 17 | 5 | 26 | 15 | 20 | 9 | 25 | 13 | 18 | 16 | 21 | 31 | 24 | 32 | 27 | 28 | 29 | 30 |
|  | Sierra Leone | 2 | 3 | 1 | 8 | 6 | 23 | 4 | 5 | 18 | 13 | 22 | 10 | 15 | 12 | 17 | 7 | 26 | 9 | 20 | 11 | 24 | 16 | 19 | 14 | 21 | 31 | 25 | 32 | 27 | 28 | 29 | 30 |
|  | Togo | 2 | 3 | 1 | 7 | 6 | 23 | 4 | 5 | 18 | 13 | 22 | 10 | 15 | 11 | 17 | 9 | 26 | 8 | 20 | 12 | 24 | 14 | 19 | 16 | 21 | 31 | 25 | 32 | 27 | 28 | 29 | 30 |

Colour intensity and number ranking are assigned according to the rank of absolute number of incident cases of each cancer type among all cancer types. Dark red and number ranking of 1 indicate the highest rank and greatest absolute number of incident cases. Dark green and number ranking of 32 indicate the lowest rank and the smallest absolute number of incident cases.

## eTable 9. AYA cancer ranking by the number of deaths at the global level and according to SDI quintile, super-regions, regions, and countries, both sexes, 2019

| **Location** | | **Other malignant neoplasms** | **Breast cancer** | **Brain and central nervous system cancer** | **Colon and rectum cancer** | **Stomach cancer** | **Cervical cancer** | **Tracheal, bronchus, and lung cancer** | **Non-Hodgkin lymphoma** | **Liver cancer** | **Other leukemia** | **Acute myeloid leukemia** | **Acute lymphoid leukemia** | **Lip and oral cavity cancer** | **Ovarian cancer** | **Hodgkin lymphoma** | **Pancreatic cancer** | **Esophageal cancer** | **Nasopharynx cancer** | **Testicular cancer** | **Chronic myeloid leukemia** | **Other pharynx cancer** | **Malignant skin melanoma** | **Kidney cancer** | **Thyroid cancer** | **Gallbladder and biliary tract cancer** | **Larynx cancer** | **Bladder cancer** | **Uterine cancer** | **Multiple myeloma** | **Chronic lymphoid leukemia** | **Mesothelioma** | **Prostate cancer** |
| --- | --- | --- | --- | --- | --- | --- | --- | --- | --- | --- | --- | --- | --- | --- | --- | --- | --- | --- | --- | --- | --- | --- | --- | --- | --- | --- | --- | --- | --- | --- | --- | --- | --- |
| **Global** | | **1** | **2** | **3** | **4** | **5** | **6** | **7** | **8** | **9** | **10** | **11** | **12** | **13** | **14** | **15** | **16** | **17** | **18** | **19** | **20** | **21** | **22** | **23** | **24** | **25** | **26** | **27** | **28** | **29** | **30** | **31** | **32** |
|  | **Low SDI** | **1** | **3** | **5** | **7** | **4** | **2** | **12** | **6** | **11** | **10** | **13** | **15** | **9** | **16** | **8** | **23** | **17** | **18** | **20** | **14** | **21** | **22** | **27** | **19** | **26** | **24** | **25** | **28** | **29** | **32** | **31** | **30** |
|  | **Low-middle SDI** | **1** | **2** | **5** | **6** | **4** | **3** | **8** | **7** | **11** | **10** | **12** | **15** | **9** | **16** | **13** | **21** | **18** | **19** | **20** | **17** | **14** | **26** | **25** | **22** | **24** | **23** | **27** | **28** | **29** | **32** | **30** | **31** |
|  | **Middle SDI** | **1** | **2** | **5** | **3** | **6** | **8** | **4** | **9** | **7** | **10** | **12** | **11** | **15** | **13** | **18** | **14** | **17** | **16** | **19** | **21** | **22** | **25** | **20** | **24** | **26** | **28** | **23** | **27** | **29** | **30** | **31** | **32** |
|  | **High-middle SDI** | **1** | **4** | **3** | **2** | **6** | **9** | **5** | **7** | **8** | **10** | **11** | **12** | **20** | **14** | **17** | **13** | **21** | **15** | **18** | **23** | **29** | **16** | **19** | **28** | **22** | **27** | **26** | **24** | **25** | **30** | **31** | **32** |
|  | **High SDI** | **1** | **2** | **3** | **4** | **8** | **9** | **5** | **6** | **11** | **15** | **7** | **12** | **19** | **14** | **17** | **13** | **20** | **22** | **18** | **21** | **28** | **10** | **16** | **27** | **23** | **29** | **25** | **26** | **24** | **32** | **30** | **31** |
| **Central Asia** | | **2** | **3** | **1** | **6** | **4** | **7** | **5** | **8** | **11** | **9** | **10** | **12** | **18** | **14** | **13** | **16** | **15** | **21** | **19** | **24** | **25** | **23** | **17** | **30** | **31** | **22** | **27** | **20** | **28** | **32** | **29** | **26** |
|  | Armenia | 3 | 2 | 1 | 5 | 6 | 7 | 4 | 9 | 13 | 8 | 11 | 10 | 21 | 14 | 17 | 12 | 29 | 24 | 15 | 30 | 32 | 19 | 18 | 28 | 31 | 22 | 23 | 20 | 27 | 26 | 16 | 25 |
|  | Azerbaijan | 1 | 4 | 3 | 6 | 5 | 8 | 2 | 10 | 17 | 7 | 9 | 13 | 21 | 15 | 11 | 14 | 12 | 26 | 18 | 25 | 29 | 24 | 16 | 27 | 28 | 19 | 23 | 22 | 30 | 31 | 32 | 20 |
|  | Georgia | 3 | 1 | 4 | 6 | 5 | 8 | 2 | 9 | 15 | 7 | 12 | 16 | 21 | 13 | 10 | 14 | 24 | 30 | 11 | 23 | 27 | 18 | 19 | 28 | 29 | 20 | 25 | 17 | 26 | 32 | 31 | 22 |
|  | Kazakhstan | 1 | 4 | 3 | 6 | 5 | 2 | 7 | 10 | 12 | 20 | 8 | 11 | 17 | 9 | 13 | 14 | 18 | 24 | 19 | 21 | 27 | 16 | 15 | 25 | 32 | 28 | 26 | 22 | 29 | 31 | 30 | 23 |
|  | Kyrgyzstan | 1 | 5 | 3 | 6 | 2 | 4 | 8 | 11 | 14 | 9 | 7 | 10 | 19 | 12 | 18 | 13 | 17 | 20 | 16 | 24 | 23 | 22 | 15 | 25 | 28 | 26 | 27 | 21 | 30 | 31 | 32 | 29 |
|  | Mongolia | 3 | 7 | 4 | 6 | 2 | 5 | 9 | 10 | 1 | 20 | 8 | 15 | 17 | 12 | 19 | 13 | 11 | 25 | 30 | 21 | 28 | 27 | 16 | 26 | 18 | 32 | 24 | 14 | 23 | 29 | 31 | 22 |
|  | Tajikistan | 3 | 4 | 1 | 5 | 2 | 11 | 6 | 7 | 13 | 8 | 9 | 10 | 20 | 16 | 18 | 17 | 12 | 24 | 32 | 23 | 26 | 22 | 14 | 31 | 30 | 21 | 25 | 15 | 28 | 29 | 19 | 27 |
|  | Turkmenistan | 1 | 3 | 2 | 7 | 4 | 5 | 6 | 12 | 9 | 10 | 11 | 14 | 19 | 15 | 8 | 20 | 13 | 23 | 18 | 25 | 26 | 24 | 17 | 28 | 30 | 27 | 29 | 31 | 16 | 32 | 22 | 21 |
|  | Uzbekistan | 2 | 3 | 1 | 8 | 4 | 7 | 6 | 5 | 11 | 9 | 10 | 12 | 14 | 16 | 15 | 17 | 13 | 19 | 20 | 24 | 23 | 25 | 18 | 32 | 30 | 22 | 26 | 21 | 27 | 28 | 29 | 31 |
| **Central Europe** | | **2** | **3** | **1** | **4** | **8** | **6** | **5** | **7** | **18** | **16** | **9** | **14** | **19** | **13** | **15** | **12** | **20** | **24** | **11** | **25** | **21** | **10** | **17** | **29** | **26** | **23** | **27** | **22** | **28** | **30** | **31** | **32** |
|  | Albania | 2 | 5 | 1 | 6 | 4 | 13 | 3 | 9 | 10 | 12 | 7 | 8 | 17 | 16 | 15 | 14 | 23 | 27 | 11 | 25 | 30 | 18 | 19 | 24 | 26 | 20 | 29 | 22 | 31 | 28 | 32 | 21 |
|  | Bosnia and Herzegovina | 5 | 3 | 1 | 2 | 6 | 8 | 4 | 7 | 14 | 18 | 9 | 11 | 19 | 10 | 16 | 13 | 23 | 30 | 15 | 21 | 22 | 12 | 17 | 27 | 24 | 25 | 26 | 20 | 28 | 31 | 29 | 32 |
|  | Bulgaria | 6 | 3 | 1 | 5 | 8 | 4 | 2 | 7 | 16 | 13 | 9 | 17 | 20 | 12 | 14 | 11 | 23 | 27 | 10 | 25 | 22 | 15 | 18 | 26 | 29 | 21 | 24 | 19 | 28 | 32 | 31 | 30 |
|  | Croatia | 2 | 3 | 1 | 4 | 8 | 11 | 5 | 6 | 17 | 19 | 9 | 14 | 18 | 12 | 16 | 13 | 21 | 27 | 10 | 22 | 20 | 7 | 15 | 29 | 25 | 23 | 26 | 28 | 24 | 32 | 30 | 31 |
|  | Czechia | 2 | 3 | 1 | 4 | 11 | 6 | 5 | 8 | 17 | 18 | 7 | 16 | 19 | 13 | 14 | 12 | 20 | 28 | 10 | 21 | 24 | 9 | 15 | 29 | 22 | 26 | 27 | 23 | 25 | 32 | 30 | 31 |
|  | Hungary | 4 | 2 | 1 | 5 | 11 | 6 | 3 | 9 | 19 | 18 | 7 | 14 | 15 | 13 | 17 | 12 | 21 | 24 | 8 | 23 | 20 | 10 | 16 | 29 | 25 | 22 | 27 | 26 | 28 | 31 | 30 | 32 |
|  | Montenegro | 5 | 3 | 1 | 4 | 13 | 6 | 2 | 8 | 14 | 23 | 7 | 11 | 19 | 16 | 9 | 12 | 22 | 31 | 10 | 20 | 26 | 15 | 18 | 25 | 27 | 17 | 24 | 21 | 28 | 29 | 32 | 30 |
|  | North Macedonia | 4 | 3 | 1 | 5 | 6 | 8 | 2 | 11 | 13 | 7 | 15 | 17 | 20 | 14 | 16 | 12 | 26 | 27 | 10 | 24 | 23 | 9 | 18 | 31 | 25 | 21 | 22 | 19 | 28 | 30 | 32 | 29 |
|  | Poland | 2 | 3 | 1 | 4 | 7 | 10 | 5 | 6 | 20 | 19 | 11 | 14 | 17 | 12 | 15 | 13 | 18 | 27 | 9 | 21 | 23 | 8 | 16 | 29 | 22 | 24 | 25 | 28 | 26 | 31 | 30 | 32 |
|  | Romania | 2 | 6 | 1 | 4 | 8 | 3 | 5 | 7 | 19 | 15 | 9 | 14 | 18 | 11 | 16 | 10 | 23 | 20 | 12 | 27 | 21 | 13 | 17 | 29 | 28 | 22 | 25 | 24 | 26 | 30 | 31 | 32 |
|  | Serbia | 3 | 2 | 1 | 5 | 9 | 6 | 4 | 7 | 17 | 15 | 11 | 16 | 19 | 12 | 14 | 13 | 20 | 25 | 10 | 22 | 23 | 8 | 18 | 30 | 28 | 26 | 27 | 21 | 29 | 24 | 31 | 32 |
|  | Slovakia | 2 | 4 | 1 | 3 | 9 | 5 | 6 | 7 | 18 | 20 | 8 | 14 | 16 | 12 | 15 | 13 | 22 | 26 | 10 | 27 | 19 | 11 | 17 | 29 | 23 | 24 | 28 | 21 | 25 | 32 | 30 | 31 |
|  | Slovenia | 3 | 2 | 1 | 4 | 9 | 10 | 5 | 7 | 14 | 21 | 8 | 15 | 18 | 13 | 16 | 11 | 22 | 30 | 12 | 20 | 19 | 6 | 17 | 28 | 24 | 27 | 25 | 29 | 23 | 32 | 26 | 31 |
| **Eastern Europe** | | **2** | **3** | **1** | **5** | **4** | **6** | **7** | **8** | **17** | **19** | **14** | **13** | **15** | **12** | **11** | **10** | **20** | **28** | **16** | **21** | **23** | **9** | **18** | **26** | **30** | **24** | **27** | **22** | **29** | **32** | **25** | **31** |
|  | Belarus | 1 | 4 | 3 | 6 | 2 | 5 | 7 | 8 | 20 | 22 | 9 | 12 | 19 | 13 | 11 | 14 | 21 | 29 | 15 | 16 | 25 | 10 | 17 | 27 | 30 | 23 | 31 | 28 | 26 | 32 | 18 | 24 |
|  | Estonia | 2 | 3 | 1 | 5 | 4 | 9 | 7 | 6 | 18 | 14 | 8 | 11 | 19 | 12 | 15 | 13 | 21 | 26 | 16 | 20 | 23 | 10 | 17 | 24 | 25 | 28 | 29 | 27 | 22 | 32 | 30 | 31 |
|  | Latvia | 2 | 3 | 1 | 5 | 4 | 8 | 7 | 6 | 17 | 16 | 11 | 14 | 19 | 12 | 13 | 9 | 20 | 27 | 15 | 21 | 22 | 10 | 18 | 29 | 28 | 23 | 26 | 24 | 25 | 32 | 30 | 31 |
|  | Lithuania | 2 | 3 | 1 | 5 | 4 | 9 | 7 | 6 | 17 | 20 | 8 | 13 | 18 | 10 | 14 | 12 | 19 | 26 | 15 | 21 | 22 | 11 | 16 | 28 | 29 | 23 | 27 | 24 | 25 | 32 | 30 | 31 |
|  | Republic of Moldova | 2 | 4 | 1 | 3 | 7 | 5 | 6 | 8 | 20 | 11 | 13 | 16 | 18 | 12 | 10 | 9 | 24 | 19 | 15 | 30 | 21 | 14 | 17 | 27 | 29 | 22 | 25 | 23 | 26 | 32 | 31 | 28 |
|  | Russian Federation | 2 | 3 | 1 | 6 | 5 | 4 | 7 | 8 | 15 | 19 | 14 | 12 | 16 | 11 | 13 | 10 | 20 | 28 | 17 | 22 | 24 | 9 | 18 | 25 | 30 | 23 | 26 | 21 | 27 | 31 | 29 | 32 |
|  | Ukraine | 1 | 4 | 2 | 5 | 3 | 11 | 6 | 7 | 19 | 17 | 12 | 13 | 16 | 14 | 8 | 10 | 22 | 26 | 15 | 20 | 23 | 9 | 18 | 29 | 31 | 25 | 28 | 24 | 30 | 32 | 21 | 27 |
| **Australasia** | | **2** | **3** | **1** | **4** | **11** | **9** | **7** | **8** | **12** | **20** | **6** | **10** | **15** | **14** | **16** | **13** | **19** | **22** | **18** | **21** | **29** | **5** | **17** | **24** | **27** | **31** | **28** | **25** | **23** | **32** | **26** | **30** |
|  | Australia | 2 | 3 | 1 | 4 | 11 | 9 | 7 | 8 | 13 | 20 | 6 | 10 | 15 | 14 | 17 | 12 | 19 | 22 | 18 | 21 | 29 | 5 | 16 | 24 | 27 | 31 | 28 | 26 | 23 | 32 | 25 | 30 |
|  | New Zealand | 2 | 1 | 3 | 5 | 9 | 11 | 8 | 7 | 10 | 21 | 6 | 12 | 18 | 14 | 16 | 15 | 20 | 23 | 13 | 22 | 28 | 4 | 17 | 19 | 27 | 31 | 26 | 25 | 24 | 32 | 29 | 30 |
| **High-income Asia Pacific** | | **2** | **3** | **5** | **4** | **1** | **10** | **6** | **9** | **7** | **14** | **8** | **11** | **15** | **12** | **24** | **13** | **23** | **21** | **18** | **20** | **30** | **19** | **16** | **25** | **17** | **31** | **26** | **22** | **27** | **32** | **28** | **29** |
|  | Brunei Darussalam | 2 | 1 | 6 | 3 | 7 | 8 | 5 | 4 | 9 | 14 | 10 | 15 | 16 | 12 | 18 | 17 | 26 | 13 | 20 | 11 | 25 | 21 | 19 | 27 | 23 | 32 | 28 | 22 | 24 | 29 | 31 | 30 |
|  | Japan | 1 | 3 | 5 | 2 | 4 | 8 | 6 | 9 | 12 | 15 | 7 | 11 | 14 | 10 | 23 | 13 | 22 | 24 | 17 | 21 | 29 | 20 | 16 | 28 | 18 | 31 | 25 | 19 | 26 | 32 | 27 | 30 |
|  | Republic of Korea | 3 | 4 | 6 | 5 | 1 | 10 | 7 | 9 | 2 | 14 | 8 | 11 | 17 | 13 | 23 | 12 | 22 | 21 | 27 | 18 | 30 | 20 | 16 | 19 | 15 | 32 | 26 | 25 | 24 | 31 | 29 | 28 |
|  | Singapore | 1 | 2 | 5 | 3 | 11 | 13 | 4 | 7 | 9 | 18 | 6 | 10 | 16 | 12 | 20 | 14 | 23 | 8 | 24 | 17 | 29 | 19 | 15 | 26 | 22 | 31 | 27 | 21 | 25 | 32 | 30 | 28 |
| **High-income North America** | | **1** | **2** | **3** | **4** | **11** | **7** | **5** | **6** | **14** | **16** | **8** | **10** | **20** | **13** | **15** | **12** | **19** | **25** | **18** | **21** | **28** | **9** | **17** | **26** | **27** | **31** | **24** | **22** | **23** | **32** | **29** | **30** |
|  | Canada | 1 | 3 | 2 | 4 | 10 | 9 | 6 | 5 | 11 | 16 | 8 | 12 | 19 | 14 | 15 | 13 | 20 | 24 | 17 | 21 | 29 | 7 | 18 | 26 | 27 | 31 | 23 | 25 | 22 | 32 | 28 | 30 |
|  | Greenland | 4 | 6 | 5 | 2 | 7 | 3 | 1 | 9 | 12 | 25 | 16 | 18 | 20 | 11 | 19 | 10 | 13 | 8 | 14 | 23 | 21 | 17 | 15 | 28 | 24 | 30 | 29 | 31 | 26 | 22 | 27 | 32 |
|  | United States of America | 1 | 2 | 3 | 4 | 11 | 7 | 5 | 6 | 14 | 16 | 8 | 10 | 20 | 13 | 15 | 12 | 19 | 25 | 18 | 21 | 28 | 9 | 17 | 26 | 27 | 29 | 24 | 22 | 23 | 32 | 31 | 30 |
| **Southern Latin America** | | **2** | **3** | **5** | **4** | **9** | **1** | **8** | **6** | **21** | **12** | **10** | **11** | **20** | **13** | **17** | **14** | **19** | **29** | **7** | **22** | **31** | **16** | **15** | **23** | **18** | **28** | **26** | **25** | **24** | **32** | **27** | **30** |
|  | Argentina | 3 | 2 | 5 | 4 | 9 | 1 | 8 | 6 | 21 | 12 | 10 | 11 | 20 | 13 | 17 | 14 | 18 | 29 | 7 | 22 | 31 | 16 | 15 | 24 | 19 | 27 | 26 | 23 | 25 | 32 | 28 | 30 |
|  | Chile | 1 | 4 | 7 | 5 | 6 | 2 | 11 | 8 | 19 | 12 | 10 | 9 | 21 | 13 | 18 | 16 | 23 | 28 | 3 | 20 | 31 | 17 | 14 | 24 | 15 | 30 | 25 | 29 | 22 | 32 | 26 | 27 |
|  | Uruguay | 4 | 1 | 6 | 5 | 8 | 2 | 7 | 3 | 20 | 12 | 10 | 14 | 18 | 15 | 17 | 13 | 21 | 24 | 9 | 19 | 30 | 16 | 11 | 27 | 22 | 25 | 26 | 28 | 23 | 32 | 29 | 31 |
| **Western Europe** | | **3** | **2** | **1** | **5** | **9** | **10** | **4** | **6** | **15** | **17** | **7** | **11** | **20** | **13** | **14** | **12** | **19** | **24** | **16** | **22** | **25** | **8** | **18** | **28** | **27** | **30** | **21** | **26** | **23** | **32** | **29** | **31** |
|  | Andorra | 2 | 4 | 1 | 6 | 9 | 8 | 3 | 5 | 10 | 18 | 7 | 12 | 20 | 16 | 14 | 13 | 19 | 25 | 17 | 21 | 24 | 11 | 15 | 27 | 22 | 30 | 28 | 26 | 23 | 32 | 29 | 31 |
|  | Austria | 2 | 3 | 1 | 7 | 10 | 9 | 4 | 8 | 15 | 18 | 5 | 11 | 19 | 14 | 16 | 12 | 20 | 29 | 13 | 21 | 22 | 6 | 17 | 30 | 25 | 27 | 26 | 23 | 24 | 32 | 28 | 31 |
|  | Belgium | 3 | 1 | 2 | 5 | 11 | 9 | 4 | 6 | 14 | 10 | 7 | 15 | 19 | 13 | 16 | 12 | 18 | 27 | 20 | 25 | 23 | 8 | 17 | 28 | 30 | 24 | 22 | 26 | 21 | 32 | 29 | 31 |
|  | Cyprus | 1 | 3 | 2 | 4 | 8 | 13 | 5 | 6 | 16 | 12 | 7 | 11 | 19 | 10 | 14 | 15 | 20 | 24 | 18 | 23 | 28 | 9 | 17 | 26 | 25 | 29 | 22 | 30 | 21 | 32 | 31 | 27 |
|  | Denmark | 2 | 3 | 1 | 4 | 13 | 9 | 5 | 8 | 16 | 18 | 6 | 11 | 20 | 10 | 15 | 12 | 19 | 29 | 14 | 21 | 24 | 7 | 17 | 25 | 26 | 30 | 22 | 28 | 23 | 32 | 27 | 31 |
|  | Finland | 2 | 3 | 1 | 5 | 9 | 16 | 7 | 4 | 13 | 19 | 6 | 10 | 18 | 11 | 14 | 12 | 21 | 30 | 15 | 22 | 27 | 8 | 17 | 23 | 24 | 31 | 26 | 25 | 20 | 32 | 28 | 29 |
|  | France | 2 | 1 | 3 | 5 | 10 | 9 | 4 | 6 | 12 | 11 | 8 | 13 | 19 | 15 | 17 | 14 | 20 | 25 | 16 | 22 | 23 | 7 | 18 | 29 | 30 | 27 | 21 | 26 | 24 | 32 | 28 | 31 |
|  | Germany | 2 | 3 | 1 | 4 | 8 | 10 | 5 | 7 | 15 | 19 | 6 | 12 | 18 | 14 | 16 | 11 | 20 | 31 | 13 | 21 | 23 | 9 | 17 | 26 | 22 | 29 | 25 | 27 | 24 | 28 | 30 | 32 |
|  | Greece | 3 | 2 | 1 | 5 | 6 | 12 | 4 | 8 | 18 | 15 | 7 | 13 | 19 | 14 | 9 | 11 | 21 | 20 | 16 | 24 | 32 | 10 | 17 | 30 | 26 | 27 | 23 | 22 | 25 | 29 | 28 | 31 |
|  | Iceland | 2 | 3 | 1 | 8 | 9 | 13 | 4 | 6 | 15 | 17 | 5 | 14 | 19 | 11 | 16 | 10 | 18 | 28 | 24 | 22 | 29 | 7 | 12 | 20 | 27 | 31 | 23 | 25 | 21 | 32 | 26 | 30 |
|  | Ireland | 3 | 2 | 1 | 4 | 11 | 8 | 7 | 5 | 17 | 20 | 9 | 13 | 19 | 10 | 14 | 12 | 16 | 27 | 18 | 23 | 29 | 6 | 15 | 24 | 28 | 31 | 22 | 26 | 21 | 32 | 25 | 30 |
|  | Israel | 3 | 1 | 2 | 5 | 9 | 12 | 7 | 4 | 16 | 15 | 6 | 10 | 19 | 14 | 11 | 13 | 21 | 25 | 18 | 20 | 32 | 8 | 17 | 23 | 30 | 29 | 24 | 27 | 22 | 26 | 28 | 31 |
|  | Italy | 1 | 2 | 3 | 6 | 9 | 14 | 5 | 4 | 18 | 16 | 7 | 10 | 19 | 13 | 11 | 12 | 22 | 20 | 15 | 21 | 29 | 8 | 17 | 27 | 24 | 30 | 26 | 25 | 23 | 32 | 28 | 31 |
|  | Luxembourg | 3 | 2 | 1 | 5 | 12 | 13 | 4 | 6 | 16 | 9 | 7 | 14 | 18 | 10 | 17 | 11 | 19 | 22 | 15 | 27 | 21 | 8 | 20 | 26 | 30 | 28 | 24 | 23 | 25 | 32 | 29 | 31 |
|  | Malta | 1 | 2 | 3 | 7 | 11 | 15 | 5 | 4 | 18 | 20 | 6 | 9 | 19 | 12 | 10 | 13 | 21 | 17 | 14 | 22 | 29 | 8 | 16 | 24 | 30 | 27 | 23 | 26 | 25 | 32 | 28 | 31 |
|  | Monaco | 5 | 3 | 4 | 7 | 15 | 16 | 2 | 1 | 14 | 6 | 8 | 17 | 24 | 12 | 10 | 13 | 20 | 26 | 11 | 23 | 30 | 9 | 18 | 25 | 29 | 22 | 21 | 31 | 19 | 32 | 28 | 27 |
|  | Netherlands | 2 | 1 | 3 | 6 | 10 | 9 | 4 | 7 | 15 | 14 | 8 | 12 | 20 | 11 | 16 | 13 | 18 | 26 | 17 | 24 | 29 | 5 | 19 | 27 | 22 | 31 | 21 | 28 | 23 | 32 | 25 | 30 |
|  | Norway | 2 | 3 | 1 | 4 | 13 | 9 | 6 | 7 | 12 | 19 | 8 | 14 | 20 | 10 | 16 | 11 | 21 | 30 | 15 | 25 | 28 | 5 | 17 | 23 | 26 | 31 | 22 | 27 | 18 | 32 | 24 | 29 |
|  | Portugal | 3 | 2 | 1 | 4 | 6 | 9 | 5 | 7 | 12 | 14 | 8 | 10 | 18 | 15 | 16 | 13 | 20 | 22 | 19 | 26 | 21 | 11 | 17 | 29 | 28 | 23 | 27 | 25 | 24 | 32 | 31 | 30 |
|  | San Marino | 1 | 3 | 2 | 8 | 6 | 12 | 5 | 4 | 16 | 10 | 13 | 7 | 18 | 15 | 11 | 14 | 24 | 20 | 19 | 25 | 29 | 9 | 17 | 23 | 26 | 27 | 21 | 31 | 22 | 32 | 30 | 28 |
|  | Spain | 2 | 1 | 3 | 5 | 7 | 12 | 4 | 6 | 15 | 13 | 8 | 10 | 19 | 14 | 16 | 11 | 20 | 22 | 18 | 24 | 25 | 9 | 17 | 28 | 30 | 27 | 21 | 26 | 23 | 32 | 29 | 31 |
|  | Sweden | 2 | 3 | 1 | 4 | 14 | 9 | 7 | 8 | 12 | 18 | 6 | 13 | 19 | 10 | 15 | 11 | 21 | 28 | 17 | 23 | 30 | 5 | 16 | 25 | 20 | 32 | 24 | 27 | 22 | 31 | 26 | 29 |
|  | Switzerland | 1 | 3 | 2 | 5 | 9 | 11 | 4 | 8 | 13 | 18 | 6 | 12 | 19 | 15 | 16 | 10 | 20 | 27 | 14 | 22 | 25 | 7 | 17 | 24 | 26 | 31 | 23 | 30 | 21 | 32 | 28 | 29 |
|  | United Kingdom | 3 | 1 | 2 | 4 | 11 | 8 | 5 | 6 | 13 | 20 | 9 | 14 | 18 | 10 | 15 | 12 | 16 | 24 | 19 | 23 | 25 | 7 | 17 | 28 | 29 | 30 | 21 | 26 | 22 | 32 | 27 | 31 |
| **Andean Latin America** | | **2** | **6** | **4** | **9** | **1** | **3** | **10** | **5** | **14** | **8** | **11** | **7** | **23** | **13** | **17** | **15** | **24** | **31** | **12** | **19** | **32** | **20** | **16** | **22** | **18** | **28** | **26** | **21** | **25** | **29** | **30** | **27** |
|  | Bolivia (Plurinational State of) | 3 | 5 | 4 | 9 | 1 | 2 | 10 | 7 | 14 | 6 | 11 | 8 | 23 | 13 | 15 | 16 | 24 | 30 | 12 | 19 | 31 | 17 | 20 | 21 | 18 | 27 | 26 | 22 | 25 | 32 | 28 | 29 |
|  | Ecuador | 2 | 7 | 6 | 8 | 1 | 4 | 11 | 5 | 16 | 10 | 9 | 3 | 23 | 13 | 14 | 15 | 24 | 30 | 12 | 18 | 32 | 19 | 17 | 21 | 20 | 28 | 26 | 22 | 25 | 31 | 29 | 27 |
|  | Peru | 3 | 7 | 2 | 8 | 1 | 4 | 9 | 5 | 14 | 6 | 11 | 10 | 22 | 12 | 18 | 15 | 24 | 31 | 13 | 19 | 32 | 20 | 16 | 23 | 17 | 29 | 28 | 21 | 25 | 27 | 30 | 26 |
| **Caribbean** | | **3** | **2** | **8** | **5** | **6** | **1** | **9** | **4** | **14** | **7** | **10** | **11** | **19** | **12** | **13** | **17** | **20** | **23** | **22** | **18** | **28** | **21** | **16** | **25** | **24** | **26** | **29** | **15** | **27** | **32** | **31** | **30** |
|  | Antigua and Barbuda | 2 | 1 | 6 | 5 | 7 | 4 | 11 | 3 | 15 | 9 | 8 | 13 | 21 | 10 | 22 | 14 | 23 | 26 | 18 | 19 | 29 | 16 | 12 | 25 | 28 | 30 | 27 | 17 | 24 | 32 | 31 | 20 |
|  | Bahamas | 2 | 1 | 6 | 4 | 7 | 3 | 9 | 5 | 14 | 12 | 8 | 13 | 20 | 10 | 17 | 16 | 19 | 24 | 32 | 21 | 25 | 18 | 11 | 28 | 26 | 27 | 30 | 15 | 22 | 31 | 29 | 23 |
|  | Barbados | 3 | 1 | 7 | 5 | 8 | 4 | 11 | 2 | 17 | 9 | 6 | 15 | 21 | 10 | 16 | 13 | 19 | 24 | 25 | 20 | 27 | 22 | 12 | 26 | 28 | 31 | 29 | 14 | 18 | 32 | 30 | 23 |
|  | Belize | 2 | 4 | 8 | 6 | 5 | 1 | 9 | 7 | 14 | 3 | 17 | 11 | 21 | 15 | 10 | 13 | 22 | 24 | 18 | 23 | 30 | 19 | 12 | 29 | 26 | 27 | 28 | 16 | 31 | 32 | 20 | 25 |
|  | Bermuda | 2 | 1 | 5 | 4 | 14 | 9 | 6 | 3 | 19 | 12 | 7 | 16 | 21 | 8 | 15 | 10 | 18 | 26 | 29 | 22 | 28 | 11 | 13 | 25 | 31 | 30 | 24 | 17 | 23 | 32 | 20 | 27 |
|  | Cuba | 1 | 5 | 2 | 6 | 13 | 3 | 7 | 4 | 22 | 17 | 8 | 10 | 19 | 11 | 9 | 14 | 21 | 24 | 18 | 16 | 28 | 20 | 15 | 25 | 29 | 23 | 27 | 12 | 26 | 32 | 30 | 31 |
|  | Dominica | 2 | 3 | 11 | 6 | 5 | 4 | 9 | 1 | 16 | 8 | 10 | 7 | 18 | 15 | 12 | 13 | 21 | 24 | 25 | 19 | 26 | 23 | 14 | 28 | 27 | 30 | 29 | 17 | 20 | 32 | 31 | 22 |
|  | Dominican Republic | 2 | 3 | 13 | 4 | 7 | 1 | 6 | 5 | 9 | 8 | 10 | 12 | 15 | 18 | 23 | 19 | 17 | 27 | 29 | 25 | 21 | 28 | 11 | 22 | 16 | 20 | 30 | 14 | 26 | 32 | 31 | 24 |
|  | Grenada | 4 | 2 | 6 | 5 | 8 | 3 | 10 | 1 | 17 | 7 | 11 | 14 | 20 | 9 | 16 | 13 | 18 | 24 | 26 | 19 | 28 | 21 | 15 | 23 | 27 | 32 | 30 | 12 | 25 | 31 | 29 | 22 |
|  | Guyana | 3 | 2 | 10 | 4 | 7 | 1 | 11 | 5 | 16 | 9 | 18 | 6 | 20 | 8 | 12 | 15 | 25 | 27 | 19 | 17 | 31 | 21 | 14 | 23 | 26 | 30 | 28 | 13 | 29 | 32 | 24 | 22 |
|  | Haiti | 3 | 2 | 7 | 8 | 4 | 1 | 9 | 6 | 15 | 5 | 10 | 11 | 19 | 14 | 12 | 22 | 21 | 20 | 25 | 16 | 31 | 17 | 18 | 23 | 24 | 29 | 28 | 13 | 26 | 32 | 27 | 30 |
|  | Jamaica | 4 | 1 | 9 | 5 | 8 | 2 | 7 | 3 | 13 | 6 | 11 | 12 | 21 | 10 | 18 | 16 | 19 | 15 | 26 | 17 | 27 | 25 | 14 | 22 | 24 | 29 | 28 | 20 | 23 | 32 | 30 | 31 |
|  | Puerto Rico | 2 | 1 | 6 | 3 | 13 | 5 | 8 | 4 | 17 | 10 | 7 | 11 | 21 | 12 | 9 | 16 | 23 | 27 | 14 | 22 | 28 | 18 | 15 | 25 | 30 | 26 | 24 | 19 | 20 | 32 | 31 | 29 |
|  | Saint Kitts and Nevis | 2 | 1 | 7 | 5 | 8 | 3 | 11 | 4 | 15 | 6 | 16 | 13 | 20 | 9 | 26 | 14 | 19 | 21 | 10 | 17 | 32 | 27 | 12 | 24 | 29 | 31 | 28 | 18 | 25 | 30 | 23 | 22 |
|  | Saint Lucia | 4 | 3 | 7 | 6 | 5 | 2 | 10 | 1 | 21 | 9 | 8 | 13 | 18 | 11 | 12 | 14 | 22 | 24 | 15 | 19 | 29 | 20 | 16 | 26 | 30 | 28 | 27 | 17 | 23 | 32 | 31 | 25 |
|  | Saint Vincent and the Grenadines | 4 | 2 | 8 | 5 | 7 | 1 | 11 | 3 | 17 | 6 | 9 | 14 | 18 | 10 | 12 | 15 | 25 | 22 | 23 | 19 | 27 | 20 | 16 | 21 | 30 | 26 | 28 | 13 | 29 | 31 | 32 | 24 |
|  | Suriname | 2 | 3 | 5 | 6 | 9 | 1 | 8 | 4 | 16 | 7 | 11 | 15 | 22 | 10 | 12 | 13 | 27 | 18 | 17 | 20 | 30 | 21 | 14 | 23 | 25 | 31 | 29 | 19 | 24 | 32 | 28 | 26 |
|  | Trinidad and Tobago | 2 | 1 | 8 | 5 | 13 | 4 | 7 | 3 | 16 | 11 | 6 | 12 | 19 | 9 | 14 | 15 | 21 | 23 | 17 | 18 | 29 | 28 | 10 | 24 | 25 | 30 | 22 | 20 | 26 | 32 | 31 | 27 |
|  | United States Virgin Islands | 4 | 1 | 5 | 2 | 10 | 6 | 7 | 3 | 15 | 12 | 8 | 18 | 20 | 9 | 21 | 11 | 19 | 23 | 32 | 25 | 27 | 14 | 13 | 29 | 26 | 30 | 31 | 16 | 17 | 28 | 22 | 24 |
| **Central Latin America** | | **1** | **5** | **7** | **6** | **3** | **2** | **12** | **8** | **16** | **11** | **9** | **4** | **20** | **13** | **14** | **15** | **21** | **28** | **10** | **19** | **31** | **18** | **17** | **23** | **22** | **27** | **26** | **25** | **24** | **32** | **30** | **29** |
|  | Colombia | 1 | 5 | 6 | 7 | 2 | 3 | 10 | 8 | 17 | 11 | 9 | 4 | 22 | 12 | 15 | 14 | 21 | 30 | 13 | 19 | 32 | 16 | 18 | 23 | 20 | 27 | 26 | 25 | 24 | 31 | 29 | 28 |
|  | Costa Rica | 1 | 7 | 8 | 3 | 2 | 5 | 13 | 4 | 12 | 15 | 9 | 6 | 21 | 14 | 10 | 16 | 24 | 19 | 11 | 20 | 28 | 18 | 17 | 22 | 23 | 29 | 27 | 26 | 25 | 32 | 31 | 30 |
|  | El Salvador | 4 | 7 | 5 | 6 | 3 | 1 | 9 | 8 | 17 | 2 | 12 | 10 | 18 | 11 | 13 | 14 | 20 | 26 | 15 | 23 | 30 | 25 | 16 | 24 | 19 | 21 | 28 | 22 | 27 | 31 | 32 | 29 |
|  | Guatemala | 4 | 8 | 7 | 6 | 1 | 2 | 9 | 10 | 13 | 3 | 12 | 5 | 19 | 14 | 17 | 16 | 18 | 27 | 11 | 26 | 30 | 23 | 15 | 22 | 20 | 25 | 29 | 24 | 28 | 32 | 31 | 21 |
|  | Honduras | 1 | 7 | 6 | 11 | 3 | 2 | 4 | 12 | 8 | 9 | 10 | 5 | 17 | 13 | 21 | 18 | 26 | 31 | 19 | 16 | 27 | 25 | 20 | 14 | 22 | 24 | 29 | 15 | 23 | 32 | 28 | 30 |
|  | Mexico | 1 | 4 | 8 | 6 | 5 | 3 | 11 | 9 | 17 | 13 | 10 | 2 | 21 | 12 | 14 | 15 | 20 | 29 | 7 | 19 | 32 | 18 | 16 | 23 | 22 | 28 | 26 | 25 | 24 | 31 | 27 | 30 |
|  | Nicaragua | 3 | 6 | 5 | 7 | 4 | 1 | 11 | 8 | 14 | 9 | 10 | 2 | 20 | 12 | 17 | 15 | 26 | 21 | 13 | 18 | 32 | 22 | 16 | 23 | 19 | 29 | 27 | 25 | 28 | 24 | 30 | 31 |
|  | Panama | 1 | 4 | 5 | 7 | 3 | 2 | 11 | 8 | 14 | 10 | 9 | 6 | 19 | 12 | 18 | 15 | 22 | 24 | 13 | 17 | 28 | 20 | 16 | 21 | 25 | 27 | 29 | 26 | 23 | 31 | 32 | 30 |
|  | Venezuela (Bolivarian Republic of) | 3 | 2 | 10 | 5 | 4 | 1 | 9 | 6 | 18 | 12 | 8 | 7 | 19 | 11 | 13 | 15 | 22 | 26 | 16 | 17 | 30 | 20 | 14 | 27 | 23 | 25 | 28 | 21 | 24 | 32 | 31 | 29 |
| **Tropical Latin America** | | **1** | **2** | **4** | **5** | **6** | **3** | **9** | **7** | **19** | **12** | **8** | **10** | **20** | **11** | **16** | **14** | **17** | **26** | **13** | **23** | **22** | **15** | **18** | **28** | **21** | **24** | **30** | **29** | **27** | **32** | **25** | **31** |
|  | Brazil | 1 | 2 | 3 | 5 | 6 | 4 | 9 | 7 | 19 | 12 | 8 | 10 | 20 | 11 | 16 | 14 | 17 | 26 | 13 | 23 | 22 | 15 | 18 | 28 | 21 | 25 | 30 | 29 | 27 | 32 | 24 | 31 |
|  | Paraguay | 2 | 3 | 6 | 4 | 7 | 1 | 10 | 5 | 20 | 11 | 9 | 8 | 18 | 13 | 14 | 15 | 21 | 29 | 12 | 19 | 24 | 17 | 16 | 22 | 25 | 26 | 30 | 23 | 27 | 32 | 31 | 28 |
| **North Africa and Middle East** | | **1** | **2** | **3** | **6** | **5** | **13** | **7** | **4** | **11** | **9** | **8** | **10** | **23** | **15** | **12** | **14** | **18** | **20** | **21** | **16** | **31** | **26** | **19** | **24** | **25** | **22** | **17** | **28** | **27** | **29** | **32** | **30** |
|  | Afghanistan | 1 | 6 | 5 | 10 | 2 | 11 | 12 | 3 | 7 | 4 | 8 | 9 | 22 | 16 | 13 | 23 | 15 | 18 | 28 | 14 | 30 | 20 | 25 | 19 | 21 | 17 | 24 | 27 | 26 | 29 | 32 | 31 |
|  | Algeria | 1 | 2 | 4 | 6 | 11 | 5 | 9 | 3 | 16 | 13 | 10 | 21 | 20 | 14 | 7 | 15 | 27 | 8 | 24 | 12 | 26 | 23 | 19 | 18 | 17 | 22 | 28 | 30 | 25 | 32 | 31 | 29 |
|  | Bahrain | 2 | 1 | 5 | 4 | 8 | 16 | 7 | 3 | 13 | 12 | 6 | 14 | 18 | 11 | 15 | 10 | 21 | 19 | 32 | 9 | 28 | 24 | 17 | 26 | 25 | 29 | 22 | 27 | 20 | 30 | 31 | 23 |
|  | Egypt | 1 | 2 | 4 | 5 | 12 | 17 | 7 | 6 | 3 | 11 | 9 | 10 | 23 | 15 | 16 | 13 | 20 | 27 | 24 | 14 | 31 | 29 | 18 | 21 | 22 | 19 | 8 | 25 | 26 | 28 | 32 | 30 |
|  | Iran (Islamic Republic of) | 3 | 2 | 1 | 7 | 4 | 17 | 5 | 8 | 11 | 10 | 6 | 9 | 22 | 13 | 12 | 15 | 16 | 30 | 20 | 14 | 31 | 21 | 18 | 27 | 23 | 19 | 25 | 28 | 24 | 26 | 32 | 29 |
|  | Iraq | 3 | 2 | 1 | 7 | 8 | 15 | 6 | 5 | 11 | 4 | 9 | 12 | 18 | 10 | 14 | 13 | 23 | 27 | 20 | 16 | 30 | 28 | 17 | 22 | 26 | 19 | 21 | 25 | 24 | 32 | 29 | 31 |
|  | Jordan | 2 | 3 | 5 | 6 | 8 | 11 | 7 | 4 | 15 | 1 | 13 | 17 | 14 | 9 | 18 | 10 | 20 | 19 | 12 | 26 | 31 | 24 | 16 | 21 | 22 | 28 | 23 | 27 | 25 | 32 | 30 | 29 |
|  | Kuwait | 2 | 1 | 4 | 3 | 10 | 13 | 7 | 5 | 14 | 6 | 8 | 12 | 17 | 9 | 16 | 11 | 18 | 26 | 28 | 20 | 30 | 25 | 15 | 27 | 23 | 29 | 19 | 22 | 24 | 32 | 31 | 21 |
|  | Lebanon | 2 | 1 | 3 | 4 | 9 | 14 | 5 | 8 | 16 | 11 | 7 | 10 | 20 | 12 | 6 | 15 | 28 | 25 | 17 | 13 | 31 | 19 | 22 | 21 | 26 | 23 | 18 | 27 | 24 | 30 | 32 | 29 |
|  | Libya | 1 | 2 | 3 | 4 | 10 | 13 | 7 | 5 | 12 | 11 | 8 | 14 | 20 | 16 | 6 | 15 | 26 | 9 | 25 | 17 | 31 | 27 | 19 | 23 | 21 | 18 | 22 | 28 | 24 | 29 | 32 | 30 |
|  | Morocco | 2 | 1 | 4 | 5 | 12 | 6 | 7 | 3 | 19 | 13 | 11 | 14 | 16 | 9 | 8 | 15 | 23 | 10 | 22 | 20 | 27 | 24 | 21 | 17 | 26 | 18 | 29 | 28 | 25 | 31 | 32 | 30 |
|  | Oman | 1 | 4 | 3 | 6 | 7 | 15 | 9 | 2 | 8 | 12 | 5 | 10 | 19 | 14 | 11 | 13 | 20 | 22 | 28 | 16 | 30 | 24 | 18 | 23 | 27 | 31 | 26 | 32 | 21 | 29 | 17 | 25 |
|  | Palestine | 3 | 2 | 1 | 6 | 8 | 16 | 5 | 7 | 10 | 4 | 11 | 14 | 19 | 12 | 9 | 13 | 23 | 24 | 30 | 15 | 31 | 26 | 17 | 21 | 25 | 29 | 22 | 18 | 20 | 27 | 32 | 28 |
|  | Qatar | 1 | 2 | 3 | 6 | 8 | 16 | 7 | 5 | 9 | 13 | 4 | 12 | 19 | 17 | 14 | 11 | 18 | 21 | 23 | 10 | 31 | 26 | 15 | 29 | 25 | 24 | 27 | 32 | 20 | 28 | 30 | 22 |
|  | Saudi Arabia | 1 | 2 | 4 | 5 | 10 | 16 | 6 | 3 | 15 | 13 | 7 | 11 | 19 | 12 | 14 | 8 | 21 | 17 | 24 | 9 | 29 | 27 | 18 | 20 | 22 | 28 | 26 | 30 | 23 | 31 | 32 | 25 |
|  | Sudan | 1 | 3 | 2 | 9 | 4 | 12 | 10 | 7 | 15 | 5 | 6 | 8 | 20 | 16 | 11 | 17 | 14 | 23 | 24 | 13 | 31 | 21 | 18 | 22 | 25 | 19 | 26 | 29 | 27 | 28 | 32 | 30 |
|  | Syrian Arab Republic | 4 | 3 | 2 | 8 | 9 | 15 | 5 | 10 | 11 | 1 | 6 | 7 | 19 | 13 | 18 | 14 | 21 | 26 | 25 | 12 | 29 | 27 | 17 | 31 | 30 | 22 | 20 | 16 | 23 | 24 | 32 | 28 |
|  | Tunisia | 2 | 1 | 6 | 4 | 7 | 10 | 3 | 9 | 17 | 15 | 5 | 12 | 16 | 13 | 11 | 14 | 27 | 8 | 26 | 19 | 28 | 23 | 20 | 22 | 21 | 18 | 24 | 30 | 25 | 29 | 32 | 31 |
|  | Turkey | 1 | 3 | 2 | 5 | 6 | 15 | 4 | 7 | 16 | 12 | 8 | 9 | 21 | 13 | 14 | 10 | 18 | 20 | 11 | 24 | 31 | 19 | 17 | 28 | 27 | 23 | 26 | 29 | 25 | 30 | 22 | 32 |
|  | United Arab Emirates | 2 | 5 | 1 | 6 | 12 | 22 | 10 | 3 | 11 | 17 | 9 | 14 | 18 | 15 | 13 | 4 | 7 | 27 | 24 | 20 | 29 | 26 | 8 | 23 | 25 | 16 | 19 | 30 | 21 | 31 | 32 | 28 |
|  | Yemen | 2 | 3 | 4 | 7 | 1 | 13 | 8 | 9 | 15 | 5 | 6 | 10 | 19 | 17 | 11 | 18 | 14 | 21 | 26 | 12 | 31 | 20 | 24 | 23 | 22 | 16 | 25 | 29 | 27 | 28 | 32 | 30 |
| **South Asia** | | **1** | **2** | **6** | **8** | **3** | **5** | **9** | **7** | **15** | **20** | **12** | **16** | **4** | **14** | **11** | **23** | **17** | **18** | **19** | **13** | **10** | **26** | **25** | **21** | **24** | **22** | **27** | **29** | **28** | **32** | **30** | **31** |
|  | Bangladesh | 1 | 2 | 3 | 11 | 5 | 4 | 8 | 6 | 9 | 20 | 10 | 14 | 7 | 15 | 12 | 22 | 17 | 18 | 19 | 16 | 13 | 26 | 25 | 21 | 24 | 23 | 28 | 31 | 27 | 32 | 30 | 29 |
|  | Bhutan | 1 | 2 | 3 | 8 | 5 | 4 | 9 | 6 | 15 | 20 | 10 | 17 | 7 | 11 | 13 | 21 | 16 | 18 | 19 | 14 | 12 | 27 | 25 | 22 | 23 | 24 | 28 | 29 | 26 | 32 | 31 | 30 |
|  | India | 1 | 2 | 6 | 8 | 3 | 4 | 10 | 7 | 14 | 20 | 11 | 15 | 5 | 17 | 13 | 23 | 16 | 18 | 19 | 12 | 9 | 27 | 25 | 21 | 24 | 22 | 28 | 30 | 26 | 32 | 29 | 31 |
|  | Nepal | 1 | 3 | 5 | 10 | 4 | 2 | 8 | 6 | 17 | 19 | 9 | 15 | 7 | 11 | 12 | 21 | 16 | 18 | 22 | 13 | 14 | 26 | 25 | 20 | 23 | 24 | 28 | 29 | 27 | 32 | 30 | 31 |
|  | Pakistan | 1 | 2 | 5 | 8 | 10 | 12 | 9 | 7 | 16 | 20 | 13 | 14 | 3 | 6 | 4 | 26 | 15 | 18 | 11 | 17 | 21 | 25 | 28 | 19 | 23 | 22 | 24 | 27 | 29 | 30 | 32 | 31 |
| **East Asia** | | **7** | **8** | **5** | **3** | **4** | **10** | **2** | **9** | **1** | **6** | **15** | **11** | **18** | **16** | **26** | **12** | **14** | **13** | **25** | **29** | **32** | **24** | **17** | **27** | **20** | **28** | **22** | **23** | **21** | **19** | **30** | **31** |
|  | China | 7 | 8 | 5 | 3 | 4 | 10 | 2 | 9 | 1 | 6 | 15 | 11 | 18 | 16 | 26 | 12 | 14 | 13 | 25 | 29 | 32 | 24 | 17 | 27 | 20 | 28 | 22 | 23 | 21 | 19 | 30 | 31 |
|  | Democratic People's Republic of Korea | 6 | 8 | 7 | 5 | 1 | 9 | 4 | 10 | 3 | 2 | 15 | 11 | 17 | 16 | 23 | 13 | 14 | 12 | 26 | 28 | 32 | 22 | 18 | 24 | 20 | 29 | 21 | 19 | 27 | 25 | 30 | 31 |
|  | Taiwan (Province of China) | 5 | 3 | 7 | 1 | 6 | 15 | 2 | 8 | 9 | 14 | 12 | 17 | 4 | 16 | 28 | 13 | 11 | 10 | 21 | 25 | 18 | 22 | 19 | 29 | 20 | 30 | 24 | 23 | 27 | 32 | 26 | 31 |
| **Oceania** | | **3** | **1** | **9** | **6** | **2** | **4** | **5** | **10** | **11** | **7** | **8** | **13** | **12** | **14** | **15** | **21** | **19** | **17** | **20** | **18** | **28** | **25** | **27** | **23** | **29** | **31** | **22** | **16** | **26** | **32** | **30** | **24** |
|  | American Samoa | 2 | 1 | 10 | 4 | 3 | 7 | 5 | 6 | 8 | 12 | 11 | 14 | 21 | 9 | 20 | 15 | 25 | 17 | 31 | 19 | 27 | 26 | 23 | 22 | 28 | 32 | 18 | 13 | 24 | 30 | 29 | 16 |
|  | Cook Islands | 2 | 1 | 6 | 7 | 5 | 10 | 3 | 8 | 4 | 11 | 9 | 18 | 13 | 14 | 20 | 12 | 17 | 27 | 25 | 22 | 29 | 24 | 19 | 21 | 28 | 31 | 15 | 23 | 26 | 32 | 30 | 16 |
|  | Fiji | 3 | 1 | 9 | 5 | 6 | 2 | 10 | 7 | 8 | 11 | 4 | 13 | 14 | 20 | 15 | 16 | 19 | 28 | 12 | 18 | 27 | 24 | 23 | 21 | 29 | 31 | 22 | 17 | 25 | 32 | 30 | 26 |
|  | Guam | 7 | 3 | 11 | 2 | 5 | 9 | 1 | 4 | 6 | 10 | 8 | 17 | 15 | 14 | 24 | 13 | 16 | 12 | 29 | 20 | 23 | 27 | 18 | 21 | 30 | 31 | 19 | 22 | 25 | 32 | 28 | 26 |
|  | Kiribati | 2 | 3 | 13 | 10 | 4 | 1 | 8 | 11 | 5 | 9 | 14 | 17 | 6 | 18 | 16 | 23 | 12 | 20 | 7 | 22 | 21 | 27 | 15 | 29 | 26 | 30 | 25 | 19 | 28 | 32 | 31 | 24 |
|  | Marshall Islands | 4 | 1 | 11 | 6 | 2 | 3 | 5 | 9 | 7 | 8 | 10 | 12 | 14 | 13 | 18 | 19 | 20 | 17 | 29 | 16 | 27 | 26 | 24 | 22 | 28 | 31 | 21 | 15 | 25 | 32 | 30 | 23 |
|  | Micronesia (Federated States of) | 3 | 1 | 11 | 6 | 2 | 4 | 5 | 9 | 7 | 8 | 10 | 12 | 14 | 13 | 18 | 17 | 20 | 19 | 27 | 15 | 28 | 26 | 24 | 22 | 29 | 31 | 21 | 16 | 25 | 32 | 30 | 23 |
|  | Nauru | 3 | 1 | 11 | 6 | 2 | 5 | 4 | 7 | 8 | 10 | 9 | 12 | 13 | 14 | 20 | 16 | 18 | 19 | 28 | 15 | 27 | 26 | 22 | 23 | 29 | 30 | 21 | 17 | 25 | 32 | 31 | 24 |
|  | Niue | 2 | 1 | 10 | 4 | 6 | 5 | 3 | 7 | 9 | 11 | 8 | 13 | 15 | 12 | 22 | 14 | 17 | 19 | 27 | 18 | 28 | 25 | 20 | 24 | 29 | 31 | 21 | 16 | 26 | 32 | 30 | 23 |
|  | Northern Mariana Islands | 2 | 3 | 9 | 4 | 5 | 7 | 1 | 6 | 10 | 11 | 8 | 14 | 12 | 16 | 26 | 13 | 19 | 15 | 29 | 23 | 18 | 27 | 20 | 22 | 28 | 30 | 21 | 17 | 25 | 31 | 32 | 24 |
|  | Palau | 1 | 4 | 10 | 8 | 6 | 3 | 2 | 9 | 7 | 16 | 12 | 15 | 5 | 19 | 24 | 11 | 14 | 25 | 32 | 23 | 22 | 17 | 20 | 13 | 28 | 29 | 21 | 31 | 26 | 30 | 27 | 18 |
|  | Papua New Guinea | 3 | 1 | 9 | 7 | 2 | 4 | 5 | 12 | 21 | 6 | 8 | 10 | 11 | 13 | 14 | 19 | 18 | 15 | 25 | 17 | 28 | 24 | 27 | 23 | 29 | 31 | 20 | 16 | 26 | 32 | 30 | 22 |
|  | Samoa | 1 | 2 | 10 | 8 | 4 | 3 | 15 | 5 | 11 | 9 | 7 | 16 | 21 | 12 | 6 | 19 | 25 | 14 | 13 | 18 | 29 | 17 | 24 | 20 | 26 | 31 | 23 | 22 | 27 | 32 | 30 | 28 |
|  | Solomon Islands | 5 | 1 | 10 | 8 | 2 | 3 | 6 | 4 | 9 | 7 | 11 | 13 | 15 | 16 | 19 | 20 | 18 | 17 | 29 | 12 | 28 | 23 | 26 | 22 | 27 | 30 | 21 | 14 | 24 | 32 | 31 | 25 |
|  | Tokelau | 3 | 1 | 11 | 7 | 4 | 2 | 5 | 8 | 6 | 10 | 9 | 12 | 15 | 13 | 19 | 16 | 20 | 17 | 27 | 18 | 28 | 26 | 23 | 25 | 29 | 31 | 21 | 14 | 24 | 32 | 30 | 22 |
|  | Tonga | 7 | 2 | 10 | 11 | 5 | 4 | 8 | 6 | 3 | 12 | 9 | 14 | 15 | 13 | 18 | 16 | 20 | 19 | 1 | 25 | 28 | 26 | 24 | 27 | 29 | 31 | 23 | 17 | 21 | 32 | 30 | 22 |
|  | Tuvalu | 3 | 1 | 11 | 7 | 2 | 4 | 5 | 8 | 6 | 9 | 10 | 12 | 14 | 13 | 18 | 19 | 20 | 15 | 27 | 16 | 28 | 25 | 24 | 23 | 29 | 30 | 21 | 17 | 26 | 32 | 31 | 22 |
|  | Vanuatu | 4 | 1 | 11 | 8 | 2 | 3 | 5 | 9 | 6 | 7 | 10 | 12 | 13 | 15 | 14 | 20 | 19 | 16 | 27 | 17 | 28 | 24 | 26 | 22 | 29 | 31 | 21 | 18 | 25 | 32 | 30 | 23 |
| **Southeast Asia** | | **2** | **1** | **6** | **3** | **10** | **8** | **4** | **7** | **9** | **5** | **11** | **13** | **16** | **12** | **17** | **15** | **19** | **14** | **20** | **21** | **25** | **26** | **18** | **22** | **24** | **28** | **29** | **23** | **30** | **32** | **27** | **31** |
|  | Cambodia | 1 | 2 | 6 | 5 | 9 | 8 | 7 | 10 | 3 | 4 | 12 | 13 | 15 | 11 | 17 | 16 | 18 | 14 | 21 | 19 | 29 | 24 | 22 | 23 | 27 | 25 | 28 | 20 | 30 | 32 | 26 | 31 |
|  | Indonesia | 2 | 1 | 6 | 3 | 10 | 7 | 4 | 8 | 15 | 5 | 11 | 12 | 16 | 9 | 17 | 13 | 19 | 14 | 20 | 22 | 29 | 24 | 18 | 23 | 27 | 28 | 25 | 21 | 30 | 32 | 26 | 31 |
|  | Lao People's Democratic Republic | 2 | 1 | 7 | 4 | 9 | 6 | 5 | 8 | 10 | 3 | 12 | 13 | 16 | 11 | 17 | 15 | 18 | 14 | 22 | 20 | 29 | 24 | 21 | 23 | 25 | 27 | 26 | 19 | 30 | 32 | 28 | 31 |
|  | Malaysia | 2 | 1 | 8 | 3 | 12 | 10 | 9 | 5 | 11 | 13 | 6 | 7 | 15 | 14 | 18 | 19 | 22 | 4 | 17 | 16 | 25 | 23 | 21 | 20 | 28 | 24 | 27 | 26 | 30 | 32 | 29 | 31 |
|  | Maldives | 1 | 3 | 2 | 6 | 11 | 10 | 8 | 5 | 7 | 14 | 4 | 15 | 16 | 9 | 13 | 12 | 19 | 25 | 31 | 21 | 28 | 20 | 18 | 22 | 29 | 27 | 26 | 30 | 23 | 32 | 17 | 24 |
|  | Mauritius | 2 | 1 | 7 | 3 | 8 | 11 | 5 | 6 | 19 | 4 | 9 | 13 | 14 | 10 | 15 | 12 | 17 | 18 | 20 | 24 | 28 | 27 | 16 | 29 | 25 | 23 | 26 | 22 | 21 | 32 | 31 | 30 |
|  | Myanmar | 2 | 3 | 8 | 5 | 9 | 7 | 6 | 1 | 13 | 4 | 11 | 12 | 16 | 10 | 17 | 15 | 18 | 14 | 22 | 21 | 29 | 24 | 20 | 23 | 27 | 28 | 26 | 19 | 30 | 32 | 25 | 31 |
|  | Philippines | 2 | 1 | 8 | 3 | 12 | 9 | 5 | 7 | 6 | 4 | 11 | 14 | 16 | 10 | 23 | 15 | 22 | 13 | 20 | 18 | 29 | 24 | 17 | 21 | 26 | 27 | 25 | 19 | 31 | 32 | 28 | 30 |
|  | Seychelles | 5 | 1 | 4 | 2 | 10 | 6 | 7 | 3 | 15 | 13 | 14 | 9 | 12 | 8 | 18 | 16 | 19 | 17 | 20 | 11 | 23 | 24 | 25 | 32 | 31 | 21 | 27 | 29 | 28 | 26 | 30 | 22 |
|  | Sri Lanka | 1 | 2 | 3 | 9 | 10 | 11 | 6 | 4 | 14 | 5 | 7 | 8 | 13 | 12 | 15 | 18 | 16 | 19 | 21 | 20 | 23 | 28 | 17 | 24 | 22 | 29 | 30 | 27 | 26 | 32 | 25 | 31 |
|  | Thailand | 3 | 4 | 7 | 6 | 9 | 8 | 2 | 10 | 1 | 5 | 12 | 19 | 15 | 11 | 23 | 13 | 16 | 14 | 21 | 18 | 25 | 28 | 20 | 27 | 17 | 24 | 26 | 29 | 31 | 32 | 22 | 30 |
|  | Timor-Leste | 1 | 3 | 5 | 4 | 9 | 7 | 6 | 8 | 10 | 2 | 11 | 12 | 16 | 13 | 15 | 17 | 18 | 14 | 19 | 22 | 29 | 24 | 20 | 23 | 27 | 28 | 26 | 21 | 31 | 32 | 25 | 30 |
|  | Viet Nam | 1 | 2 | 7 | 3 | 5 | 6 | 4 | 10 | 20 | 14 | 11 | 9 | 12 | 13 | 16 | 17 | 23 | 8 | 18 | 21 | 19 | 25 | 22 | 15 | 27 | 24 | 29 | 31 | 28 | 32 | 26 | 30 |
| **Central Sub-Saharan Africa** | | **3** | **2** | **7** | **8** | **5** | **1** | **9** | **4** | **12** | **6** | **13** | **14** | **18** | **15** | **11** | **19** | **10** | **21** | **17** | **16** | **31** | **20** | **23** | **24** | **29** | **27** | **22** | **26** | **28** | **32** | **30** | **25** |
|  | Angola | 2 | 3 | 7 | 5 | 6 | 1 | 9 | 4 | 13 | 8 | 12 | 15 | 18 | 16 | 11 | 19 | 10 | 21 | 17 | 14 | 31 | 20 | 22 | 24 | 29 | 26 | 23 | 27 | 28 | 32 | 30 | 25 |
|  | Central African Republic | 3 | 2 | 7 | 8 | 4 | 1 | 9 | 6 | 12 | 5 | 16 | 13 | 15 | 17 | 11 | 21 | 10 | 20 | 18 | 14 | 31 | 19 | 24 | 25 | 27 | 23 | 22 | 26 | 28 | 32 | 30 | 29 |
|  | Congo | 3 | 2 | 8 | 5 | 6 | 1 | 7 | 4 | 12 | 9 | 13 | 18 | 17 | 11 | 15 | 14 | 10 | 22 | 19 | 16 | 31 | 20 | 21 | 25 | 29 | 28 | 23 | 24 | 27 | 32 | 30 | 26 |
|  | Democratic Republic of the Congo | 3 | 2 | 7 | 9 | 5 | 1 | 8 | 4 | 11 | 6 | 14 | 13 | 18 | 15 | 10 | 20 | 12 | 21 | 16 | 17 | 31 | 19 | 23 | 25 | 29 | 27 | 22 | 26 | 28 | 32 | 30 | 24 |
|  | Equatorial Guinea | 1 | 3 | 7 | 5 | 9 | 2 | 6 | 4 | 11 | 8 | 10 | 16 | 18 | 12 | 17 | 14 | 13 | 22 | 15 | 19 | 28 | 21 | 20 | 25 | 30 | 29 | 23 | 27 | 26 | 32 | 31 | 24 |
|  | Gabon | 3 | 1 | 7 | 5 | 8 | 2 | 6 | 4 | 12 | 9 | 11 | 16 | 19 | 10 | 15 | 14 | 13 | 23 | 18 | 17 | 29 | 21 | 20 | 26 | 30 | 28 | 22 | 27 | 25 | 32 | 31 | 24 |
| **Eastern Sub-Saharan Africa** | | **1** | **3** | **8** | **5** | **4** | **2** | **20** | **7** | **13** | **12** | **18** | **14** | **17** | **11** | **6** | **22** | **9** | **15** | **21** | **10** | **27** | **19** | **23** | **16** | **29** | **26** | **24** | **28** | **25** | **32** | **30** | **31** |
|  | Burundi | 1 | 3 | 8 | 7 | 4 | 2 | 17 | 6 | 11 | 10 | 20 | 18 | 13 | 14 | 5 | 22 | 9 | 12 | 21 | 15 | 27 | 16 | 24 | 19 | 29 | 23 | 26 | 28 | 25 | 32 | 30 | 31 |
|  | Comoros | 1 | 3 | 8 | 4 | 5 | 2 | 13 | 6 | 12 | 11 | 19 | 18 | 16 | 10 | 7 | 21 | 9 | 14 | 22 | 17 | 28 | 15 | 23 | 20 | 29 | 27 | 26 | 24 | 25 | 32 | 30 | 31 |
|  | Djibouti | 1 | 3 | 7 | 4 | 5 | 2 | 10 | 6 | 12 | 13 | 18 | 21 | 15 | 11 | 8 | 20 | 9 | 14 | 19 | 17 | 27 | 16 | 23 | 22 | 29 | 26 | 25 | 28 | 24 | 32 | 30 | 31 |
|  | Eritrea | 1 | 3 | 8 | 5 | 4 | 2 | 11 | 6 | 14 | 10 | 20 | 18 | 15 | 12 | 7 | 22 | 9 | 13 | 21 | 17 | 27 | 16 | 23 | 19 | 29 | 24 | 25 | 26 | 28 | 32 | 30 | 31 |
|  | Ethiopia | 1 | 3 | 5 | 9 | 6 | 2 | 18 | 13 | 15 | 16 | 11 | 8 | 17 | 12 | 7 | 23 | 21 | 14 | 19 | 4 | 30 | 20 | 22 | 10 | 24 | 26 | 25 | 28 | 27 | 32 | 29 | 31 |
|  | Kenya | 1 | 2 | 6 | 7 | 5 | 3 | 17 | 4 | 12 | 14 | 16 | 15 | 10 | 13 | 9 | 18 | 8 | 11 | 27 | 20 | 23 | 19 | 25 | 21 | 26 | 24 | 28 | 29 | 22 | 32 | 30 | 31 |
|  | Madagascar | 2 | 3 | 8 | 5 | 4 | 1 | 13 | 6 | 12 | 10 | 20 | 18 | 16 | 11 | 7 | 22 | 9 | 15 | 21 | 17 | 28 | 14 | 23 | 19 | 29 | 26 | 25 | 24 | 27 | 32 | 30 | 31 |
|  | Malawi | 1 | 3 | 8 | 9 | 14 | 2 | 17 | 5 | 12 | 15 | 10 | 13 | 16 | 11 | 7 | 22 | 4 | 24 | 20 | 23 | 32 | 6 | 21 | 18 | 30 | 28 | 19 | 26 | 25 | 31 | 27 | 29 |
|  | Mozambique | 1 | 3 | 7 | 6 | 11 | 2 | 13 | 5 | 10 | 8 | 17 | 16 | 22 | 9 | 4 | 20 | 19 | 30 | 18 | 14 | 28 | 12 | 21 | 15 | 27 | 23 | 24 | 26 | 25 | 32 | 29 | 31 |
|  | Rwanda | 1 | 3 | 8 | 4 | 6 | 2 | 13 | 5 | 9 | 12 | 19 | 18 | 16 | 10 | 7 | 22 | 11 | 15 | 21 | 17 | 26 | 14 | 23 | 20 | 29 | 25 | 27 | 28 | 24 | 32 | 30 | 31 |
|  | Somalia | 2 | 4 | 7 | 10 | 3 | 1 | 18 | 9 | 11 | 6 | 20 | 15 | 16 | 14 | 5 | 22 | 8 | 12 | 21 | 13 | 29 | 17 | 27 | 19 | 28 | 23 | 24 | 25 | 26 | 32 | 30 | 31 |
|  | South Sudan | 1 | 3 | 8 | 5 | 6 | 2 | 13 | 4 | 12 | 10 | 14 | 19 | 15 | 11 | 7 | 21 | 9 | 16 | 22 | 18 | 26 | 17 | 23 | 20 | 29 | 28 | 25 | 24 | 27 | 32 | 30 | 31 |
|  | Uganda | 1 | 3 | 11 | 4 | 6 | 2 | 16 | 8 | 7 | 13 | 21 | 22 | 15 | 10 | 5 | 18 | 9 | 12 | 20 | 19 | 23 | 14 | 24 | 17 | 29 | 27 | 28 | 26 | 25 | 32 | 31 | 30 |
|  | United Republic of Tanzania | 1 | 3 | 7 | 5 | 6 | 2 | 12 | 4 | 17 | 11 | 19 | 18 | 15 | 10 | 8 | 21 | 9 | 14 | 22 | 16 | 27 | 13 | 23 | 20 | 29 | 28 | 25 | 26 | 24 | 32 | 30 | 31 |
|  | Zambia | 1 | 3 | 8 | 4 | 6 | 2 | 11 | 5 | 17 | 12 | 18 | 19 | 13 | 10 | 7 | 21 | 9 | 15 | 22 | 16 | 28 | 14 | 23 | 20 | 29 | 27 | 24 | 25 | 26 | 32 | 30 | 31 |
| **Southern Sub-Saharan Africa** | | **3** | **2** | **8** | **6** | **9** | **1** | **7** | **4** | **5** | **10** | **14** | **18** | **17** | **12** | **16** | **13** | **11** | **23** | **20** | **31** | **29** | **15** | **21** | **24** | **30** | **26** | **19** | **28** | **22** | **25** | **32** | **27** |
|  | Botswana | 3 | 2 | 7 | 4 | 8 | 1 | 6 | 5 | 16 | 9 | 12 | 15 | 14 | 10 | 22 | 13 | 11 | 24 | 18 | 23 | 27 | 17 | 19 | 32 | 30 | 25 | 20 | 29 | 21 | 28 | 31 | 26 |
|  | Eswatini | 3 | 5 | 8 | 6 | 10 | 2 | 9 | 4 | 1 | 7 | 13 | 16 | 17 | 12 | 15 | 14 | 11 | 21 | 20 | 26 | 28 | 18 | 19 | 24 | 30 | 25 | 22 | 29 | 23 | 31 | 32 | 27 |
|  | Lesotho | 4 | 3 | 10 | 7 | 6 | 1 | 9 | 5 | 2 | 8 | 15 | 17 | 14 | 12 | 13 | 16 | 11 | 20 | 19 | 23 | 29 | 18 | 21 | 26 | 31 | 24 | 22 | 27 | 25 | 32 | 28 | 30 |
|  | Namibia | 1 | 2 | 5 | 8 | 15 | 3 | 12 | 4 | 11 | 9 | 16 | 17 | 7 | 14 | 10 | 18 | 23 | 20 | 13 | 29 | 22 | 6 | 21 | 24 | 32 | 19 | 26 | 27 | 25 | 31 | 28 | 30 |
|  | South Africa | 4 | 3 | 8 | 6 | 10 | 1 | 7 | 2 | 5 | 9 | 15 | 18 | 17 | 12 | 14 | 13 | 11 | 23 | 20 | 32 | 28 | 16 | 19 | 27 | 29 | 26 | 21 | 31 | 22 | 24 | 30 | 25 |
|  | Zimbabwe | 3 | 2 | 10 | 6 | 5 | 1 | 7 | 13 | 4 | 17 | 12 | 16 | 19 | 9 | 15 | 14 | 8 | 22 | 23 | 26 | 31 | 11 | 28 | 20 | 29 | 25 | 18 | 24 | 21 | 27 | 32 | 30 |
| **Western Sub-Saharan Africa** | | **3** | **2** | **5** | **7** | **6** | **1** | **11** | **4** | **9** | **10** | **12** | **14** | **19** | **13** | **8** | **16** | **21** | **17** | **24** | **15** | **31** | **18** | **22** | **26** | **28** | **25** | **20** | **30** | **27** | **32** | **29** | **23** |
|  | Benin | 2 | 3 | 7 | 9 | 5 | 1 | 10 | 4 | 6 | 8 | 11 | 12 | 18 | 15 | 16 | 14 | 17 | 22 | 23 | 13 | 30 | 21 | 20 | 25 | 27 | 26 | 19 | 29 | 28 | 32 | 31 | 24 |
|  | Burkina Faso | 3 | 2 | 6 | 8 | 4 | 1 | 9 | 5 | 11 | 7 | 10 | 13 | 18 | 14 | 15 | 16 | 17 | 21 | 23 | 12 | 30 | 20 | 22 | 24 | 27 | 26 | 19 | 28 | 29 | 32 | 31 | 25 |
|  | Cabo Verde | 2 | 8 | 3 | 9 | 4 | 5 | 7 | 6 | 1 | 10 | 12 | 14 | 19 | 15 | 23 | 11 | 13 | 20 | 25 | 16 | 26 | 22 | 18 | 24 | 30 | 27 | 17 | 29 | 28 | 31 | 32 | 21 |
|  | Cameroon | 3 | 2 | 7 | 6 | 5 | 1 | 8 | 4 | 20 | 9 | 10 | 14 | 19 | 12 | 17 | 11 | 16 | 13 | 21 | 15 | 30 | 22 | 23 | 28 | 27 | 25 | 18 | 29 | 26 | 32 | 31 | 24 |
|  | Chad | 2 | 3 | 8 | 9 | 4 | 1 | 10 | 5 | 6 | 7 | 11 | 12 | 19 | 16 | 14 | 17 | 15 | 21 | 23 | 13 | 29 | 20 | 22 | 25 | 27 | 26 | 18 | 28 | 30 | 32 | 31 | 24 |
|  | Côte d'Ivoire | 2 | 3 | 7 | 6 | 5 | 1 | 9 | 4 | 8 | 10 | 11 | 15 | 19 | 14 | 16 | 13 | 17 | 22 | 23 | 12 | 29 | 21 | 20 | 26 | 27 | 25 | 18 | 30 | 28 | 32 | 31 | 24 |
|  | Gambia | 3 | 4 | 6 | 5 | 10 | 2 | 12 | 9 | 1 | 7 | 8 | 13 | 16 | 14 | 11 | 18 | 21 | 23 | 17 | 15 | 31 | 19 | 20 | 22 | 26 | 29 | 24 | 27 | 28 | 32 | 30 | 25 |
|  | Ghana | 5 | 1 | 3 | 6 | 9 | 2 | 8 | 4 | 7 | 13 | 10 | 16 | 23 | 12 | 19 | 11 | 18 | 25 | 22 | 15 | 29 | 21 | 17 | 31 | 26 | 24 | 14 | 30 | 27 | 32 | 28 | 20 |
|  | Guinea | 3 | 4 | 11 | 7 | 5 | 1 | 9 | 13 | 2 | 12 | 15 | 16 | 8 | 10 | 6 | 21 | 22 | 23 | 18 | 17 | 28 | 14 | 24 | 20 | 29 | 27 | 19 | 25 | 30 | 32 | 31 | 26 |
|  | Guinea-Bissau | 3 | 2 | 6 | 7 | 4 | 1 | 10 | 5 | 8 | 9 | 11 | 13 | 18 | 14 | 15 | 16 | 17 | 21 | 23 | 12 | 31 | 20 | 22 | 24 | 27 | 25 | 19 | 28 | 29 | 32 | 30 | 26 |
|  | Liberia | 3 | 2 | 7 | 9 | 5 | 1 | 10 | 4 | 6 | 8 | 11 | 13 | 19 | 12 | 16 | 15 | 17 | 22 | 23 | 14 | 30 | 21 | 20 | 25 | 28 | 26 | 18 | 29 | 27 | 32 | 31 | 24 |
|  | Mali | 4 | 5 | 7 | 6 | 2 | 1 | 11 | 10 | 3 | 8 | 9 | 14 | 19 | 18 | 13 | 16 | 20 | 25 | 22 | 15 | 29 | 17 | 23 | 21 | 28 | 24 | 12 | 26 | 31 | 32 | 30 | 27 |
|  | Mauritania | 2 | 3 | 8 | 7 | 5 | 1 | 9 | 4 | 6 | 10 | 11 | 14 | 20 | 12 | 16 | 13 | 17 | 23 | 22 | 15 | 30 | 21 | 19 | 25 | 29 | 27 | 18 | 28 | 26 | 32 | 31 | 24 |
|  | Niger | 2 | 4 | 6 | 7 | 3 | 1 | 8 | 10 | 19 | 5 | 9 | 11 | 18 | 14 | 12 | 16 | 15 | 24 | 21 | 13 | 29 | 20 | 22 | 32 | 26 | 25 | 17 | 27 | 28 | 31 | 30 | 23 |
|  | Nigeria | 2 | 1 | 5 | 7 | 12 | 3 | 9 | 6 | 11 | 8 | 10 | 13 | 20 | 14 | 4 | 17 | 24 | 15 | 31 | 16 | 30 | 18 | 19 | 27 | 26 | 21 | 25 | 32 | 23 | 29 | 28 | 22 |
|  | Sao Tome and Principe | 1 | 3 | 15 | 5 | 7 | 2 | 4 | 6 | 8 | 12 | 10 | 13 | 21 | 9 | 26 | 16 | 17 | 28 | 22 | 14 | 31 | 23 | 18 | 19 | 20 | 27 | 11 | 25 | 29 | 32 | 30 | 24 |
|  | Senegal | 3 | 2 | 6 | 7 | 5 | 1 | 9 | 4 | 12 | 8 | 10 | 11 | 19 | 14 | 15 | 16 | 17 | 23 | 22 | 13 | 29 | 20 | 21 | 25 | 27 | 26 | 18 | 30 | 28 | 32 | 31 | 24 |
|  | Sierra Leone | 3 | 2 | 6 | 9 | 4 | 1 | 10 | 5 | 7 | 8 | 11 | 13 | 18 | 14 | 15 | 16 | 17 | 21 | 23 | 12 | 31 | 20 | 22 | 25 | 27 | 26 | 19 | 28 | 29 | 32 | 30 | 24 |
|  | Togo | 3 | 2 | 7 | 8 | 5 | 1 | 9 | 4 | 6 | 10 | 11 | 12 | 18 | 14 | 16 | 15 | 17 | 22 | 23 | 13 | 30 | 20 | 21 | 25 | 27 | 26 | 19 | 28 | 29 | 32 | 31 | 24 |

Colour intensity and number ranking are assigned according to the rank of absolute number of deaths of each cancer type among all cancer types. Dark red and number ranking of 1 indicate the highest rank and greatest absolute number of deaths. Dark green and number ranking of 32 indicate the lowest rank and the smallest absolute number of deaths.

eTable 10. AYA cancer ranking by the number of DALYs at the global level and according to SDI quintile, super-regions, regions, and countries, both sexes, 2019

| **Location** | | **Other malignant neoplasms** | **Breast cancer** | **Brain and central nervous system cancer** | **Colon and rectum cancer** | **Stomach cancer** | **Cervical cancer** | **Tracheal, bronchus, and lung cancer** | **Non-Hodgkin lymphoma** | **Liver cancer** | **Other leukemia** | **Acute lymphoid leukemia** | **Acute myeloid leukemia** | **Lip and oral cavity cancer** | **Ovarian cancer** | **Hodgkin lymphoma** | **Pancreatic cancer** | **Nasopharynx cancer** | **Testicular cancer** | **Esophageal cancer** | **Chronic myeloid leukemia** | **Malignant skin melanoma** | **Other pharynx cancer** | **Kidney cancer** | **Thyroid cancer** | **Gallbladder and biliary tract cancer** | **Larynx cancer** | **Bladder cancer** | **Uterine cancer** | **Multiple myeloma** | **Chronic lymphoid leukemia** | **Mesothelioma** | **Prostate cancer** |
| --- | --- | --- | --- | --- | --- | --- | --- | --- | --- | --- | --- | --- | --- | --- | --- | --- | --- | --- | --- | --- | --- | --- | --- | --- | --- | --- | --- | --- | --- | --- | --- | --- | --- |
| **Global** | | **1** | **2** | **3** | **4** | **5** | **6** | **7** | **8** | **9** | **10** | **11** | **12** | **13** | **14** | **15** | **16** | **17** | **18** | **19** | **20** | **21** | **22** | **23** | **24** | **25** | **26** | **27** | **28** | **29** | **30** | **31** | **32** |
|  | **Low SDI** | **1** | **3** | **5** | **8** | **4** | **2** | **12** | **6** | **11** | **9** | **14** | **13** | **10** | **16** | **7** | **23** | **18** | **20** | **17** | **15** | **21** | **22** | **26** | **19** | **27** | **24** | **25** | **28** | **29** | **32** | **31** | **30** |
|  | **Low-middle SDI** | **1** | **2** | **5** | **7** | **4** | **3** | **8** | **6** | **11** | **10** | **13** | **12** | **9** | **16** | **14** | **21** | **19** | **20** | **18** | **17** | **26** | **15** | **24** | **22** | **25** | **23** | **27** | **28** | **29** | **32** | **30** | **31** |
|  | **Middle SDI** | **1** | **2** | **4** | **3** | **6** | **10** | **5** | **8** | **7** | **9** | **11** | **12** | **15** | **13** | **17** | **14** | **16** | **19** | **18** | **21** | **25** | **22** | **20** | **23** | **26** | **28** | **24** | **27** | **29** | **30** | **31** | **32** |
|  | **High-middle SDI** | **1** | **4** | **3** | **2** | **6** | **8** | **5** | **7** | **9** | **10** | **11** | **12** | **20** | **14** | **17** | **13** | **15** | **16** | **21** | **22** | **18** | **29** | **19** | **26** | **23** | **28** | **25** | **24** | **27** | **30** | **31** | **32** |
|  | **High SDI** | **1** | **2** | **3** | **4** | **8** | **9** | **5** | **6** | **12** | **15** | **11** | **7** | **19** | **14** | **17** | **13** | **22** | **16** | **20** | **21** | **10** | **28** | **18** | **23** | **27** | **30** | **25** | **24** | **26** | **32** | **31** | **29** |
| **Central Asia** | | **2** | **3** | **1** | **6** | **4** | **7** | **5** | **8** | **11** | **9** | **12** | **10** | **18** | **14** | **13** | **16** | **21** | **19** | **15** | **24** | **23** | **25** | **17** | **30** | **31** | **22** | **27** | **20** | **28** | **32** | **29** | **26** |
|  | Armenia | 2 | 3 | 1 | 5 | 6 | 7 | 4 | 9 | 13 | 8 | 10 | 11 | 21 | 14 | 17 | 12 | 24 | 15 | 30 | 29 | 19 | 32 | 18 | 27 | 31 | 22 | 23 | 20 | 28 | 25 | 16 | 26 |
|  | Azerbaijan | 1 | 4 | 2 | 6 | 5 | 8 | 3 | 10 | 17 | 7 | 12 | 9 | 21 | 14 | 11 | 15 | 27 | 18 | 13 | 26 | 24 | 28 | 16 | 25 | 30 | 19 | 23 | 22 | 29 | 31 | 32 | 20 |
|  | Georgia | 2 | 1 | 4 | 6 | 5 | 8 | 3 | 9 | 15 | 7 | 16 | 12 | 22 | 13 | 11 | 14 | 29 | 10 | 24 | 23 | 18 | 28 | 19 | 26 | 30 | 20 | 25 | 17 | 27 | 32 | 31 | 21 |
|  | Kazakhstan | 1 | 4 | 3 | 6 | 5 | 2 | 7 | 9 | 12 | 20 | 11 | 8 | 18 | 10 | 13 | 14 | 25 | 17 | 19 | 21 | 16 | 28 | 15 | 24 | 32 | 27 | 26 | 22 | 29 | 31 | 30 | 23 |
|  | Kyrgyzstan | 1 | 5 | 3 | 6 | 2 | 4 | 8 | 11 | 14 | 9 | 10 | 7 | 19 | 12 | 17 | 13 | 20 | 16 | 18 | 23 | 22 | 24 | 15 | 25 | 28 | 26 | 27 | 21 | 31 | 30 | 32 | 29 |
|  | Mongolia | 3 | 7 | 4 | 6 | 2 | 5 | 9 | 10 | 1 | 20 | 14 | 8 | 17 | 12 | 19 | 13 | 25 | 29 | 11 | 21 | 27 | 28 | 16 | 26 | 18 | 32 | 24 | 15 | 23 | 30 | 31 | 22 |
|  | Tajikistan | 3 | 4 | 1 | 5 | 2 | 11 | 6 | 7 | 12 | 8 | 10 | 9 | 20 | 16 | 18 | 17 | 25 | 32 | 13 | 23 | 22 | 26 | 14 | 31 | 30 | 21 | 24 | 15 | 28 | 29 | 19 | 27 |
|  | Turkmenistan | 1 | 3 | 2 | 7 | 4 | 5 | 6 | 12 | 9 | 10 | 13 | 11 | 19 | 15 | 8 | 21 | 22 | 18 | 14 | 25 | 24 | 26 | 17 | 28 | 31 | 27 | 29 | 30 | 16 | 32 | 23 | 20 |
|  | Uzbekistan | 2 | 3 | 1 | 7 | 4 | 8 | 6 | 5 | 12 | 9 | 11 | 10 | 15 | 16 | 13 | 17 | 19 | 20 | 14 | 24 | 25 | 23 | 18 | 32 | 30 | 22 | 26 | 21 | 27 | 28 | 29 | 31 |
| **Central Europe** | | **2** | **3** | **1** | **4** | **9** | **6** | **5** | **7** | **18** | **16** | **14** | **10** | **19** | **12** | **15** | **13** | **24** | **8** | **21** | **25** | **11** | **22** | **17** | **28** | **27** | **23** | **26** | **20** | **29** | **30** | **31** | **32** |
|  | Albania | 1 | 5 | 2 | 6 | 4 | 13 | 3 | 9 | 11 | 12 | 8 | 7 | 17 | 16 | 15 | 14 | 28 | 10 | 23 | 25 | 18 | 30 | 19 | 24 | 26 | 22 | 29 | 21 | 31 | 27 | 32 | 20 |
|  | Bosnia and Herzegovina | 3 | 4 | 1 | 2 | 7 | 8 | 5 | 6 | 16 | 18 | 10 | 9 | 20 | 11 | 15 | 14 | 30 | 13 | 24 | 21 | 12 | 22 | 17 | 27 | 25 | 23 | 26 | 19 | 29 | 31 | 28 | 32 |
|  | Bulgaria | 6 | 3 | 1 | 5 | 8 | 4 | 2 | 7 | 17 | 12 | 15 | 10 | 20 | 11 | 14 | 13 | 27 | 9 | 23 | 25 | 16 | 22 | 19 | 26 | 29 | 21 | 24 | 18 | 28 | 32 | 31 | 30 |
|  | Croatia | 2 | 3 | 1 | 4 | 10 | 11 | 5 | 6 | 17 | 18 | 12 | 9 | 19 | 13 | 15 | 14 | 29 | 8 | 21 | 22 | 7 | 20 | 16 | 28 | 26 | 23 | 25 | 27 | 24 | 32 | 30 | 31 |
|  | Czechia | 2 | 3 | 1 | 4 | 11 | 5 | 6 | 9 | 18 | 17 | 16 | 10 | 19 | 12 | 14 | 13 | 29 | 7 | 20 | 21 | 8 | 24 | 15 | 25 | 23 | 28 | 27 | 22 | 26 | 32 | 30 | 31 |
|  | Hungary | 3 | 2 | 1 | 5 | 11 | 6 | 4 | 9 | 19 | 18 | 14 | 8 | 15 | 13 | 17 | 12 | 24 | 7 | 23 | 21 | 10 | 20 | 16 | 29 | 26 | 22 | 27 | 25 | 28 | 31 | 30 | 32 |
|  | Montenegro | 4 | 3 | 1 | 5 | 13 | 6 | 2 | 7 | 15 | 23 | 11 | 8 | 19 | 16 | 9 | 14 | 31 | 10 | 22 | 20 | 12 | 26 | 18 | 24 | 27 | 17 | 25 | 21 | 29 | 28 | 32 | 30 |
|  | North Macedonia | 4 | 2 | 1 | 5 | 6 | 8 | 3 | 11 | 14 | 7 | 17 | 16 | 20 | 13 | 15 | 12 | 28 | 9 | 26 | 24 | 10 | 23 | 19 | 31 | 25 | 21 | 22 | 18 | 29 | 30 | 32 | 27 |
|  | Poland | 2 | 3 | 1 | 4 | 9 | 11 | 5 | 6 | 20 | 18 | 14 | 10 | 17 | 12 | 15 | 13 | 27 | 7 | 19 | 21 | 8 | 23 | 16 | 29 | 24 | 22 | 25 | 26 | 28 | 31 | 30 | 32 |
|  | Romania | 2 | 6 | 1 | 4 | 8 | 3 | 5 | 7 | 19 | 15 | 14 | 9 | 18 | 11 | 16 | 12 | 20 | 10 | 24 | 28 | 13 | 21 | 17 | 26 | 29 | 22 | 25 | 23 | 27 | 30 | 31 | 32 |
|  | Serbia | 3 | 2 | 1 | 5 | 10 | 6 | 4 | 7 | 17 | 15 | 16 | 11 | 19 | 12 | 13 | 14 | 24 | 8 | 21 | 22 | 9 | 25 | 18 | 28 | 29 | 26 | 27 | 20 | 30 | 23 | 31 | 32 |
|  | Slovakia | 2 | 4 | 1 | 3 | 10 | 5 | 6 | 7 | 18 | 19 | 12 | 9 | 16 | 13 | 14 | 15 | 27 | 8 | 22 | 29 | 11 | 20 | 17 | 26 | 23 | 24 | 28 | 21 | 25 | 32 | 30 | 31 |
|  | Slovenia | 2 | 3 | 1 | 4 | 9 | 11 | 6 | 7 | 14 | 21 | 15 | 8 | 18 | 12 | 16 | 13 | 30 | 10 | 22 | 20 | 5 | 19 | 17 | 26 | 24 | 27 | 25 | 29 | 23 | 32 | 28 | 31 |
| **Eastern Europe** | | **2** | **3** | **1** | **5** | **4** | **6** | **7** | **8** | **17** | **19** | **12** | **14** | **16** | **13** | **10** | **11** | **28** | **15** | **21** | **22** | **9** | **23** | **18** | **24** | **31** | **25** | **27** | **20** | **29** | **32** | **26** | **30** |
|  | Belarus | 1 | 4 | 2 | 6 | 3 | 5 | 8 | 7 | 20 | 22 | 12 | 9 | 19 | 14 | 10 | 15 | 29 | 13 | 21 | 16 | 11 | 26 | 17 | 25 | 31 | 24 | 30 | 28 | 27 | 32 | 18 | 23 |
|  | Estonia | 2 | 3 | 1 | 5 | 4 | 9 | 8 | 6 | 18 | 14 | 11 | 7 | 19 | 13 | 12 | 16 | 26 | 15 | 22 | 20 | 10 | 24 | 17 | 21 | 27 | 29 | 28 | 25 | 23 | 32 | 31 | 30 |
|  | Latvia | 2 | 3 | 1 | 6 | 4 | 8 | 7 | 5 | 17 | 16 | 12 | 13 | 19 | 15 | 10 | 11 | 28 | 14 | 20 | 21 | 9 | 22 | 18 | 27 | 29 | 23 | 25 | 24 | 26 | 32 | 31 | 30 |
|  | Lithuania | 2 | 3 | 1 | 5 | 4 | 8 | 9 | 6 | 17 | 19 | 12 | 7 | 18 | 11 | 14 | 13 | 27 | 15 | 20 | 21 | 10 | 24 | 16 | 25 | 29 | 23 | 28 | 22 | 26 | 32 | 30 | 31 |
|  | Republic of Moldova | 2 | 4 | 1 | 3 | 7 | 5 | 6 | 8 | 20 | 11 | 16 | 12 | 18 | 13 | 10 | 9 | 19 | 15 | 24 | 29 | 14 | 21 | 17 | 26 | 30 | 22 | 25 | 23 | 27 | 32 | 31 | 28 |
|  | Russian Federation | 2 | 3 | 1 | 6 | 5 | 4 | 7 | 8 | 15 | 19 | 10 | 14 | 16 | 11 | 12 | 13 | 28 | 17 | 21 | 22 | 9 | 25 | 18 | 23 | 30 | 24 | 26 | 20 | 27 | 31 | 29 | 32 |
|  | Ukraine | 1 | 4 | 2 | 5 | 3 | 10 | 7 | 6 | 19 | 17 | 13 | 12 | 16 | 14 | 8 | 11 | 26 | 15 | 22 | 20 | 9 | 23 | 18 | 28 | 31 | 25 | 29 | 24 | 30 | 32 | 21 | 27 |
| **Australasia** | | **1** | **3** | **2** | **5** | **11** | **9** | **8** | **7** | **12** | **21** | **10** | **6** | **17** | **14** | **16** | **13** | **23** | **15** | **19** | **22** | **4** | **29** | **18** | **20** | **28** | **31** | **26** | **25** | **24** | **32** | **27** | **30** |
|  | Australia | 2 | 3 | 1 | 5 | 11 | 9 | 8 | 7 | 12 | 20 | 10 | 6 | 16 | 14 | 15 | 13 | 23 | 17 | 19 | 22 | 4 | 29 | 18 | 21 | 28 | 31 | 25 | 26 | 24 | 32 | 27 | 30 |
|  | New Zealand | 1 | 2 | 3 | 5 | 9 | 13 | 8 | 7 | 12 | 20 | 11 | 6 | 18 | 14 | 15 | 16 | 23 | 10 | 22 | 21 | 4 | 29 | 17 | 19 | 27 | 31 | 26 | 25 | 24 | 32 | 30 | 28 |
| **High-income Asia Pacific** | | **1** | **3** | **5** | **4** | **2** | **10** | **6** | **9** | **8** | **14** | **11** | **7** | **15** | **12** | **24** | **13** | **22** | **17** | **25** | **21** | **19** | **30** | **16** | **23** | **18** | **31** | **26** | **20** | **27** | **32** | **29** | **28** |
|  | Brunei Darussalam | 2 | 1 | 5 | 4 | 7 | 8 | 6 | 3 | 10 | 14 | 15 | 9 | 16 | 12 | 17 | 18 | 13 | 19 | 27 | 11 | 21 | 26 | 20 | 24 | 23 | 32 | 28 | 22 | 25 | 29 | 31 | 30 |
|  | Japan | 1 | 2 | 5 | 3 | 4 | 8 | 6 | 9 | 12 | 16 | 10 | 7 | 14 | 11 | 21 | 13 | 25 | 15 | 23 | 22 | 20 | 30 | 17 | 26 | 19 | 31 | 24 | 18 | 27 | 32 | 28 | 29 |
|  | Republic of Korea | 2 | 4 | 6 | 5 | 1 | 10 | 7 | 9 | 3 | 13 | 11 | 8 | 18 | 14 | 22 | 12 | 21 | 25 | 24 | 19 | 20 | 30 | 16 | 17 | 15 | 29 | 26 | 23 | 27 | 32 | 31 | 28 |
|  | Singapore | 1 | 2 | 4 | 3 | 11 | 13 | 5 | 7 | 10 | 18 | 9 | 6 | 16 | 12 | 19 | 14 | 8 | 22 | 25 | 17 | 20 | 29 | 15 | 23 | 24 | 30 | 26 | 21 | 27 | 32 | 31 | 28 |
| **High-income North America** | | **1** | **2** | **3** | **4** | **11** | **7** | **6** | **5** | **15** | **17** | **10** | **8** | **20** | **13** | **12** | **14** | **26** | **16** | **19** | **21** | **9** | **29** | **18** | **23** | **27** | **30** | **25** | **22** | **24** | **32** | **31** | **28** |
|  | Canada | 1 | 3 | 2 | 4 | 11 | 9 | 6 | 5 | 12 | 17 | 10 | 8 | 19 | 14 | 13 | 16 | 25 | 15 | 20 | 21 | 7 | 30 | 18 | 24 | 27 | 31 | 22 | 26 | 23 | 32 | 28 | 29 |
|  | Greenland | 4 | 6 | 5 | 2 | 7 | 1 | 3 | 8 | 12 | 24 | 17 | 15 | 20 | 11 | 19 | 10 | 9 | 13 | 14 | 23 | 18 | 21 | 16 | 27 | 25 | 30 | 29 | 32 | 26 | 22 | 28 | 31 |
|  | United States of America | 1 | 2 | 3 | 4 | 11 | 7 | 6 | 5 | 15 | 17 | 10 | 8 | 20 | 12 | 13 | 14 | 26 | 16 | 19 | 22 | 9 | 29 | 18 | 23 | 27 | 30 | 25 | 21 | 24 | 32 | 31 | 28 |
| **Southern Latin America** | | **2** | **3** | **6** | **4** | **9** | **1** | **8** | **7** | **21** | **12** | **10** | **11** | **20** | **13** | **16** | **14** | **29** | **5** | **19** | **22** | **17** | **31** | **15** | **23** | **18** | **28** | **26** | **24** | **25** | **32** | **27** | **30** |
|  | Argentina | 3 | 2 | 5 | 4 | 10 | 1 | 8 | 6 | 21 | 12 | 9 | 11 | 20 | 13 | 16 | 14 | 29 | 7 | 18 | 22 | 17 | 31 | 15 | 23 | 19 | 27 | 25 | 24 | 26 | 32 | 28 | 30 |
|  | Chile | 1 | 4 | 6 | 5 | 7 | 3 | 11 | 8 | 19 | 12 | 9 | 10 | 22 | 13 | 17 | 18 | 28 | 2 | 24 | 20 | 16 | 31 | 14 | 21 | 15 | 30 | 25 | 29 | 23 | 32 | 26 | 27 |
|  | Uruguay | 4 | 1 | 6 | 5 | 9 | 2 | 7 | 3 | 20 | 10 | 13 | 11 | 18 | 15 | 14 | 16 | 24 | 8 | 21 | 19 | 17 | 31 | 12 | 25 | 22 | 26 | 27 | 28 | 23 | 32 | 29 | 30 |
| **Western Europe** | | **1** | **3** | **2** | **4** | **10** | **11** | **5** | **6** | **16** | **17** | **9** | **8** | **19** | **13** | **12** | **15** | **25** | **14** | **20** | **21** | **7** | **27** | **18** | **23** | **28** | **29** | **22** | **26** | **24** | **32** | **30** | **31** |
|  | Andorra | 2 | 3 | 1 | 6 | 10 | 8 | 4 | 5 | 12 | 18 | 9 | 7 | 21 | 17 | 14 | 13 | 26 | 15 | 19 | 20 | 11 | 28 | 16 | 24 | 22 | 29 | 27 | 25 | 23 | 32 | 30 | 31 |
|  | Austria | 1 | 3 | 2 | 8 | 12 | 11 | 5 | 7 | 15 | 18 | 9 | 6 | 19 | 14 | 16 | 13 | 30 | 10 | 20 | 21 | 4 | 23 | 17 | 26 | 27 | 29 | 25 | 22 | 24 | 32 | 31 | 28 |
|  | Belgium | 3 | 1 | 2 | 5 | 13 | 9 | 4 | 6 | 16 | 11 | 10 | 8 | 19 | 12 | 15 | 14 | 28 | 17 | 20 | 24 | 7 | 25 | 18 | 26 | 31 | 27 | 21 | 23 | 22 | 32 | 29 | 30 |
|  | Cyprus | 1 | 3 | 2 | 5 | 9 | 14 | 6 | 4 | 16 | 12 | 10 | 7 | 19 | 11 | 13 | 15 | 25 | 17 | 22 | 21 | 8 | 30 | 18 | 24 | 27 | 28 | 20 | 29 | 23 | 31 | 32 | 26 |
|  | Denmark | 2 | 3 | 1 | 4 | 15 | 9 | 5 | 8 | 16 | 18 | 11 | 7 | 20 | 12 | 13 | 14 | 29 | 10 | 19 | 21 | 6 | 25 | 17 | 23 | 27 | 30 | 22 | 26 | 24 | 32 | 28 | 31 |
|  | Finland | 2 | 3 | 1 | 5 | 10 | 16 | 8 | 4 | 14 | 19 | 9 | 7 | 18 | 11 | 12 | 13 | 30 | 15 | 23 | 22 | 6 | 27 | 17 | 20 | 24 | 31 | 26 | 25 | 21 | 32 | 29 | 28 |
|  | France | 1 | 2 | 3 | 5 | 13 | 10 | 4 | 7 | 15 | 12 | 9 | 8 | 19 | 16 | 14 | 17 | 26 | 11 | 20 | 22 | 6 | 23 | 18 | 27 | 30 | 28 | 21 | 24 | 25 | 32 | 29 | 31 |
|  | Germany | 2 | 3 | 1 | 4 | 9 | 11 | 5 | 7 | 16 | 19 | 10 | 6 | 18 | 14 | 15 | 13 | 29 | 12 | 20 | 21 | 8 | 24 | 17 | 22 | 23 | 30 | 25 | 27 | 26 | 28 | 32 | 31 |
|  | Greece | 3 | 2 | 1 | 5 | 8 | 12 | 4 | 7 | 18 | 15 | 11 | 9 | 19 | 16 | 6 | 13 | 20 | 14 | 22 | 24 | 10 | 32 | 17 | 29 | 27 | 26 | 23 | 21 | 25 | 30 | 28 | 31 |
|  | Iceland | 2 | 3 | 1 | 8 | 10 | 14 | 4 | 6 | 16 | 17 | 12 | 5 | 20 | 9 | 15 | 11 | 26 | 21 | 19 | 23 | 7 | 30 | 13 | 18 | 28 | 32 | 24 | 25 | 22 | 31 | 27 | 29 |
|  | Ireland | 3 | 2 | 1 | 4 | 12 | 8 | 7 | 6 | 18 | 20 | 10 | 9 | 19 | 11 | 13 | 14 | 26 | 15 | 17 | 23 | 5 | 29 | 16 | 24 | 28 | 31 | 21 | 25 | 22 | 32 | 27 | 30 |
|  | Israel | 3 | 1 | 2 | 5 | 10 | 12 | 7 | 4 | 16 | 15 | 9 | 6 | 19 | 13 | 11 | 14 | 25 | 18 | 22 | 20 | 8 | 32 | 17 | 21 | 30 | 29 | 24 | 27 | 23 | 26 | 28 | 31 |
|  | Italy | 1 | 2 | 3 | 5 | 10 | 14 | 7 | 4 | 18 | 16 | 6 | 9 | 19 | 13 | 11 | 15 | 21 | 12 | 24 | 20 | 8 | 29 | 17 | 22 | 27 | 30 | 26 | 23 | 25 | 32 | 28 | 31 |
|  | Luxembourg | 2 | 3 | 1 | 7 | 12 | 15 | 4 | 5 | 17 | 9 | 14 | 8 | 18 | 10 | 16 | 13 | 23 | 11 | 19 | 26 | 6 | 24 | 20 | 22 | 30 | 28 | 25 | 21 | 27 | 32 | 29 | 31 |
|  | Malta | 1 | 2 | 3 | 7 | 13 | 15 | 5 | 4 | 18 | 20 | 8 | 6 | 19 | 12 | 10 | 14 | 17 | 11 | 21 | 22 | 9 | 29 | 16 | 23 | 31 | 27 | 24 | 25 | 26 | 32 | 28 | 30 |
|  | Monaco | 5 | 3 | 4 | 7 | 16 | 17 | 2 | 1 | 15 | 6 | 14 | 8 | 25 | 12 | 10 | 13 | 26 | 9 | 20 | 22 | 11 | 30 | 18 | 24 | 29 | 23 | 21 | 31 | 19 | 32 | 28 | 27 |
|  | Netherlands | 1 | 2 | 3 | 6 | 11 | 10 | 5 | 7 | 16 | 15 | 9 | 8 | 20 | 12 | 14 | 17 | 27 | 13 | 19 | 23 | 4 | 29 | 18 | 22 | 24 | 31 | 21 | 26 | 25 | 32 | 28 | 30 |
|  | Norway | 2 | 3 | 1 | 5 | 14 | 9 | 7 | 6 | 12 | 18 | 15 | 8 | 20 | 10 | 16 | 13 | 29 | 11 | 22 | 25 | 4 | 30 | 17 | 21 | 27 | 31 | 23 | 26 | 19 | 32 | 24 | 28 |
|  | Portugal | 3 | 2 | 1 | 4 | 7 | 10 | 6 | 5 | 12 | 13 | 9 | 8 | 19 | 15 | 14 | 16 | 21 | 17 | 20 | 27 | 11 | 22 | 18 | 28 | 29 | 24 | 25 | 23 | 26 | 32 | 31 | 30 |
|  | San Marino | 1 | 3 | 2 | 8 | 7 | 12 | 6 | 4 | 16 | 10 | 5 | 13 | 19 | 15 | 11 | 14 | 20 | 18 | 25 | 24 | 9 | 29 | 17 | 21 | 26 | 28 | 22 | 31 | 23 | 32 | 30 | 27 |
|  | Spain | 1 | 2 | 3 | 5 | 8 | 13 | 4 | 6 | 16 | 12 | 9 | 7 | 19 | 15 | 14 | 11 | 22 | 18 | 20 | 23 | 10 | 27 | 17 | 24 | 30 | 28 | 21 | 25 | 26 | 32 | 29 | 31 |
|  | Sweden | 2 | 3 | 1 | 5 | 15 | 9 | 7 | 8 | 13 | 18 | 11 | 6 | 19 | 10 | 16 | 12 | 29 | 14 | 21 | 22 | 4 | 30 | 17 | 25 | 20 | 32 | 24 | 27 | 23 | 31 | 26 | 28 |
|  | Switzerland | 1 | 3 | 2 | 5 | 11 | 13 | 4 | 7 | 14 | 18 | 9 | 8 | 19 | 15 | 16 | 12 | 26 | 10 | 20 | 21 | 6 | 25 | 17 | 23 | 27 | 31 | 24 | 29 | 22 | 32 | 30 | 28 |
|  | United Kingdom | 2 | 1 | 3 | 4 | 13 | 8 | 7 | 5 | 14 | 20 | 10 | 9 | 19 | 11 | 12 | 15 | 23 | 18 | 17 | 22 | 6 | 26 | 16 | 25 | 29 | 30 | 21 | 27 | 24 | 32 | 28 | 31 |
| **Andean Latin America** | | **2** | **7** | **4** | **9** | **1** | **3** | **10** | **5** | **14** | **8** | **6** | **11** | **23** | **13** | **17** | **15** | **31** | **12** | **24** | **19** | **20** | **32** | **16** | **21** | **18** | **28** | **26** | **22** | **25** | **29** | **30** | **27** |
|  | Bolivia (Plurinational State of) | 3 | 5 | 4 | 9 | 1 | 2 | 11 | 8 | 14 | 6 | 7 | 10 | 22 | 13 | 15 | 17 | 30 | 12 | 24 | 18 | 16 | 31 | 20 | 21 | 19 | 27 | 26 | 23 | 25 | 32 | 28 | 29 |
|  | Ecuador | 2 | 8 | 6 | 7 | 1 | 4 | 11 | 5 | 17 | 10 | 3 | 9 | 23 | 13 | 14 | 16 | 29 | 12 | 24 | 18 | 19 | 32 | 15 | 20 | 22 | 28 | 26 | 21 | 25 | 31 | 30 | 27 |
|  | Peru | 2 | 7 | 3 | 9 | 1 | 4 | 10 | 5 | 14 | 6 | 8 | 11 | 23 | 12 | 17 | 15 | 31 | 13 | 24 | 19 | 20 | 32 | 16 | 22 | 18 | 29 | 28 | 21 | 25 | 27 | 30 | 26 |
| **Caribbean** | | **3** | **2** | **8** | **5** | **7** | **1** | **9** | **4** | **14** | **6** | **11** | **10** | **19** | **13** | **12** | **17** | **23** | **22** | **20** | **18** | **21** | **29** | **16** | **24** | **25** | **26** | **28** | **15** | **27** | **32** | **31** | **30** |
|  | Antigua and Barbuda | 2 | 1 | 6 | 5 | 9 | 4 | 11 | 3 | 15 | 8 | 12 | 7 | 22 | 10 | 21 | 14 | 26 | 18 | 23 | 19 | 16 | 29 | 13 | 25 | 28 | 30 | 27 | 17 | 24 | 32 | 31 | 20 |
|  | Bahamas | 2 | 1 | 6 | 5 | 8 | 3 | 9 | 4 | 14 | 13 | 12 | 7 | 20 | 10 | 16 | 17 | 24 | 32 | 19 | 21 | 18 | 26 | 11 | 25 | 27 | 28 | 30 | 15 | 22 | 31 | 29 | 23 |
|  | Barbados | 3 | 1 | 7 | 5 | 8 | 4 | 12 | 2 | 17 | 9 | 15 | 6 | 21 | 10 | 16 | 13 | 24 | 26 | 20 | 18 | 22 | 27 | 11 | 25 | 28 | 31 | 29 | 14 | 19 | 32 | 30 | 23 |
|  | Belize | 2 | 4 | 8 | 7 | 6 | 1 | 9 | 5 | 14 | 3 | 11 | 17 | 20 | 15 | 10 | 13 | 24 | 18 | 22 | 23 | 19 | 30 | 12 | 28 | 26 | 29 | 27 | 16 | 31 | 32 | 21 | 25 |
|  | Bermuda | 2 | 1 | 5 | 4 | 15 | 9 | 6 | 3 | 18 | 12 | 16 | 7 | 22 | 8 | 14 | 11 | 26 | 28 | 19 | 21 | 10 | 29 | 13 | 23 | 31 | 30 | 24 | 17 | 25 | 32 | 20 | 27 |
|  | Cuba | 1 | 5 | 2 | 6 | 13 | 3 | 8 | 4 | 21 | 17 | 9 | 7 | 20 | 12 | 10 | 15 | 25 | 18 | 22 | 16 | 19 | 28 | 14 | 24 | 30 | 23 | 26 | 11 | 27 | 32 | 29 | 31 |
|  | Dominica | 2 | 4 | 11 | 7 | 5 | 3 | 10 | 1 | 16 | 8 | 6 | 9 | 18 | 15 | 12 | 14 | 24 | 25 | 21 | 19 | 23 | 27 | 13 | 26 | 29 | 30 | 28 | 17 | 20 | 32 | 31 | 22 |
|  | Dominican Republic | 1 | 3 | 13 | 4 | 7 | 2 | 6 | 5 | 9 | 8 | 12 | 10 | 15 | 17 | 23 | 19 | 26 | 29 | 18 | 25 | 28 | 22 | 11 | 21 | 16 | 20 | 30 | 14 | 27 | 32 | 31 | 24 |
|  | Grenada | 4 | 3 | 6 | 5 | 9 | 2 | 11 | 1 | 17 | 7 | 14 | 10 | 20 | 8 | 16 | 13 | 24 | 26 | 18 | 19 | 21 | 30 | 15 | 23 | 28 | 32 | 27 | 12 | 25 | 31 | 29 | 22 |
|  | Guyana | 2 | 3 | 10 | 4 | 8 | 1 | 11 | 5 | 17 | 9 | 6 | 19 | 20 | 7 | 12 | 15 | 26 | 18 | 25 | 16 | 21 | 31 | 13 | 23 | 27 | 30 | 28 | 14 | 29 | 32 | 24 | 22 |
|  | Haiti | 3 | 2 | 7 | 8 | 4 | 1 | 11 | 6 | 15 | 5 | 10 | 9 | 19 | 13 | 12 | 22 | 20 | 25 | 21 | 16 | 17 | 31 | 18 | 23 | 24 | 29 | 28 | 14 | 26 | 32 | 27 | 30 |
|  | Jamaica | 4 | 1 | 8 | 5 | 9 | 2 | 7 | 3 | 13 | 6 | 12 | 11 | 22 | 10 | 17 | 18 | 15 | 26 | 21 | 16 | 25 | 27 | 14 | 20 | 24 | 29 | 28 | 19 | 23 | 32 | 30 | 31 |
|  | Puerto Rico | 2 | 1 | 6 | 4 | 14 | 5 | 8 | 3 | 19 | 11 | 10 | 7 | 21 | 12 | 9 | 16 | 26 | 13 | 23 | 22 | 18 | 29 | 15 | 24 | 30 | 27 | 25 | 17 | 20 | 32 | 31 | 28 |
|  | Saint Kitts and Nevis | 1 | 2 | 7 | 5 | 8 | 3 | 13 | 4 | 15 | 6 | 11 | 16 | 20 | 9 | 26 | 14 | 19 | 10 | 21 | 17 | 27 | 32 | 12 | 22 | 30 | 31 | 28 | 18 | 25 | 29 | 24 | 23 |
|  | Saint Lucia | 4 | 3 | 7 | 6 | 5 | 2 | 10 | 1 | 21 | 9 | 12 | 8 | 18 | 11 | 13 | 14 | 24 | 15 | 22 | 19 | 20 | 29 | 16 | 26 | 30 | 28 | 27 | 17 | 23 | 31 | 32 | 25 |
|  | Saint Vincent and the Grenadines | 4 | 3 | 8 | 5 | 7 | 1 | 11 | 2 | 17 | 6 | 13 | 9 | 18 | 10 | 12 | 15 | 22 | 23 | 25 | 19 | 20 | 27 | 16 | 21 | 31 | 26 | 28 | 14 | 29 | 30 | 32 | 24 |
|  | Suriname | 2 | 3 | 5 | 6 | 10 | 1 | 8 | 4 | 16 | 7 | 14 | 11 | 22 | 9 | 12 | 13 | 18 | 17 | 27 | 20 | 21 | 30 | 15 | 23 | 26 | 31 | 29 | 19 | 24 | 32 | 28 | 25 |
|  | Trinidad and Tobago | 2 | 1 | 8 | 5 | 13 | 4 | 7 | 3 | 16 | 12 | 10 | 6 | 19 | 9 | 14 | 15 | 23 | 17 | 21 | 18 | 28 | 29 | 11 | 24 | 25 | 30 | 22 | 20 | 27 | 32 | 31 | 26 |
|  | United States Virgin Islands | 4 | 1 | 5 | 2 | 10 | 6 | 7 | 3 | 15 | 11 | 17 | 8 | 20 | 9 | 19 | 13 | 22 | 32 | 21 | 25 | 14 | 29 | 12 | 26 | 27 | 30 | 31 | 16 | 18 | 28 | 23 | 24 |
| **Central Latin America** | | **1** | **5** | **7** | **6** | **4** | **2** | **12** | **8** | **16** | **11** | **3** | **9** | **20** | **13** | **14** | **15** | **28** | **10** | **21** | **19** | **18** | **32** | **17** | **22** | **23** | **27** | **26** | **24** | **25** | **31** | **30** | **29** |
|  | Colombia | 1 | 6 | 5 | 7 | 2 | 3 | 10 | 8 | 17 | 11 | 4 | 9 | 22 | 13 | 14 | 15 | 29 | 12 | 21 | 19 | 16 | 32 | 18 | 23 | 20 | 28 | 26 | 25 | 24 | 31 | 30 | 27 |
|  | Costa Rica | 1 | 7 | 8 | 3 | 2 | 6 | 13 | 4 | 12 | 15 | 5 | 9 | 21 | 14 | 10 | 17 | 19 | 11 | 24 | 20 | 18 | 28 | 16 | 22 | 23 | 29 | 27 | 26 | 25 | 32 | 31 | 30 |
|  | El Salvador | 4 | 7 | 5 | 6 | 3 | 2 | 10 | 8 | 17 | 1 | 9 | 12 | 18 | 11 | 13 | 14 | 26 | 15 | 20 | 23 | 25 | 30 | 16 | 22 | 19 | 24 | 28 | 21 | 27 | 31 | 32 | 29 |
|  | Guatemala | 4 | 8 | 7 | 6 | 1 | 2 | 9 | 10 | 13 | 3 | 5 | 12 | 19 | 14 | 16 | 17 | 27 | 11 | 18 | 26 | 23 | 30 | 15 | 22 | 21 | 25 | 28 | 24 | 29 | 32 | 31 | 20 |
|  | Honduras | 1 | 8 | 6 | 11 | 3 | 2 | 4 | 12 | 9 | 7 | 5 | 10 | 17 | 13 | 21 | 19 | 31 | 18 | 27 | 16 | 25 | 26 | 20 | 14 | 22 | 24 | 29 | 15 | 23 | 32 | 28 | 30 |
|  | Mexico | 1 | 4 | 8 | 6 | 7 | 3 | 13 | 9 | 17 | 12 | 2 | 10 | 20 | 11 | 14 | 15 | 29 | 5 | 21 | 19 | 18 | 32 | 16 | 22 | 23 | 28 | 26 | 25 | 24 | 31 | 27 | 30 |
|  | Nicaragua | 3 | 6 | 5 | 7 | 4 | 1 | 11 | 8 | 14 | 9 | 2 | 10 | 19 | 12 | 16 | 15 | 21 | 13 | 26 | 18 | 23 | 32 | 17 | 22 | 20 | 28 | 27 | 25 | 29 | 24 | 31 | 30 |
|  | Panama | 1 | 5 | 4 | 7 | 6 | 2 | 11 | 9 | 14 | 10 | 3 | 8 | 19 | 12 | 18 | 15 | 24 | 13 | 22 | 17 | 21 | 29 | 16 | 20 | 25 | 27 | 28 | 26 | 23 | 31 | 32 | 30 |
|  | Venezuela (Bolivarian Republic of) | 3 | 2 | 10 | 5 | 4 | 1 | 9 | 7 | 18 | 13 | 6 | 8 | 19 | 11 | 12 | 16 | 25 | 15 | 22 | 17 | 20 | 30 | 14 | 23 | 24 | 26 | 29 | 21 | 27 | 32 | 31 | 28 |
| **Tropical Latin America** | | **1** | **3** | **2** | **5** | **6** | **4** | **9** | **7** | **19** | **12** | **10** | **8** | **20** | **11** | **15** | **14** | **26** | **13** | **17** | **22** | **16** | **23** | **18** | **27** | **21** | **24** | **30** | **29** | **28** | **32** | **25** | **31** |
|  | Brazil | 1 | 3 | 2 | 5 | 6 | 4 | 9 | 7 | 19 | 12 | 10 | 8 | 20 | 11 | 15 | 14 | 26 | 13 | 17 | 22 | 16 | 23 | 18 | 28 | 21 | 25 | 30 | 29 | 27 | 32 | 24 | 31 |
|  | Paraguay | 2 | 3 | 7 | 4 | 9 | 1 | 11 | 5 | 20 | 10 | 6 | 8 | 18 | 13 | 14 | 15 | 29 | 12 | 21 | 19 | 17 | 24 | 16 | 22 | 25 | 26 | 30 | 23 | 28 | 32 | 31 | 27 |
| **North Africa and Middle East** | | **1** | **2** | **3** | **6** | **5** | **13** | **7** | **4** | **11** | **9** | **10** | **8** | **24** | **14** | **12** | **15** | **20** | **21** | **18** | **16** | **25** | **31** | **19** | **22** | **26** | **23** | **17** | **28** | **27** | **30** | **32** | **29** |
|  | Afghanistan | 1 | 6 | 5 | 10 | 2 | 11 | 14 | 3 | 7 | 4 | 8 | 9 | 21 | 16 | 12 | 23 | 19 | 26 | 15 | 13 | 20 | 30 | 25 | 18 | 22 | 17 | 24 | 28 | 27 | 29 | 32 | 31 |
|  | Algeria | 1 | 2 | 4 | 6 | 11 | 5 | 10 | 3 | 15 | 12 | 19 | 9 | 21 | 14 | 7 | 16 | 8 | 22 | 27 | 13 | 23 | 26 | 20 | 18 | 17 | 24 | 28 | 30 | 25 | 31 | 32 | 29 |
|  | Bahrain | 2 | 1 | 5 | 4 | 8 | 16 | 7 | 3 | 15 | 12 | 13 | 6 | 18 | 11 | 14 | 10 | 19 | 31 | 21 | 9 | 25 | 28 | 17 | 24 | 26 | 29 | 22 | 27 | 20 | 30 | 32 | 23 |
|  | Egypt | 1 | 2 | 3 | 6 | 12 | 18 | 7 | 5 | 4 | 11 | 10 | 9 | 24 | 15 | 16 | 13 | 26 | 23 | 20 | 14 | 29 | 31 | 17 | 21 | 22 | 19 | 8 | 25 | 27 | 28 | 32 | 30 |
|  | Iran (Islamic Republic of) | 3 | 2 | 1 | 7 | 4 | 17 | 6 | 8 | 12 | 10 | 9 | 5 | 22 | 13 | 11 | 15 | 30 | 20 | 16 | 14 | 21 | 31 | 18 | 23 | 26 | 19 | 24 | 28 | 25 | 27 | 32 | 29 |
|  | Iraq | 2 | 3 | 1 | 6 | 8 | 15 | 7 | 5 | 11 | 4 | 12 | 9 | 19 | 10 | 13 | 14 | 26 | 18 | 23 | 16 | 28 | 30 | 17 | 22 | 27 | 21 | 20 | 25 | 24 | 32 | 29 | 31 |
|  | Jordan | 2 | 3 | 5 | 6 | 8 | 13 | 7 | 4 | 15 | 1 | 17 | 12 | 14 | 9 | 18 | 11 | 19 | 10 | 21 | 26 | 24 | 31 | 16 | 20 | 23 | 28 | 22 | 25 | 27 | 32 | 30 | 29 |
|  | Kuwait | 2 | 1 | 3 | 4 | 11 | 13 | 7 | 5 | 14 | 6 | 10 | 8 | 18 | 9 | 16 | 12 | 27 | 28 | 19 | 20 | 26 | 30 | 15 | 22 | 24 | 29 | 17 | 23 | 25 | 32 | 31 | 21 |
|  | Lebanon | 1 | 2 | 3 | 4 | 10 | 14 | 5 | 8 | 16 | 11 | 9 | 7 | 21 | 12 | 6 | 15 | 25 | 17 | 28 | 13 | 20 | 31 | 22 | 18 | 26 | 23 | 19 | 27 | 24 | 30 | 32 | 29 |
|  | Libya | 1 | 2 | 3 | 4 | 11 | 14 | 7 | 6 | 13 | 10 | 12 | 8 | 20 | 16 | 5 | 15 | 9 | 24 | 27 | 17 | 26 | 31 | 19 | 21 | 23 | 18 | 22 | 28 | 25 | 29 | 32 | 30 |
|  | Morocco | 2 | 1 | 4 | 5 | 12 | 6 | 7 | 3 | 20 | 13 | 14 | 11 | 16 | 9 | 8 | 17 | 10 | 22 | 24 | 19 | 23 | 27 | 21 | 15 | 26 | 18 | 29 | 28 | 25 | 31 | 32 | 30 |
|  | Oman | 1 | 4 | 3 | 6 | 7 | 15 | 11 | 2 | 8 | 12 | 9 | 5 | 19 | 14 | 10 | 13 | 23 | 26 | 21 | 16 | 25 | 31 | 18 | 20 | 28 | 30 | 27 | 32 | 22 | 29 | 17 | 24 |
|  | Palestine | 2 | 3 | 1 | 6 | 8 | 16 | 5 | 7 | 10 | 4 | 13 | 11 | 19 | 12 | 9 | 14 | 23 | 28 | 24 | 15 | 25 | 31 | 17 | 20 | 26 | 30 | 22 | 18 | 21 | 27 | 32 | 29 |
|  | Qatar | 1 | 2 | 3 | 6 | 8 | 16 | 7 | 4 | 9 | 13 | 11 | 5 | 19 | 17 | 14 | 12 | 23 | 21 | 18 | 10 | 26 | 31 | 15 | 27 | 25 | 24 | 28 | 32 | 22 | 29 | 30 | 20 |
|  | Saudi Arabia | 1 | 2 | 4 | 5 | 10 | 16 | 6 | 3 | 15 | 12 | 8 | 7 | 20 | 13 | 14 | 11 | 17 | 22 | 21 | 9 | 27 | 30 | 18 | 19 | 23 | 28 | 26 | 29 | 25 | 31 | 32 | 24 |
|  | Sudan | 1 | 3 | 2 | 9 | 4 | 12 | 10 | 8 | 14 | 5 | 7 | 6 | 20 | 16 | 11 | 17 | 24 | 23 | 15 | 13 | 22 | 31 | 18 | 21 | 26 | 19 | 25 | 29 | 27 | 28 | 32 | 30 |
|  | Syrian Arab Republic | 4 | 3 | 2 | 8 | 10 | 15 | 5 | 9 | 11 | 1 | 7 | 6 | 20 | 13 | 17 | 14 | 25 | 23 | 22 | 12 | 27 | 30 | 18 | 29 | 31 | 21 | 19 | 16 | 24 | 26 | 32 | 28 |
|  | Tunisia | 2 | 1 | 6 | 4 | 8 | 11 | 3 | 9 | 17 | 15 | 12 | 5 | 16 | 13 | 10 | 14 | 7 | 26 | 27 | 20 | 22 | 28 | 21 | 18 | 23 | 19 | 24 | 31 | 25 | 29 | 32 | 30 |
|  | Turkey | 1 | 3 | 2 | 4 | 6 | 16 | 5 | 7 | 17 | 12 | 9 | 8 | 21 | 13 | 14 | 11 | 20 | 10 | 18 | 24 | 19 | 32 | 15 | 26 | 28 | 23 | 25 | 29 | 27 | 30 | 22 | 31 |
|  | United Arab Emirates | 1 | 4 | 2 | 6 | 13 | 23 | 10 | 3 | 11 | 16 | 14 | 8 | 19 | 15 | 12 | 5 | 27 | 24 | 7 | 20 | 25 | 29 | 9 | 22 | 26 | 17 | 18 | 30 | 21 | 31 | 32 | 28 |
|  | Yemen | 2 | 3 | 4 | 9 | 1 | 13 | 10 | 8 | 15 | 5 | 7 | 6 | 19 | 17 | 11 | 18 | 22 | 25 | 14 | 12 | 20 | 31 | 24 | 21 | 23 | 16 | 26 | 29 | 27 | 28 | 32 | 30 |
| **South Asia** | | **1** | **2** | **6** | **8** | **3** | **5** | **9** | **7** | **15** | **20** | **16** | **12** | **4** | **14** | **11** | **23** | **19** | **17** | **18** | **13** | **26** | **10** | **25** | **21** | **24** | **22** | **27** | **29** | **28** | **32** | **30** | **31** |
|  | Bangladesh | 1 | 2 | 3 | 12 | 5 | 4 | 10 | 6 | 8 | 20 | 13 | 9 | 7 | 15 | 11 | 22 | 17 | 19 | 18 | 16 | 26 | 14 | 25 | 21 | 24 | 23 | 28 | 31 | 27 | 32 | 30 | 29 |
|  | Bhutan | 1 | 2 | 3 | 8 | 6 | 4 | 10 | 5 | 16 | 20 | 15 | 9 | 7 | 11 | 12 | 22 | 19 | 18 | 17 | 14 | 27 | 13 | 25 | 21 | 23 | 24 | 28 | 30 | 26 | 32 | 31 | 29 |
|  | India | 1 | 2 | 5 | 8 | 3 | 4 | 10 | 7 | 14 | 20 | 15 | 11 | 6 | 16 | 13 | 23 | 17 | 19 | 18 | 12 | 27 | 9 | 25 | 21 | 24 | 22 | 28 | 30 | 26 | 32 | 29 | 31 |
|  | Nepal | 1 | 3 | 5 | 11 | 4 | 2 | 10 | 6 | 16 | 19 | 13 | 8 | 7 | 9 | 12 | 22 | 18 | 21 | 17 | 14 | 26 | 15 | 25 | 20 | 23 | 24 | 28 | 29 | 27 | 32 | 30 | 31 |
|  | Pakistan | 1 | 2 | 5 | 8 | 11 | 13 | 9 | 7 | 16 | 19 | 14 | 12 | 3 | 6 | 4 | 26 | 20 | 10 | 15 | 18 | 25 | 21 | 28 | 17 | 23 | 22 | 24 | 27 | 29 | 30 | 32 | 31 |
| **East Asia** | | **7** | **8** | **5** | **3** | **4** | **11** | **2** | **9** | **1** | **6** | **10** | **15** | **18** | **16** | **26** | **12** | **13** | **25** | **14** | **29** | **24** | **32** | **17** | **27** | **20** | **28** | **21** | **22** | **23** | **19** | **30** | **31** |
|  | China | 7 | 8 | 5 | 3 | 4 | 11 | 2 | 9 | 1 | 6 | 10 | 15 | 18 | 16 | 26 | 12 | 13 | 25 | 14 | 29 | 24 | 32 | 17 | 27 | 20 | 28 | 21 | 22 | 23 | 19 | 30 | 31 |
|  | Democratic People's Republic of Korea | 5 | 8 | 7 | 6 | 2 | 9 | 4 | 10 | 3 | 1 | 11 | 15 | 18 | 16 | 21 | 13 | 12 | 25 | 14 | 27 | 23 | 32 | 17 | 24 | 20 | 29 | 22 | 19 | 28 | 26 | 30 | 31 |
|  | Taiwan (Province of China) | 4 | 3 | 7 | 1 | 6 | 15 | 2 | 8 | 10 | 13 | 17 | 12 | 5 | 16 | 27 | 14 | 9 | 20 | 11 | 26 | 22 | 18 | 19 | 25 | 21 | 30 | 24 | 23 | 29 | 32 | 28 | 31 |
| **Oceania** | | **2** | **1** | **9** | **7** | **3** | **4** | **5** | **10** | **11** | **6** | **12** | **8** | **13** | **14** | **15** | **21** | **18** | **19** | **20** | **17** | **25** | **28** | **27** | **23** | **29** | **31** | **22** | **16** | **26** | **32** | **30** | **24** |
|  | American Samoa | 1 | 2 | 9 | 4 | 3 | 7 | 5 | 6 | 8 | 12 | 14 | 11 | 21 | 10 | 20 | 16 | 17 | 31 | 26 | 19 | 24 | 27 | 23 | 22 | 29 | 32 | 18 | 13 | 25 | 30 | 28 | 15 |
|  | Cook Islands | 2 | 1 | 6 | 8 | 5 | 10 | 3 | 7 | 4 | 11 | 17 | 9 | 14 | 13 | 19 | 12 | 27 | 24 | 18 | 22 | 25 | 29 | 20 | 21 | 28 | 31 | 16 | 23 | 26 | 32 | 30 | 15 |
|  | Fiji | 3 | 1 | 9 | 5 | 7 | 2 | 10 | 6 | 8 | 11 | 13 | 4 | 14 | 20 | 15 | 17 | 28 | 12 | 21 | 18 | 24 | 27 | 23 | 19 | 29 | 31 | 22 | 16 | 26 | 32 | 30 | 25 |
|  | Guam | 5 | 3 | 11 | 2 | 6 | 10 | 1 | 4 | 7 | 9 | 17 | 8 | 15 | 13 | 23 | 14 | 12 | 28 | 16 | 21 | 27 | 24 | 18 | 20 | 30 | 31 | 19 | 22 | 26 | 32 | 29 | 25 |
|  | Kiribati | 2 | 3 | 13 | 10 | 4 | 1 | 9 | 11 | 5 | 8 | 16 | 14 | 6 | 18 | 15 | 23 | 20 | 7 | 12 | 22 | 27 | 21 | 17 | 29 | 26 | 30 | 25 | 19 | 28 | 32 | 31 | 24 |
|  | Marshall Islands | 4 | 1 | 11 | 6 | 2 | 3 | 5 | 9 | 7 | 8 | 12 | 10 | 14 | 13 | 18 | 19 | 17 | 27 | 20 | 16 | 25 | 28 | 24 | 22 | 29 | 31 | 21 | 15 | 26 | 32 | 30 | 23 |
|  | Micronesia (Federated States of) | 3 | 1 | 11 | 6 | 2 | 4 | 5 | 9 | 8 | 7 | 12 | 10 | 14 | 13 | 18 | 17 | 19 | 27 | 20 | 15 | 25 | 28 | 24 | 21 | 29 | 31 | 22 | 16 | 26 | 32 | 30 | 23 |
|  | Nauru | 3 | 1 | 11 | 6 | 2 | 5 | 4 | 7 | 10 | 9 | 12 | 8 | 13 | 14 | 20 | 16 | 18 | 27 | 19 | 15 | 26 | 28 | 23 | 22 | 29 | 30 | 21 | 17 | 25 | 32 | 31 | 24 |
|  | Niue | 2 | 1 | 10 | 4 | 6 | 5 | 3 | 7 | 9 | 11 | 13 | 8 | 15 | 12 | 21 | 14 | 18 | 27 | 19 | 17 | 25 | 28 | 20 | 23 | 29 | 31 | 22 | 16 | 26 | 32 | 30 | 24 |
|  | Northern Mariana Islands | 1 | 3 | 9 | 4 | 6 | 7 | 2 | 5 | 10 | 11 | 13 | 8 | 12 | 15 | 25 | 14 | 16 | 28 | 19 | 24 | 27 | 18 | 20 | 21 | 29 | 30 | 23 | 17 | 26 | 31 | 32 | 22 |
|  | Palau | 1 | 4 | 10 | 8 | 6 | 3 | 2 | 9 | 7 | 17 | 15 | 12 | 5 | 19 | 24 | 11 | 25 | 32 | 14 | 22 | 18 | 23 | 20 | 13 | 28 | 29 | 21 | 31 | 26 | 30 | 27 | 16 |
|  | Papua New Guinea | 2 | 1 | 9 | 7 | 3 | 4 | 5 | 12 | 22 | 6 | 10 | 8 | 11 | 13 | 14 | 19 | 15 | 25 | 18 | 17 | 24 | 28 | 27 | 23 | 29 | 31 | 20 | 16 | 26 | 32 | 30 | 21 |
|  | Samoa | 1 | 2 | 10 | 9 | 5 | 3 | 15 | 4 | 11 | 8 | 16 | 7 | 21 | 12 | 6 | 19 | 14 | 13 | 25 | 18 | 17 | 29 | 24 | 20 | 27 | 31 | 23 | 22 | 28 | 32 | 30 | 26 |
|  | Solomon Islands | 4 | 1 | 10 | 8 | 2 | 3 | 6 | 5 | 9 | 7 | 12 | 11 | 15 | 16 | 18 | 20 | 17 | 29 | 19 | 13 | 23 | 28 | 26 | 22 | 27 | 30 | 21 | 14 | 25 | 32 | 31 | 24 |
|  | Tokelau | 2 | 1 | 11 | 6 | 4 | 3 | 5 | 8 | 7 | 10 | 12 | 9 | 15 | 13 | 19 | 16 | 17 | 27 | 21 | 18 | 26 | 28 | 24 | 23 | 29 | 31 | 20 | 14 | 25 | 32 | 30 | 22 |
|  | Tonga | 5 | 2 | 10 | 11 | 7 | 6 | 8 | 4 | 3 | 12 | 14 | 9 | 15 | 13 | 17 | 16 | 19 | 1 | 21 | 25 | 26 | 28 | 24 | 27 | 29 | 31 | 23 | 18 | 22 | 32 | 30 | 20 |
|  | Tuvalu | 2 | 1 | 11 | 6 | 3 | 4 | 5 | 8 | 7 | 9 | 12 | 10 | 14 | 13 | 18 | 19 | 15 | 27 | 20 | 16 | 25 | 28 | 24 | 23 | 29 | 30 | 22 | 17 | 26 | 32 | 31 | 21 |
|  | Vanuatu | 3 | 1 | 11 | 8 | 2 | 4 | 5 | 9 | 7 | 6 | 12 | 10 | 13 | 15 | 14 | 20 | 16 | 27 | 19 | 17 | 24 | 28 | 25 | 23 | 29 | 31 | 21 | 18 | 26 | 32 | 30 | 22 |
| **Southeast Asia** | | **2** | **1** | **6** | **3** | **10** | **8** | **4** | **7** | **9** | **5** | **13** | **11** | **16** | **12** | **17** | **15** | **14** | **18** | **20** | **22** | **25** | **26** | **19** | **21** | **24** | **28** | **29** | **23** | **31** | **32** | **27** | **30** |
|  | Cambodia | 1 | 2 | 6 | 5 | 10 | 8 | 7 | 9 | 3 | 4 | 13 | 12 | 15 | 11 | 16 | 17 | 14 | 20 | 18 | 19 | 24 | 29 | 22 | 23 | 27 | 25 | 28 | 21 | 30 | 32 | 26 | 31 |
|  | Indonesia | 2 | 1 | 6 | 3 | 10 | 8 | 5 | 7 | 15 | 4 | 12 | 11 | 16 | 9 | 17 | 13 | 14 | 20 | 19 | 23 | 24 | 29 | 18 | 22 | 27 | 28 | 25 | 21 | 30 | 32 | 26 | 31 |
|  | Lao People's Democratic Republic | 2 | 1 | 6 | 4 | 9 | 7 | 5 | 8 | 10 | 3 | 13 | 12 | 17 | 11 | 15 | 16 | 14 | 22 | 18 | 20 | 24 | 29 | 21 | 23 | 26 | 28 | 25 | 19 | 30 | 32 | 27 | 31 |
|  | Malaysia | 2 | 1 | 8 | 3 | 12 | 10 | 9 | 4 | 11 | 13 | 6 | 7 | 15 | 14 | 18 | 19 | 5 | 17 | 22 | 16 | 23 | 27 | 21 | 20 | 28 | 24 | 25 | 26 | 30 | 32 | 29 | 31 |
|  | Maldives | 1 | 4 | 2 | 6 | 12 | 10 | 8 | 5 | 7 | 14 | 13 | 3 | 16 | 9 | 11 | 15 | 26 | 30 | 19 | 22 | 21 | 28 | 18 | 20 | 29 | 27 | 25 | 31 | 23 | 32 | 17 | 24 |
|  | Mauritius | 2 | 1 | 7 | 3 | 9 | 11 | 6 | 5 | 19 | 4 | 13 | 8 | 14 | 10 | 15 | 12 | 18 | 20 | 17 | 24 | 28 | 29 | 16 | 25 | 27 | 23 | 26 | 22 | 21 | 32 | 31 | 30 |
|  | Myanmar | 2 | 3 | 7 | 5 | 9 | 8 | 6 | 1 | 13 | 4 | 12 | 11 | 17 | 10 | 16 | 15 | 14 | 21 | 18 | 22 | 24 | 29 | 19 | 23 | 27 | 28 | 26 | 20 | 30 | 32 | 25 | 31 |
|  | Philippines | 1 | 2 | 8 | 3 | 12 | 9 | 5 | 6 | 7 | 4 | 13 | 11 | 16 | 10 | 22 | 15 | 14 | 19 | 23 | 18 | 24 | 29 | 17 | 21 | 27 | 26 | 25 | 20 | 31 | 32 | 28 | 30 |
|  | Seychelles | 5 | 1 | 4 | 2 | 10 | 6 | 7 | 3 | 15 | 11 | 8 | 13 | 14 | 9 | 18 | 16 | 17 | 20 | 19 | 12 | 24 | 23 | 25 | 32 | 31 | 21 | 27 | 28 | 29 | 26 | 30 | 22 |
|  | Sri Lanka | 1 | 2 | 3 | 9 | 10 | 12 | 7 | 4 | 15 | 5 | 8 | 6 | 14 | 11 | 13 | 18 | 19 | 21 | 16 | 20 | 28 | 24 | 17 | 22 | 23 | 29 | 30 | 26 | 27 | 32 | 25 | 31 |
|  | Thailand | 2 | 4 | 7 | 6 | 9 | 8 | 3 | 10 | 1 | 5 | 19 | 12 | 15 | 11 | 22 | 14 | 13 | 20 | 16 | 18 | 28 | 27 | 21 | 25 | 17 | 24 | 26 | 29 | 31 | 32 | 23 | 30 |
|  | Timor-Leste | 1 | 3 | 4 | 5 | 9 | 8 | 6 | 7 | 10 | 2 | 12 | 11 | 16 | 13 | 15 | 17 | 14 | 18 | 19 | 22 | 24 | 29 | 20 | 23 | 28 | 27 | 26 | 21 | 31 | 32 | 25 | 30 |
|  | Viet Nam | 1 | 2 | 7 | 3 | 5 | 6 | 4 | 10 | 19 | 15 | 8 | 11 | 12 | 13 | 16 | 18 | 9 | 17 | 23 | 22 | 25 | 20 | 21 | 14 | 27 | 24 | 28 | 31 | 29 | 32 | 26 | 30 |
| **Central Sub-Saharan Africa** | | **2** | **3** | **7** | **8** | **6** | **1** | **9** | **4** | **11** | **5** | **13** | **14** | **18** | **15** | **10** | **20** | **21** | **17** | **12** | **16** | **19** | **31** | **22** | **24** | **29** | **27** | **23** | **26** | **28** | **32** | **30** | **25** |
|  | Angola | 2 | 3 | 8 | 5 | 7 | 1 | 9 | 4 | 13 | 6 | 14 | 11 | 18 | 16 | 10 | 19 | 21 | 17 | 12 | 15 | 20 | 31 | 22 | 24 | 29 | 26 | 23 | 27 | 28 | 32 | 30 | 25 |
|  | Central African Republic | 2 | 3 | 7 | 8 | 4 | 1 | 9 | 6 | 12 | 5 | 13 | 15 | 16 | 17 | 10 | 21 | 20 | 18 | 11 | 14 | 19 | 31 | 24 | 23 | 27 | 25 | 22 | 26 | 29 | 32 | 30 | 28 |
|  | Congo | 3 | 2 | 7 | 5 | 6 | 1 | 8 | 4 | 12 | 9 | 16 | 13 | 18 | 11 | 14 | 15 | 22 | 19 | 10 | 17 | 20 | 31 | 21 | 25 | 29 | 28 | 23 | 24 | 27 | 32 | 30 | 26 |
|  | Democratic Republic of the Congo | 2 | 3 | 7 | 9 | 6 | 1 | 8 | 5 | 11 | 4 | 13 | 14 | 18 | 15 | 10 | 20 | 21 | 16 | 12 | 17 | 19 | 31 | 23 | 24 | 29 | 27 | 22 | 26 | 28 | 32 | 30 | 25 |
|  | Equatorial Guinea | 1 | 3 | 6 | 5 | 9 | 2 | 7 | 4 | 11 | 8 | 14 | 10 | 19 | 12 | 17 | 16 | 22 | 15 | 13 | 18 | 21 | 29 | 20 | 25 | 30 | 28 | 23 | 27 | 26 | 32 | 31 | 24 |
|  | Gabon | 1 | 2 | 7 | 5 | 8 | 3 | 6 | 4 | 12 | 9 | 15 | 11 | 19 | 10 | 16 | 14 | 23 | 18 | 13 | 17 | 21 | 29 | 20 | 25 | 30 | 28 | 22 | 27 | 26 | 32 | 31 | 24 |
| **Eastern Sub-Saharan Africa** | | **1** | **3** | **8** | **7** | **6** | **2** | **20** | **5** | **14** | **12** | **11** | **17** | **18** | **13** | **4** | **22** | **16** | **21** | **9** | **10** | **19** | **28** | **23** | **15** | **29** | **26** | **24** | **27** | **25** | **32** | **30** | **31** |
|  | Burundi | 1 | 3 | 7 | 8 | 4 | 2 | 18 | 6 | 11 | 9 | 17 | 20 | 14 | 13 | 5 | 22 | 12 | 21 | 10 | 15 | 16 | 27 | 24 | 19 | 29 | 23 | 25 | 28 | 26 | 32 | 30 | 31 |
|  | Comoros | 1 | 3 | 8 | 5 | 6 | 2 | 13 | 4 | 12 | 11 | 17 | 18 | 16 | 9 | 7 | 21 | 14 | 22 | 10 | 19 | 15 | 28 | 23 | 20 | 29 | 27 | 26 | 24 | 25 | 32 | 30 | 31 |
|  | Djibouti | 1 | 3 | 7 | 4 | 6 | 2 | 11 | 5 | 13 | 12 | 19 | 18 | 15 | 10 | 8 | 21 | 14 | 20 | 9 | 17 | 16 | 27 | 23 | 22 | 29 | 26 | 24 | 28 | 25 | 32 | 30 | 31 |
|  | Eritrea | 1 | 3 | 8 | 6 | 4 | 2 | 11 | 5 | 14 | 10 | 18 | 20 | 15 | 12 | 7 | 22 | 13 | 21 | 9 | 17 | 16 | 27 | 23 | 19 | 29 | 25 | 24 | 26 | 28 | 32 | 30 | 31 |
|  | Ethiopia | 1 | 3 | 6 | 9 | 8 | 2 | 19 | 13 | 16 | 15 | 5 | 11 | 17 | 12 | 7 | 23 | 14 | 18 | 21 | 4 | 20 | 30 | 22 | 10 | 24 | 26 | 25 | 27 | 28 | 32 | 29 | 31 |
|  | Kenya | 1 | 2 | 6 | 7 | 5 | 3 | 17 | 4 | 12 | 14 | 15 | 16 | 10 | 13 | 8 | 19 | 11 | 27 | 9 | 20 | 18 | 23 | 24 | 21 | 26 | 25 | 28 | 29 | 22 | 32 | 30 | 31 |
|  | Madagascar | 1 | 3 | 8 | 6 | 4 | 2 | 14 | 5 | 12 | 10 | 18 | 20 | 16 | 11 | 7 | 22 | 15 | 21 | 9 | 17 | 13 | 28 | 23 | 19 | 29 | 26 | 24 | 25 | 27 | 32 | 30 | 31 |
|  | Malawi | 1 | 3 | 8 | 9 | 15 | 2 | 18 | 5 | 13 | 14 | 12 | 10 | 17 | 11 | 7 | 22 | 24 | 20 | 4 | 23 | 6 | 32 | 21 | 16 | 30 | 28 | 19 | 26 | 25 | 31 | 27 | 29 |
|  | Mozambique | 1 | 3 | 6 | 8 | 11 | 2 | 14 | 5 | 10 | 7 | 15 | 17 | 22 | 9 | 4 | 20 | 30 | 18 | 19 | 13 | 12 | 28 | 21 | 16 | 27 | 23 | 24 | 26 | 25 | 32 | 29 | 31 |
|  | Rwanda | 1 | 3 | 8 | 5 | 6 | 2 | 16 | 4 | 9 | 12 | 18 | 19 | 15 | 10 | 7 | 22 | 14 | 21 | 11 | 17 | 13 | 27 | 23 | 20 | 29 | 26 | 25 | 28 | 24 | 32 | 30 | 31 |
|  | Somalia | 2 | 4 | 7 | 10 | 3 | 1 | 19 | 8 | 11 | 6 | 13 | 20 | 16 | 15 | 5 | 22 | 12 | 21 | 9 | 14 | 17 | 29 | 26 | 18 | 28 | 23 | 24 | 25 | 27 | 32 | 30 | 31 |
|  | South Sudan | 1 | 3 | 8 | 5 | 6 | 2 | 13 | 4 | 12 | 10 | 15 | 14 | 16 | 11 | 7 | 22 | 17 | 21 | 9 | 19 | 18 | 26 | 23 | 20 | 29 | 28 | 24 | 25 | 27 | 32 | 30 | 31 |
|  | Uganda | 1 | 3 | 10 | 4 | 7 | 2 | 16 | 6 | 8 | 13 | 22 | 21 | 15 | 11 | 5 | 18 | 12 | 20 | 9 | 19 | 14 | 23 | 24 | 17 | 29 | 27 | 28 | 25 | 26 | 32 | 31 | 30 |
|  | United Republic of Tanzania | 1 | 3 | 6 | 5 | 7 | 2 | 12 | 4 | 19 | 10 | 17 | 18 | 15 | 11 | 8 | 22 | 14 | 21 | 9 | 16 | 13 | 27 | 23 | 20 | 29 | 28 | 25 | 26 | 24 | 32 | 30 | 31 |
|  | Zambia | 1 | 3 | 8 | 4 | 6 | 2 | 12 | 5 | 18 | 11 | 17 | 19 | 13 | 10 | 7 | 21 | 15 | 22 | 9 | 14 | 16 | 28 | 23 | 20 | 29 | 26 | 24 | 25 | 27 | 32 | 30 | 31 |
| **Southern Sub-Saharan Africa** | | **3** | **2** | **8** | **6** | **10** | **1** | **7** | **4** | **5** | **9** | **17** | **13** | **18** | **12** | **15** | **14** | **23** | **19** | **11** | **29** | **16** | **30** | **21** | **24** | **31** | **26** | **20** | **28** | **22** | **25** | **32** | **27** |
|  | Botswana | 3 | 2 | 7 | 4 | 9 | 1 | 6 | 5 | 16 | 8 | 14 | 12 | 15 | 10 | 22 | 13 | 24 | 17 | 11 | 23 | 18 | 27 | 19 | 32 | 30 | 25 | 20 | 29 | 21 | 28 | 31 | 26 |
|  | Eswatini | 3 | 5 | 8 | 6 | 10 | 2 | 9 | 4 | 1 | 7 | 16 | 13 | 17 | 11 | 14 | 15 | 21 | 20 | 12 | 26 | 18 | 28 | 19 | 24 | 30 | 25 | 22 | 29 | 23 | 31 | 32 | 27 |
|  | Lesotho | 3 | 4 | 10 | 8 | 7 | 1 | 9 | 5 | 2 | 6 | 15 | 16 | 14 | 12 | 13 | 17 | 20 | 19 | 11 | 23 | 18 | 30 | 21 | 26 | 31 | 24 | 22 | 27 | 25 | 32 | 28 | 29 |
|  | Namibia | 1 | 2 | 5 | 8 | 16 | 3 | 13 | 4 | 11 | 9 | 17 | 15 | 7 | 14 | 10 | 18 | 19 | 12 | 24 | 27 | 6 | 23 | 21 | 22 | 32 | 20 | 26 | 28 | 25 | 31 | 29 | 30 |
|  | South Africa | 4 | 3 | 7 | 6 | 10 | 1 | 9 | 2 | 5 | 8 | 17 | 14 | 18 | 12 | 15 | 13 | 23 | 20 | 11 | 32 | 16 | 28 | 19 | 27 | 29 | 26 | 21 | 31 | 22 | 24 | 30 | 25 |
|  | Zimbabwe | 2 | 3 | 10 | 6 | 5 | 1 | 7 | 13 | 4 | 17 | 16 | 11 | 19 | 9 | 15 | 14 | 21 | 23 | 8 | 26 | 12 | 31 | 28 | 20 | 29 | 25 | 18 | 24 | 22 | 27 | 32 | 30 |
| **Western Sub-Saharan Africa** | | **3** | **2** | **4** | **8** | **7** | **1** | **11** | **5** | **9** | **10** | **13** | **12** | **19** | **14** | **6** | **16** | **17** | **23** | **21** | **15** | **18** | **31** | **22** | **26** | **28** | **25** | **20** | **29** | **27** | **32** | **30** | **24** |
|  | Benin | 2 | 3 | 6 | 9 | 5 | 1 | 11 | 4 | 7 | 8 | 12 | 10 | 18 | 15 | 14 | 16 | 22 | 23 | 17 | 13 | 21 | 30 | 20 | 25 | 27 | 26 | 19 | 28 | 29 | 32 | 31 | 24 |
|  | Burkina Faso | 3 | 2 | 6 | 8 | 4 | 1 | 9 | 5 | 11 | 7 | 12 | 10 | 18 | 14 | 15 | 16 | 21 | 23 | 17 | 13 | 20 | 30 | 22 | 24 | 28 | 26 | 19 | 27 | 29 | 32 | 31 | 25 |
|  | Cabo Verde | 2 | 8 | 3 | 10 | 6 | 5 | 7 | 4 | 1 | 9 | 14 | 12 | 19 | 15 | 23 | 11 | 20 | 25 | 13 | 16 | 22 | 26 | 18 | 24 | 30 | 27 | 17 | 29 | 28 | 31 | 32 | 21 |
|  | Cameroon | 3 | 2 | 7 | 6 | 5 | 1 | 9 | 4 | 20 | 8 | 13 | 10 | 19 | 12 | 16 | 11 | 14 | 21 | 17 | 15 | 22 | 30 | 23 | 26 | 28 | 25 | 18 | 29 | 27 | 32 | 31 | 24 |
|  | Chad | 2 | 3 | 8 | 9 | 4 | 1 | 10 | 5 | 7 | 6 | 12 | 11 | 19 | 15 | 13 | 17 | 21 | 22 | 16 | 14 | 20 | 29 | 23 | 25 | 27 | 26 | 18 | 28 | 30 | 32 | 31 | 24 |
|  | Côte d'Ivoire | 2 | 3 | 6 | 7 | 5 | 1 | 9 | 4 | 8 | 10 | 13 | 11 | 19 | 15 | 16 | 14 | 22 | 23 | 17 | 12 | 21 | 29 | 20 | 26 | 27 | 25 | 18 | 30 | 28 | 32 | 31 | 24 |
|  | Gambia | 2 | 4 | 6 | 5 | 11 | 3 | 13 | 9 | 1 | 7 | 12 | 8 | 17 | 14 | 10 | 18 | 23 | 16 | 21 | 15 | 19 | 32 | 20 | 22 | 26 | 28 | 24 | 27 | 29 | 30 | 31 | 25 |
|  | Ghana | 5 | 1 | 3 | 6 | 10 | 2 | 9 | 4 | 7 | 13 | 15 | 8 | 23 | 12 | 19 | 11 | 25 | 21 | 18 | 16 | 22 | 30 | 17 | 31 | 26 | 24 | 14 | 29 | 27 | 32 | 28 | 20 |
|  | Guinea | 3 | 4 | 10 | 7 | 5 | 1 | 9 | 14 | 2 | 12 | 15 | 13 | 8 | 11 | 6 | 21 | 22 | 18 | 23 | 17 | 16 | 28 | 24 | 20 | 29 | 27 | 19 | 25 | 30 | 32 | 31 | 26 |
|  | Guinea-Bissau | 3 | 2 | 6 | 8 | 4 | 1 | 10 | 5 | 7 | 9 | 13 | 11 | 18 | 14 | 15 | 16 | 21 | 23 | 17 | 12 | 20 | 31 | 22 | 24 | 28 | 26 | 19 | 27 | 29 | 32 | 30 | 25 |
|  | Liberia | 2 | 3 | 7 | 9 | 5 | 1 | 10 | 4 | 6 | 8 | 12 | 11 | 19 | 13 | 16 | 15 | 22 | 23 | 17 | 14 | 21 | 30 | 20 | 25 | 28 | 26 | 18 | 29 | 27 | 32 | 31 | 24 |
|  | Mali | 4 | 5 | 7 | 6 | 2 | 1 | 13 | 10 | 3 | 8 | 11 | 9 | 19 | 16 | 12 | 18 | 25 | 22 | 21 | 15 | 17 | 29 | 23 | 20 | 28 | 24 | 14 | 27 | 31 | 30 | 32 | 26 |
|  | Mauritania | 2 | 3 | 5 | 8 | 7 | 1 | 9 | 4 | 6 | 10 | 13 | 11 | 20 | 12 | 16 | 14 | 23 | 22 | 17 | 15 | 21 | 30 | 19 | 25 | 29 | 27 | 18 | 26 | 28 | 32 | 31 | 24 |
|  | Niger | 2 | 4 | 6 | 8 | 3 | 1 | 9 | 10 | 19 | 5 | 11 | 7 | 18 | 14 | 12 | 16 | 24 | 21 | 15 | 13 | 20 | 29 | 22 | 32 | 26 | 25 | 17 | 27 | 28 | 31 | 30 | 23 |
|  | Nigeria | 2 | 1 | 5 | 7 | 13 | 3 | 10 | 6 | 11 | 8 | 12 | 9 | 20 | 14 | 4 | 17 | 15 | 30 | 24 | 16 | 18 | 31 | 19 | 27 | 26 | 21 | 25 | 32 | 23 | 29 | 28 | 22 |
|  | Sao Tome and Principe | 1 | 3 | 15 | 5 | 7 | 2 | 4 | 6 | 9 | 11 | 13 | 10 | 22 | 8 | 26 | 16 | 27 | 20 | 18 | 14 | 23 | 31 | 17 | 19 | 21 | 28 | 12 | 25 | 29 | 32 | 30 | 24 |
|  | Senegal | 2 | 3 | 6 | 8 | 5 | 1 | 9 | 4 | 12 | 7 | 11 | 10 | 19 | 15 | 14 | 16 | 23 | 22 | 17 | 13 | 21 | 30 | 20 | 25 | 27 | 26 | 18 | 29 | 28 | 32 | 31 | 24 |
|  | Sierra Leone | 2 | 3 | 6 | 9 | 5 | 1 | 10 | 4 | 7 | 8 | 12 | 11 | 18 | 14 | 15 | 16 | 21 | 23 | 17 | 13 | 20 | 31 | 22 | 25 | 28 | 26 | 19 | 27 | 29 | 32 | 30 | 24 |
|  | Togo | 2 | 3 | 6 | 8 | 5 | 1 | 10 | 4 | 7 | 9 | 12 | 11 | 18 | 14 | 15 | 16 | 22 | 23 | 17 | 13 | 21 | 30 | 20 | 25 | 27 | 26 | 19 | 28 | 29 | 32 | 31 | 24 |

Colour intensity and number ranking are assigned according to the rank of absolute number of DALYs of each cancer type among all cancer types. Dark red and number ranking of 1 indicate the highest rank and greatest absolute DALY burden. Dark green and number ranking of 32 indicate the lowest rank and the smallest absolute DALY burden.

## eTable 11. Ranking of the number of incident cases of childhood cancer, AYA cancer, and cancers among the population aged above 39 years at the global level and according to SDI quintile, both sexes, 2019

| **Cancer type** | **Total Incident cases** | **Global rank** | **Low SDI rank** | **Low-middle SDI rank** | **Middle SDI rank** | **High-middle SDI rank** | **High SDI rank** |
| --- | --- | --- | --- | --- | --- | --- | --- |
| Tracheal, bronchus, and lung cancer | 2227116 | 1 | 5 | 3 | 1 | 1 | 2 |
| Colon and rectum cancer | 2089033 | 2 | 8 | 4 | 2 | 2 | 1 |
| Breast cancer | 1832495 | 3 | 2 | 2 | 3 | 3 | 4 |
| Prostate cancer | 1404980 | 4 | 7 | 6 | 6 | 5 | 3 |
| Stomach cancer | 1220798 | 5 | 6 | 5 | 4 | 4 | 5 |
| **AYA cancer** | **1194382** | **6** | **1** | **1** | **5** | **6** | **6** |
| Other malignant neoplasms | 609732 | 7 | 9 | 9 | 9 | 8 | 10 |
| Esophageal cancer | 526475 | 8 | 10 | 11 | 8 | 10 | 15 |
| Pancreatic cancer | 520896 | 9 | 15 | 13 | 11 | 9 | 7 |
| Bladder cancer | 510210 | 10 | 13 | 17 | 12 | 7 | 8 |
| Liver cancer | 505216 | 11 | 12 | 12 | 7 | 13 | 14 |
| Cervical cancer | 446282 | 12 | 3 | 7 | 10 | 15 | 22 |
| Uterine cancer | 415626 | 13 | 18 | 20 | 15 | 11 | 12 |
| Non-Hodgkin lymphoma | 389857 | 14 | 17 | 18 | 16 | 14 | 9 |
| Lip and oral cavity cancer | 342572 | 15 | 11 | 8 | 14 | 18 | 16 |
| Kidney cancer | 338321 | 16 | 24 | 23 | 19 | 12 | 13 |
| Childhood cancer | 291319 | 17 | 4 | 10 | 13 | 22 | 27 |
| Ovarian cancer | 256079 | 18 | 14 | 16 | 17 | 17 | 18 |
| Malignant skin melanoma | 250858 | 19 | 30 | 30 | 29 | 19 | 11 |
| Brain and central nervous system cancer | 246549 | 20 | 21 | 21 | 18 | 16 | 17 |
| Larynx cancer | 204935 | 21 | 16 | 15 | 20 | 20 | 26 |
| Gallbladder and biliary tract cancer | 195370 | 22 | 20 | 19 | 23 | 23 | 20 |
| Thyroid cancer | 184659 | 23 | 22 | 22 | 21 | 24 | 21 |
| Other pharynx cancer | 159798 | 24 | 19 | 14 | 25 | 27 | 23 |
| Multiple myeloma | 152757 | 25 | 23 | 25 | 26 | 25 | 19 |
| Nasopharynx cancer | 146820 | 26 | 26 | 24 | 22 | 21 | 33 |
| Other leukemia | 111529 | 27 | 25 | 26 | 24 | 28 | 30 |
| Chronic lymphoid leukemia | 99204 | 28 | 27 | 28 | 30 | 29 | 25 |
| Acute myeloid leukemia | 91600 | 29 | 29 | 27 | 28 | 30 | 24 |
| Acute lymphoid leukemia | 80670 | 30 | 33 | 33 | 27 | 26 | 29 |
| Chronic myeloid leukemia | 53288 | 31 | 28 | 29 | 33 | 32 | 28 |
| Hodgkin lymphoma | 49508 | 32 | 31 | 31 | 31 | 31 | 31 |
| Mesothelioma | 33044 | 33 | 32 | 32 | 34 | 34 | 32 |
| Testicular cancer | 32859 | 34 | 34 | 34 | 32 | 33 | 34 |

Childhood cancer burden is represented by the total incident cases for a population aged 0-15 years. AYA cancer burden is represented by the total incident cases for a population aged 15-39 years. For adults aged above 39 years, each cancer type is listed separately. Colour intensity and number ranking are assigned according to the rank of incident cases of each cancer type at the global level or at a particular SDI quintile.

## eTable 12. Ranking of the number of deaths of childhood cancer, AYA cancer, and cancers among the population aged above 39 years at the global level and according to SDI quintile, both sexes, 2019

| **Cancer type** | **Total Deaths** | **Global rank** | **Low SDI rank** | **Low-middle SDI rank** | **Middle SDI rank** | **High-middle SDI rank** | **High SDI rank** |
| --- | --- | --- | --- | --- | --- | --- | --- |
| Tracheal, bronchus, and lung cancer | 2017659 | 1 | 3 | 1 | 1 | 1 | 1 |
| Colon and rectum cancer | 1056977 | 2 | 7 | 4 | 3 | 2 | 2 |
| Stomach cancer | 929290 | 3 | 4 | 2 | 2 | 3 | 6 |
| Breast cancer | 657572 | 4 | 2 | 3 | 6 | 4 | 4 |
| Pancreatic cancer | 523498 | 5 | 13 | 12 | 8 | 5 | 3 |
| Esophageal cancer | 491853 | 6 | 9 | 8 | 4 | 6 | 9 |
| Prostate cancer | 485960 | 7 | 6 | 7 | 9 | 7 | 5 |
| Liver cancer | 462714 | 8 | 12 | 11 | 5 | 8 | 7 |
| **AYA cancer** | **396117** | **9** | **1** | **5** | **7** | **9** | **18** |
| Other malignant neoplasms | 328800 | 10 | 10 | 6 | 10 | 10 | 11 |
| Cervical cancer | 253311 | 11 | 5 | 9 | 11 | 16 | 22 |
| Non-Hodgkin lymphoma | 227992 | 12 | 16 | 15 | 13 | 14 | 8 |
| Bladder cancer | 226685 | 13 | 14 | 20 | 15 | 11 | 10 |
| Brain and central nervous system cancer | 197160 | 14 | 20 | 19 | 12 | 12 | 14 |
| Lip and oral cavity cancer | 189018 | 15 | 11 | 10 | 14 | 18 | 19 |
| Ovarian cancer | 188979 | 16 | 17 | 17 | 16 | 15 | 13 |
| Gallbladder and biliary tract cancer | 170051 | 17 | 19 | 16 | 17 | 17 | 15 |
| Kidney cancer | 159708 | 18 | 25 | 23 | 20 | 13 | 12 |
| Larynx cancer | 121106 | 19 | 15 | 14 | 18 | 19 | 27 |
| Multiple myeloma | 111794 | 20 | 22 | 24 | 24 | 20 | 16 |
| Other pharynx cancer | 109847 | 21 | 18 | 13 | 23 | 23 | 24 |
| Childhood cancer | 98834 | 22 | 8 | 18 | 22 | 28 | 31 |
| Other leukemia | 93300 | 23 | 24 | 25 | 19 | 22 | 23 |
| Uterine cancer | 89833 | 24 | 21 | 22 | 25 | 21 | 21 |
| Acute myeloid leukemia | 74756 | 25 | 29 | 27 | 27 | 24 | 17 |
| Nasopharynx cancer | 65093 | 26 | 23 | 21 | 21 | 26 | 29 |
| Malignant skin melanoma | 58306 | 27 | 31 | 30 | 28 | 25 | 20 |
| Chronic lymphoid leukemia | 43603 | 28 | 28 | 28 | 30 | 27 | 25 |
| Thyroid cancer | 42524 | 29 | 26 | 26 | 26 | 29 | 28 |
| Mesothelioma | 28261 | 30 | 32 | 32 | 33 | 30 | 26 |
| Chronic myeloid leukemia | 23011 | 31 | 27 | 29 | 31 | 32 | 30 |
| Acute lymphoid leukemia | 20445 | 32 | 33 | 33 | 29 | 31 | 32 |
| Hodgkin lymphoma | 17730 | 33 | 30 | 31 | 32 | 33 | 33 |
| Testicular cancer | 4963 | 34 | 34 | 34 | 34 | 34 | 34 |

Childhood cancer burden is represented by the total number of deaths for a population aged 0-15 years. AYA cancer burden is represented by the total number of deaths for a population aged 15-39 years. For adults aged above 39 years, each cancer type is listed separately. Colour intensity and number ranking are assigned according to the rank of number of deaths of each cancer type at the global level or at a particular SDI quintile.

## eTable 13. Ranking of the number of DALYs of childhood cancer, AYA cancer, and cancers among population aged above 39 years at the global level and according to SDI quintile, both sexes, 2019

| **Cancer type** | **Total DALYs** | **Global rank** | **Low SDI rank** | **Low-middle SDI rank** | **Middle SDI rank** | **High-middle SDI rank** | **High SDI rank** |
| --- | --- | --- | --- | --- | --- | --- | --- |
| Tracheal, bronchus, and lung cancer | 44455896 | 1 | 5 | 2 | 1 | 1 | 1 |
| **AYA cancer** | **23491284** | **2** | **1** | **1** | **2** | **4** | **8** |
| Colon and rectum cancer | 22613026 | 3 | 7 | 5 | 4 | 2 | 2 |
| Stomach cancer | 20647083 | 4 | 6 | 4 | 3 | 3 | 6 |
| Breast cancer | 18135893 | 5 | 3 | 3 | 5 | 5 | 3 |
| Esophageal cancer | 11322264 | 6 | 8 | 10 | 7 | 7 | 9 |
| Liver cancer | 11207007 | 7 | 12 | 11 | 6 | 8 | 7 |
| Pancreatic cancer | 11128219 | 8 | 13 | 14 | 8 | 6 | 4 |
| Prostate cancer | 8590539 | 9 | 10 | 12 | 12 | 9 | 5 |
| Childhood cancer | 8302464 | 10 | 2 | 6 | 11 | 19 | 25 |
| Other malignant neoplasms | 7865559 | 11 | 9 | 8 | 10 | 10 | 11 |
| Cervical cancer | 7394472 | 12 | 4 | 7 | 9 | 12 | 21 |
| Brain and central nervous system cancer | 5246533 | 13 | 20 | 19 | 13 | 11 | 14 |
| Non-Hodgkin lymphoma | 5232955 | 14 | 16 | 16 | 14 | 16 | 10 |
| Lip and oral cavity cancer | 4899411 | 15 | 11 | 9 | 15 | 17 | 20 |
| Ovarian cancer | 4787371 | 16 | 15 | 17 | 16 | 14 | 15 |
| Bladder cancer | 4268109 | 17 | 17 | 20 | 17 | 13 | 12 |
| Kidney cancer | 3578986 | 18 | 25 | 23 | 22 | 15 | 13 |
| Gallbladder and biliary tract cancer | 3488911 | 19 | 19 | 18 | 18 | 18 | 17 |
| Larynx cancer | 3134033 | 20 | 14 | 15 | 19 | 20 | 26 |
| Other pharynx cancer | 2989599 | 21 | 18 | 13 | 23 | 24 | 23 |
| Multiple myeloma | 2401642 | 22 | 23 | 25 | 25 | 22 | 16 |
| Uterine cancer | 2218859 | 23 | 22 | 22 | 24 | 21 | 22 |
| Other leukemia | 2136811 | 24 | 24 | 24 | 20 | 23 | 24 |
| Nasopharynx cancer | 1937448 | 25 | 21 | 21 | 21 | 25 | 30 |
| Acute myeloid leukemia | 1717800 | 26 | 28 | 27 | 26 | 26 | 18 |
| Malignant skin melanoma | 1423925 | 27 | 31 | 31 | 29 | 27 | 19 |
| Thyroid cancer | 1024054 | 28 | 27 | 26 | 27 | 29 | 29 |
| Chronic lymphoid leukemia | 887088 | 29 | 29 | 29 | 30 | 28 | 28 |
| Mesothelioma | 611739 | 30 | 33 | 32 | 33 | 31 | 27 |
| Acute lymphoid leukemia | 604254 | 31 | 32 | 33 | 28 | 30 | 31 |
| Chronic myeloid leukemia | 588473 | 32 | 26 | 28 | 31 | 33 | 32 |
| Hodgkin lymphoma | 498221 | 33 | 30 | 30 | 32 | 32 | 33 |
| Testicular cancer | 159846 | 34 | 34 | 34 | 34 | 34 | 34 |

Childhood cancer burden is represented by the total DALYs for the population aged 0-15 years. AYA cancer burden is represented by the total DALYs for the population aged 15-39 years. For adults aged above 39 years, each cancer type is listed separately. Colour intensity and number ranking are assigned according to the rank of absolute DALYs of each cancer type at the global level or at a particular SDI quintile.
